# Analysis of Genetic Variation of Rice Straw Characteristics and Its Influence on Biomass

**DOI:** 10.1002/pld3.70134

**Published:** 2026-01-06

**Authors:** Mahta Mohamadiaza, Naser Farrokhi, Asadollah Ahmadikhah, Pär K. Ingvarsson, Mehdi Jahanfar

**Affiliations:** ^1^ Department of Cell and Molecular Biology, Faculty of Life Sciences and Biotechnology Shahid Beheshti University Tehran Iran; ^2^ Department of Plant Biology Swedish University of Agricultural Sciences Uppsala Sweden

**Keywords:** biofuel, cell wall, epistasis, genome‐wide association study (GWAS), haplotype analysis, heritability, lodging resistance, quantitative trait loci (QTLs)

## Abstract

Rice straw is a key source of lignocellulosic biomass. GWAS can be used to identify genetic loci controlling stem morphological traits that influence biomass. This study aimed to investigate the genotypic diversity of rice straw internodes through GWAS, using 34,232 single‐nucleotide polymorphic sites with a minor allelic frequency (MAF) greater than 0.05. Morphological traits (32) were evaluated in 149 rice accessions at the heading stage. Among the 32 measured traits, 26 were found to be significant. GWAS identified 173 significant SNPs located within 64 QTLs with a putative function in biomass production. Among all the putative genes identified, 21 were selected as candidate genes, including *WAK 53a* and *DUF* (*248*, *295, 309, 1740*, *3444, 3464*, *3475*). In general, the identified candidate genes were grouped into five categories: cytoskeletal and transport of cell wall components, growth and development, cell wall biosynthesis, wall‐modifying genes, and regulatory genes. The three major TF groups were WRKY, ERF, and MYB. Haplotype analysis identified seven haplogroups, with five being significant. Path analysis revealed that panicle dry weight (0.64) and internode 3 dry weight (0.57) had the highest positive correlation with biomass. Our findings can be implemented in genome editing methodologies for functional characterization of the candidate genes. This study represents the first comprehensive GWAS of various stem‐related morphological traits in *Oryza sativa* , aiming to identify candidate genes involved in lignocellulosic biomass production and to inform targeted breeding approaches.

## 1 Introduction

Rice ( *Oryza sativa* L.) is the second most produced food grain worldwide, after wheat. According to the Food and Agriculture Organization of the United Nations (FAO 2022), around 760 million tons of rice are produced annually on 164 million ha of arable land. It generates approximately 972 Tg of waste per year, primarily in the form of grain husk and straw (Ramos et al. 2023). Rice straw (RS) is the most abundant agricultural waste, with ~6 T/ha per year (Torregrosa et al. 2021). The annual global production of RS ranges from 370 to 520 million tons, which generates a significant amount of residue after harvest (Van Hung et al. 2020). Efforts have been made to address environmental issues, including post‐harvest straw burning and the population surge of *Chilo suppressalis* (Torregrosa et al. 2021). RS waste represents a significant amount of lignocellulosic biomass that can be converted into energy (heating, electricity generation, and biofuels for transportation), chemical feedstocks in biorefineries (Binod et al. 2010; Cosgrove 2024), construction and insulation materials (Dingcong et al. 2024), and packaging materials (Ramos et al. 2023). RS has also been utilized directly as a biosurfactant (Makkar et al. 2011), a heavy metal absorbent (Kardam et al. 2014; Amer et al. 2017), animal feed, a bed for mushroom culture (Demont et al. 2020), and drug carriers (Yusefi et al. 2020), among others. Due to the high bioavailability of silica in RS (~15% of its dry matter), it has potential applications in other industries (Oladosu et al. 2016), including the use of RS as a scaffold for animal stem cell proliferation and differentiation (our group, unpublished data).

**FIGURE 1:**
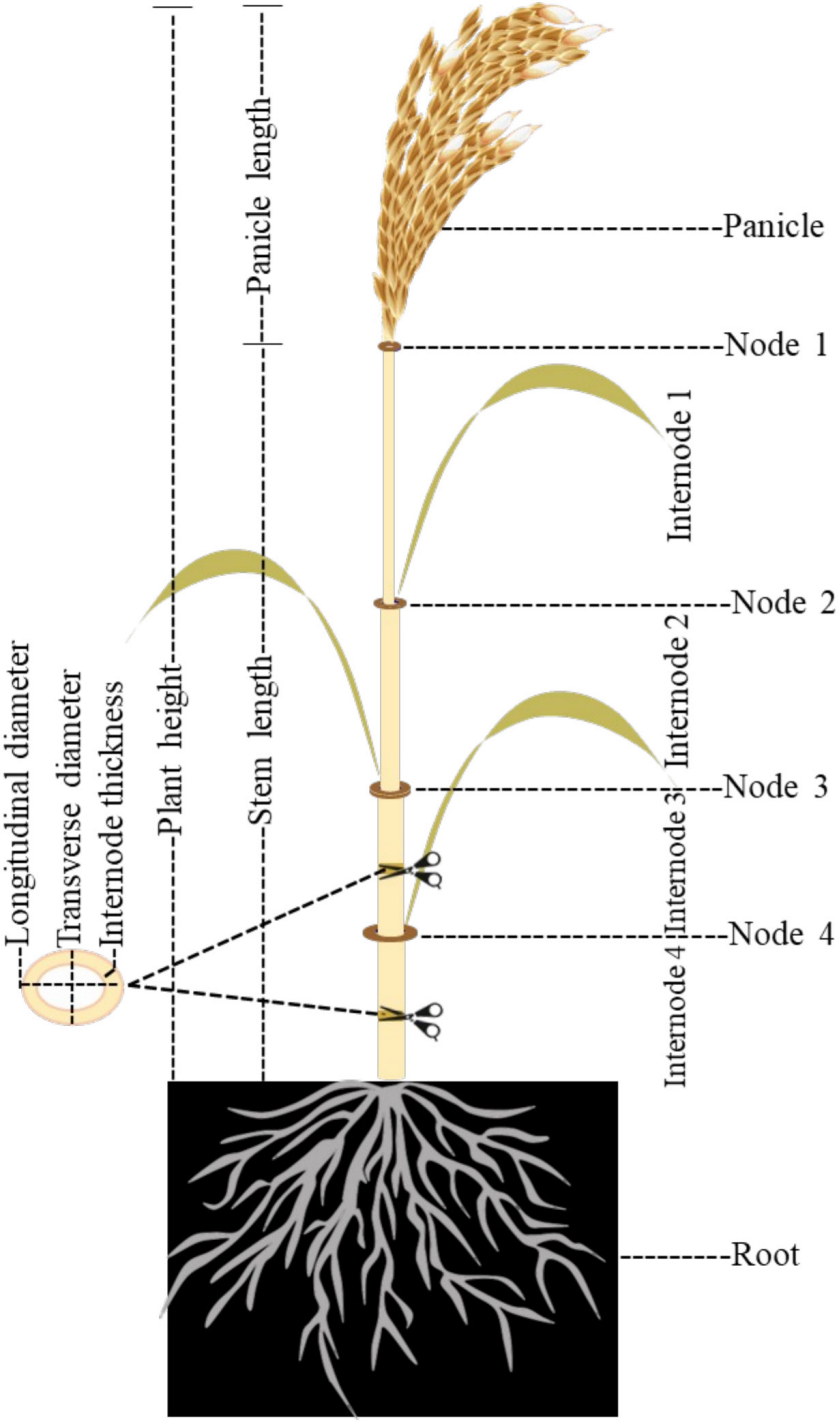
Structure of rice stem. The rice stem consists of nodes and internodes, generally erect and cylindrical, with hollow internodes and solid nodes. The number and length of internodes vary among different varieties. The length of internodes in descending order is: internode 1 > internode 2 > internode 3 > internode 4 from top to bottom. Plant height is defined as panicle length plus stem length; panicle length is measured from the knotting area of the panicle to the tip of the highest grain, excluding the awn, and stem length is the distance from the base of the plant to node 1.

Grass stem growth results from repeated divisions of intercalary meristems located at the nodes (Figure 1). New cells are produced from the node. After elongation, they push the upper nodes upward, and finally, the last node differentiates into a floral meristem (Kapp et al. 2015). The internodes of RS are more suitable for the pulp and paper industry because they have longer fibers and less lignin (Binod et al. 2010). The secondary cell wall in RS has a complex and heterogeneous composition, mainly consisting of cellulose (35%–47%), pectins (~5%), and matrix polysaccharides (18%), including mixed‐linked glucans and lignin (19%–24%) (Siró and Plackett 2010; Panahabadi et al. 2022).

RS represents a structurally important component of the rice plant, contributing to stem mechanical strength and potentially influencing lodging susceptibility (Siró and Plackett 2010; Panahabadi et al. 2022). Lodging results from stem displacement, influenced by plant characteristics and external factors that affect secondary cell walls. These characteristics include both anatomical (our group, unpublished data) and morphological traits measured in this study, which previous reports have associated with lodging‐related mechanics (Mengistie and McDonald 2023). Studies indicate that lignin and cellulose content may be positively correlated with lodging resistance, although most investigations have not directly examined the effects of specific structural features of cellulose and lignin. Understanding these aspects of cell wall biology is crucial for improving biomass utilization and developing plants with valuable traits for the food, agricultural, and bioenergy industries (de Souza et al. 2023).

We performed a GWAS to identify significant QTLs and candidate genes involved in biomass production by quantifying RS node and internode traits (see Table 1). The relationship between the introduced candidate genes and biomass production, mediated by genes involved in growth and secondary cell wall development, will be further established. Once their roles are confirmed, these candidate genes can serve as accurate and reliable markers for screening, selecting, and breeding plants with high biomass production in targeted genome‐based breeding programs.

**TABLE 1:** Thirty‐two morphological traits examined in this study.

Diameter traits (mm)	Weight traits (mg)	Length traits (cm)
Node 1 diameter	Panicle dry weight	Plant height
Node 2 diameter	Shoot dry weight	Stem length
Node 3 diameter	Internode 1 dry weight	Panicle length
Node 4 diameter	Internode 2 dry weight	Number of nodes
Internode 1 diameter	Internode 3 dry weight	Internode 1 length
Internode 2 diameter	Internode 4 dry weight	Internode 2 length
The average diameter of internode 3	Node 1 dry weight	Internode 3 length
The average diameter of Internode 4	Node 2 dry weight	Internode 4 length
Longitudinal diameter of Internode 3	Node 3 dry weight	
Transverse diameter of Internode 3	Biomass (the total weight of the aerial part of the plant) plant part (biomass weight)	
Longitudinal diameter of Internode 4		
Transverse diameter of Internode 4		
Internode 3 thickness (micron)		
Cross‐sectional area of Internode 3		

## 2 Materials and Methods

### 2.1 Plant Materials and Growth Conditions

A total of 149 rice genotypes (out of 282 genotypes obtained from the International Rice Research Institute, Philippines; Table S1) were grown in a completely randomized design with three replicates (25 × 25 cm spacing in plots of 1 × 2 m each) at Shavor Research Station, Khuzestan, Iran (48°27′ E, 31°50′ N) during the 2021–2022 cultivation season. Accessions belonged to TEJ (temperate *japonica* ), IND (indica), AUS (aus), ARO (aromatic), TRJ (tropical japonica), and ADMIX subpopulations. The plots were harvested by hand at maturity over three consecutive days. First, the three plants with the tallest tillers were selected, and the main stems of all three plants were collected for each genotype. Diameter and length traits were measured immediately after harvest in the field. The samples were transported to the laboratory for determination of dry weight at 25°C. To ensure complete drying, samples were randomly selected and weighed over two consecutive days. When no weight change was observed, the dry weight of all samples was measured.

### 2.2 Trait Measurement and Genetic Variability

Traits such as plant height (the distance between the base of the plant and the tip of the highest seed, including spikes), panicle length (from the neck node of the panicle to the tip of the highest seed, excluding spikes), stem length, internode length (first, second, third, and fourth internodes from top to bottom), dry weight of panicle, dry weight of internodes (excluding leaves and leaf sheaths associated with each internode), dry weight of nodes, number of nodes and internodes, diameter of each node and internode, as well as thickness and area of the third internode were recorded (Table 1). Traits were measured in accordance with the standard Rice Evaluation System guidance (Lee et al. 1994).

Considering the elliptical shape (two diameters, Figure 1) of the RS, the average diameter of the basal internodes (third and fourth internodes) was calculated using the following equation (Chuanren et al. 2004)

$$\left(\text{Avgstem diameter}\right)\;\mathrm{Sd}=\frac{D+d}{2}$$

where *Sd* is the average diameter of the internode in millimeters and *D* and *d* represent the longitudinal and transverse diameters, respectively (Figure 1). ImageJ software was used to measure the thickness (in microns) of the third internode (at the midpoint of its length) after preparing a 0.5 cm cross‐section of each sample on the flat surface, which was scanned using a standard Canon flatbed scanner with a resolution of 600 dpi. To obtain a quantitative value for each replicate, measurements were taken from seven points on each sample (due to the bumpy cross‐sectional surface of the stem) and then averaged.

Phenotypic variation among cultivated rice genotypes was assessed by measuring relevant traits in three biological replicates. Descriptive statistics, including mean, standard deviation, and coefficient of variation (CV%), were calculated using SPSS v.16.

To calculate the broad‐sense heritability (H^2^) of each trait, the R packages *heritability* (https://CRAN.R‐project.org/package=heritability) and *rptR* (Stoffel et al. 2017) were used [H^2^b = V_G_/V_P_, H^2^n = V_A_/V_P_, where V_G_ is the genotypic variance, V_P_ is the phenotypic variance, and V_A_ is the additive variance].

### 2.3 GWAS Analysis

Genotyping data obtained from the 44.1 K rice SNP array were downloaded from Gramene (http://gramene.org) for all accessions. The sequencing and development of the hybridization SNP array have been described previously (Zhao et al. 2011). After removing SNPs with a minor allelic frequency (MAF) of less than 0.05, a total of 34,232 high‐quality SNP markers from this dataset were used for GWAS (Bradbury et al. 2007). To describe the population structure and clustering of the studied genotypes based on features such as breed and location, PC1 versus PC2 was plotted as a biplot using the filtered SNPs (34.2 K). The PCA analysis and the corresponding plot (Figure S1) were generated using the *rMVP* package in R (https://CRAN.R‐project.org/package=rMVP) (Yin et al. 2021). The kinship matrix was obtained using the R package *popkin* (https://cran.r‐project.org/web/packages/popkin/index.html), which estimates kinship based on unbiased genetic coancestry from SNP genotype data (34.2 K) (Ochoa and Storey 2021). It was then included as a covariate in the GWAS analysis performed using the *rMVP* package. To identify loci responsible for variation in biomass production in the selected rice accessions, GWAS was conducted using the high‐quality SNP dataset with two models, FarmCPU and MLM, while considering the first three PCs to correct for population structure in R (Kaler et al. 2020). After a thorough comparison of the GWAS results obtained from the two models, which revealed that the outcomes were broadly similar or identical, and considering that most of the stem morphological traits exhibit quantitative inheritance, the multi‐locus model implemented in FarmCPU (Liu, Huang, et al. 2016; Sandhu et al. 2024) was ultimately selected for the GWAS analyses, as it represents the best‐performing approach among those evaluated in the present study. While examining the quantile–quantile (Q‐Q) plots for each trait, SNPs were considered significant when the markers were associated with morphological traits at −log_10_(*p*) ≥ 4 (Bonferroni correction at the 5% level) (Team 2016) and FDR < 0.05 (using the Benjamini–Hochberg method to select more reliable candidate genes and associations, Figure S2) (Ferreira and Zwinderman 2006). Manhattan and Q–Q plots of the GWAS results were generated using the *qqman* package (Turner 2014; Valenzuela et al. 2016).

Significant SNPs identified in the GWAS analyses were mapped to the rice genome (Rice Annotation Project Database; https://rapdb.dna.affrc.go.jp/). Functional annotation of all significant SNPs was performed using SNPEff 4.3 (Cingolani et al. 2012). A reference genome database in SNPEff for *O. sativa* (IRGSP v5.0) was used for annotation. The predicted effects of significant SNPs were categorized by impact as “high” (stop gained, frameshift variants), “moderate” (non‐synonymous substitution, missense variants, in‐frame deletion), “low” (synonymous substitution), and “modifier” (exon variant, upstream gene variant) (Cingolani et al. 2012).

### 2.4 Post‐GWAS Analysis

All downstream analyses were performed on genes associated with significant SNPs using various R packages. Promoter analysis was conducted to determine which of the identified significant SNPs are located in the promoter region of their associated genes using the *IRanges* package in R (https://bioconductor.org/packages/release/bioc/html/IRanges.html). The 1.5‐kb genomic DNA sequences upstream of the start codons of genes (Wang et al. 2015) that were associated with significant SNPs were extracted from the rice genome. These sequences were analyzed using the “New PLACE” database (https://www.dna.affrc.go.jp/PLACE/?action=newplace) to identify potential *cis*‐regulatory elements (CREs) in the promoters of the genes associated with significant SNPs. PlantTFDB v5.0 (https://planttfdb.gao‐lab.org/index.php) was used to identify transcription factors (TFs) among the genes associated with significant SNPs.

To identify key traits affecting biomass and facilitate decisions in indirect selection, path analysis using phenotypic data and epistasis analysis were performed to investigate and model the interactive effects of two or more genes or SNPs on a trait. The *agricolae* package (https://CRAN.R‐project.org/package=agricolae) was used for path analysis, where biomass is modeled as a function of other traits. Analysis of epistasis, which helps identify novel genes and gene combinations and improve understanding of the genetic regulation of complex traits (Ahsan et al. 2019), was conducted using the *FRGEpistasis* package (https://CRAN.R‐project.org/package=FRGEpistasis). All pairwise epistatic effects for all significant SNPs with *p* ≤ 0.05 were assessed.

The expression patterns of the significant SNPs located in genomic regions (gene and promoter) were determined by RNA‐Seq data analysis in 13 different tissues (root, leaf, seedling, shoot, stem, meristem, flower, seed, embryo, endosperm, panicle, female reproductive, and male reproductive), retrieved from the Rice RNA‐Seq database. The expression levels were reported based on FPKM counts (http://ipf.sustech.edu.cn/pub/ricerna/).

To investigate the association between allelic combinations (haplotypes) in a genomic region and a trait, haplotype analysis was performed using the *geneHapR* package (https://CRAN.R‐project.org/package=geneHapR). Haplotype analysis was performed using significant SNPs located within 300 kb of each other (a standard window size according to rice LD decay). The repetitive SNPs were merged into a single haplotype (Zhang et al. 2021).

### 2.5 Candidate Gene Identification

All the significant SNPs [more than four significant SNPs located within 300 kb of each other, known as the SNPs within an LD block] were grouped into QTLs. All genes identified within significant QTL regions were functionally annotated using the Rice Genome Annotation Project (RGAP) database (http://rice.plantbiology.msu.edu/) as well as in the International Rice Genome Sequencing Project (IRGSP) (https://rapdb.dna.affrc.go.jp/). All hypothetical genes and transposable elements were discarded when examining protein‐coding sequences, and a thorough literature review was performed for each gene.

Among all the identified genes, biomass/wall‐related genes were selected based on significant SNPs and functional evidence in rice and other species. These genes were considered as potential candidate genes. STRING (Search Tool for the Retrieval of Interacting Genes—https://string‐db.org/) was employed to construct protein–protein interaction (PPI) networks between genes identified in significant QTLs and candidate genes involved in biomass production and cell wall biology, with the confidence parameter set to a threshold of 0.40. Disconnected nodes were hidden in the network.

## 3 Results

### 3.1 Phenotypic Variation

Phenotypic diversity of RS was estimated for 32 morphological traits using three biological replicates in the 149 rice accessions. All traits showed large variation across 149 rice accessions included in the study, with the greatest and smallest variation observed in the Internode 4 dry weight (CV = 85%) and number of nodes (CV = 14%), respectively (Figure S2).

Broad‐sense heritabilities (*H* ^2^_b_) are reported in Table S3, including the heritability of biomass. The highest heritability (0.97) was obtained for Internode 3 thickness and shoot dry weight. These results indicate a substantial contribution of genotype to the variation in biomass production in RS.

Building on our previous study on the association between rice genotypes and phenotypes (Panahabadi et al. 2022), we further analyzed phenotypic data for 26 of the 32 measured RS traits, selecting those that showed significant associations in GWAS. Our results revealed high variability and heritability across all traits, as well as strong correlations among many of them (Tables S4 and S5). Consistent with Crowell et al. (2016), these traits exhibit a strong genetic component.

### 3.2 PCA and Population Stratification

The PCA plot of genome‐wide SNP data shows the distribution of accessions, which were subsequently grouped into three main clusters based on their genetic background and geographic origin (Figure S1). This is also apparent in the kinship matrix, which summarizes the distribution of pairwise relative relationship coefficients among all the accessions in the association panel based on SNP information (Figure S4). Genetic relatedness was greater within populations than between populations, as expected (Panahabadi et al. 2022).

### 3.3 GWAS Analysis

**FIGURE 2:**
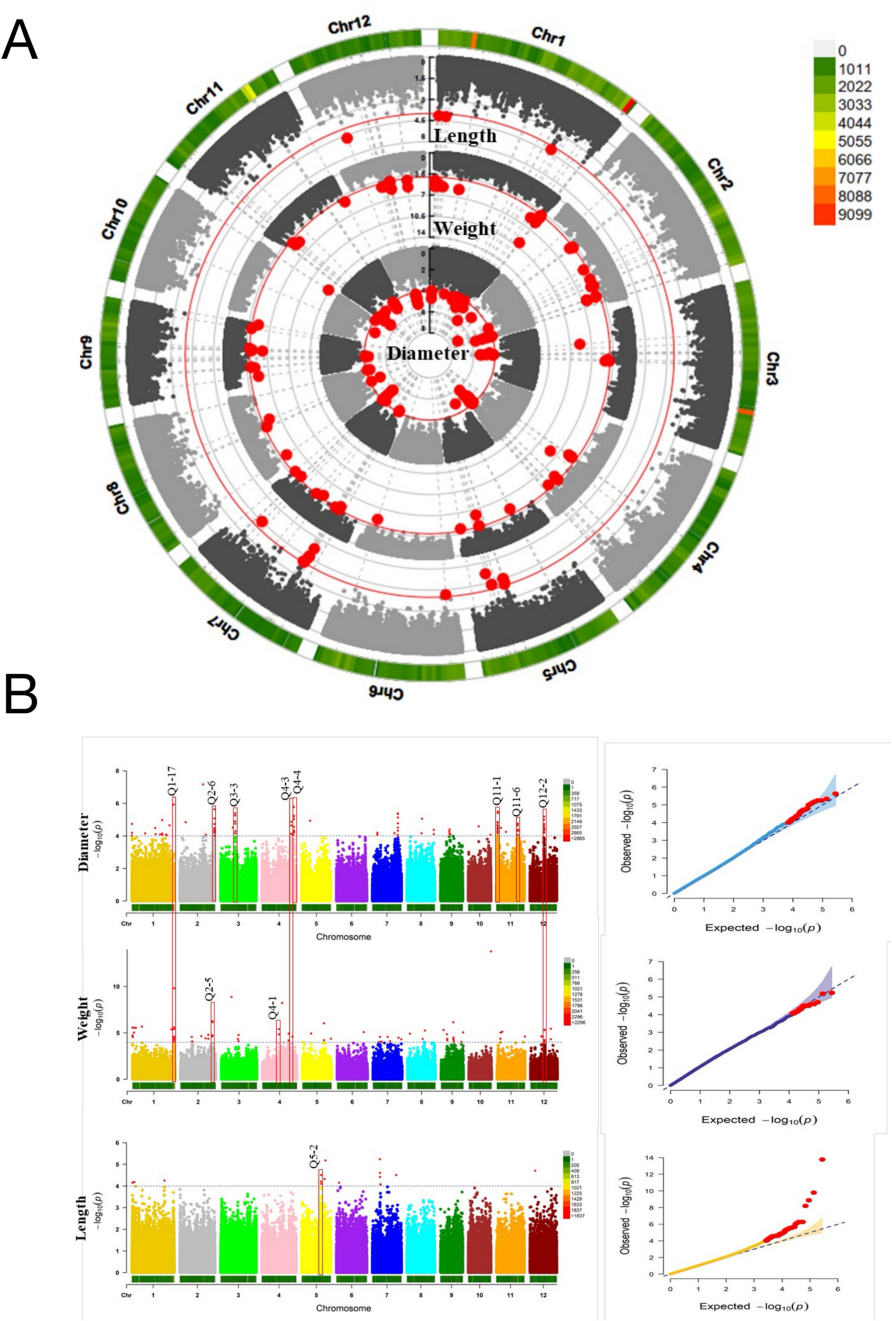
Genome‐wide association mapping of traits measured at the heading stage in field‐grown rice accessions. A) Circular Manhattan plot showing SNPs associated with diameter‐, weight‐, and length‐related traits using the FarmCPU model. SNP density per chromosome is displayed from the center outwards, and each dot represents a SNP. The red solid line denotes the FarmCPU significance threshold (*p* ≤ 1e‐4). B) Rectangular Manhattan plot for diameter‐, weight‐, and length‐related traits. Vertical red boxes indicate QTL locations. Light blue horizontal dotted lines represent the genome‐wide significance threshold (*p* ≤ 1e‐4). The Q‐Q plots show expected versus observed *p*‐values for the FarmCPU model; red diagonal lines indicate the null hypothesis, and the shaded area represents a 95% confidence interval.

Manhattan plots were generated for all traits, but are reported here only for those with significant associations (Figures 2A and S3). The FarmCPU model identified 173 significant marker–trait associations with −log_10_(*p*) ≥ 4 across 26 traits (Table S6). The SNPs with the strongest associations were located on Chromosomes 1, 3, 4, and 10 and were all associated with the Internode 4 dry weight trait (Figure 2B, Table S6). Two genes had missense variants as identified by SNPEff analyses (Cingolani et al. 2012) (Table S7): one conservative variant (F256Y) in *Os02g0820800* (a DNA–binding protein) and one non‐conservative variant (G129D) in *Os03g0375601* (a hypothetical gene).

#### 3.3.1 Analysis of Promoter Regions of Putative Candidate Genes

A promoter analysis was performed using a 1500‐nucleotide window (Wang et al. 2015) upstream of the start codon for all genes to determine whether the corresponding CREs overlapped with significant SNPs identified in the GWAS. Among the 173 significant SNPs (−log_10_(*p*) ≥ 4 and FDR < 0.05) (Figure 2B, Table S8), 96 SNPs (~55% of all the SNPs) were located within gene sequences (39 SNPs in exons; i.e., ~40% of the SNPs; Table S9) and 38 SNPs in the promoter regions (~20% of SNPs; Table S10), based on information extracted from the rice GTF file available on RAPdb (http://rice.plantbiology.msu.edu/). A total of 12 CREs were identified in these promoter regions (Table S10).

#### 3.3.2 Classification of TFs

In plants, TFs play key roles in regulating target gene expression across various signal transduction cascades. According to results from PlantTFDB v5.0, three TFs from the *MYB*, *ERF*, and *WRKY* families were identified in the GWAS signals. Specifically, MYB was associated with shoot dry weight, while ERF and WRKY were associated with internode four length.

*MYB* (*LOC_Os05g37730*, in the proximity to Q5–3) has been implicated in plant growth and development. Analysis of the function of *OsMYB58/63* and its co‐expressed MYB genes (*OsMYB55/61*, *OsMYB55/61‐L*, *OsMYB58/63*, and *OsMYB42/85*), with high expression in culm internodes and nodes, revealed that they co‐express with genes encoding cell wall biosynthetic enzymes in rice (Noda et al. 2015). More specifically, *OsMYB58/63* upregulates *cellulose synthase A7* (*OsCesA7*), a secondary wall‐specific gene in rice (Noda et al. 2015). *OsMYB103L*, a putative MYB master switch of other TFs and a mediator of cellulose biosynthesis and secondary wall formation, binds to the promoters of *CESA4*, *CESA7*, *CESA9*, and *Brittle Culm 1* (*bc1*) and was identified by analysis of a naturally occurring rice variety called culm easily fragile (CEF) (Ye et al. 2015). In a recent study, *OsMYB14* was shown to act as a negative regulator of plant height in rice, and its function is mediated by auxin metabolism and GA biosynthesis (Kim et al. 2024).

*WRKY* family members (*LOC_Os07g40570*, in proximity to Q7–4) have been reported to participate in plant growth, development, metabolism, plant height, and internode and stem elongation (Chen et al. 2017). For instance, the role of *OsWRKY21* (*LOC_Os01g60640*) in regulating internode elongation and plant height in rice has been demonstrated (Wei et al. 2021). Overexpression of *OsWRKY21* resulted in a semi‐dwarf phenotype with shortened internodes (Wei et al. 2021), whereas CRISPR/Cas9 knockout lines displayed the opposite phenotype (Wei et al. 2021). Interestingly, exogenous application of GA_3_ at the seedling stage restored the semi‐dwarf phenotype (Wei et al. 2021).

*AP2/ERF* superfamily (*LOC_Os05g39590*, in close proximity to Q5–4) is a plant‐specific family of TFs (Xie et al. 2022). Overexpression of *OsAP2‐39* in rice transgenic lines resulted in reduced biomass and shortened internodes (55% height reduction), including the uppermost one (Yaish et al. 2010). Ectopic expression of an AP2/ERF TF, *OsEATB*, led to reduced internode length and plant height (Qi et al. 2011). *OsRPH1*, another AP2/ERF, negatively regulates plant height and internode length in rice (Ma et al. 2020). Similarly, *OSDREB2B*, an AP2/ERF TF, has been shown to negatively regulate plant height in rice in coordination with *OsWRKY21* by affecting internode length (Ma et al. 2022).

### 3.4 Post‐GWAS Analyses of Significant SNPs

#### 3.4.1 Path Analysis

**FIGURE 3:**
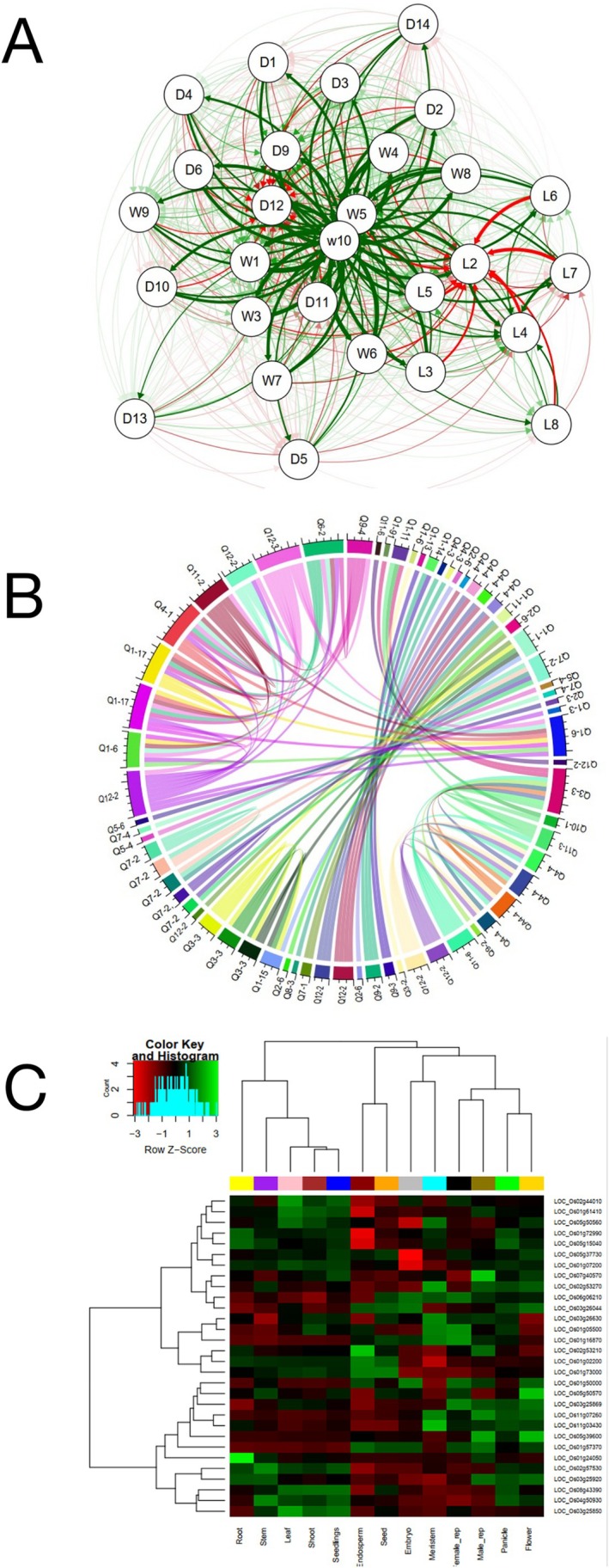
Post‐GWAS analyses, including path analysis, epistasis analysis, and expression data analysis results. A) Results of path analysis. Biomass weight is the dependent variable. Green and red lines indicate positive and negative effects, respectively. The thickness of the lines represents the strength of the connections. D1 (Node 1 diameter), D2 (Node 2 diameter), D3 (Node 3 diameter), D4 (Node 4 diameter), D5 (Internode 1 diameter), D6 (Internode 2 diameter), D7 (average diameter of internode 3), D8 (average diameter of internode 4), D9 (longitudinal diameter of internode 3), D10 (transverse diameter of internode 3), D11 (longitudinal diameter of internode 4), D12 (transverse diameter of internode 4), D13 (internode 3 thickness), D14 (cross‐sectional area of internode 3), W1 (panicle dry weight), W2 (shoot dry weight), W3 (internode 1 dry weight), W4 (internode 2 dry weight), W5 (internode 3 dry weight), W6 (internode 4 dry weight), W7 (node 1 dry weight), W8 (node 2 dry weight), W9 (node 3 dry weight), W10 (biomass), L1 (plant height), L2 (stem length), L3 (panicle length), L4 (number of nodes), L5 (internode 1 length), L6 (internode 2 length), L7 (internode 3 length), L8 (internode 4 length). B) Overview of identified epistatic loci at the heading stage of rice. The Circos plot illustrates SNP–SNP interactions for each trait, comprising 110 significant epistatic interactions (*p* ≤ 0.05). The plot was derived from GWAS results. QTLs with significant epistatic associations are arranged around the circle. The Circlize package was used to visualize the significant interactions. Trait names and their significant epistatic associations are highlighted, and internal lines indicate epistatic interactions between SNPs influencing heading‐stage traits. C) Heatmap showing differential expression of selected candidate genes across various tissues, based on RNA‐Seq data. Green and red colors indicate high and low expression levels, respectively.

Path analysis provides information on the direct and indirect effects of each contributing trait on biomass (Dewey and Lu 1959; Chavan et al. 2012). It also enables breeders to rank the genetic attributes according to their contributions. This analysis was performed on the 26 traits identified as significant in GWAS and serves a descriptive purpose, not being directly integrated into the genetic analyses. We performed a path analysis with biomass weight as the dependent variable. The results showed that the number of nodes had the highest positive direct effect (0.65) on biomass weight, followed by panicle dry weight (0.64) and Internode 3 dry weight (0.57) (Table S11). The largest negative direct effects were observed for stem length (−0.64), followed by the transverse diameter of Internode 4 (−0.33) and Internode 3 length (−0.22). Therefore, these traits may be for biomass production in rice (Figure 3A). In a quantitative trait loci analysis of *Setaria viridis* , a C4 panicoid grass, the number of nodes on the main culm and plant height were shown to be highly correlated with biomass production (Mauro‐Herrera and Doust 2016).

#### 3.4.2 Analysis of Epistatic Interactions

Complex traits in rice are often regulated by epistatic interactions, in addition to the additive effects of individual genes, making the relationship between genotype and phenotype highly complex (Kato and Horibata 2022). A total of 110 epistatic interactions were identified among the significant SNPs (*p* ≤ 0.05). Based on these interactions, a genetic network was subsequently constructed (Figure 3B). The findings of this study show that, among all significant epistatic interactions, nine SNPs were involved in more than four significant interactions. Information about genes associated with these significant SNPs and their epistatic interactions is presented in Table 2. The hub SNPs (i.e., SNPs that interact with several other SNPs) could be biologically important for biomass production and cell wall biology. The molecular basis of these interactions may correspond to the regulation of gene expression and/or the activity of *trans*‐acting elements. Genes with the strongest epistatic interactions were related to shoot dry weight, including interactions between *LOC_Os12g25200* (similar to chloride channel protein) and *LOC_Os01g72990* (protein kinase) involving nine significant SNP‐by‐SNP interactions, and between *LOC_Os01g73000* (zinc finger RING/FYVE/PHD‐type domain‐containing protein) and *LOC_Os01g16870* (similar to Argonaute 4 protein) involving eight significant SNP‐by‐SNP interactions.

**TABLE 2:** Details of significant SNPs identified through GWAS with epistatic interactions.

Traits	SNP (from)	POS	Gene ID	Gene name	Number of epistatic interactions	SNP (to)	POS	Gene ID	Gene name
Transverse diameter of Internode 3	id3007659	Q3–3	LOC_Os03g26630	Similar to SAP domain containing protein	7	id4011513	Q4–4	LOC_Os04g55290	Protein BUD31 homolog 1
						id4011518	Q4–4	LOC_Os04g55360	Similar^a^ to H0811D08.10 protein
						ud4002236	Q4–4	LOC_Os04g55360	Similar to H0811D08.10 protein
						id4011523	Q4–4	LOC_Os04g55360	Similar to H0811D08.10 protein
						id9002755	Q9–2	LOC_Os09g16010	BURP domain‐containing protein 14
						id11001839	Q11–3	LOC_Os11g08670	ATP‐NAD kinase
						id11008620	Q11–6	LOC_Os11g37340	Similar to F‐box domain containing protein
	id11001839	Q11–3	LOC_Os11g08670	ATP‐NAD kinase	4	id1018646	Q1–11	LOC_Os01g53750	Glycoside hydrolase family 17 protein
						id3007659	Q3–3	LOC_Os03g26630	Similar to SAP domain containing protein
						wd10002398	Q10–1	LOC_Os10g21050	Similar to Cytochrome P450 family protein
						id11008620	Q11–6	LOC_Os11g37340	Similar to F‐box domain containing protein
Average diameter of Internode 3, transverse diameter of Internode 3, shoot dry weight	id12005213	Q12–2	LOC_Os12g25200	Similar to chloride channel protein	9	id4011518	Q4–4	LOC_Os04g55360	Similar to H0811D08.10 protein
						ud4002236	Q4–4	LOC_Os04g55360	Similar to H0811D08.10 protein
						id4011523	Q4–4	LOC_Os04g55360	Similar to H0811D08.10 protein
						id1007155	Q1–6	LOC_Os01g16870	Similar to Argonaute 4 protein
						id1007156	Q1–6	LOC_Os01g16870	Similar to Argonaute 4 protein
						id4004869	Q4–1	LOC_Os04g28570	Similar to OSIGBa0092G14.8 protein
						id6015588	Q6–2	LOC_Os06g45380	Similar to Vacuolar sorting receptor 6 precursor (Epidermal growth factor receptor‐like protein 6)
						id12005215	Q12–2	LOC_Os12g25200	Similar to chloride channel protein
						id12006815	Q12–3	None	Hypothetical conserved gene
Longitudinal diameter of Internode 3	id1000027	Q1–1	LOC_Os01g01350	Snf7 family protein	5	id1024348	Q1–15	LOC_Os01g66100	Semi‐dwarf‐1
						id3007489	Q3–3	LOC_Os03g26044	Cellulose synthase‐like A5
						id3007509	Q3–3	LOC_Os03g26044	Cellulose synthase‐like A5
						ud3000834	Q3–3	LOC_Os03g26200	Protein of unknown function DUF248 methyltransferase putative family protein
						wd12002512	Q12–2	LOC_Os12g25400	Hypothetical protein
Shoot dry weight	id12005215	Q12–2	LOC_Os12g25200	Similar to chloride channel protein	9	id1001247	Q1–3	LOC_Os01g03660	Similar to MYBL2
						id1007155	Q1–6	LOC_Os01g16870	Similar to Argonaute 4 protein
						id1007156	Q1–6	LOC_Os01g16870	Similar to Argonaute 4 protein
						dd1001700	Q1–17	LOC_Os01g72990	Protein kinase
						dd1001737	Q1–17	LOC_Os01g73000	Zinc finger RING/FYVE/PHD‐type domain containing protein
						id4004869	Q4–1	LOC_Os04g28570	Similar to OSIGBa0092G14.8 protein
						id6015588	Q6–2	LOC_Os06g45380	Similar to Vacuolar sorting receptor 6 precursor (Epidermal growth factor receptor‐like protein 6)
						id12005213	Q12–2	LOC_Os12g25200	Similar to chloride channel protein

						id12006815	Q12–3	None	Hypothetical conserved gene
	dd1001700	Q1–17	LOC_Os01g72990	Protein kinase	9	id1007155	Q1–6	LOC_Os01g16870	Similar to Argonaute 4 protein
						id1007156	Q1–6	LOC_Os01g16870	Similar to Argonaute 4 protein
						dd1001737	Q1–17	LOC_Os01g73000	Zinc finger RING/FYVE/PHD‐type domain‐containing protein
						id4004869	Q4–1	LOC_Os04g28570	Similar to OSIGBa0092G14.8 protein
						id6015588	Q6–2	LOC_Os06g45380	Similar to Vacuolar sorting receptor 6 precursor (Epidermal growth factor receptor‐like protein 6)
						id9003485	Q9–4	LOC_Os09g21000	Similar to Potassium transporter 13
						id11001392	Q11–2	LOC_Os11g07260	Serine/threonine protein kinase‐related domain‐containing protein
						id12005215	Q12–2	LOC_Os12g25200	Similar to chloride channel protein
						id12006815	Q12–3	None	Hypothetical conserved gene
	dd1001737	Q1–17	LOC_Os01g73000	Zinc finger RING/FYVE/PHD‐type domain containing protein	8	id1007155	Q1–6	LOC_Os01g16870	Similar to Argonaute 4 protein
					id1007156	Q1–6	LOC_Os01g16870	Similar to Argonaute 4 protein

						dd1001700	Q1–17	LOC_Os01g72990	Protein kinase
						id4004869	Q4–1	LOC_Os04g28570	Similar to OSIGBa0092G14.8 protein
						id6015588	Q6–2	LOC_Os06g45380	Similar to Vacuolar sorting receptor 6 precursor (Epidermal growth factor receptor‐like protein 6)
						id12006815	Q12–3	None	Hypothetical conserved gene
	id9003485	Q9–4	LOC_Os09g21000	Similar to Potassium transporter 13	5	dd1001700	Q1–17	LOC_Os01g72990	Protein kinase
						dd1001737	Q1–17	LOC_Os01g73000	Zinc finger RING/FYVE/PHD‐type domain‐containing protein
						id4004869	Q4–1	LOC_Os04g28570	Similar to OSIGBa0092G14.8 protein
						id6015588	Q6–2	LOC_Os06g45380	Similar to Vacuolar sorting receptor 6 precursor (Epidermal growth factor receptor‐like protein 6)
						id12006815	Q12–3	None	Hypothetical conserved gene
	id1007155	Q1–6	LOC_Os01g16870	Similar to Argonaute 4 protein	8	id1007156	Q1–6	LOC_Os01g16870	Similar to Argonaute 4 protein
						dd1001700	Q1–17	LOC_Os01g72990	Protein kinase
					dd1001737	Q1–17	LOC_Os01g73000	Zinc finger RING/FYVE/PHD‐type domain containing protein

						id4004869	Q4–1	LOC_Os04g28570	Similar to OSIGBa0092G14.8 protein
						id11001392	Q11–2	LOC_Os11g07260	Serine/threonine protein kinase‐related domain containing protein
						id12005213	Q12–2	LOC_Os12g25200	Similar to chloride channel protein
						id12005215	Q12–2	LOC_Os12g25200	Similar to chloride channel protein
					id12006815	Q12–3	None	Hypothetical conserved gene

^a^ Similar to refers to genes that have not yet been assigned a specific name or well‐characterized function but are predicted to have a function similar to that of another gene.

The highest number of epistatic interactions (nine interactions) was observed for *LOC_Os12g25200* (affecting the average diameter of Internode 3, transverse diameter of Internode 3, and shoot dry weight) and *LOC_Os01g72990* (affecting shoot dry weight; Table 2). In addition to the epistatic interaction between these two genes, their connections with *LOC_Os01g16870* (*similar to Argonaute 4*, *AGO4*) were noted to be important. Although the relevance of *AGO4* for culm‐related traits has not been established in rice, *OsmiR397b* plays a role in defining plant height by down‐regulating the *OsLAC* (as a target gene) (Zhang et al. 2013) through the action of *OsAGO17* (Zhong et al. 2020).

#### 3.4.3 Expression Profiling

Expression profiling using data from the Plant Public RNA‐seq Database (https://plantrnadb.com/ricerna/) revealed tissue‐specific differential expression patterns of genes harboring significant SNPs. Transcriptomic data were available for 30 non‐repetitive genes, several of which were associated with multiple traits identified in our GWAS analyses (Figure 3C). For instance, *LOC_Os02g57530* and *LOC_Os04g50930* exhibited the highest expression levels in the stem (Figure 3C). Nevertheless, the expression analysis was inconclusive, as none of the candidate genes identified in the GWAS analyses showed stem‐specific expression; their transcripts were abundant in multiple other tissues.

#### 3.4.4 Haplotype Analysis

**FIGURE 4:**
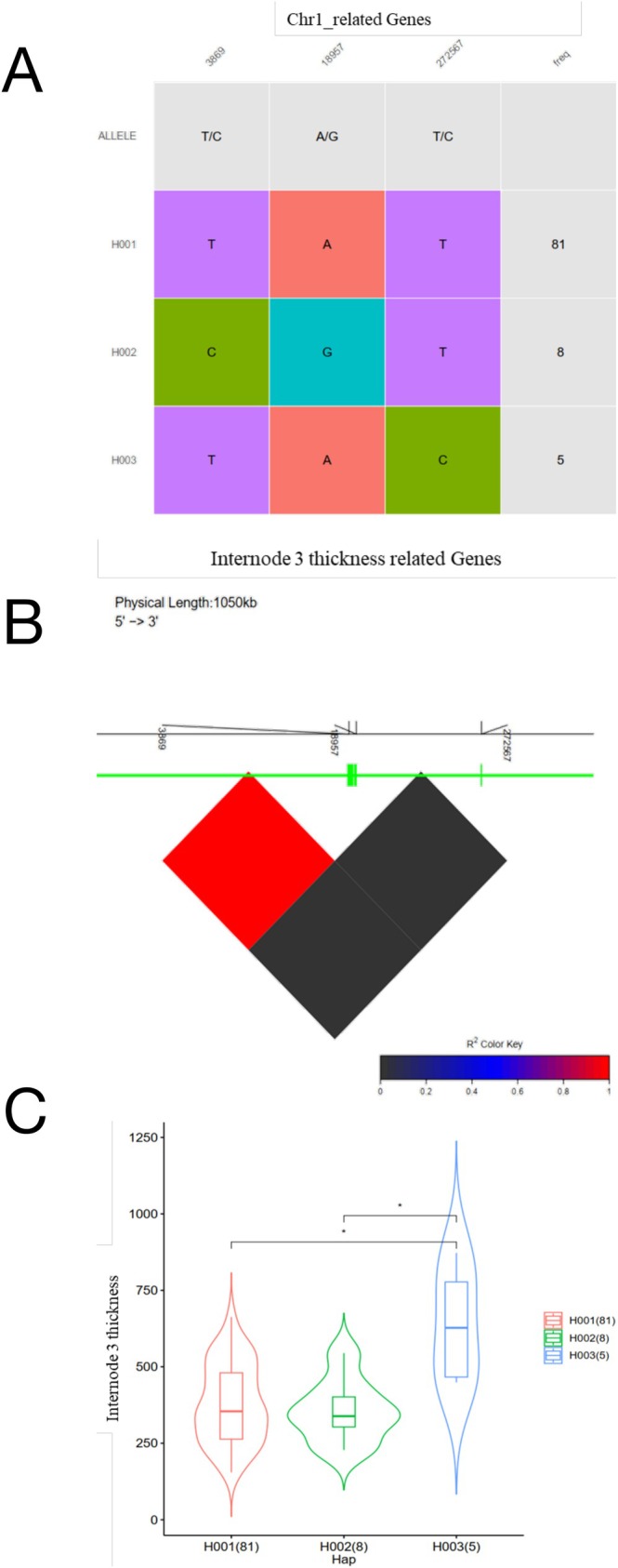
Visualization of haplotype classification, genomic diversity, and evolutionary network of candidate genes on chromosome 1. A) Haplotype classification of trait‐related genes; each line represents a haplotype, and colored columns represent loci. Haplotype frequency is shown in the last column. B) LD‐block visualization of each trait‐related genomic region. The gene model is presented at the top of the plot; the line represents the genomic region, and the rectangles represent exons. The oblique line below the gene model represents variants. The numbers indicate the positions of the variants. The LD block with the color key is at the bottom; red indicates perfect LD, and black indicates no LD. C) Phenotypic comparisons among accessions possessing different haplotypes; * indicates *p* < 0.05.

Haplotypes are linear arrangements of alleles (Judson et al. 2002) and can be inferred from genotyping data (Niu 2004). GWAS have emerged as a powerful approach for identifying underutilized allele/haplotype combinations for crop improvements (Myles et al. 2009). Identification of candidate genes and their functional haplotypes (alleles) for QTLs provides crucial information for determining causal genes. It facilitates further validation and application of the identified QTLs in trait improvement (Islam, Naveed, et al. 2022). Seven haplotype groups were identified on Chromosomes 1, 2, 3, 4, 7, and 11. Following the removal of rare haplotypes, five significant cases remained. The genotypes of each haplotype, their positions and allelic types, the global distributions of the three main haplotypes, linkage disequilibrium, and the evolutionary relationships among haplotypes are depicted in a haplotype network (Figures 4 and S5).

Three haplotypes (*H001*, *H002*, and *H003*) were identified on chromosome 1 in genes related to shoot dry weight and Internode 3 thickness (Figures 4A and S5A‐a). *H001* and *H002* are predominant in the TRJ and IND subpopulations, respectively (Figure S5A‐b). Global distributions of the three main haplotypes in genes related to shoot dry weight and Internode 3 thickness are illustrated in Figure S5A‐c, showing that *H003* and *H002* are predominantly Asian haplotypes. For the SNPs investigated on Chromosome 1, the LD block extended approximately 1 Mb. Two SNP markers within these genomic regions showed substantial LD (Figure 4B), and allelic variation within the haplotypes led to significant differences (*p* ≤ 0.05) between *H001*/*H003* and *H002*/*H003* haplotypes for Internode 3 thickness (Figure 4C). There was no significant difference in shoot dry weight among haplotypes. Among haplotypes on Chromosome 1, three SNP combinations within the *H003* haplotype had a greater effect on Internode 3 thickness than those in other haplotypes (Figure 4C). Therefore, the *H003* haplotype is preferred for selection in breeding programs (Figure 4C).

On Chromosome 2, eight haplotypes were identified, which are related to shoot dry weight, biomass weight, Node 4 diameter, and the average diameter of Internode 4. Their positions on Chromosome 2, haplotype network, and their geographical distribution are presented in Figure S5B‐a–d. For the SNPs investigated on Chromosome 2, the LD block region enabled phenotypic comparisons among samples carrying different haplotypes (Figure S5B‐f). Significant phenotypic differences were observed among haplotypes, indicating that allelic variation at these loci is associated with variation in the trait. For example, variation in haplotype alleles led to significant differences (*p* < 0.01) between the *H004* and *H006* haplotypes for both biomass weight and Node 4 diameter (Figure S5B‐f).

On Chromosome 3, five haplotypes were identified, which are related to the average diameter of Internode 3, longitudinal diameter, and transverse diameter of Internode 3 (Figure S5C‐a–d). For the investigated SNPs on Chromosome 3, three of the five SNP markers are in strong LD (Figure S5C‐e). Except for *H005* for the longitudinal diameter of Internode 3, pairwise interactions between other haplotypes were significant (*p* < 0.05 or *p* < 0.01) for all other traits (Figure S5C‐f). On Chromosome 3, the combination of favorable alleles identified in haplotype *H004* yielded significantly greater phenotypes for all three traits: the average, longitudinal, and transverse diameter of Internode 3 (Figure S5C‐f), and thus, this haplotype can be considered the most favorable option for breeding these traits.

On Chromosome 4, two haplogroups were identified, of which only one showed a significant association. This haplogroup was associated with the cross‐sectional area of Internode 3 (Figure S5Da‐d). On this chromosome, two of the three identified SNP markers were in strong LD (Figure S5D‐e). Significant differences were observed between haplotypes *H001*/*H002* (*p* < 0.05) and *H002*/*H003* (*p* < 0.001) for the cross‐sectional area of Internode 3 (Figure S5D‐f). Three SNP combinations in the H002 haplotype of the cross‐sectional area of Internode 3 had a greater effect than other haplotypes on Chromosome 4 (Figure S5D‐f).

Three haplotypes were reported on Chromosome 11, spanning genes related to Node 2 diameter, Node 3 diameter, Internode 2 diameter, the average of longitudinal diameter of Internode 3, and the cross‐sectional area of Internode 3 (Figure S5Ea‐d). All five SNP markers within this genomic region showed significant LD (Figure S5E‐e) and variation in haplotype alleles led to significant differences (*p* < 0.001) between the *H001*/*H002* haplotypes for five traits: Node 2 diameter, Node 3 diameter, the average diameter of Internode 3, the longitudinal diameter of Internode 3, and the cross‐sectional area of Internode 3. The highest median values in all traits (Node 2 diameter, Node 3 diameter, the cross‐sectional area of Internode 3, the average diameter of Internode 3, the longitudinal diameter of Internode 3, and Internode 2 diameter [*p* < 0.01]) belonged to haplotypes *H001* and *H002* (Figure S5E‐f). Therefore, the *H001* and *H002* haplotypes are preferred in breeding programs.

In summary, our results indicate that if selection for biomass weight and the cross‐sectional area of Internode 3 is considered, the use of major haplotypes located on Chromosomes 2, 4, and 11 is preferred. These favorable alleles can be pyramided into target rice lines through marker‐assisted selection (Sinha et al. 2020). In haplotype‐based allele mining of a MAGIC (multi‐parent advanced generation intercross) rice population for culm length, two genes, *Sd1* (semi‐dwarf 1), which controls gibberellin biosynthesis, and *Cytochrome C*, were reported to exhibit allelic variation between founder classes (Ogawa et al. 2018). Haplotype analysis further revealed that haplotypes with canopy‐lowering effects increase biomass allocation to grain yield by increasing the ratio of panicle weight to leaf and stem weight (Ogawa et al. 2021).

### 3.5 Identification of Candidate Genes

In this study, GWAS‐identified SNPs were mapped to 64 QTLs, of which 11 were significant. A total of 230 gene models were identified in the 11 significant QTL regions defined by significant marker‐trait associations (MTAs). Genes with domains of unknown functions and hypothetical genes were excluded from the final list of genes. This list was further filtered to include only genes with known roles in plant cell wall metabolism or biomass production, including rice (Table S12). We identified 21 rice genes or gene families associated with biomass weight and cell wall biology, including several that have not been previously reported (Table S12). After reviewing the literature, we curated the functional annotations of these genes for further study, which are summarized in Table 3. We further categorized the associated genes into cytoskeletal, vesicle trafficking, and transport of cell wall components, growth and development, cell wall biosynthesis and modification, and regulatory gene groups.

**TABLE 3:** Marker‐trait associations (MTAs)/single nucleotide polymorphisms (SNPs) associated with biomass and cell wall biology candidate genes in *O. sativa* .

Trait	QTLs	Chr	Candidate gene ID	Candidate gene name	Annotation	References
Shoot dry weight, Internode 4 dry weight, Internode 3 thickness	Q1–17	1	LOC_Os01g73170, LOC_Os01g73200	Similar to Peroxidase^b^	Wall polymerization such as lignification, suberization and cross‐linking of cell‐wall constituents	(Passardi et al. 2004)
		1	LOC_Os01g73230	Similar to Syntaxin 61	Transport of cell wall components to the plasma membrane	(Chen et al. 2019)
		1	LOC_Os01g73310	Similar to Actin	Cargo trafficking, cellular motility, apical growth, cell wall formation, and cytoplasmic streaming	(Li, Li, et al. 2014; Yuan et al. 2023)
Shoot dry weight, biomass weight, Node 4 diameter, average diameter of Internode 4, Longitudinal diameter of Internode 4, transverse diameter of Internode 4	Q2–5, 6	2	LOC_Os02g53270, LOC_Os02g53350	Protein of unknown function DUF1740, DUF3464 domain‐containing protein^b^	Regulating the shape and size of the cell during plant growth	(Lin et al. 2020; Tian et al. 2022; Zaynab et al. 2023)
		2	LOC_Os02g53520	Similar to Kinesin heavy chain‐like protein	Lower morphogenesis, trichome development, cell division and the formation of phragmoplasts‐ vesicle trafficking in both primary and secondary cell walls ‐cell wall development, in rapidly dividing cells and the height of rice‐ a brittle culm phenotype in rice‐in cell cycle progression and cell wall composition	(Li et al. 2012; Ali and Yang 2020)
		2	LOC_Os02g53620	Similar to Nuclear transcription factor Y subunit A‐3	Photoperiod‐induced flowering and in regulating rice grain fullness, increased crop yield, rice height regulation, regulating chloroplast biogenesis in rice	(Xu et al. 2022; Kavi Kishor et al. 2023; Zhang, Liu, et al. 2023; Zhang et al. 2024)
Longitudinal diameter of Internode 3, average diameter of Internode 3, transverse diameter of Internode 3	Q3–3	3	LOC_Os03g25990	Similar to Expansin (Expansin2)^a^	In cell wall loosening and cell enlargement and plant growth, the cell extension and developmental processes of cell wall modification,	(Jin et al. 2020)
		3	LOC_Os03g26044	Cellulose synthase‐like A5^a^	Determines plant shape, size, and specific physicochemical properties, in cell wall biogenesis, maintains crop productivity, in the biosynthesis of (1,3;1,4)‐β‐D‐glucans	(Wang et al. 2010; Huang et al. 2022; Sod et al. 2023)
		3	LOC_Os03g26090	GWT1 family protein	In the formation of cell wall structural components, in the development and differentiation of stems and roots, deposit cellulose at the cell wall, affecting plant height, leaf and stem brittleness,	(Tian et al. 2023)
		3	LOC_Os03g26200	Protein of unknown function DUF248 methyltransferase putative family protein		
Shoot dry weight, Internode 3 dry weight, cross‐sectional area of Internode 3, Longitudinal diameter of Internode 4, average diameter of Internode 4, biomass weight, Internode 2 diameter, average diameter of Internode 3, Transverse diameter of Internode 3	Q4–1, Q4–3, Q4–4	4	LOC_Os04g51009, LOC_Os04g51040, LOC_Os04g51030, LOC_Os04g51050	Similar to Wall‐associated kinase‐like protein^b^	Increased cell wall thickening in stem sclerenchyma and reduced cell expansion in the stem, the amplitude of secondary wall deposition, and ultimately, stem height	(Cai et al. 2023)
		4	LOC_Os04g51110	WD40 repeat‐like domain‐containing protein	In many plant development and physiological processes, the first internode elongation in rice	(Ke et al. 2023)
		4	LOC_Os04g51340	Similar to pectin acetylesterase	In the middle lamella and primary cell wall, and plays a critical role in wall plasticity and cellular adhesion, profoundly impact the wall's mechanical properties, in cell wall acetylation,	(Philippe et al. 2017; Kim et al. 2020)
		4	LOC_Os04g51440	Similar to actin filament bundling protein		
		4	LOC_Os04g51450, LOC_Os04g51460, LOC_Os04g51520, LOC_Os04g51510	Similar to xyloglucan endotransglycosylase^b^	Regulation of cell expansion in rice, a more central role in monocotyledon cell wall restructuring, in contributing to internodal stem growth, in the cleavage and rearrangement of the cell wall, affecting its extensibility in plants	(Yokoyama et al. 2004; Hara et al. 2014; Sarmiento‐López et al. 2023; Gao et al. 2024)
Internode 2 length, Internode 2 length	Q5–2	5	LOC_Os05g33610, LOC_Os05g33710	WD40 repeat‐like domain containing protein^b^		
		5	LOC_Os05g33670	Protein of unknown function DUF3475 domain‐containing protein		
		5	LOC_Os05g33730	Soluble gibberellin receptor^a^	Shoot elongation in vegetative tissues, improves biomass production, plant height, stem length, leaf width and flower time, seed germination, stem elongation, leaf expansion, pollen maturation and induction of flowering in rice	(Ueguchi‐Tanaka et al. 2005; Wang, Li, et al. 2017; Kawai et al. 2022)
Node 3 diameter, longitudinal diameter of Internode 3, Internode 2 diameter, Node 2 diameter, cross‐sectional area of Internode 3, average diameter of Internode 3, Node 1 diameter, Transverse diameter of Internode 3,	Q11–1, Q11–6	11	LOC_Os11g03390	FHA domain‐containing protein^a^	Development of root and shoot meristems	(Kashihara et al. 2022; Wang 2023)
		11	LOC_Os11g03410	Similar to secondary cell wall‐related glycosyltransferase family 47	The plant growth phenotype and restored secondary wall thickness, in xylan biosynthesis during secondary cell wall formation	(Zhang et al. 2014)
		11	LOC_Os11g03420	Similar to ZF‐HD homeobox protein (mini zinc finger MIF1)	Modulate biological processes in rice, curl and drooping of rice leaves, an important role in rice morphogenesis, especially the formation and distribution of bulliform cells, the number of cells and length of internodes	(Xu et al. 2014; Islam, Nupur, et al. 2022; Zheng et al. 2022)
		11	LOC_Os11g03580, LOC_Os11g37060, LOC_Os11g36560	Protein of unknown function DUF309, DUF295 family protein^b^		
		11	LOC_Os11g03610, LOC_Os11g37300, LOC_Os11g37390, LOC_Os11g37340	Cyclin‐like F‐box domain‐containing protein^b^	Control of cell proliferation and differentiation, floral transition, panicle and seed development, anther development, regulation of stem development, regulating leaf development	(Jiang et al. 2007; Boycheva et al. 2015; Xu et al. 2021)
		11	LOC_Os11g03660	VQ domain‐containing protein	Grain size, promote flowering	(Hao et al. 2022; Wang et al. 2023)
		11	LOC_Os11g03740	Similar to Pectinesterase inhibitor domain‐containing protein^a^		
		11	LOC_Os11g03794	WD40 repeat‐like domain‐containing protein		
Shoot dry weight, Node 1 diameter, average diameter of Internode 3, longitudinal diameter of Internode 3, Internode 1 dry weight, transverse diameter of Internode 3, Node 2 dry weight, Internode 2 diameter	Q12–2	12	LOC_Os12g25450, LOC_Os12g25490	*O*‐methyltransferase family 2 protein^b^	Reinforcement and growth of the plant's cell wall‐ key roles in stem lignin synthesis	(Karabourniotis et al. 2020; Liang et al. 2022)
		12	LOC_Os12g25690, LOC_Os12g25700	Similar to UDP‐glucose 6‐dehydrogenase^b^	Plant cell wall synthesis, spire and immature xylem development‐keeping cell wall integrity, a dwarf phenotype in *A. thaliana*	(Jia et al. 2021)

^a^ Candidate gene associated with a significant SNP.

^b^ Some candidate gene IDs refer to the same gene and thus share one gene name.

#### 3.5.1 Analysis of PPI Network

Various proteins interact to form a PPI network, which helps to regulate gene expression and biological signal transmission. The PPI network was initially constructed among the 11 significant QTLs (Table S12) and subsequently established among 21 candidate genes (Table 3). The PPI network analysis revealed strong associations between the candidate genes, particularly actin, WD40, and kinesin, and other candidate genes (Figure S6). Mutant analyses of wheat actin demonstrated its role in regulating plant height (Li, Cao, et al. 2023; Xie et al. 2023). Rice early heading date 5 (*Ehd5*), a WD40 domain‐containing protein, has been shown to interact with plant height to positively regulate flowering time (Zhang, Feng, et al. 2023). Similarly, in cucumber (Li et al. 2022) and rice (Wu et al. 2014), kinesins have been shown to influence plant height and internode length through comparative mutant analyses.

## 4 Discussion

RS, a byproduct of rice cultivation, is a SiO_2_‐rich lignocellulosic residue with numerous applications in daily lives (DeVree et al. 2021; Islam et al. 2021). As the main contributor to rice biomass, RS can negatively impact the harvest index depending on vegetative growth. Therefore, breeding programs aim to develop a resilient rice ideotype that balances panicle weight tolerance with high yield. For downstream applications such as biofuel production, a balance between lignin content and polysaccharide constituents of the cell wall is critical for efficient decomposition (Panahabadi et al. 2021), without adversely affecting plant stature.

Despite decades of research on genes and proteins involved in cell wall biosynthesis, hydrolysis, and modification, a clear picture of the functional and regulatory elements that affect this ultrastructure remains lacking (Panahabadi et al. 2021). GWAS has been widely used to map QTLs affecting structural changes in the plant cell wall (Panahabadi et al. 2021). Previous GWAS of the rice stem has so far focused on non‐structural carbohydrates (Wang, Han, et al. 2017), lodging resistance (Yadav et al. 2017; Sowadan et al. 2018; Chigira et al. 2020; Guo et al. 2021; Meng et al. 2021; Nomura et al. 2021; Rashid et al. 2022; Badri et al. 2024), biomass digestibility, lignin content, and saccharification efficiency (Liu, Gómez, et al. 2016; Norton et al. 2018), node number, internode elongation, and plant height (Wei et al. 2021; Malik et al. 2022; Sanchez et al. 2022; Cai et al. 2023), anthocyanin pigmentation (Haghi et al. 2022), silica content (Gowda et al. 2023), and anatomical traits (Li et al. 2024). The candidate genes identified through GWAS analyses can be utilized for the development of hybrid rice varieties, the generation of new transgenic lines, and the functional characterization of the corresponding genes.

Biomass production is a complex trait influenced by multiple interrelated component traits. In this study, we measured multiple traits related to RS and performed GWAS using the FarmCPU model, which identified 21 candidate genes or gene families associated with the regulation of lignocellulosic biomass production. To obtain a deeper understanding of how the identified genes contribute to cell wall biology and, consequently, to lignocellulosic biomass production, we functionally categorized these genes into five classes and examined their potential biological roles. This classification facilitated a more systematic interpretation of gene functions and revealed potential links between distinct cellular processes and biomass accumulation pathways.

### 4.1 Functional Classification of Candidate Gene

#### 4.1.1 Cytoskeletal and the Transport of Cell Wall Components Genes

*Actin* (*LOC_Os04g51440*; located in the close vicinity of Q4–4 QTL; Table 3) has been implicated in the formation and thickening of the secondary cell wall (Oladosu et al. 2016; Panahabadi et al. 2022). Cell expansion requires tight regulation of actin dynamics (Shi et al. 2013). An actin‐binding protein, rice morphology determinant (RMD), has been shown to organize cell microtubules and influence cell growth and morphogenesis (Li, Liang, et al. 2014). Additionally, the *O. sativa* actin‐interacting protein 1 (*OsAIP1*) promotes actin turnover, which facilitates cell elongation and growth (Shi et al. 2013).

*Kinesins* (*LOC_Os02g53520*; located in the close vicinity of Q2–6) play multiple roles in microtubule dynamics and morphogenesis, contributing to the maintenance of cell shapes and mechanical integrity (Ali and Yang 2020). For instance, FRAGILE FIBER1 (*FRA1*; a Kinesin‐4 family member) was initially identified as a regulator of cellulose microfibril alignment in secondary cell walls (Zhang et al. 2010), a processive motor (Ganguly et al. 2017), and as a regulator of vesicle trafficking of non‐cellulosic cell wall components (Kong et al. 2015; Zhu et al. 2015). The *fra1* null mutant exhibits a slightly reduced pectin content and decreased cell wall thickness (Ali and Yang 2020).

*Syntaxin 61* (*SYP61*; *LOC_Os01g73230*; located in the close vicinity of Q1–17) is involved in vesicle trafficking and the transport of cell wall components to the plasma membrane. Mutants of this gene exhibit significant alterations in the cell wall structure and composition (Zhang, Zhou, et al. 2023).

#### 4.1.2 Growth and Development Genes

Wall‐associated kinase (WAK) family members (LOC_Os04g51009, LOC_Os04g51030, LOC_Os04g51040, LOC_Os04g51050 [WAK53a]; located in the close vicinity of Q4–3) are involved in cell wall expansion and thickening and play a crucial role in stem strength, plant morphology, and growth (Verica and He 2002; Cai et al. 2023). For instance, stems of *wak10* mutants exhibit reduced internode length (Cai et al. 2023). In our previous study, several WAK family genes (*OsWAK1*, *50*, *52*, *53b*) were identified as candidate genes based on the analysis of the flanking sequences of significant SNPs associated with lignin content in RS (Panahabadi et al. 2022).

*DUF* (domain of unknown function) family members (*DUF 248*, *295*, *309*,*1740*, *3444*, *3464*, *3475*; located in the close vicinity of QTLs: Q2–5, Q3–3, Q5–2, Q11–1, Q11–6) were identified as candidate genes for the target traits in our study (Table S12). DUF proteins are largely uncharacterized (Zaynab et al. 2023) but have been proposed to play roles in plant growth and development (Ganie et al. 2017; Kaur et al. 2020; Yang, Niu, et al. 2020; Waseem et al. 2021), including the control of lemma and palea development, and leaf rolling in rice (Li et al. 2012; Yang et al. 2016), sugar accumulation (Zhu et al. 2018), regulation of cell shape and size (Lin et al. 2020; Tian et al. 2022), and seedling length (Wang, Wang, Yuan, et al. 2024). Additionally, *DUF248* (*LOC_Os03g26200*; located in the close vicinity of Q3–3) exhibits an epistatic interaction with an *Snf7* family protein (*LOC_Os01g01350*; located in the close vicinity of Q1–1).

The *F‐box* family (*LOC_Os11g03610*, *LOC_Os11g37300*, *LOC_Os11g37340*, *LOC_Os11g37390*; located in the close vicinity of Q11–1 and Q11–6) was identified as candidate genes (Table S12). Some members from this large F‐box gene family (Li et al. 2020) have been shown to regulate plant growth and development (Jain et al. 2007; Kuroda et al. 2012; Carbonnel et al. 2020; Xu et al. 2021). Among the F‐box genes identified in this study, *LOC_Os11g37340* exhibits epistatic interactions with genes encoding a *SAP domain‐containing protein* and *ATP‐NAD kinase* (Table 2).

*Gibberellin receptor* (*LOC_Os05g33730*; located in the close vicinity of Q5–2) promotes plant elongation and enhances biomass production across plant species (Miao et al. 2020; Kawai et al. 2022). Appropriate levels of gibberellins (GAs) in rice stimulate shoot elongation, thereby regulating plant height (Kawai et al. 2022).

Forkhead‐associated (FHA) domain protein (*LOC_Os11g03390*; located in the close vicinity of Q11–1) is a small, well‐characterized protein module. *OsFHA1* is a nuclear‐localized protein (Kashihara et al. 2022). In *Arabidopsis thaliana* , *AtFHA1* is expressed at low levels in roots and vascular tissues of the stem during specific developmental stages (Ahn et al. 2003). DDL, another FHA‐domain protein in *Arabidopsis* , has been shown that *ddl* mutants exhibit pleiotropic growth defects, including plant dwarfism (Wang 2023).

#### 4.1.3 Cell Wall Biosynthetic Genes

*UDP‐glucose dehydrogenase* (UGD: *LOC_Os12g25690*, *LOC_Os12g25700*; located in close vicinity of Q12–2) is a cell wall‐specific biochemical precursor‐converting enzyme that generates UDP‐glucuronic, the substrate for glycosyltransferases that predominantly synthesize pectin (Endres and Tenhaken 2011; Reboul et al. 2011; Jia et al. 2021). UGD enzymes are crucial for maintaining cell wall integrity. In *A. thaliana* , *ugd2,3* double mutants exhibit a dwarf phenotype (Reboul et al. 2011; Kawasaki et al. 2021).

*CslA5* (*LOC_Os03g26044*; located in the close vicinity of Q3–3), a cellulose synthase‐like enzyme, was identified in this study, consistent with our previous report (Panahabadi et al. 2022). In *O. glaberrima* stunted by virus infection, a member of the *CslA9* family (cellulose synthase‐like A9) showed significantly reduced expression (Budot et al. 2014), suggesting a putative role in defining the RS characteristics and plant height. *CslA5* also exhibited an epistatic interaction with a Snf7 family protein (*LOC_Os01g01350*; located in the close vicinity of Q1–1; Table 2).

O‐methyltransferase (OMT) family members (LOC_Os12g25450, LOC_Os12g25490) are versatile enzymes involved in the biosynthetic pathways of phenolics and flavonoids (Li, Li, et al. 2021; Li, Sun, et al. 2021). Phenolics serve to protect and reinforce cellulose fibers (Karabourniotis et al. 2020) and participate in the synthesis of lignins from phenylpropanoids (Bugos et al. 1991; Liang et al. 2022), thereby enhancing cell wall stiffness and providing mechanical strength to the stem (Barros et al. 2015).

GT47 glycosyltransferase family (LOC_Os11g03410; located in the close vicinity of Q11–1) is a homolog of *IRX10* (IRREGULAR XYLEM10). *IRX10L* and *OsGT47A* are highly expressed in rice stem and play critical roles in secondary wall thickening via xylan synthesis (Zhang et al. 2014). These genes are named *IRX* because they are associated with irregular xylem (*irx*) mutants that exhibit secondary cell wall deficiencies (Turner and Somerville 1997). The IRX family includes members of GT47 (FRA8[IRX7]/F8H[IRX7L]) (Wu et al. 2010). Secondary cell walls are the major form of biomass, providing an important source of renewable and sustainable energy in the form of polysaccharides. The presence of lignin and xylan in the secondary cell wall increases the difficulty of cellulose degradation and negatively affects the utilization of plant biomass energy. Xylans, the main components of cell wall matrix polysaccharides, play a critical structural role through interactions with cellulose microfibrils (Scheller and Ulvskov 2010).

#### 4.1.4 Cell Wall‐Modifying Genes

*Expansins* (*LOC_Os03g25990*; located in the close vicinity of Q3–3) are cell wall‐related proteins involved in wall loosening and cell enlargement in a pH‐dependent manner (Cosgrove 2000). They have been implicated in controlling stem mechanical strength (Marowa et al. 2016; Cosgrove 2021) and play important roles in plant growth (Guo YaoMin et al. 2016; Jin et al. 2020; Yang, Zhang, et al. 2020).

*Peroxidases* (*LOC_Os01g73170*, *LOC_Os01g73200*; located in the close vicinity of Q1–17), particularly the secretory types, are involved in cell wall metabolism, suberization, lignification, and cross‐linking of cell‐wall constituents (Kidwai et al. 2020; Zhang, Zhou, et al. 2023). They polymerize hydroxycinnamic acid and its derivatives to generate phenoxy radicals, which can be deposited on the extracellular surface to strengthen the cell wall and construct xylem vessels (Pandey et al. 2017). Furthermore, it has been proposed that they contribute to cell elongation via auxin oxidation and hydroxyl radical generation, which together loosen or stiffen the cell wall (Shigeto and Tsutsumi 2016).

Xyloglucan endotransglucosylase/hydrolases (XTHs: LOC_Os01g73310, LOC_Os04g51450; located in the close vicinity of Q1–17 and Q4–3), a subfamily of the GH16 family with roles in cell wall synthesis and remodeling for greater extensibility (Li, Hua, et al. 2023; Stratilová et al. 2023), represents loci associated with the cross‐sectional area of Internode 3 and biomass weight. Recently, we reported their involvement in the metabolism of cellular polysaccharides and glucans (Panahabadi et al. 2022).

*Pectin acetylesterase* (PAE: *LOC_Os04g51340*; located in the close vicinity of Q4–3) modulates the degree of pectin acetylation by cleaving the acetyl ester bonds (Philippe et al. 2017), presumably reducing the porosity of the gel‐like pectin structure and enhancing cell wall rigidity.

#### 4.1.5 Regulatory Genes

*WD40* proteins (*LOC_Os04g51110*, *LOC_Os05g33710, LOC_Os05g33610* and *LOC_Os11g03794*; located in the close vicinity of Q4–3, Q5–2 and Q11–1) belong to a transcription factor family that plays important regulatory roles in plant development and physiological processes (Kwantes and Wichard 2022). For instance, rice *OsLIS‐L1*, containing WD40 motifs, has been implicated in first internode elongation (Gao et al. 2012).

*Nuclear transcription factor Y* family (NF‐Y: *LOC_Os02g53620*; located in the close vicinity of Q2–6) displays substantial functional diversity through the regulation of cell proliferation (Kavi Kishor et al. 2023). In rice, *OsNF‐YB11* suppresses the expression of flowering‐related genes, *NF‐YB1* regulates rice grain filling, and *NF‐YC12* controls endosperm growth (Zhang et al. 2024). In other crops, *NF‐YB* has been reported to play a significant role in determining yield under drought or chronic water scarcity (Yu et al. 2021) and to enhance biomass accumulation in forest species (Zhang, Liu, et al. 2023).

*Zinc finger‐homeodomain proteins* (ZF‐HD/ZHD; *LOC_Os11g03420*; located in the close vicinity of Q11–1) are TFs involved in biological processes (Islam, Nupur, et al. 2022). In rice, overexpression of *OsZF‐HD1* led to a curly‐drooping leaf phenotype (Zheng et al. 2022). *OsZHD1* plays an important role in the formation and distribution of bulliform cells. Overexpression of *OsZHD1* produced pleiotropic phenotypes, including semi‐dwarf stature, shorter roots, a smaller number of tillers, and reduced panicle length. In addition, scanning electron microscopy (SEM) revealed that the reduction in cell number accounted for the shorter internodes in the mutant lines (Xu et al. 2014).

Valine‐glutamine (VQ)‐motif‐containing proteins (LOC_Os11g03660; located in the close vicinity of Q11–6) act as transcriptional regulatory cofactors and play a crucial role in regulating various physiological and biochemical processes in plants (Wang et al. 2023). Among the interacting proteins of VQ, *WRKY* TFs are the most prominent (Dong et al. 2022). *OsVQ1* interacts with *OsMPK6* and enhances the expression of genes that promote flowering (Wang et al. 2021).

### 4.2 Potential Links to Lodging‐Related Pathways

Stem morphological traits such as rind thickness and stem diameter are key determinants of stem mechanical strength and lodging resistance in cereal crops including maize, rice, and wheat (Robertson et al. 2017; Shah et al. 2019; Stubbs, McMahan, et al. 2020; Stubbs, Seegmiller, et al. 2020; Li et al. 2024). Previous studies have reported the important contribution of the third and fourth internodes to lodging resistance in rice (Hoshikawa and Wang 1990). Because cell wall composition and structure are central to both mechanical reinforcement and biomass saccharification efficiency, modification in cell wall–related pathways has been proposed as a potential strategy to improve both lodging resistance and biomass utilization (Wang, Wang, Yan, et al. 2024). Although the present study did not directly measure lodging resistance, several candidate genes identified through GWAS were associated with morphological traits of the third and fourth internodes (Table 3), which have been implicated in lodging‐related mechanics in previous reports. Therefore, these genes may participate in biological processes relevant to stem stability; however, further functional validation and direct measurement of lodging indices are required to establish causal relationships.

Taken together, our findings highlight genetic components associated with lignocellulosic biomass production and suggest potential connections to pathways influencing stem structural integrity. By integrating GWAS using the FarmCPU model with functional categorization, we identified key gene families potentially involved in cell wall biosynthesis, structural reinforcement, and biomass accumulation. Although these associations indicate possible overlap between biomass‐related pathways and lodging‐related mechanisms, confirming their functional roles in lodging resistance will require additional targeted studies.

## Author Contributions

MM carried out the harvest, sampled and measured the traits, did the initial analysis and drafted the initial manuscript. NF conceptualised the research, planned the experiments, supervised the study, and read and edited the manuscript. AA co‐supervised the research, developed code required for data analysis, helped with the analyses, and read and edited the manuscript. PKI advised on research, read and edited the manuscript; MJ advised on research, read and edited the manuscript.

## Funding

The authors received no specific funding for this work.

## Conflicts of Interest

All authors declare that they have no conflicts of interest.

## Peer Review

The peer review history for this article is available in the Supporting Information for this article.

## Supporting information


**Data S1:** Peer Review.

**Figure S1:** Population structure of rice association panel. Principal components analysis (PCA) was performed on genome‐wide SNP data. The plot of the first two principal components (PC1 and PC2) shows the distribution of accessions. The three major clusters were determined based on genetic background and geographic origin, and are indicated by different colors. PCA was used to visualize variation, but cluster assignments were based on these additional criteria.
**Figure S2:** Phenotypic distribution of individual traits.
**Figure S3:** Genome‐wide association mapping of traits measured at the heading stage in field‐grown rice accessions. Manhattan (left) and Q‐Q (right) plots based on MLM and FarmCPU models for all traits. The red horizontal dashed lines indicate the genome‐wide significance thresholds.
**Figure S4:** Phylogenetic tree represented as a kinship plot, efficiently separating the 149 accessions into five major geographical subpopulation clusters: TEJ (Temperate japonica), IND (indica), AUS (aus), TRJ (Tropical japonica), and ADMIX. Green indicates the highest correlation between pairs of individuals, while blue indicates the lowest. A hierarchical clustering tree based on pairwise kinship values for all accessions is shown along the top and left axes.
**Figure S5:** Visualization of genomic diversity and evolutionary network of candidate genes on chromosomes 1 (A), 2 (B), 3 (C), 4 (D), and 11 (E). a) Visualization of variant positions in candidate genes; the black line represents intergenic regions, and rectangles represent exons. Flags indicate variants, and the coordinates with alleles in parentheses are displayed above the gene model. Two or more transcripts are shown in different colors. b) Trait‐related gene haplotype network. Each circle represents a haplotype, and its size indicates the number of accessions. The pies in different colors represent the frequency of different rice subpopulations in each haplotype. Symbols on the lines between haplotypes indicate the number of variants. c) Geo‐distribution of major haplotypes of *chr1_ related genes* on chromosome 1, *chr2_ related genes*, *chr3_ related genes* on chromosome 3, *chr4_ related genes* on chromosome 4, and *chr11_ related genes* on chromosome 11. Circle size represents accession counts, and the pies in different colors represent the proportion of classified haplotype categories for relevant accessions derived from different eco‐regions. The Arabic numeral inside each circle indicates the number of accessions at that location. d) Haplotype classification of trait‐related genes; each line represents a haplotype, and colored columns represent loci. Haplotype frequency is shown in the last column. e) LD‐block visualization of each trait‐related genomic region. The gene model is presented at the top of the plot; the line represents the genomic region, and the rectangles represent exons. The oblique line below the gene model represents variants. The numbers indicate the positions of the variants. The LD‐block with the color key lies at the bottom, where red indicates perfect LD and black indicates no LD. f) Phenotypic comparisons among accessions possessing different haplotypes; * indicates *p* < 0.05, ** indicates *p* < 0.01, *** indicates *p* < 0.001.
**Figure S6:** PPI network: A) Network among all 230 SNP‐associated proteins within the identified QTLs. B) Network among the three candidate genes with the highest number of interactions. Interactions were derived from high‐throughput experiments and curated databases with medium confidence (score ≥ 0.40). Nodes represent proteins, edges indicate interactions, line thickness reflects interaction strength, and colored lines denote different interaction types. Gene nodes with ribbon structures indicate the presence of 3D structural information.

**Table S1:** Information of rice genotypes examined in the study.

**Table S2:** Estimating the heritability of the traits.

**Table S3:** Descriptive statistics for 32 morphological traits.

**Table S4:** The characteristics of significant SNPs extracted from GWAS.

**Table S5:** Examining the significance threshold with the FDR.

**Table S6:** Characteristics of the SNPs in the exonic/intronic regions of the identified genes.

**Table S7:** The presence/absence of *cis* elements in the gene promoters.

**Table S8:** SNPEff analysis results.

**Table S9:** pld370134‐sup‐0011‐Table_S9.pdf. **QTLs.** The list of 24 candidate genes.

**Table S10:** Identification of QTLs with pleiotropic effects (pQTLs).

**Table S11:** Path coefficient analysis showing direct (colorful) and indirect effects of various traits in biomass.

**Table S12:** Correlation coefficient analysis between the traits.

## Data Availability

Data used is available in the supplementary materials or from the authors upon request.
