# Supplementary material for: Analysis of Genetic Variation of Rice Straw Characteristics and Its Influence on Biomass
Source: Plant Direct. 2026 Jan 6;10(1):e70134. doi: 10.1002/pld3.70134 (PMC12771682; doi:10.1002/pld3.70134)
Supplement: Supplementary file 2 — Figure S1: Population structure of rice association panel. Principal components analysis (PCA) was performed on genome‐wide SNP data. The plot of the first two principal components (PC1 and PC2) shows the distribution of accessions. The three major clusters were determined based on genetic background and geographic origin, and are indicated by different colors. PCA was used to visualize variation, but cluster assignments were based on these additional criteria. Figure S2: Phenotypic distribution of individual traits. Figure S3: Genome‐wide association mapping of traits measured at the heading stage in field‐grown rice accessions. Manhattan (left) and Q‐Q (right) plots based on MLM and FarmCPU models for all traits. The red horizontal dashed lines indicate the genome‐wide significance thresholds. Figure S4: Phylogenetic tree represented as a kinship plot, efficiently separating the 149 accessions into five major geographical subpopulation clusters: TEJ (Temperate japonica), IND (indica), AUS (aus), TRJ (Tropical japonica), and ADMIX. Green indicates the highest correlation between pairs of individuals, while blue indicates the lowest. A hierarchical clustering tree based on pairwise kinship values for all accessions is shown along the top and left axes. Figure S5: Visualization of genomic diversity and evolutionary network of candidate genes on chromosomes 1 (A), 2 (B), 3 (C), 4 (D), and 11 (E). a) Visualization of variant positions in candidate genes; the black line represents intergenic regions, and rectangles represent exons. Flags indicate variants, and the coordinates with alleles in parentheses are displayed above the gene model. Two or more transcripts are shown in different colors. b) Trait‐related gene haplotype network. Each circle represents a haplotype, and its size indicates the number of accessions. The pies in different colors represent the frequency of different rice subpopulations in each haplotype. Symbols on the lines between haplotypes indicate th [file PLD3-10-e70134-s011.docx]

# *Supplementary Figures*


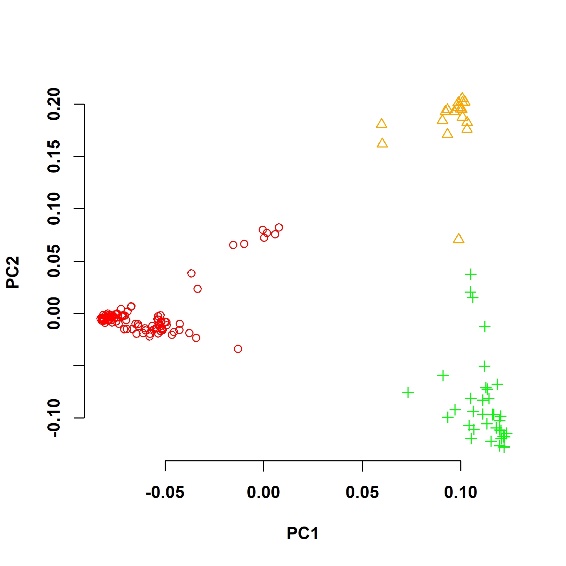


FIGURE S1| Population structure of rice association panel. Principal components analysis (PCA) was performed on genome-wide SNP data. The plot of the first two principal components (PC1 and PC2) shows the distribution of accessions. The three major clusters were determined based on genetic background and geographic origin, and are indicated by different colors. PCA was used to visualize variation, but cluster assignments were based on these additional criteria.

| 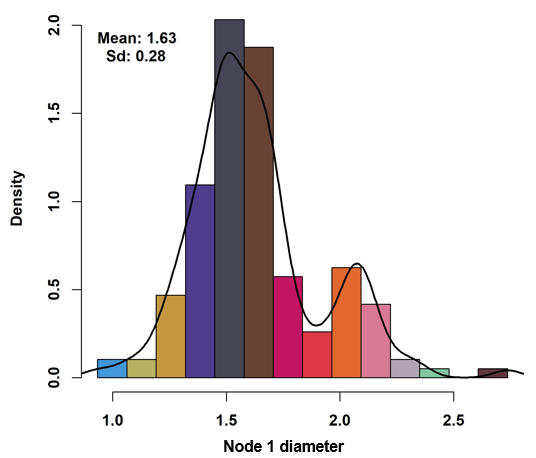 | 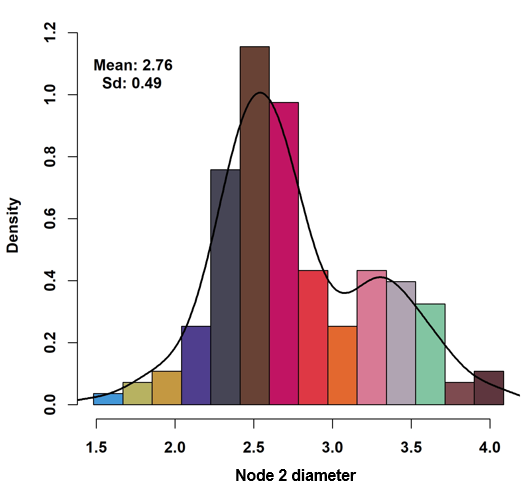 | 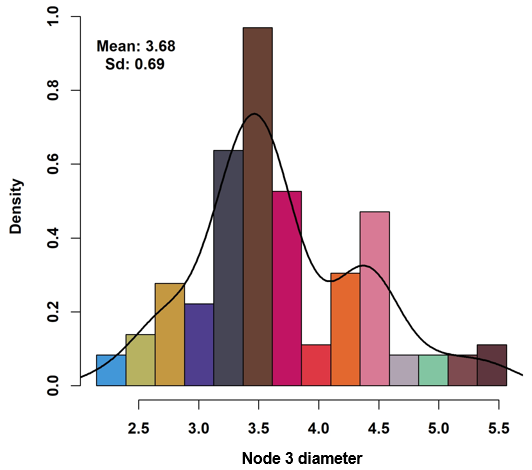 | 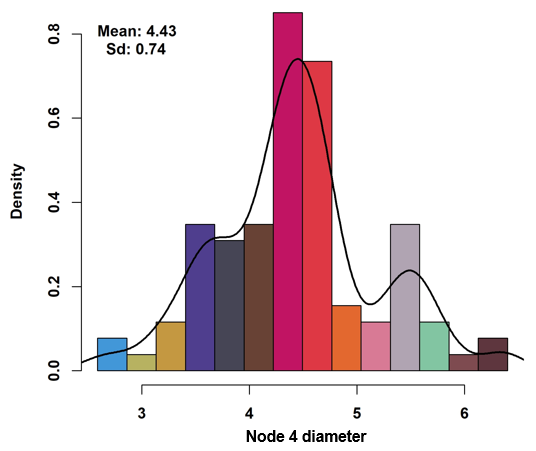 |
| --- | --- | --- | --- |
| 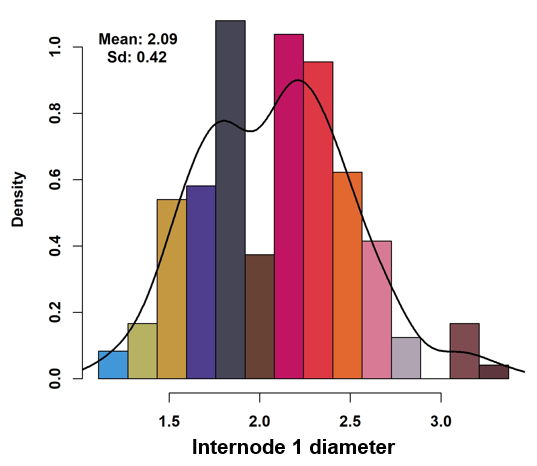 | 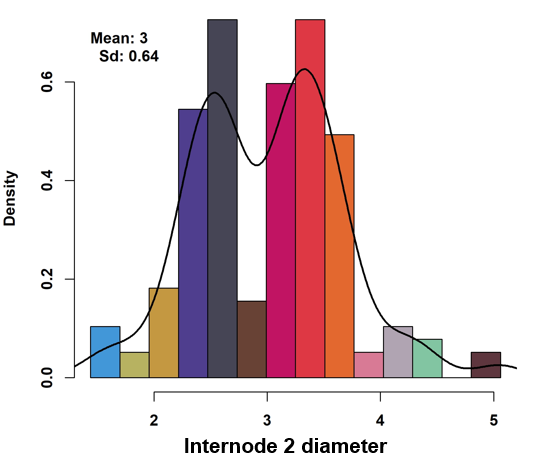 | 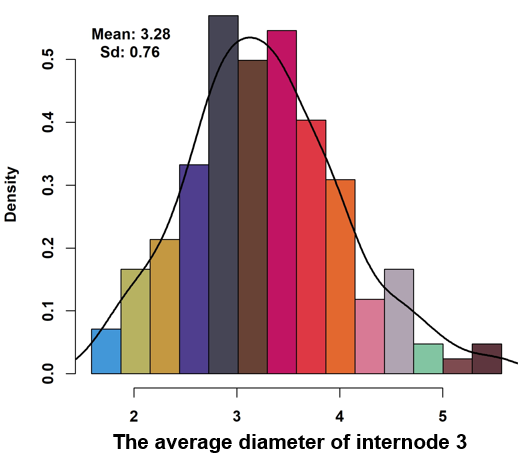 | 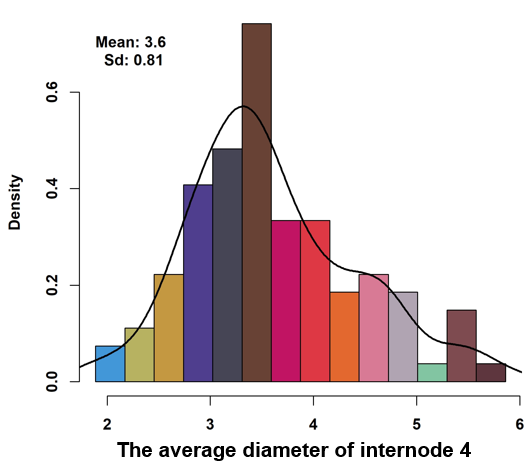 |
| 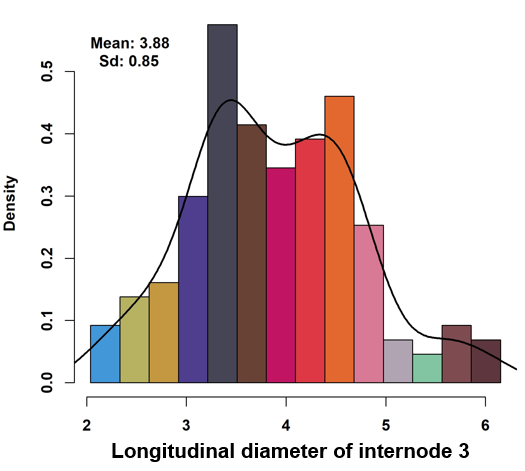 | 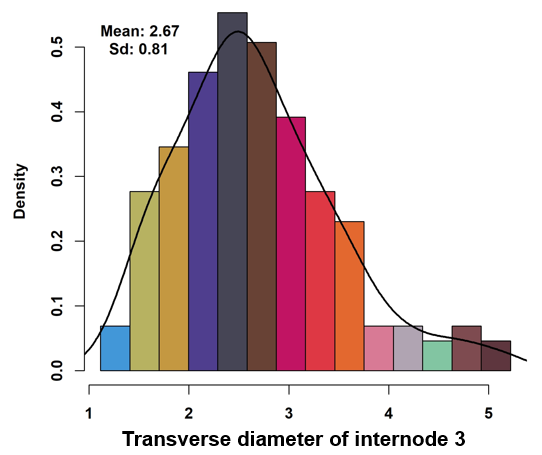 | 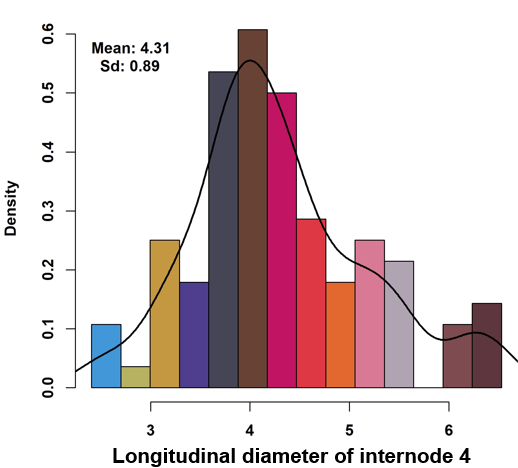 | 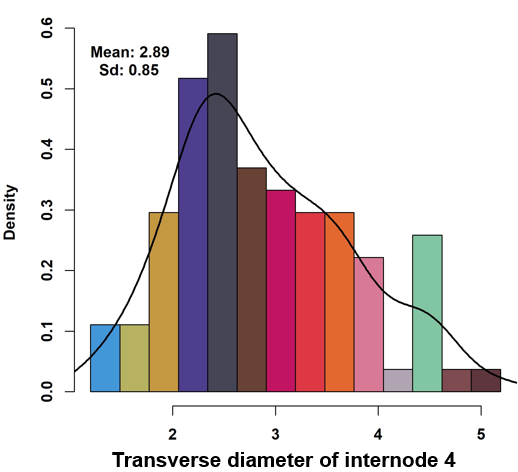 |
| 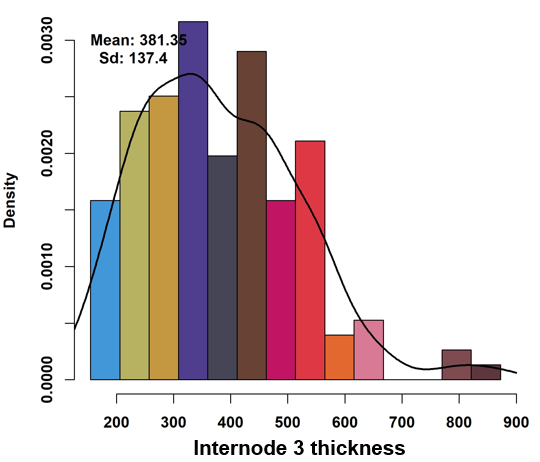 | 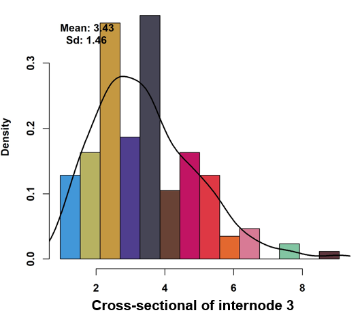 | 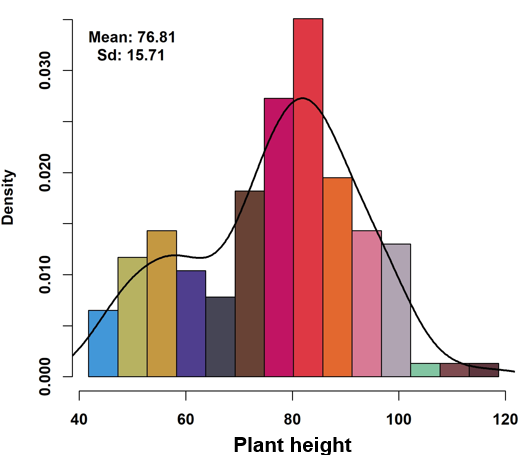 | 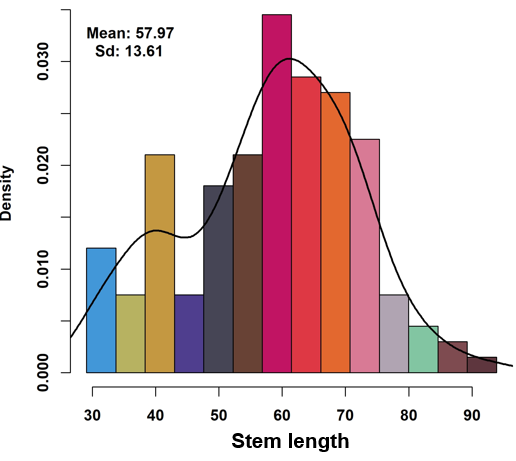 |
| 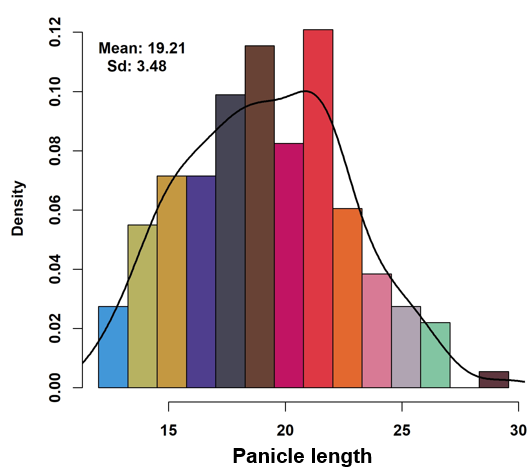 | 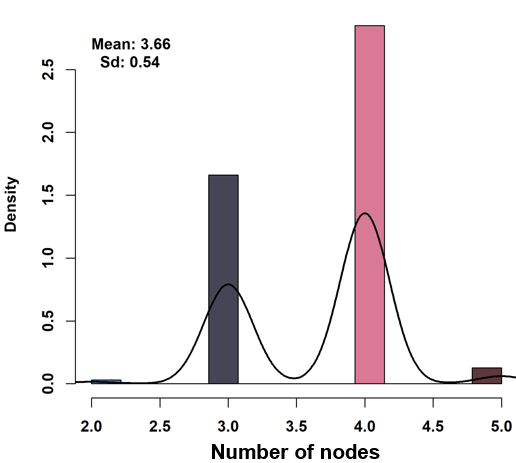 | 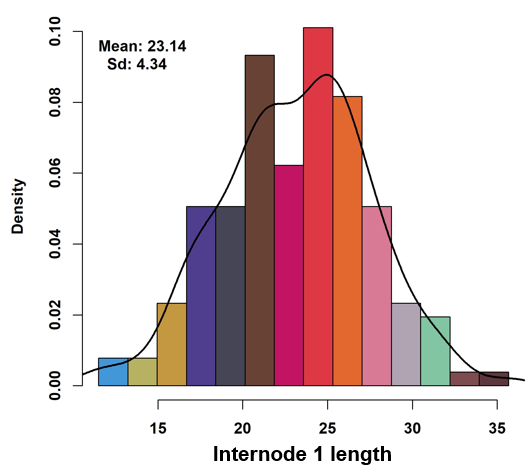 | 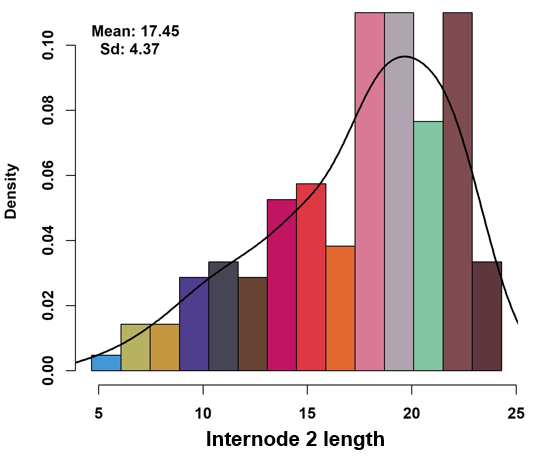 |
| 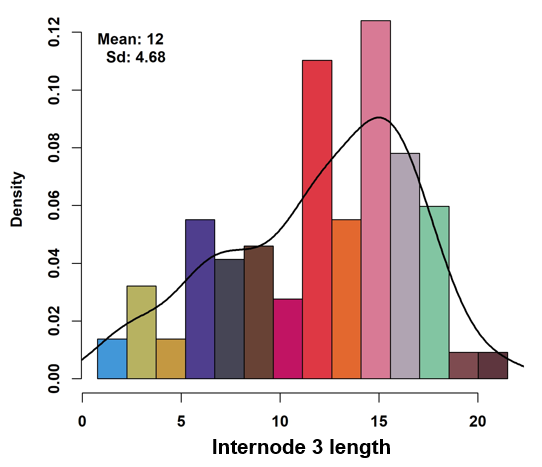 | 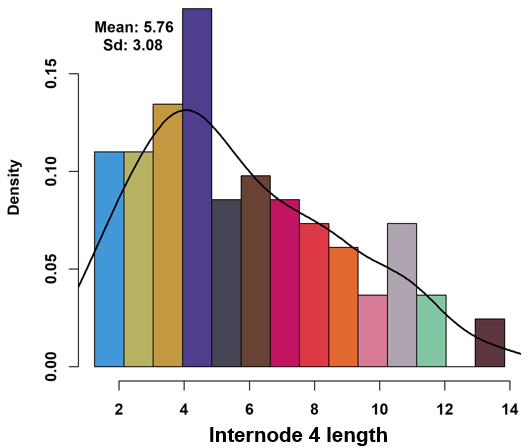 | 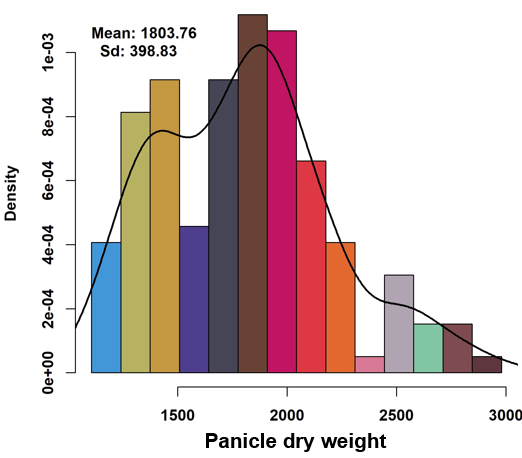 | 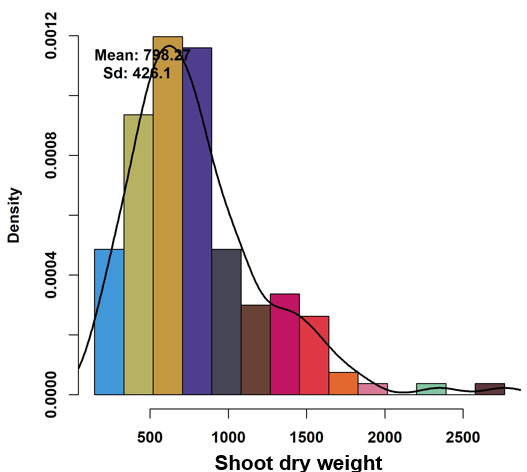 |
| 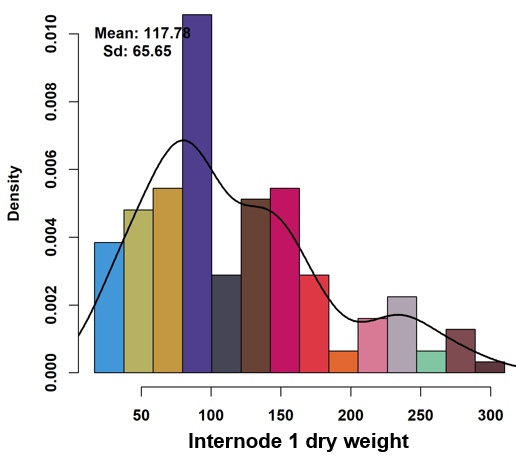 | 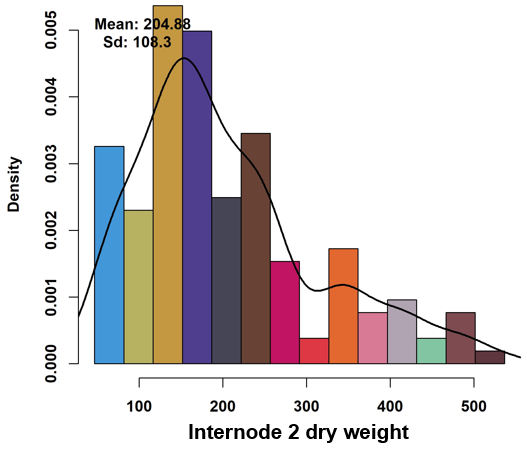 | 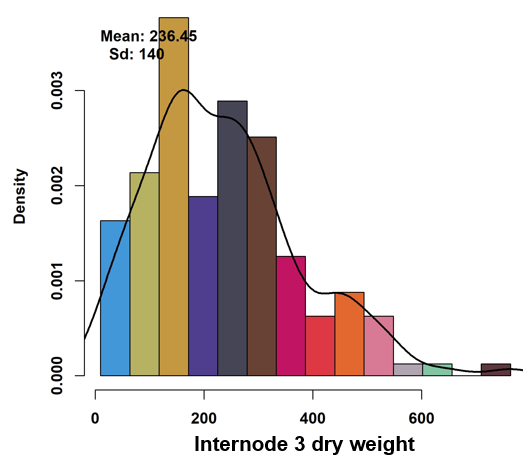 | 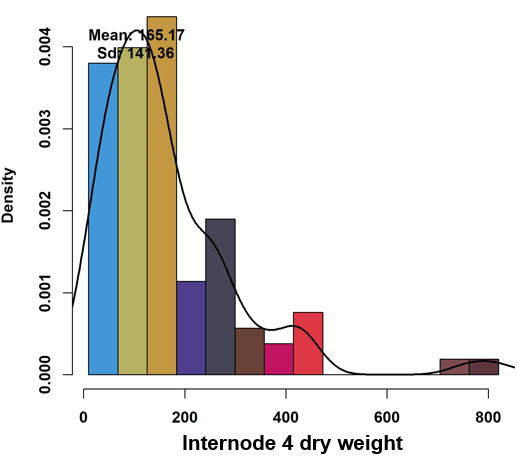 |
| 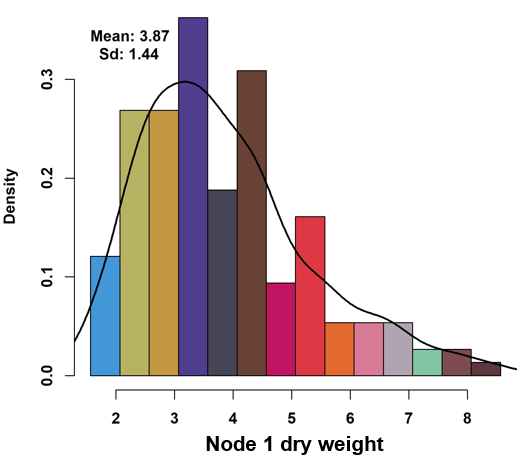 | 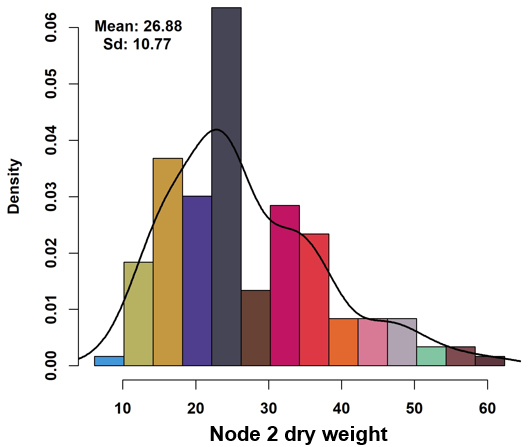 | 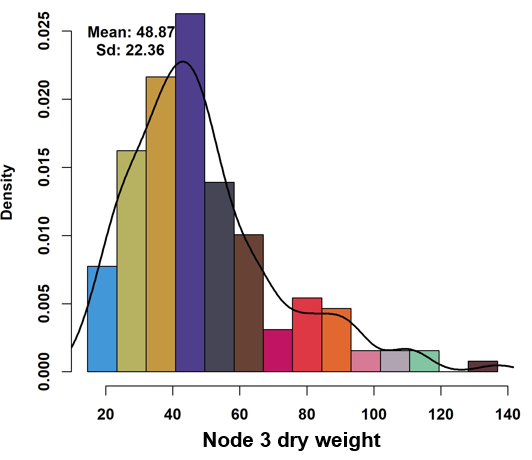 | 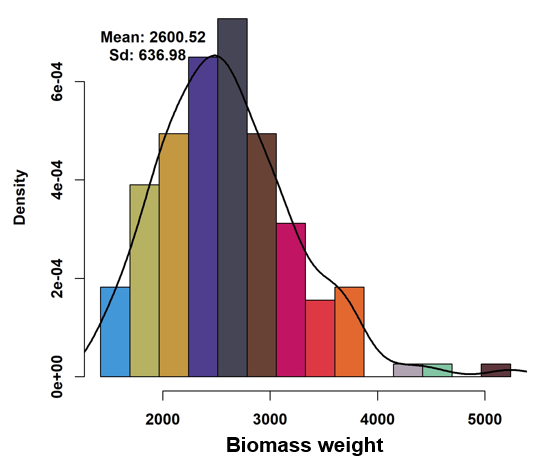 |

**FIGURE S2 |** Phenotypic distribution of individual traits.


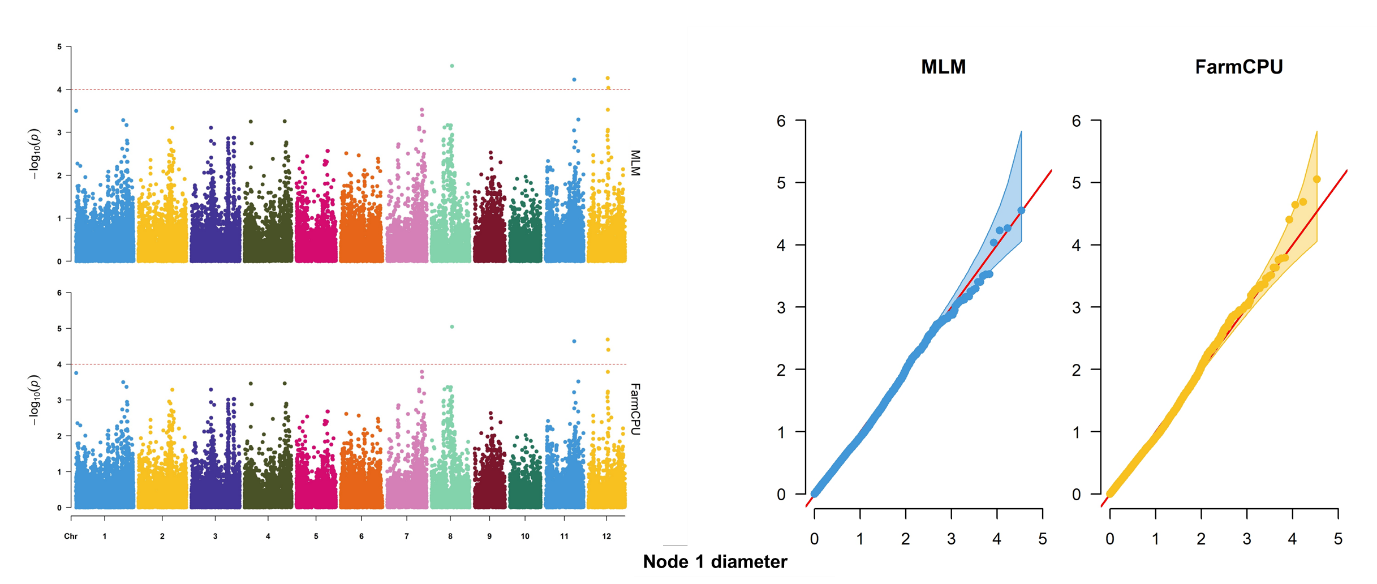


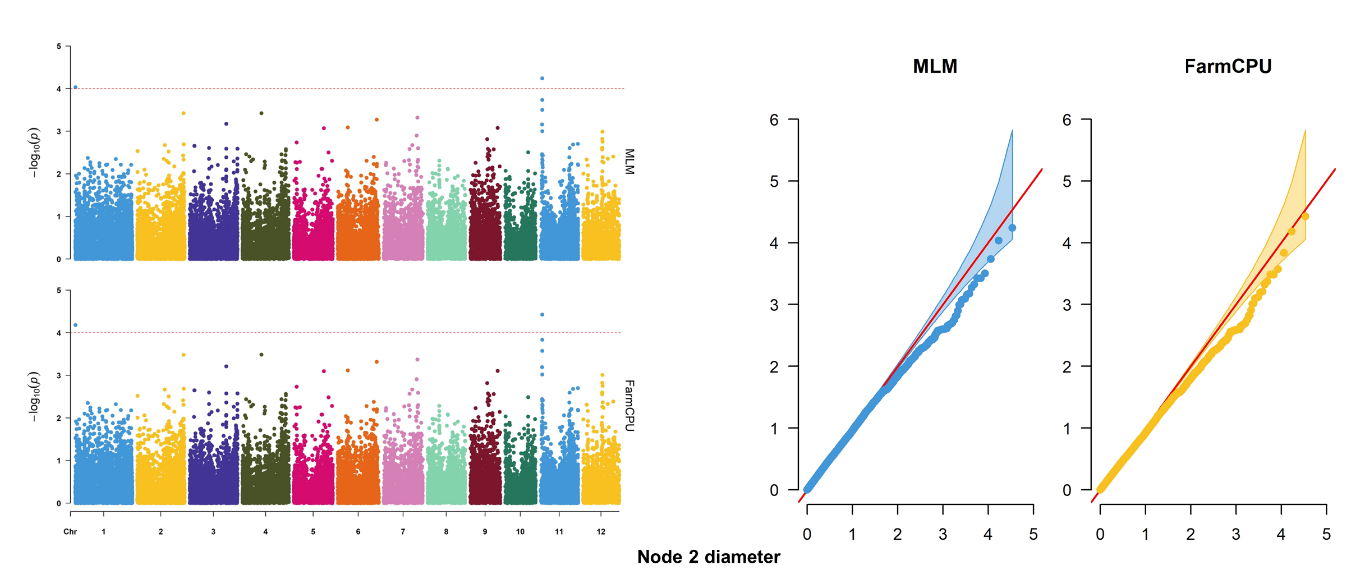


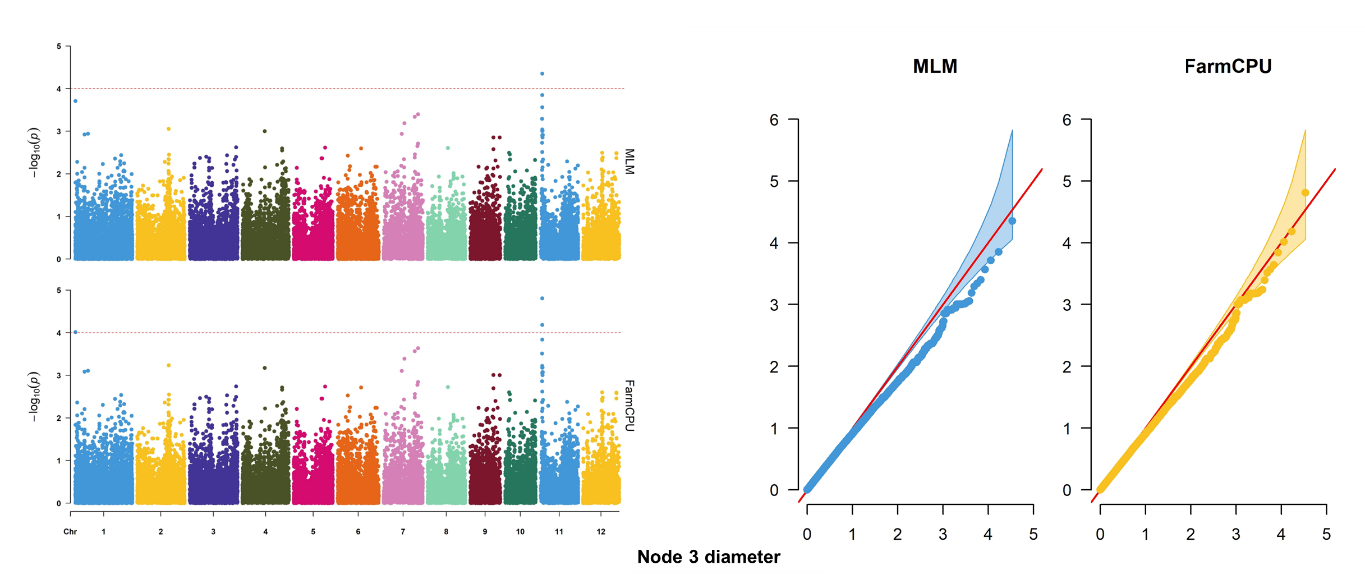


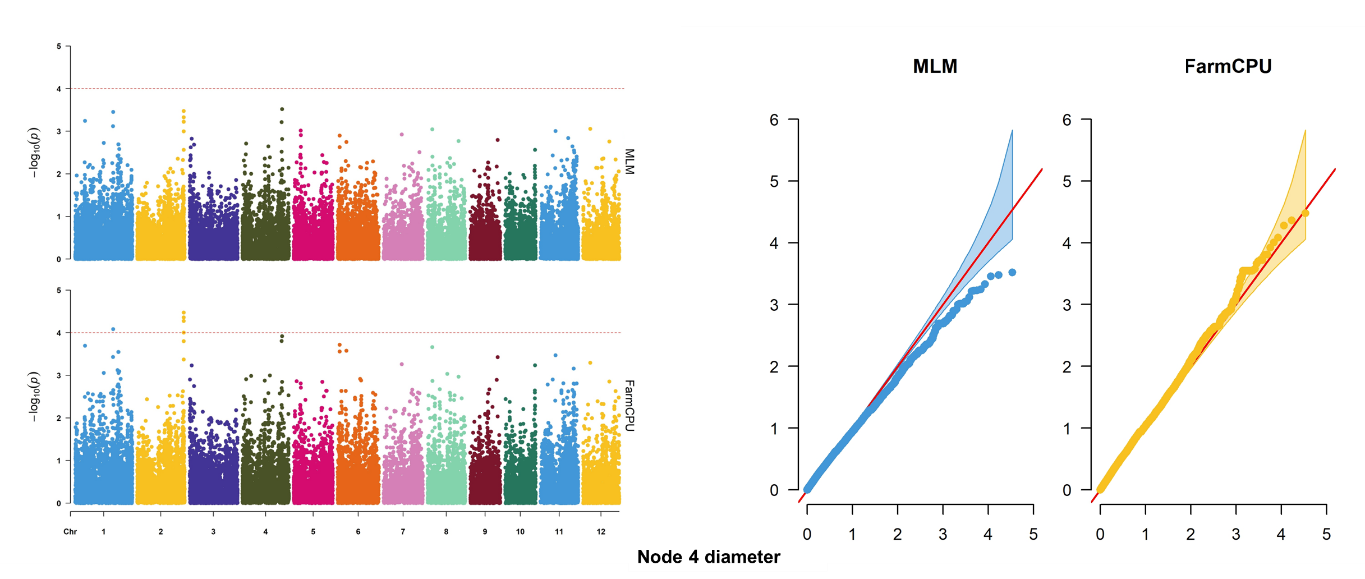


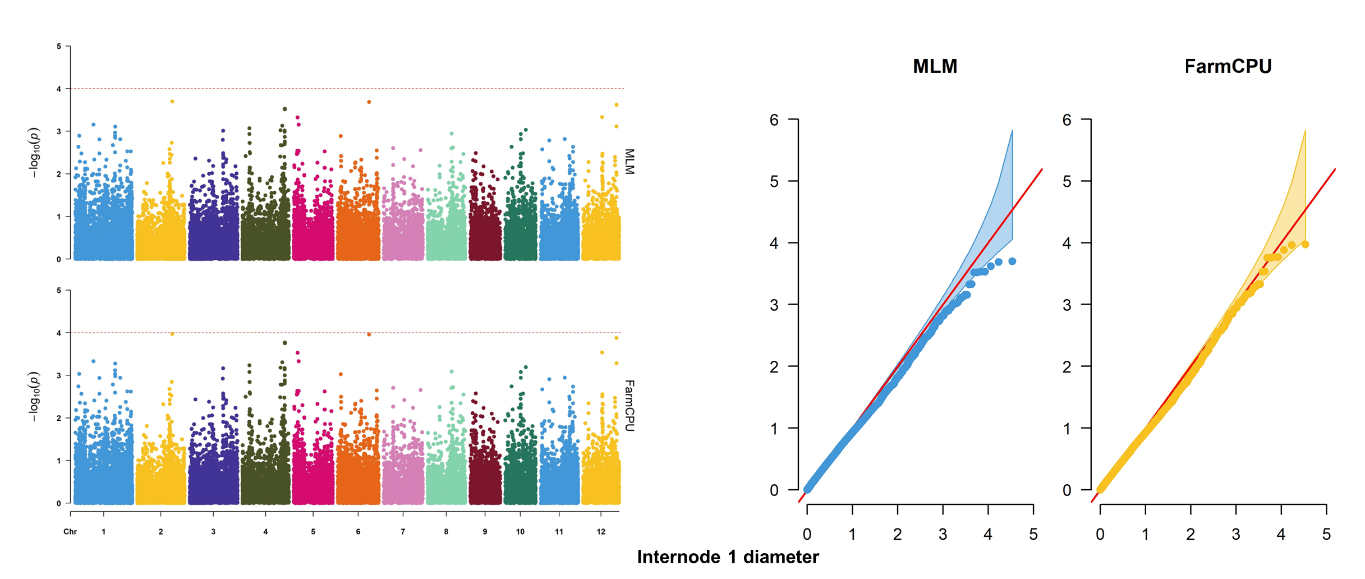


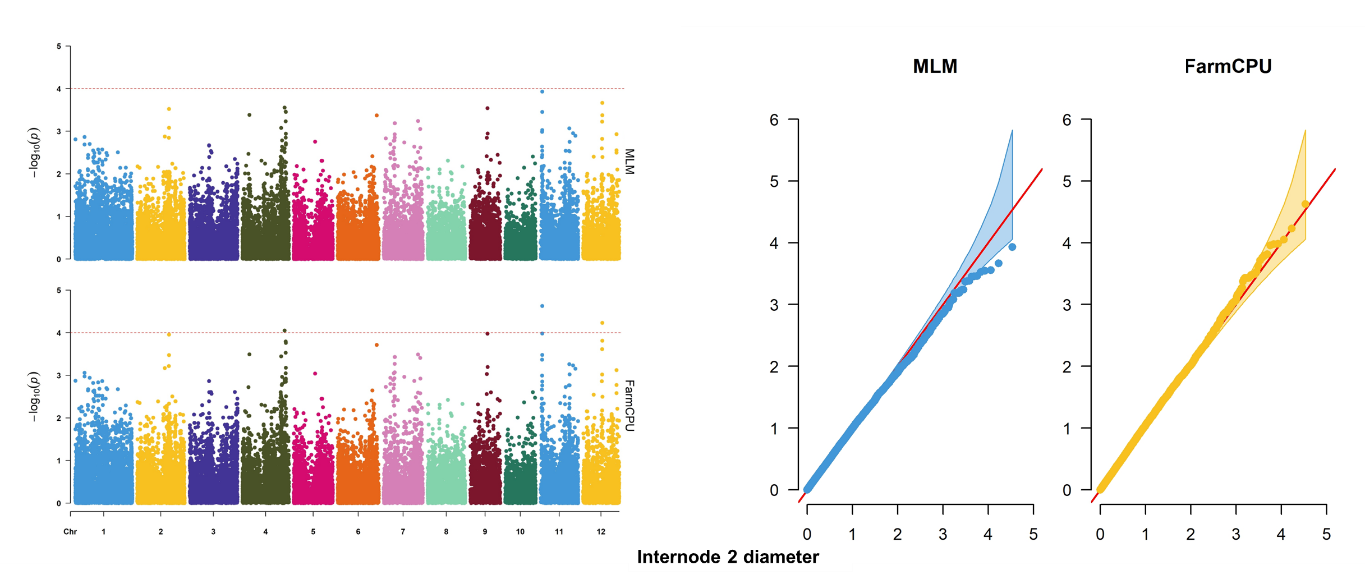


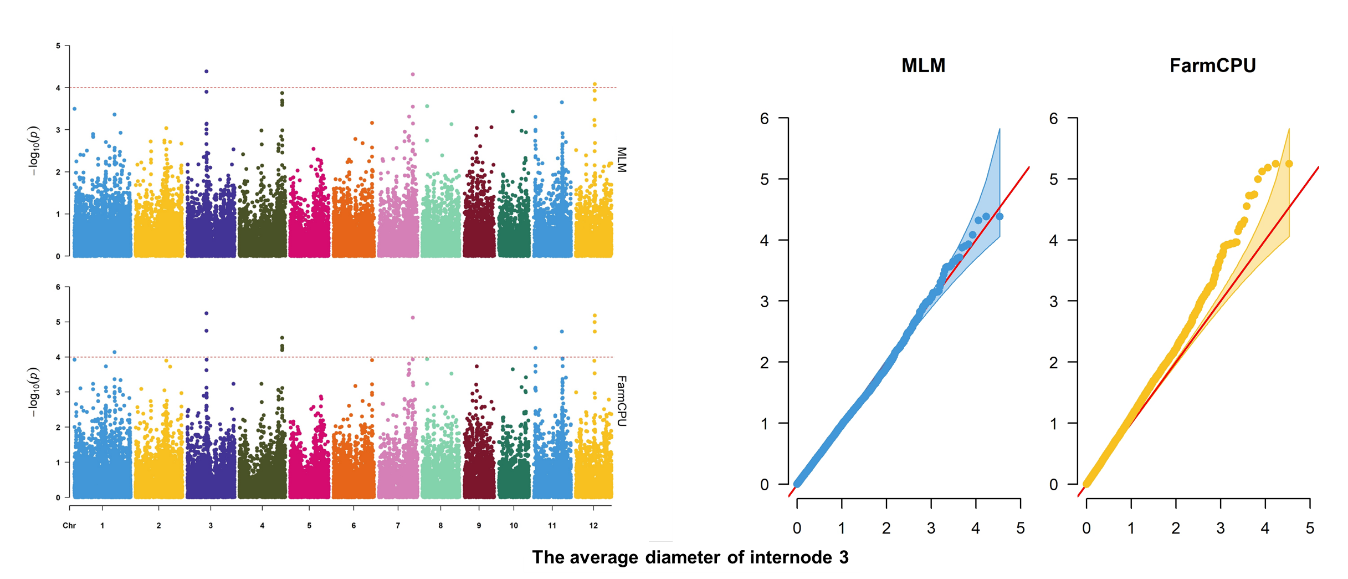


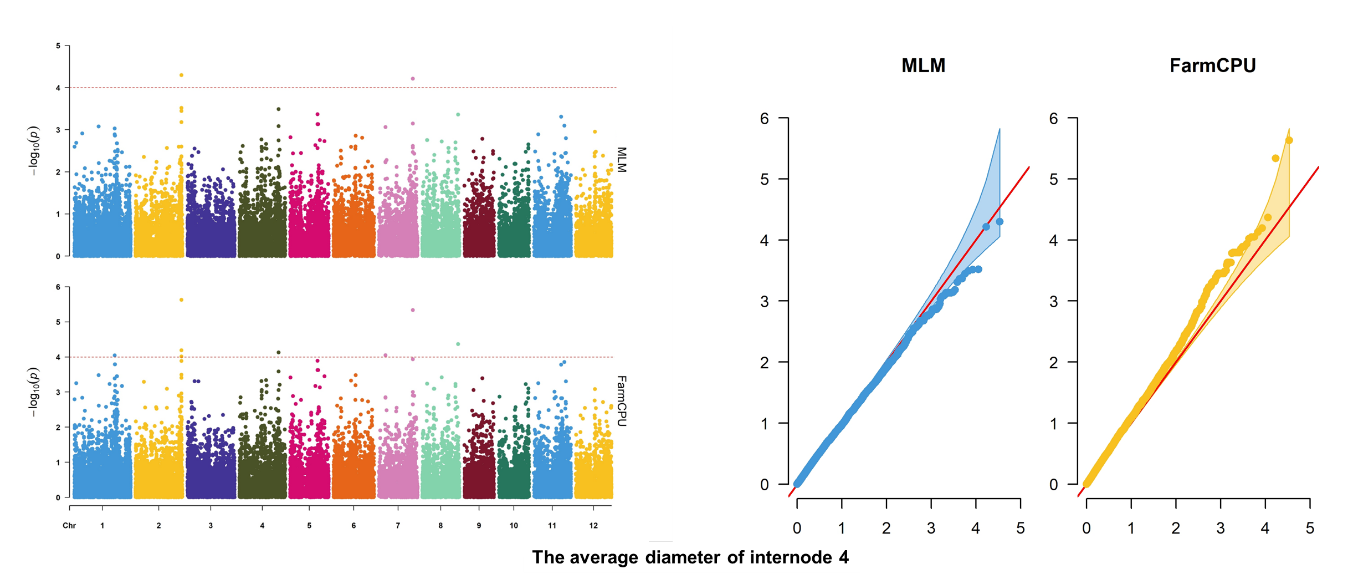


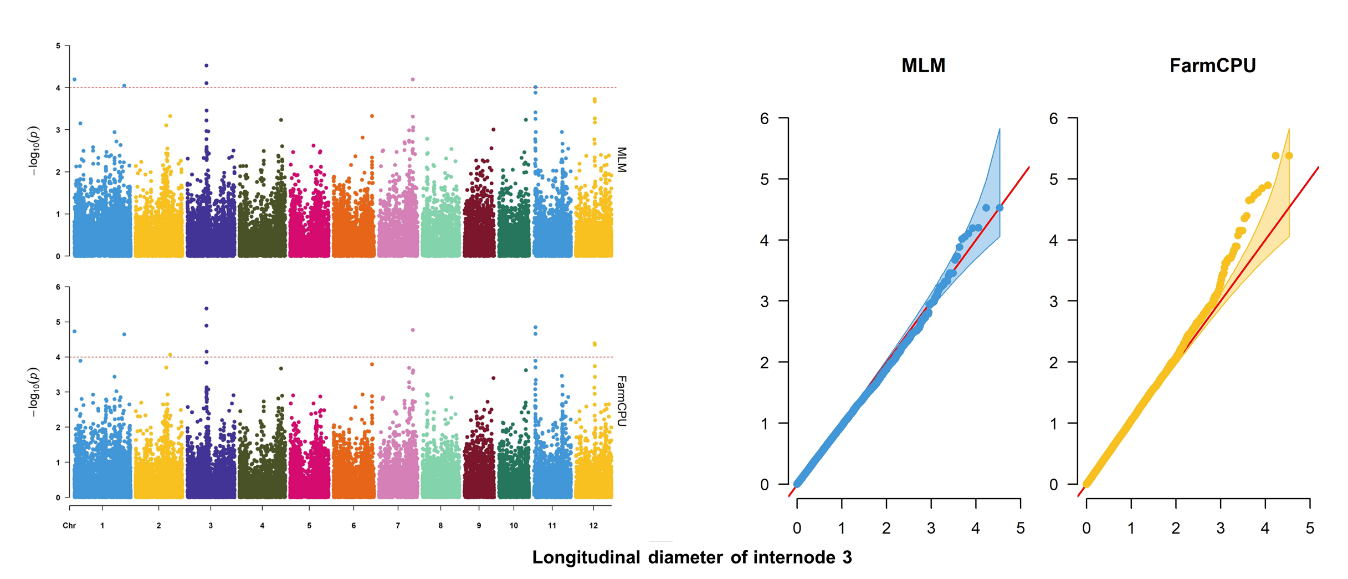


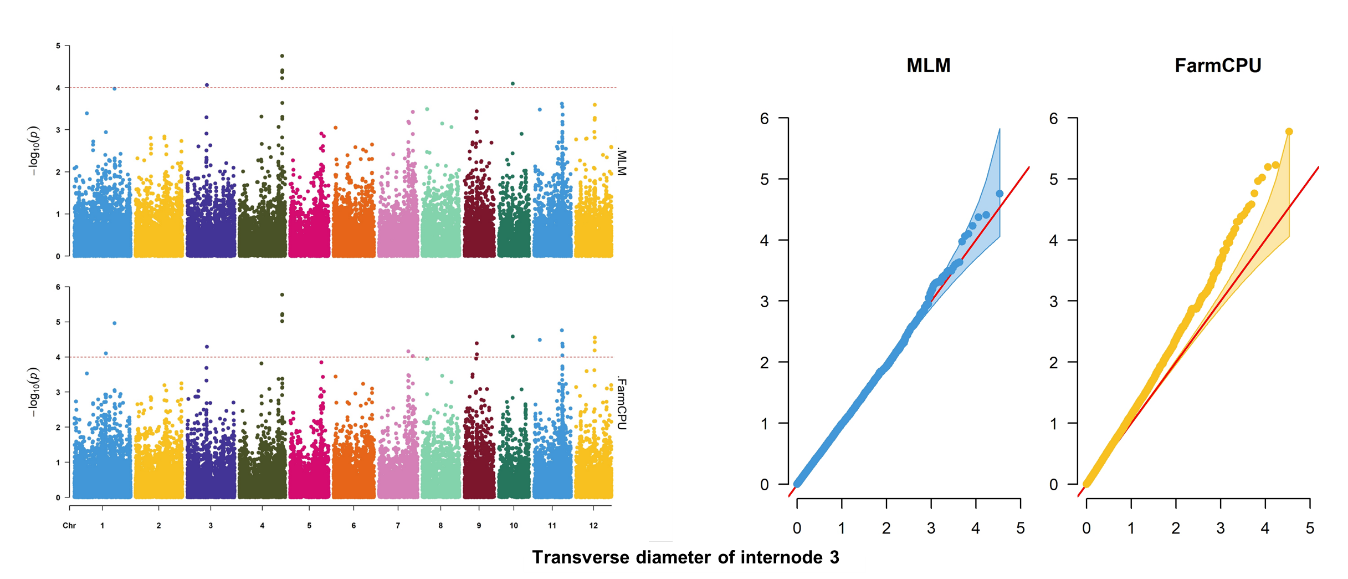


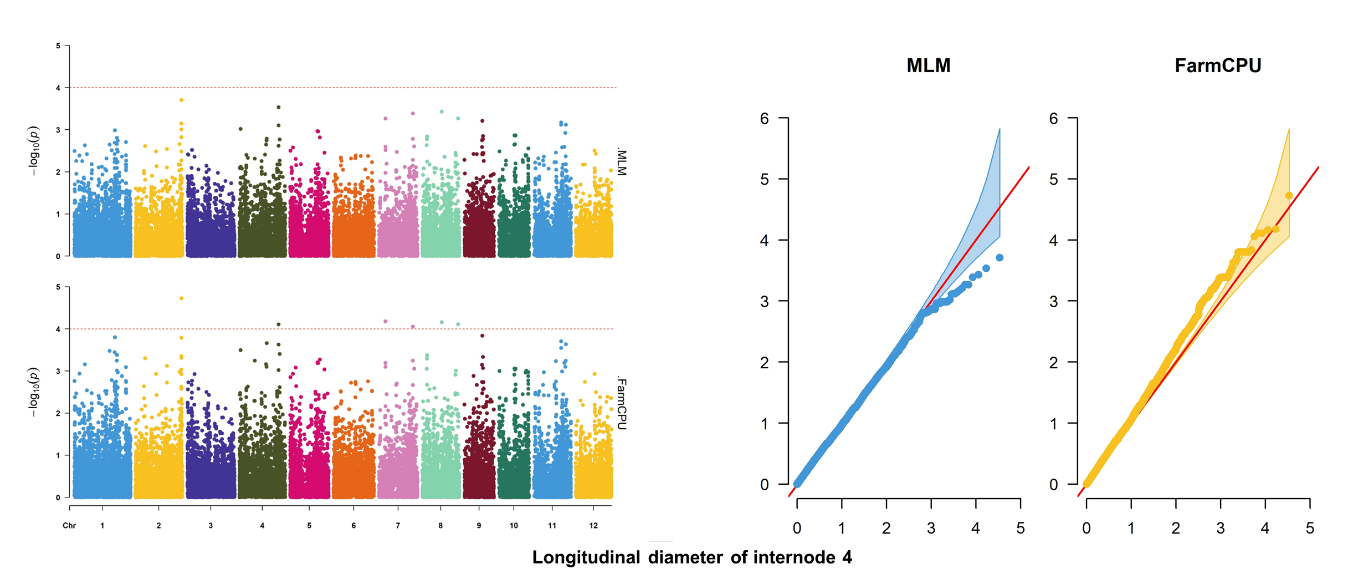


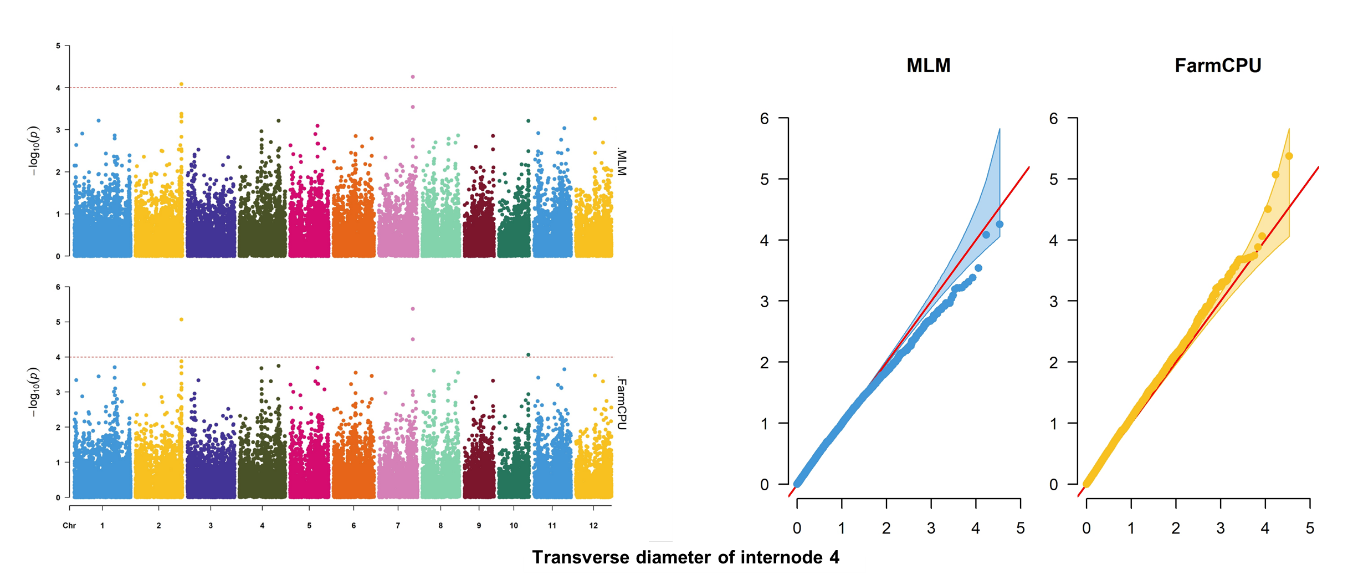


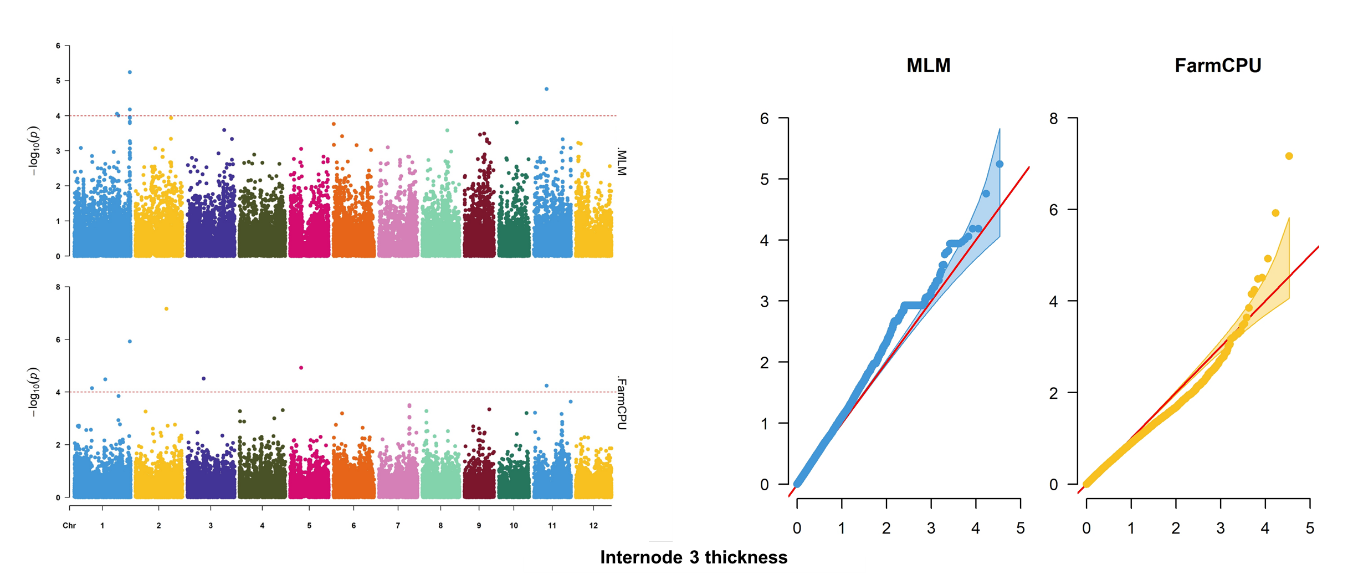


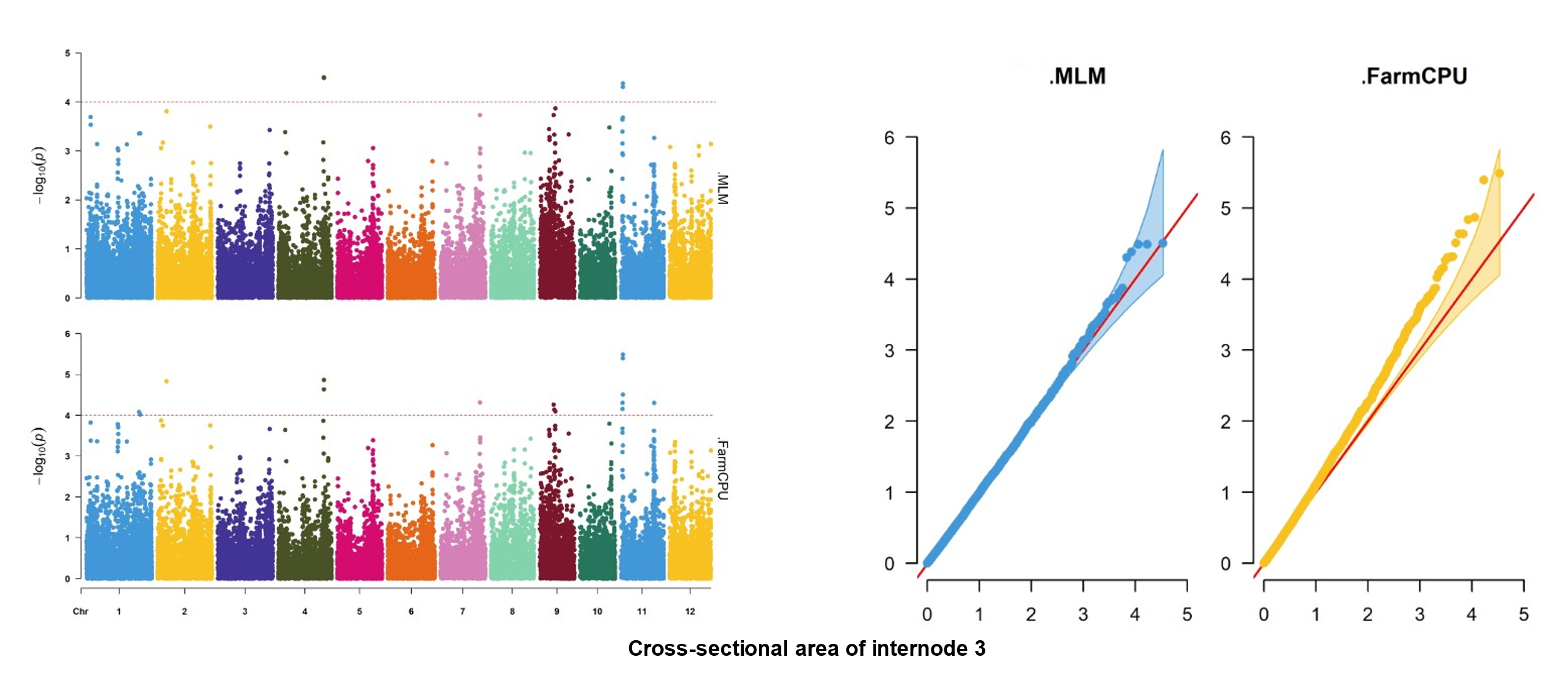


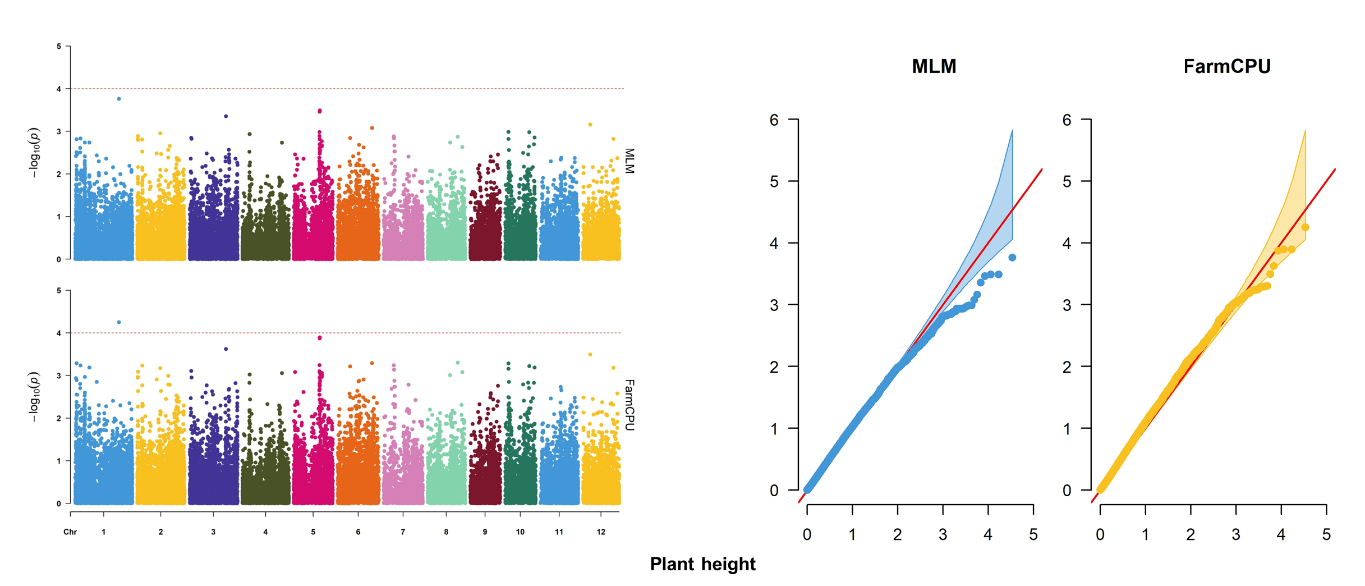


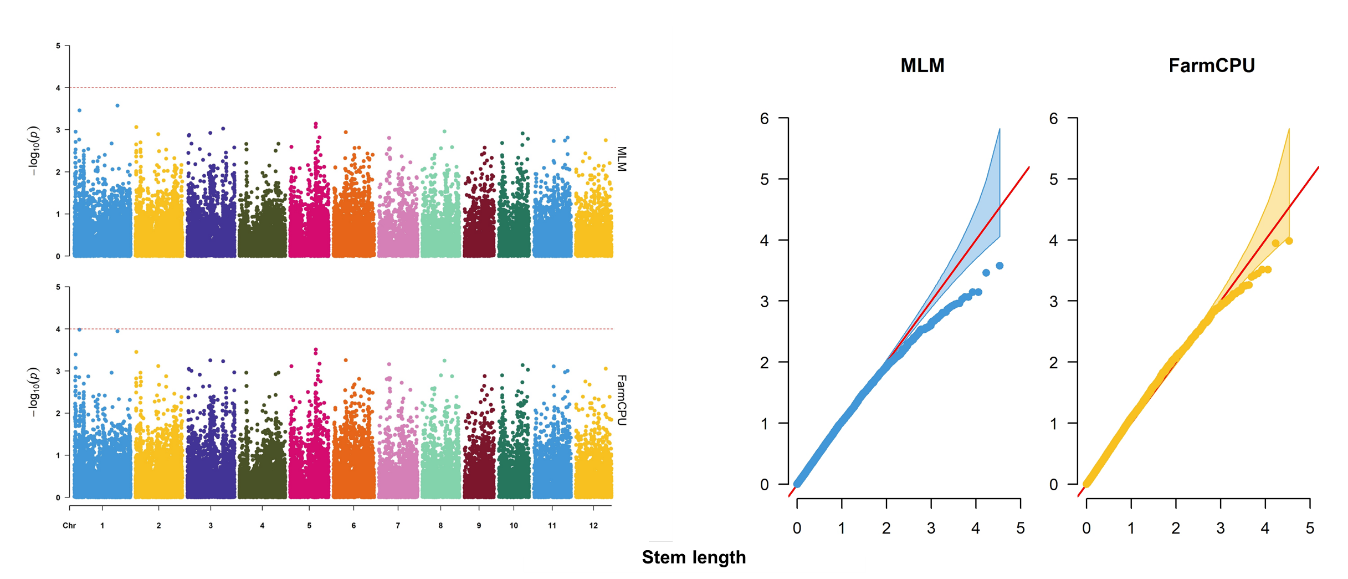


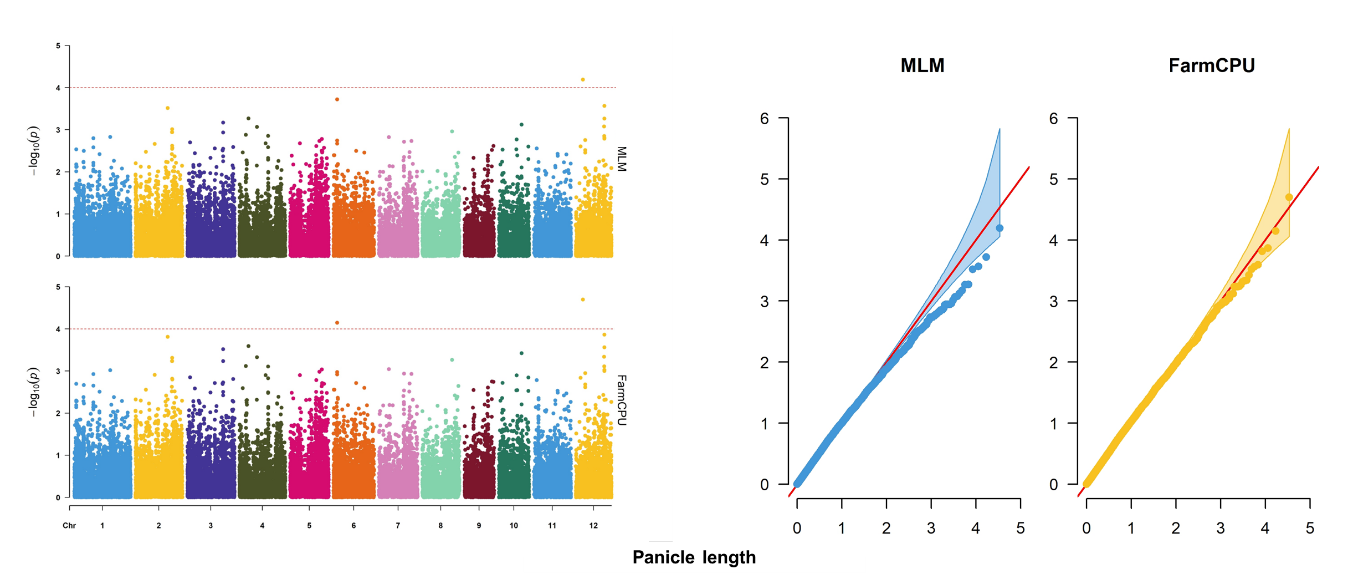


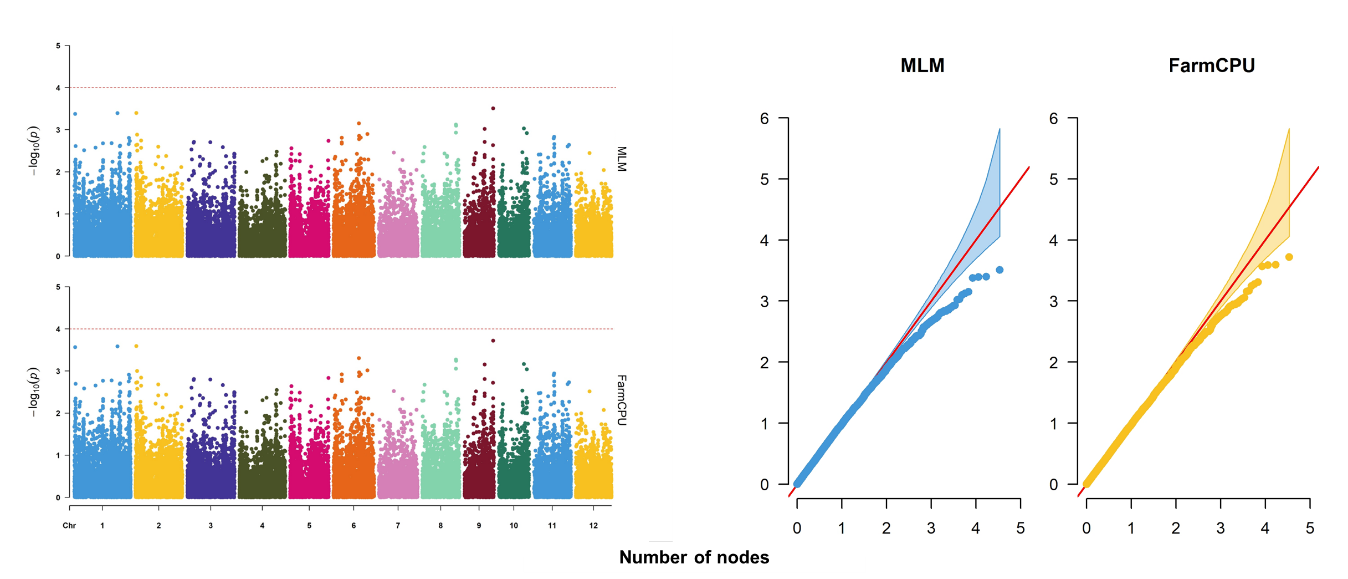


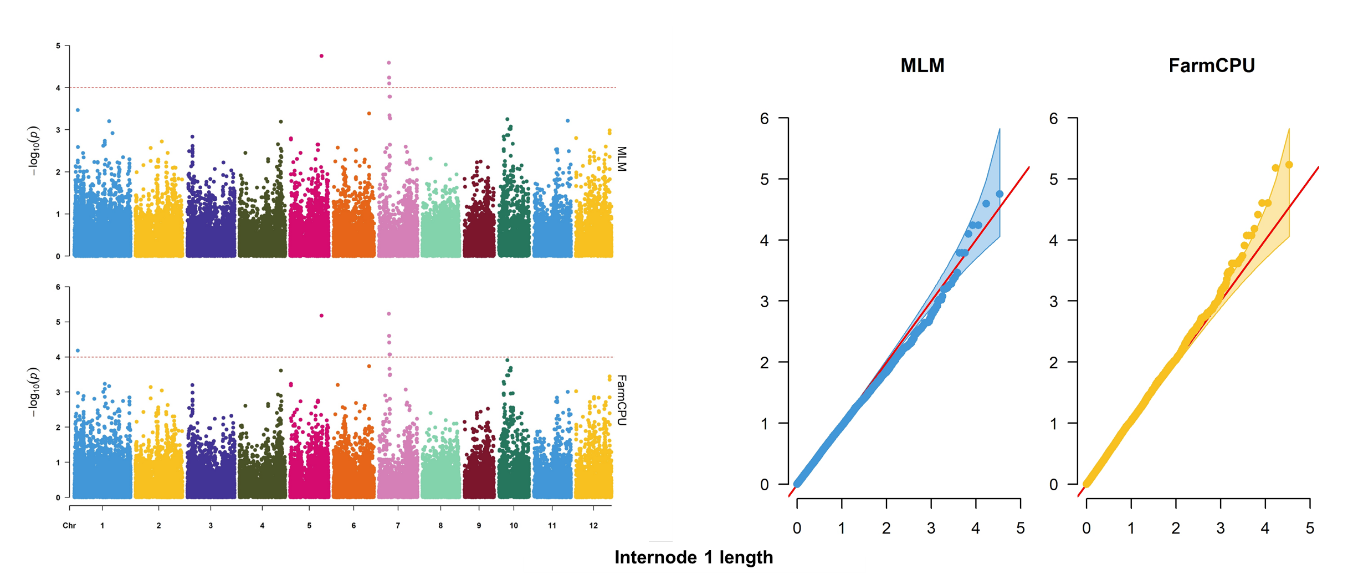


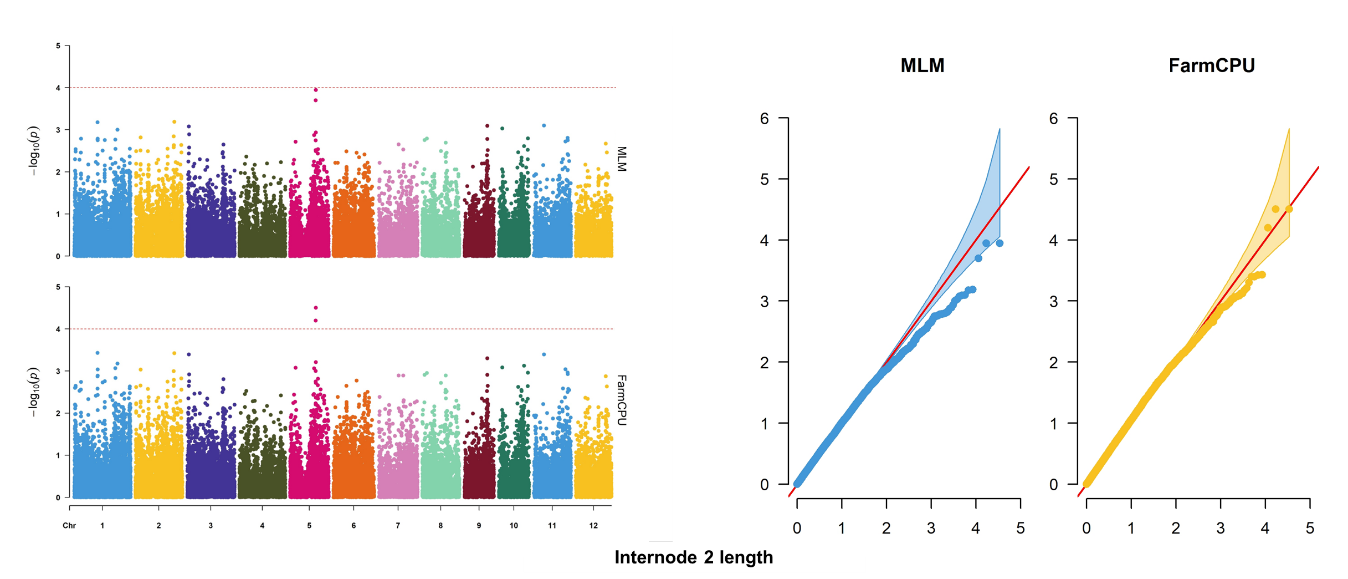


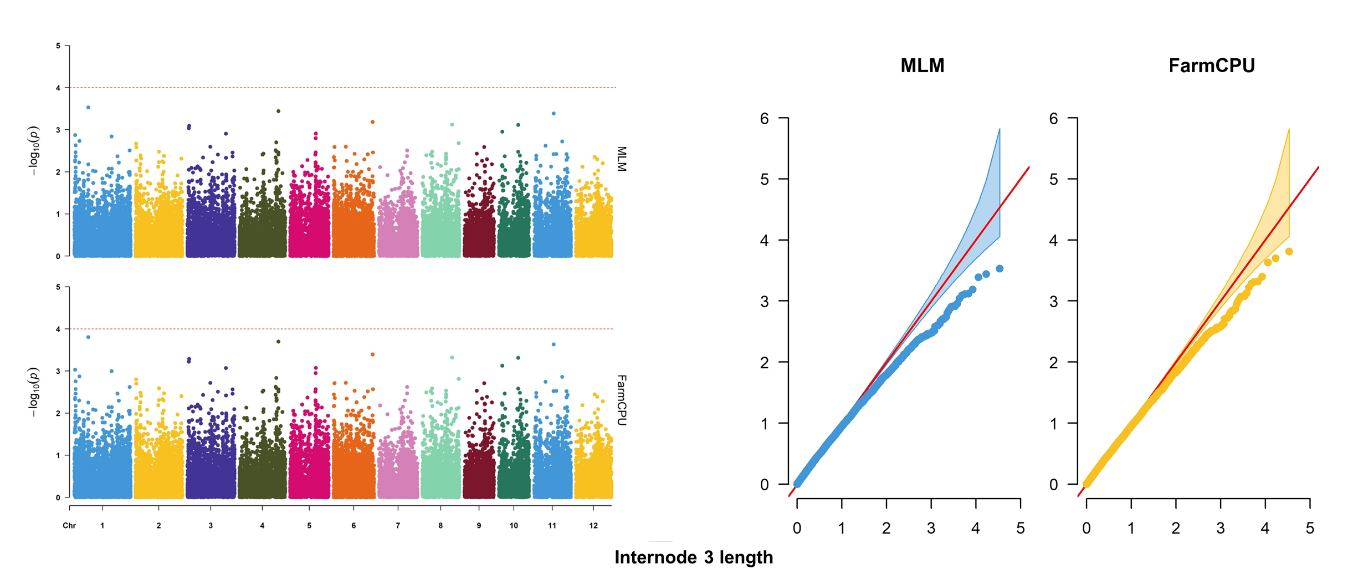


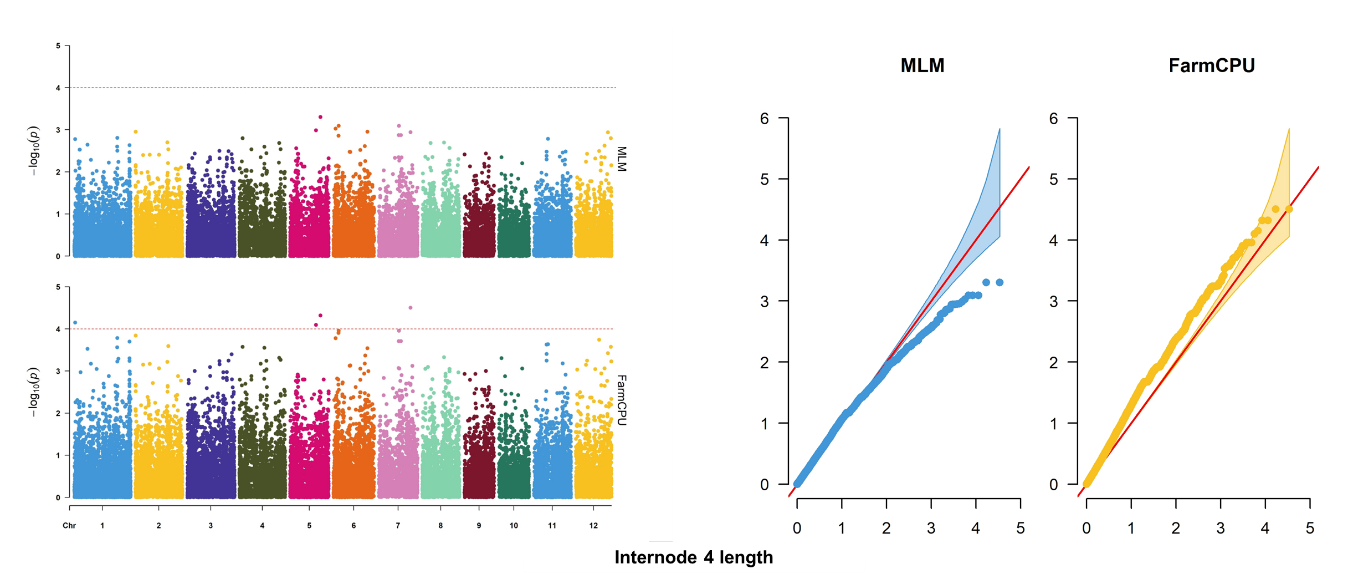


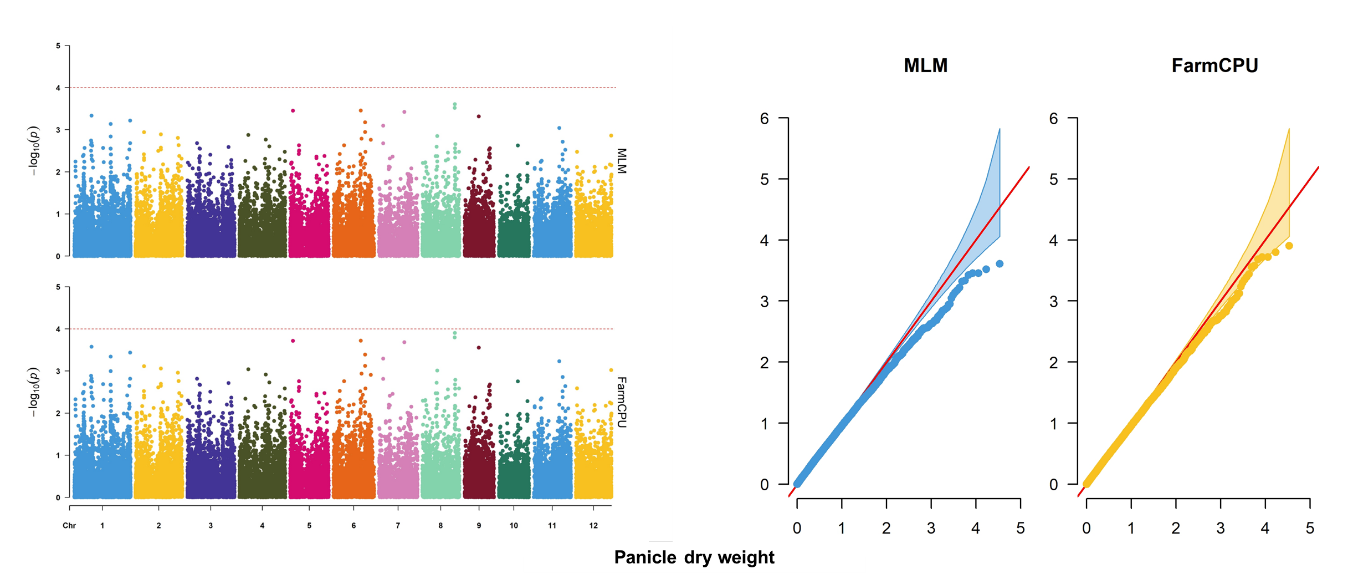


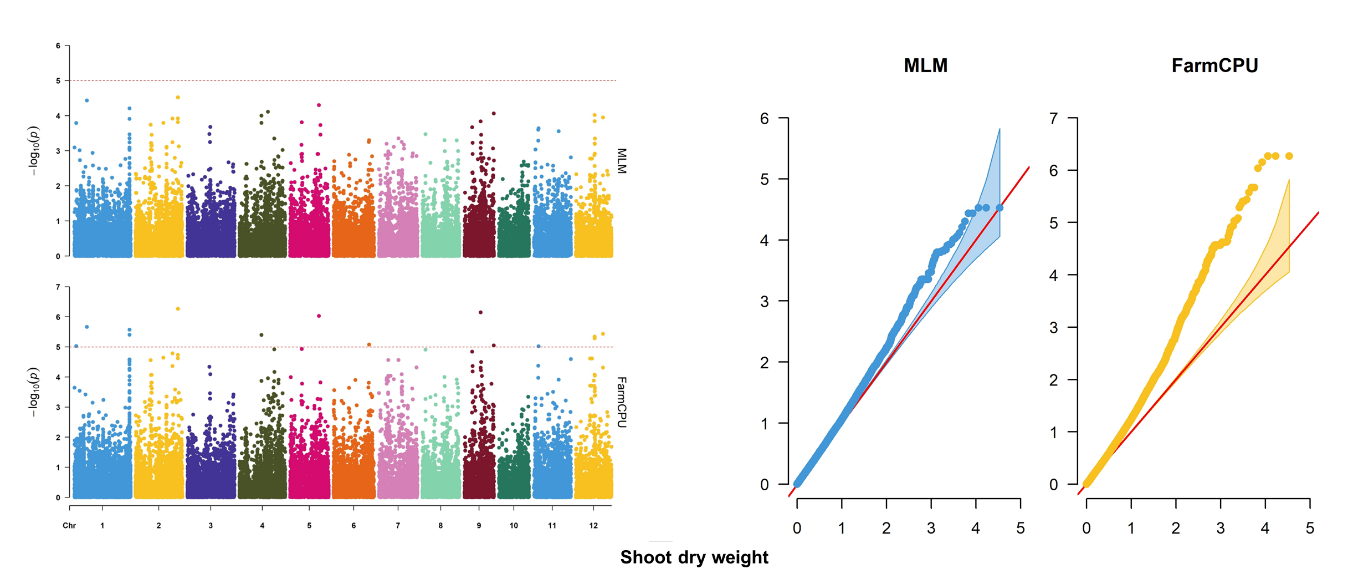


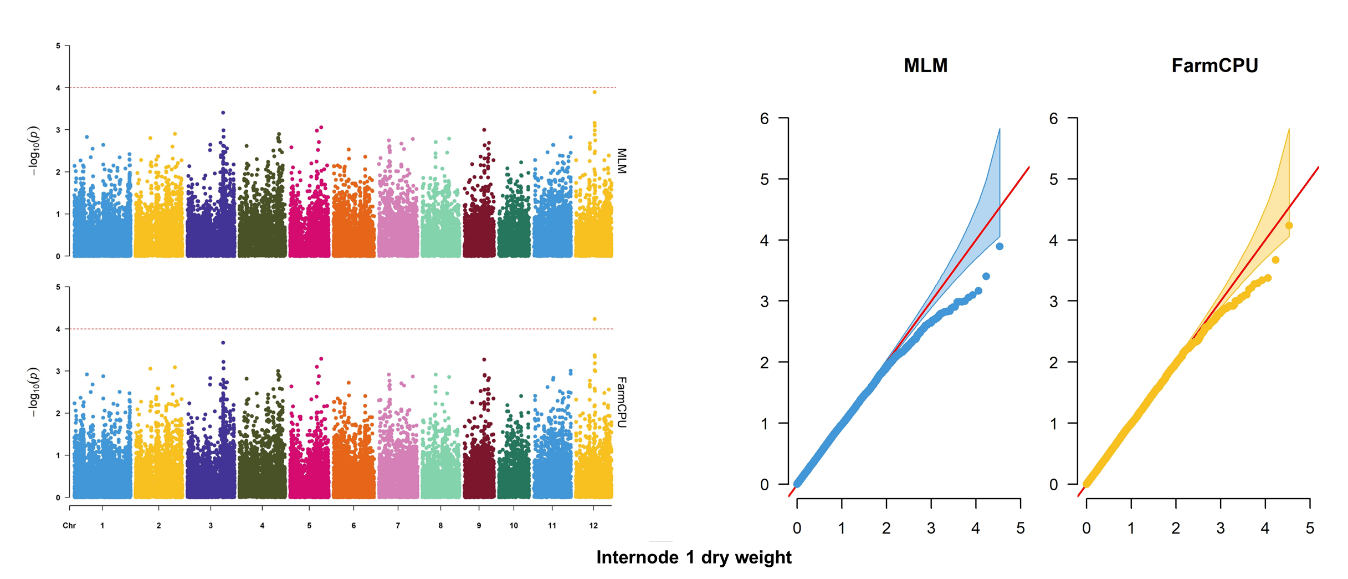


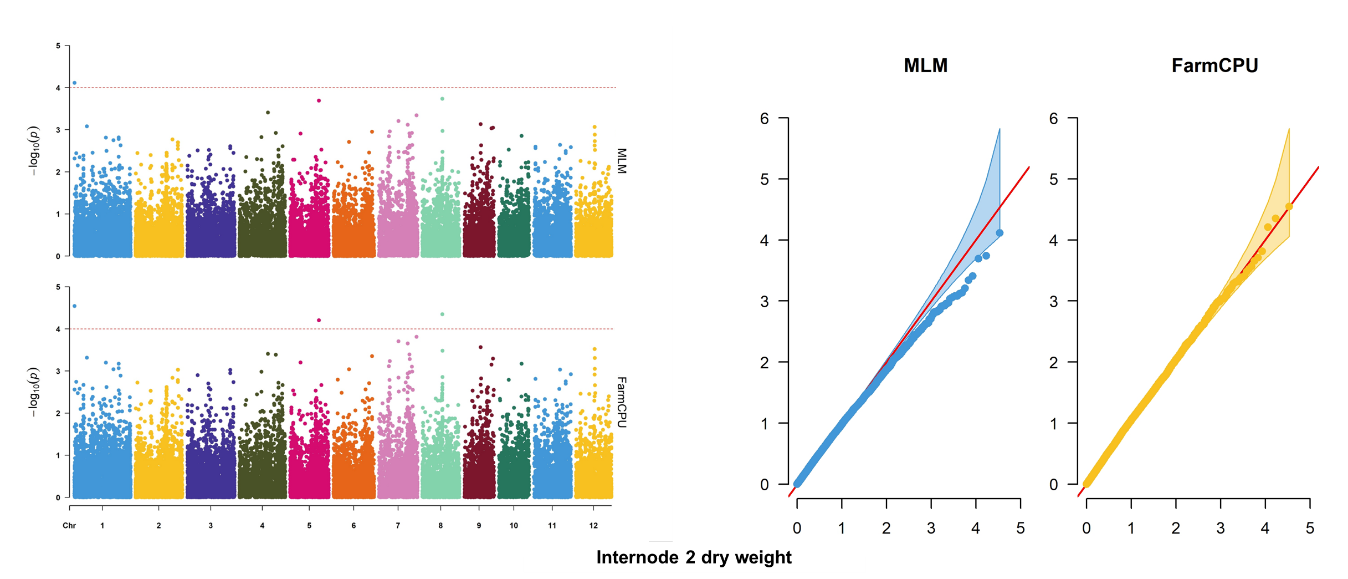


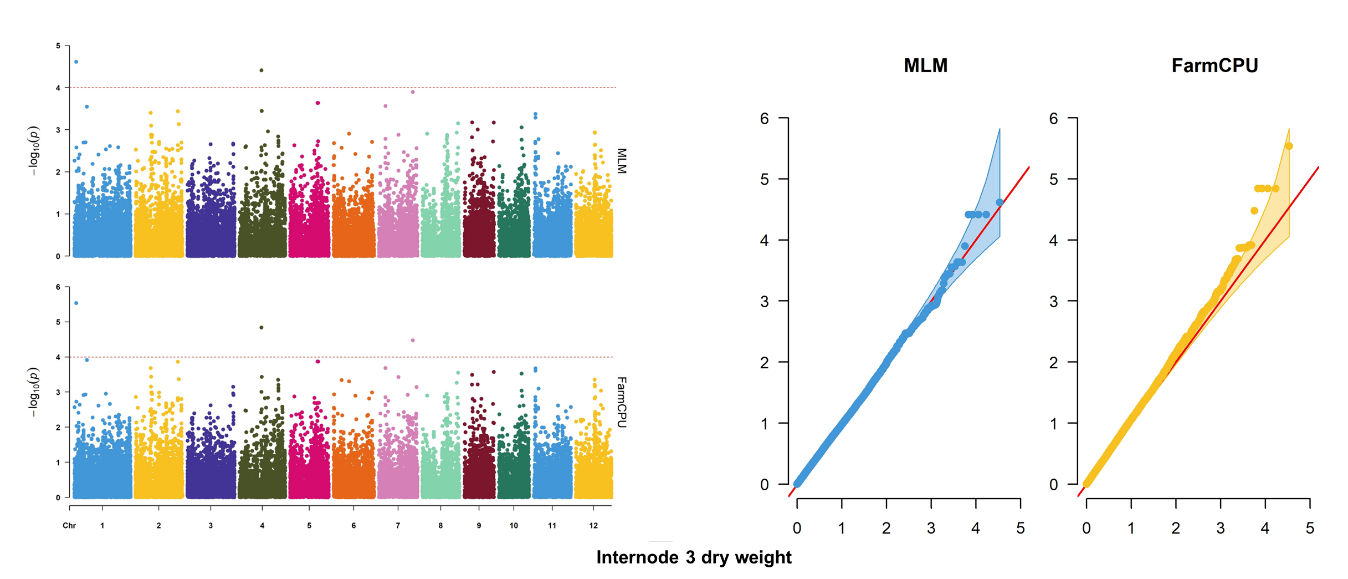


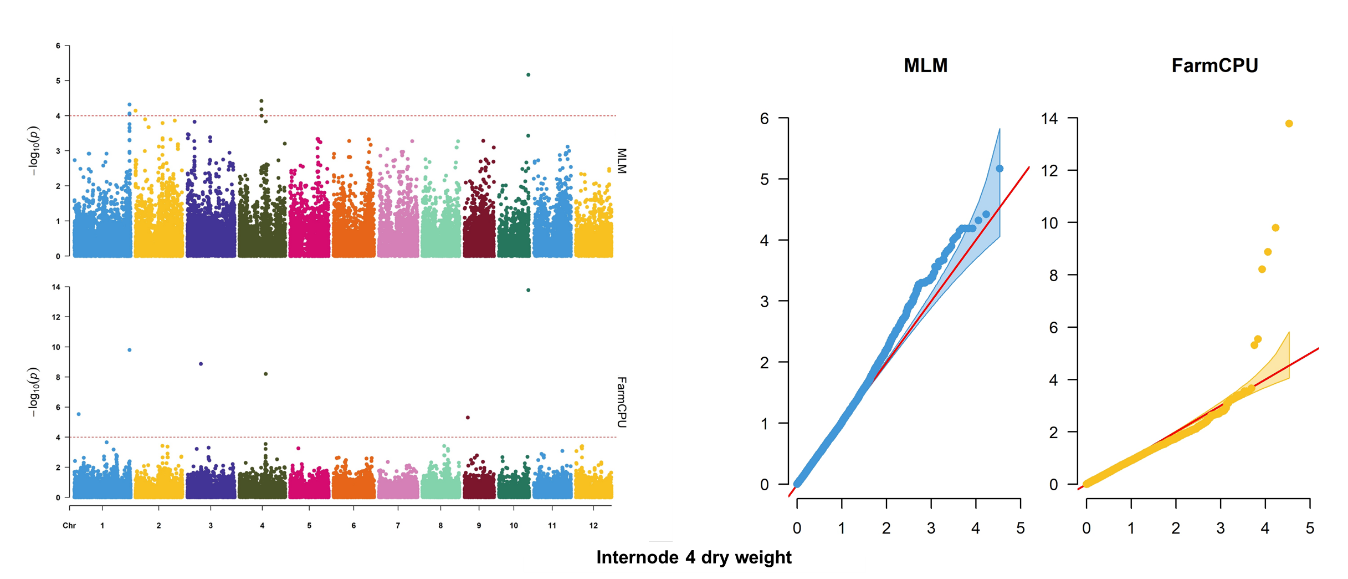


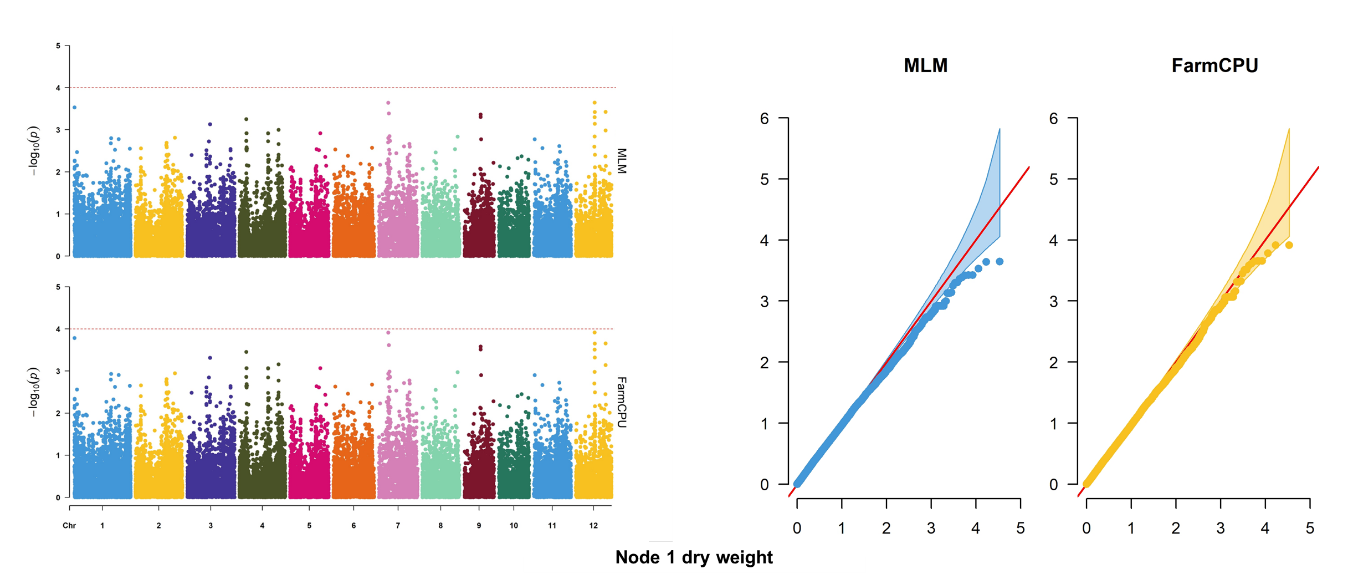


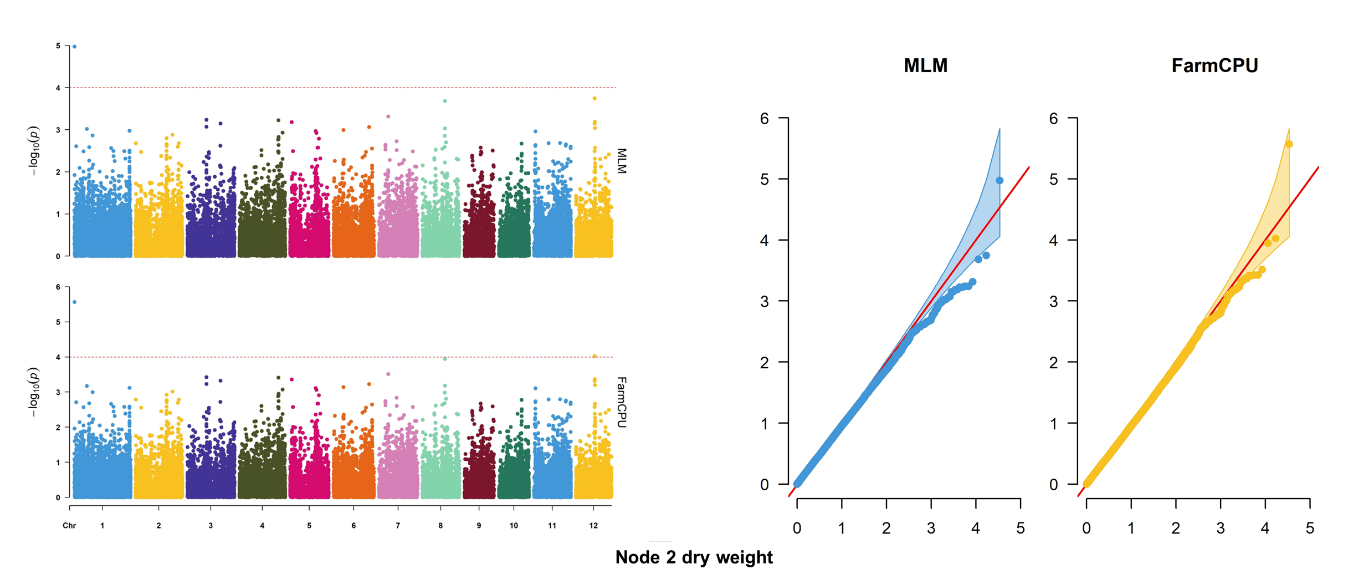


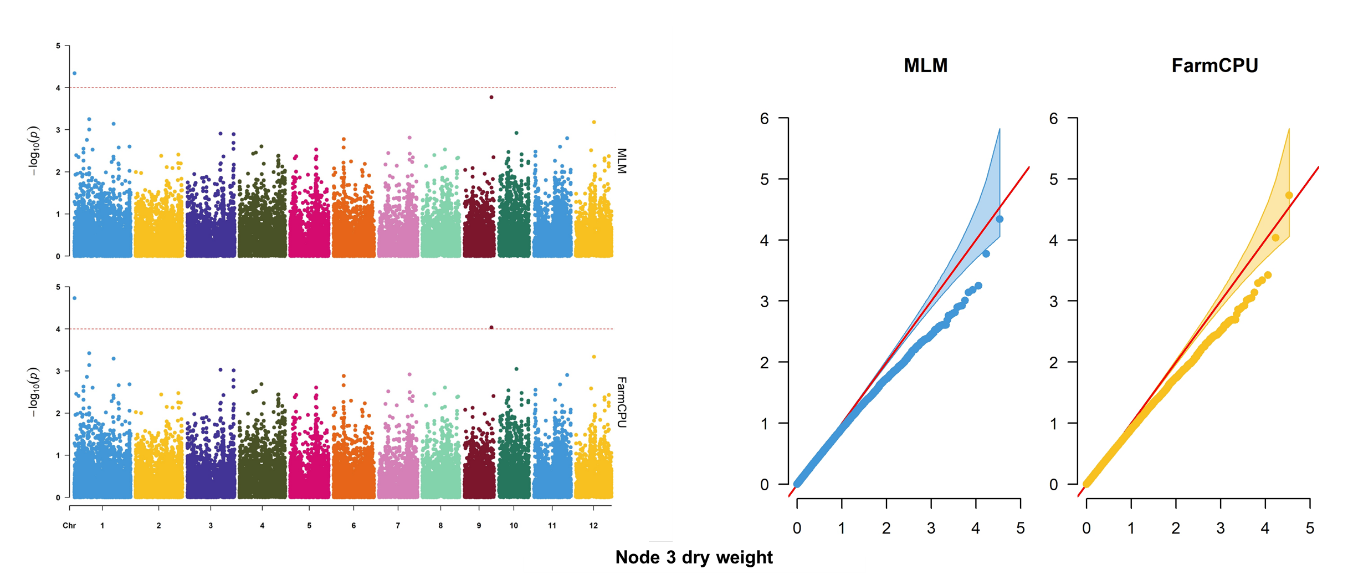


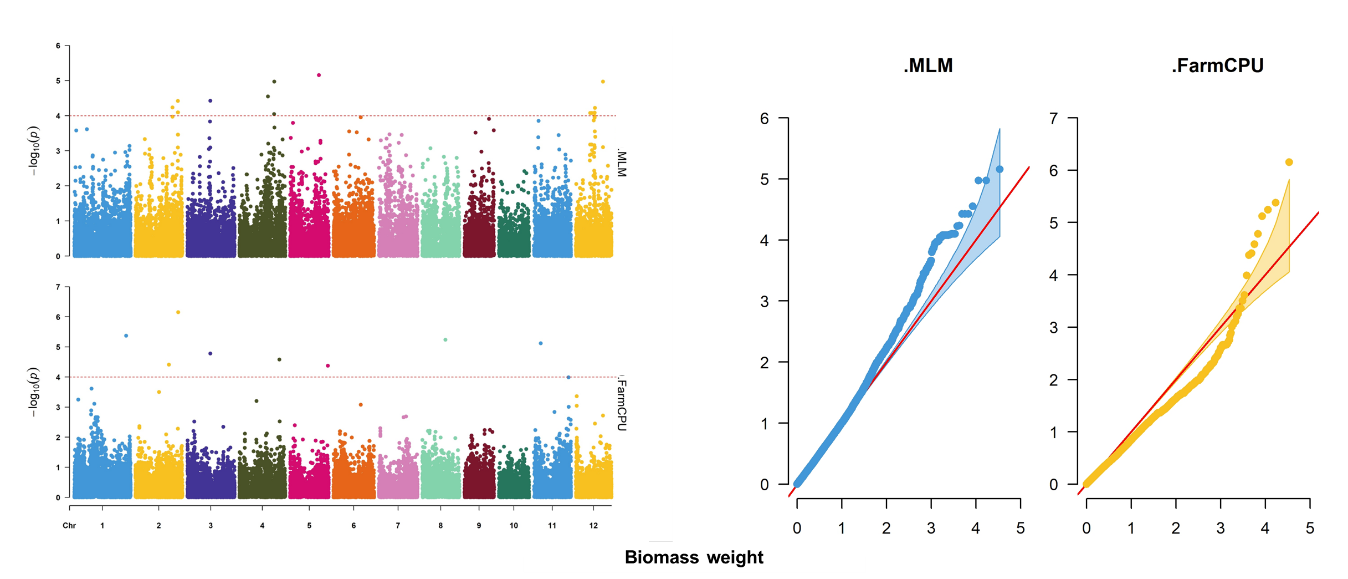


FIGURE S3 | Genome-wide association mapping of traits measured at the heading stage in field-grown rice accessions. Manhattan (left) and Q-Q (right) plots based on MLM and FarmCPU models for all traits. The red horizontal dashed lines indicate the genome-wide significance thresholds.


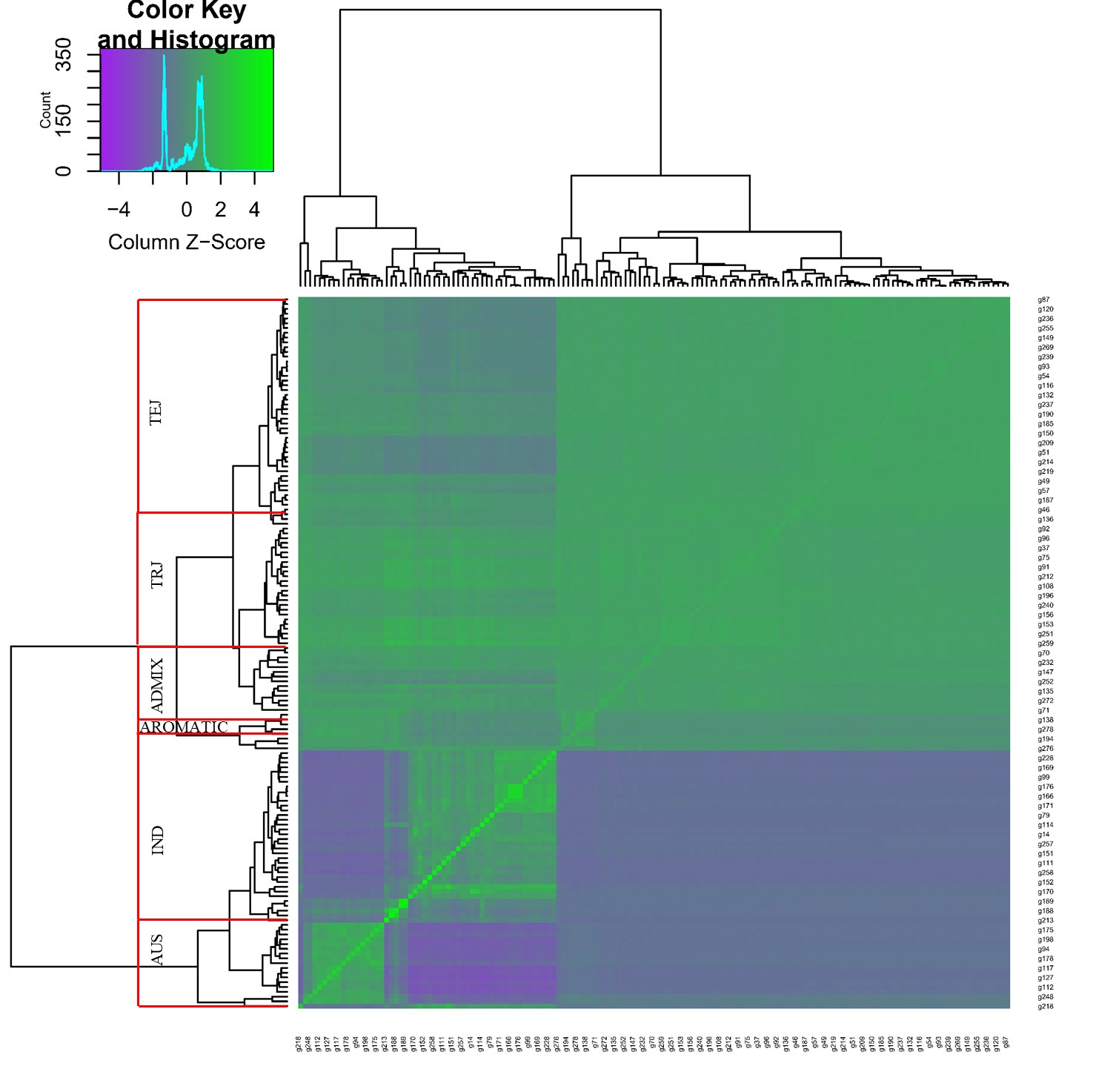


**FIGURE S4|** Phylogenetic tree represented as a kinship plot, efficiently separating the 149 accessions into five major geographical subpopulation clusters: TEJ (Temperate japonica), IND (indica), AUS (aus), TRJ (Tropical japonica), and ADMIX. Green indicates the highest correlation between pairs of individuals, while blue indicates the lowest. A hierarchical clustering tree based on pairwise kinship values for all accessions is shown along the top and left axes.

| **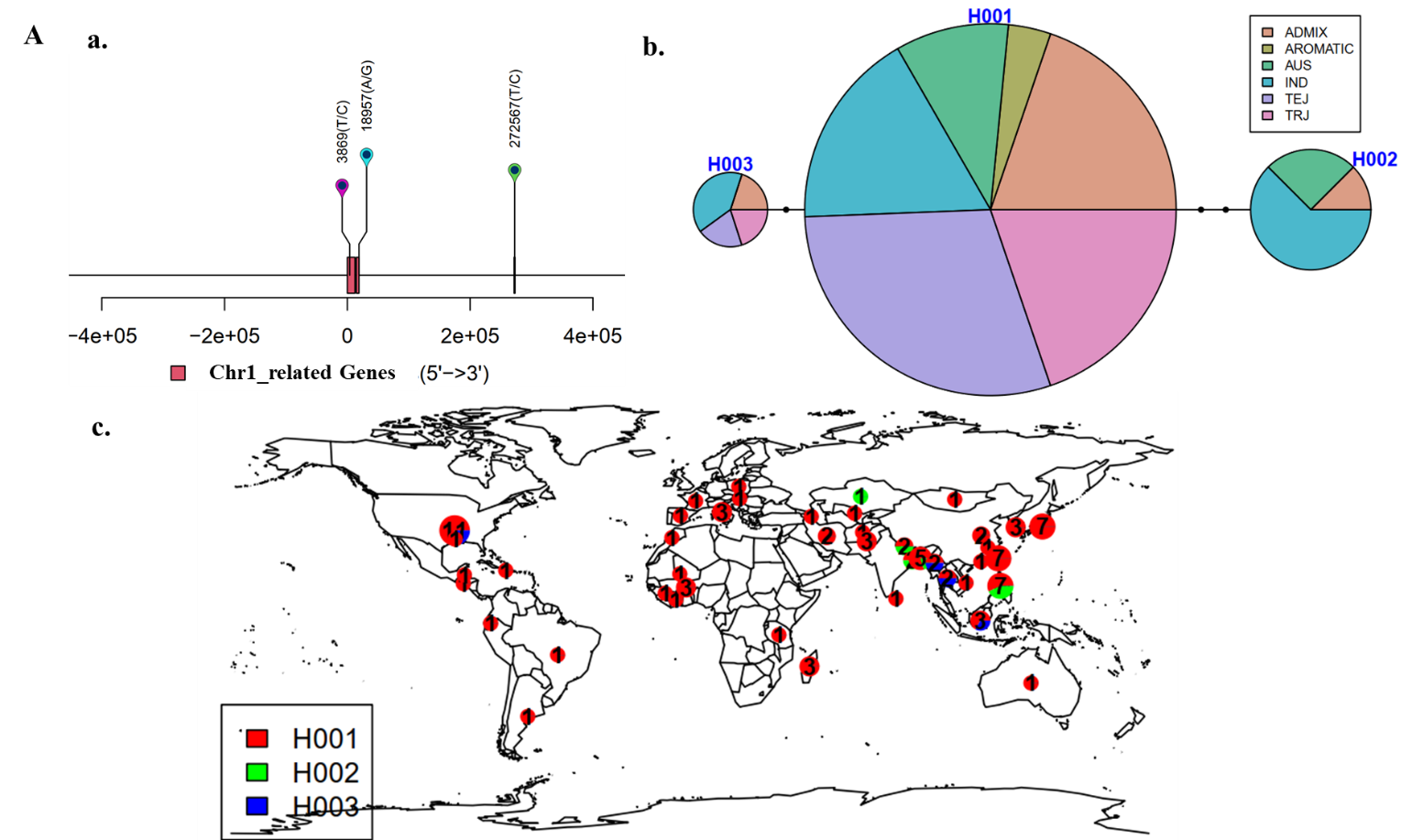** |
| --- |
| **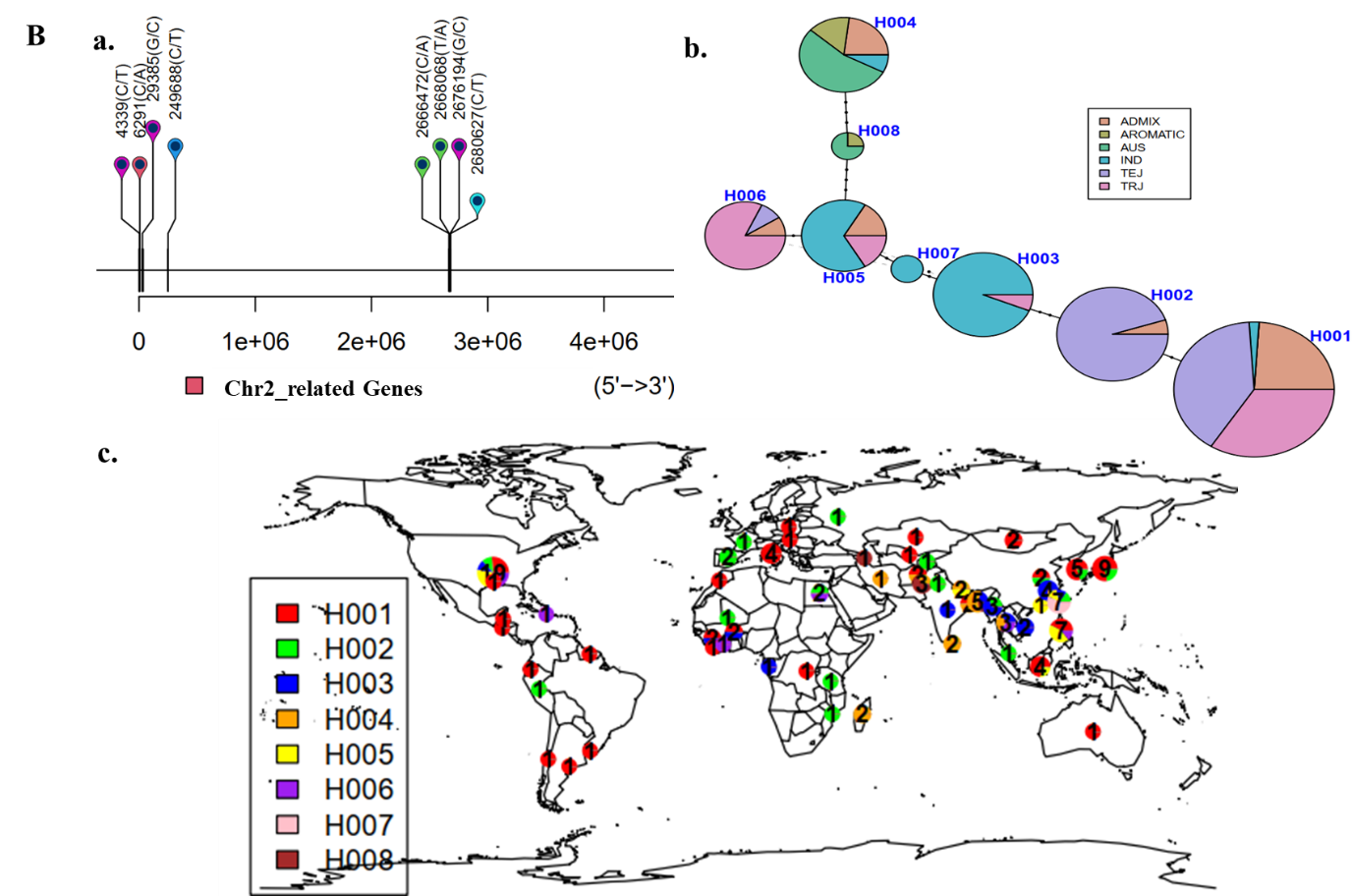**  **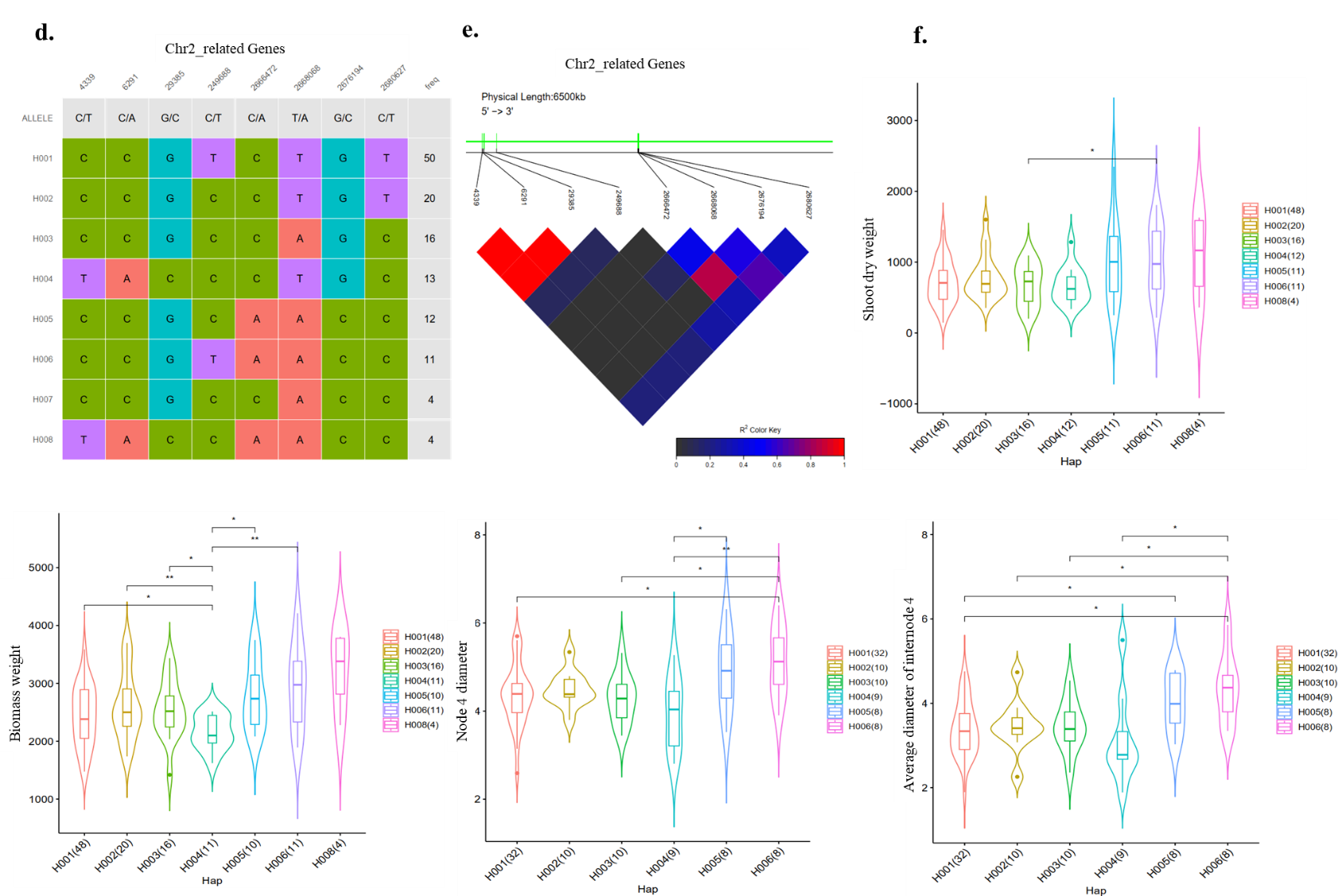** |
| **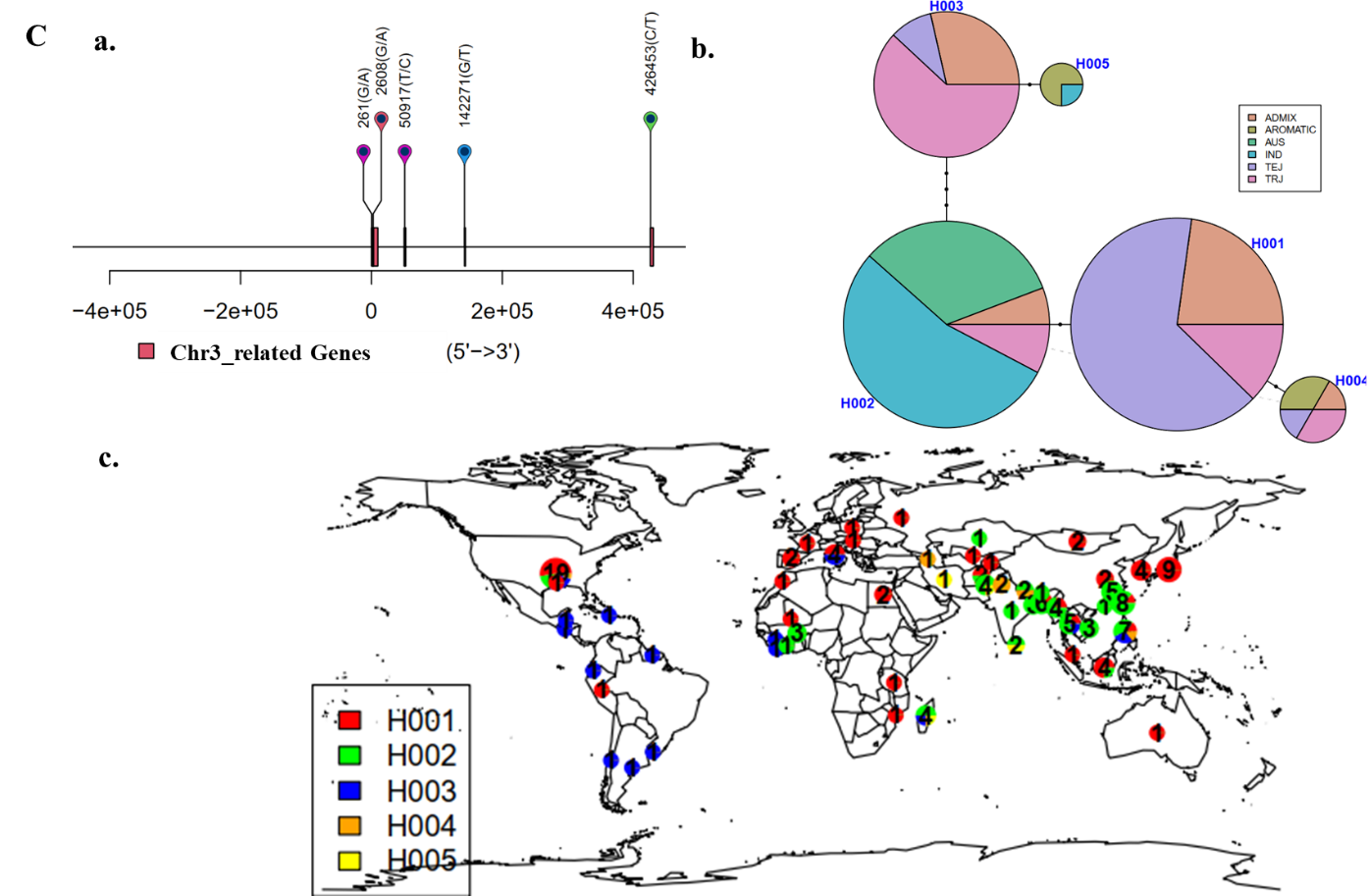**  **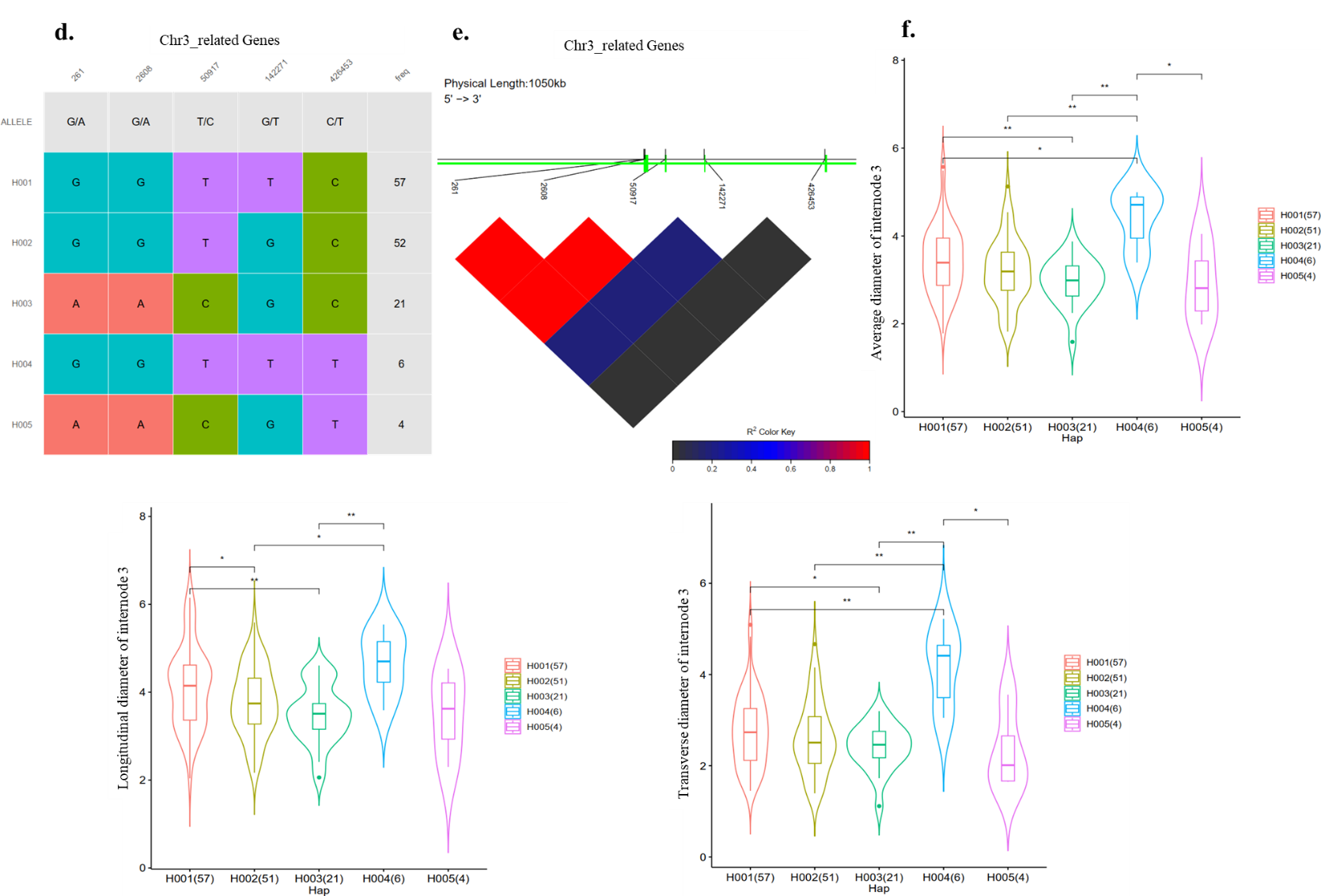** |
| **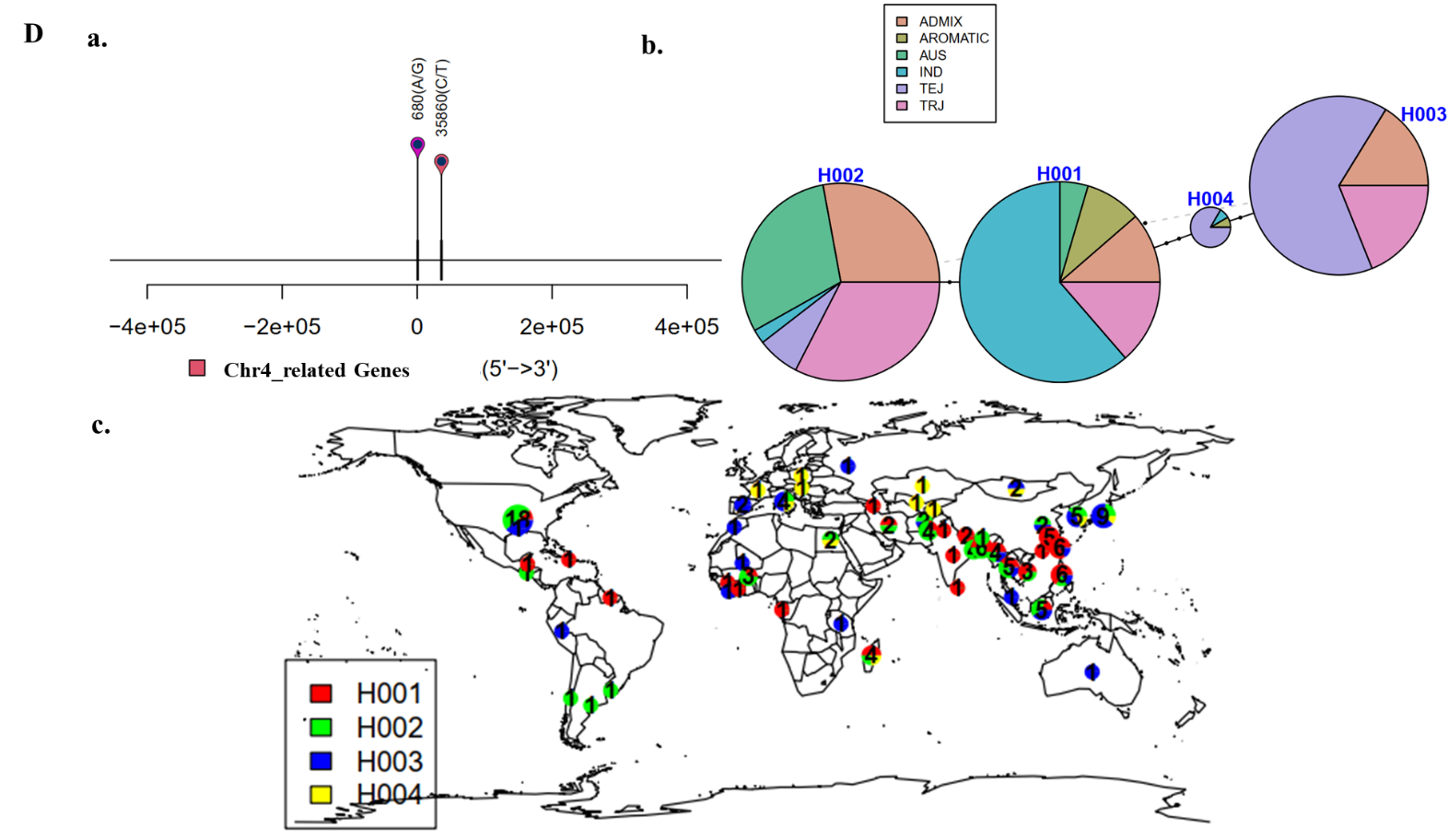**  **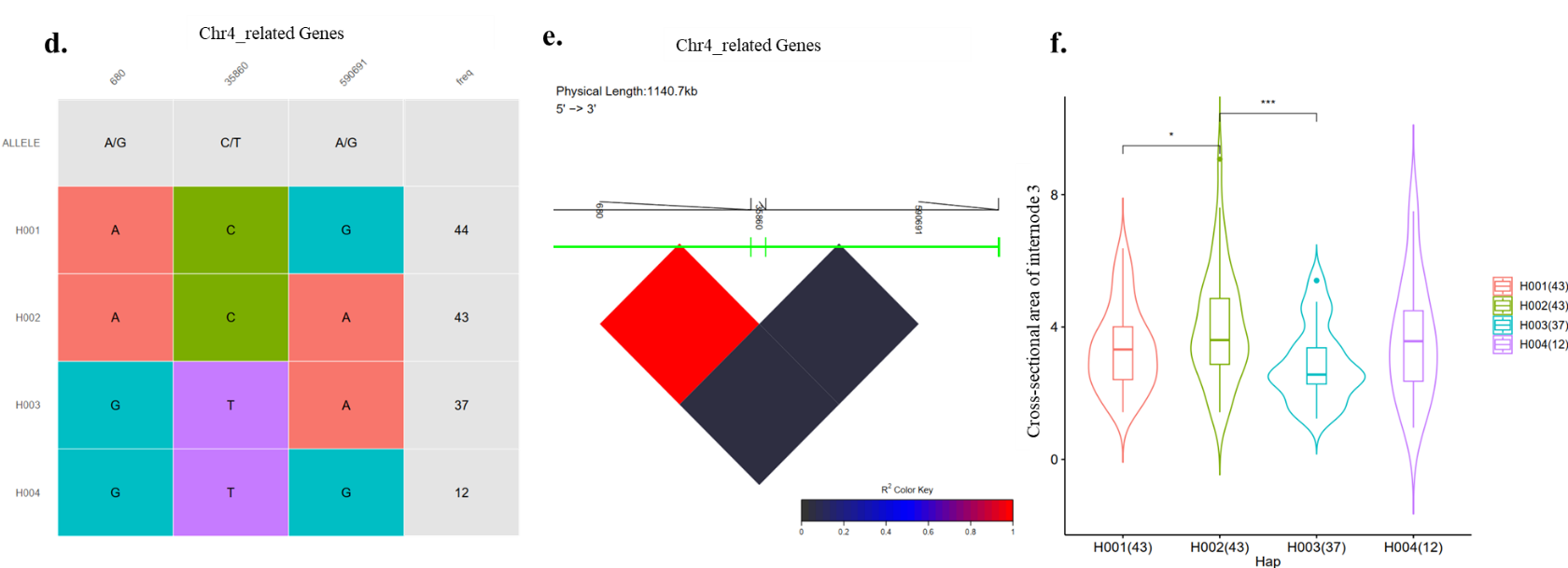** |
| **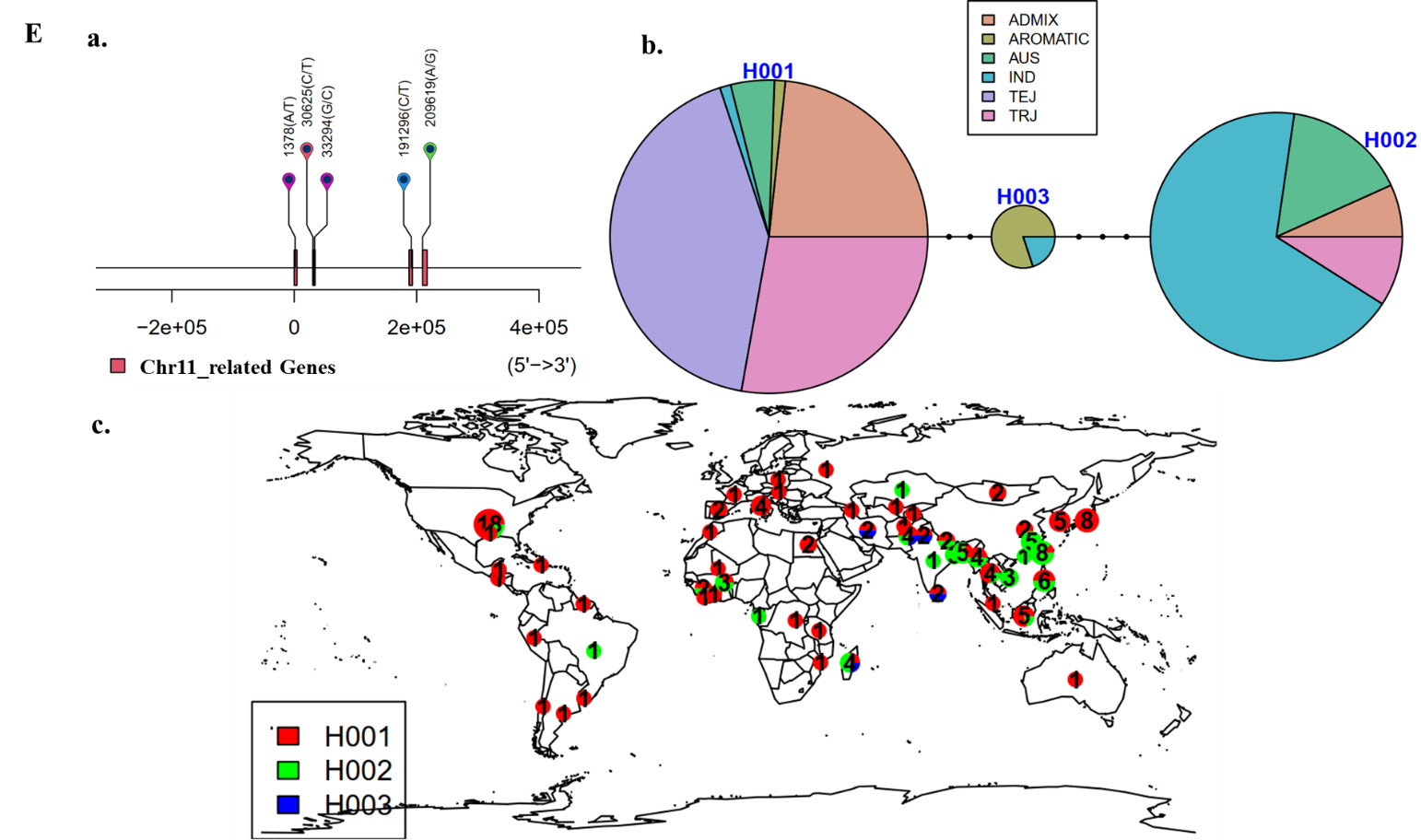**  **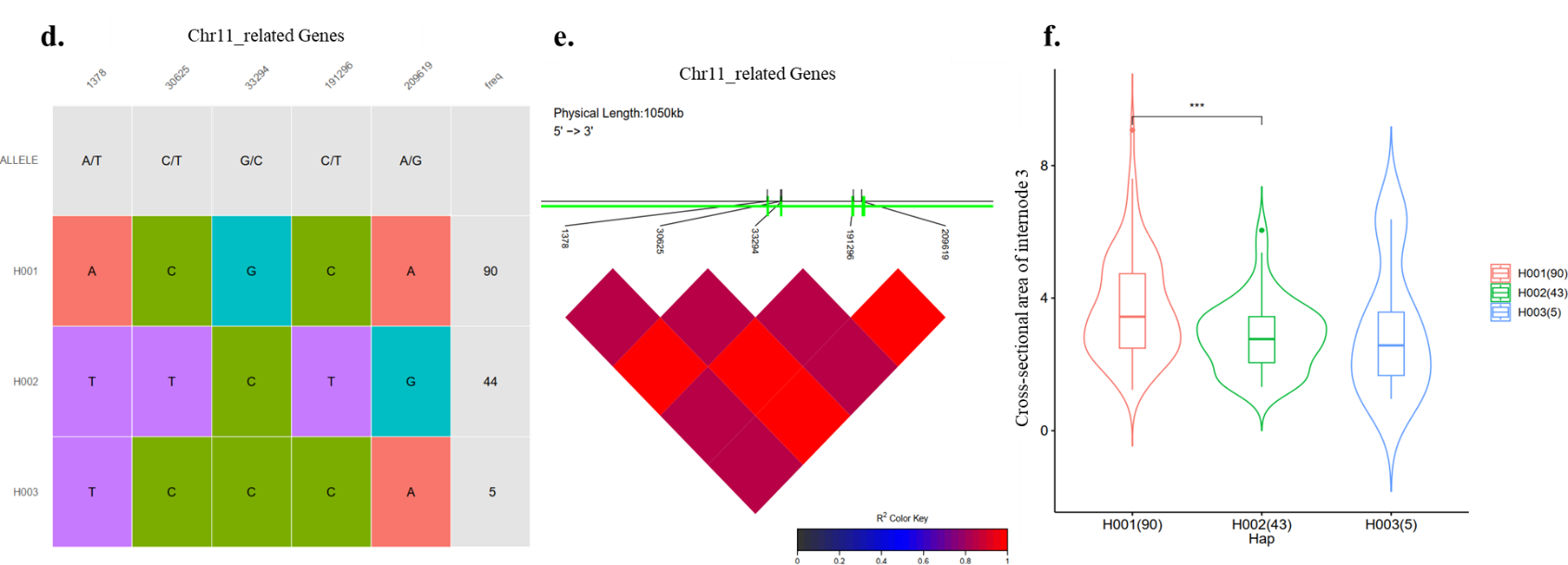**  **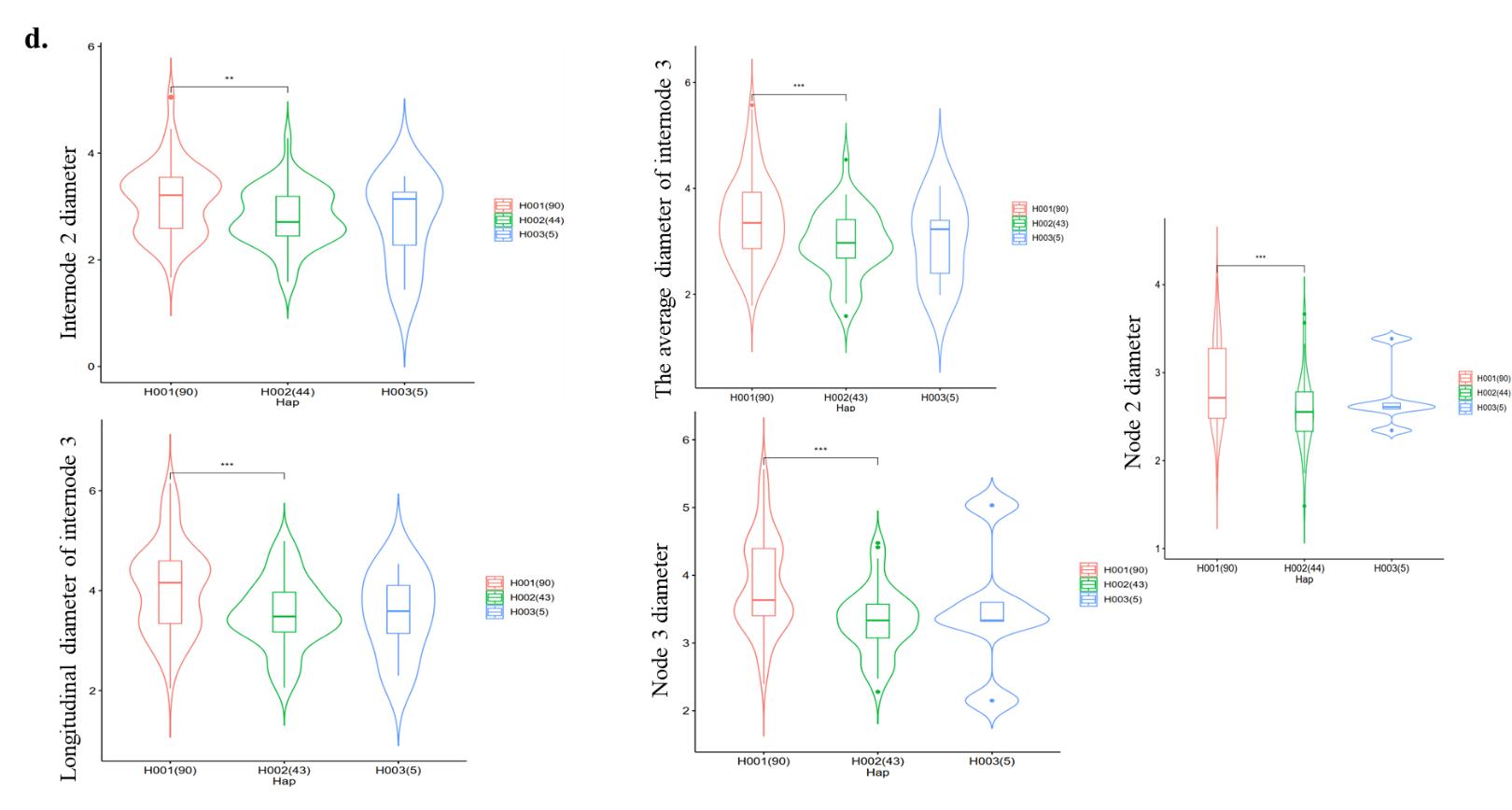** |

FIGURE S5| Visualization of genomic diversity and evolutionary network of candidate genes on chromosomes 1 (A), 2 (B), 3 (C), 4 (D), and 11 (E). a) Visualization of variant positions in candidate genes; the black line represents intergenic regions, and rectangles represent exons. Flags indicate variants, and the coordinates with alleles in parentheses are displayed above the gene model. Two or more transcripts are shown in different colors. b) Trait-related gene haplotype network. Each circle represents a haplotype, and its size indicates the number of accessions. The pies in different colors represent the frequency of different rice subpopulations in each haplotype. Symbols on the lines between haplotypes indicate the number of variants. c) Geo-distribution of major haplotypes of *chr1_ related genes* on chromosome 1, *chr2_ related genes*, *chr3_ related genes* on chromosome 3, *chr4_ related genes* on chromosome 4, and *chr11_ related genes* on chromosome 11. Circle size represents accession counts, and the pies in different colors represent the proportion of classified haplotype categories for relevant accessions derived from different eco-regions. The Arabic numeral inside each circle indicates the number of accessions at that location. d) Haplotype classification of trait-related genes; each line represents a haplotype, and colored columns represent loci. Haplotype frequency is shown in the last column. e) LD-block visualization of each trait-related genomic region. The gene model is presented at the top of the plot; the line represents the genomic region, and the rectangles represent exons. The oblique line below the gene model represents variants. The numbers indicate the positions of the variants. The LD-block with the color key lies at the bottom, where red indicates perfect LD and black indicates no LD. f) Phenotypic comparisons among accessions possessing different haplotypes; * indicates p < 0.05, ** indicates p < 0.01, *** indicates p < 0.001.


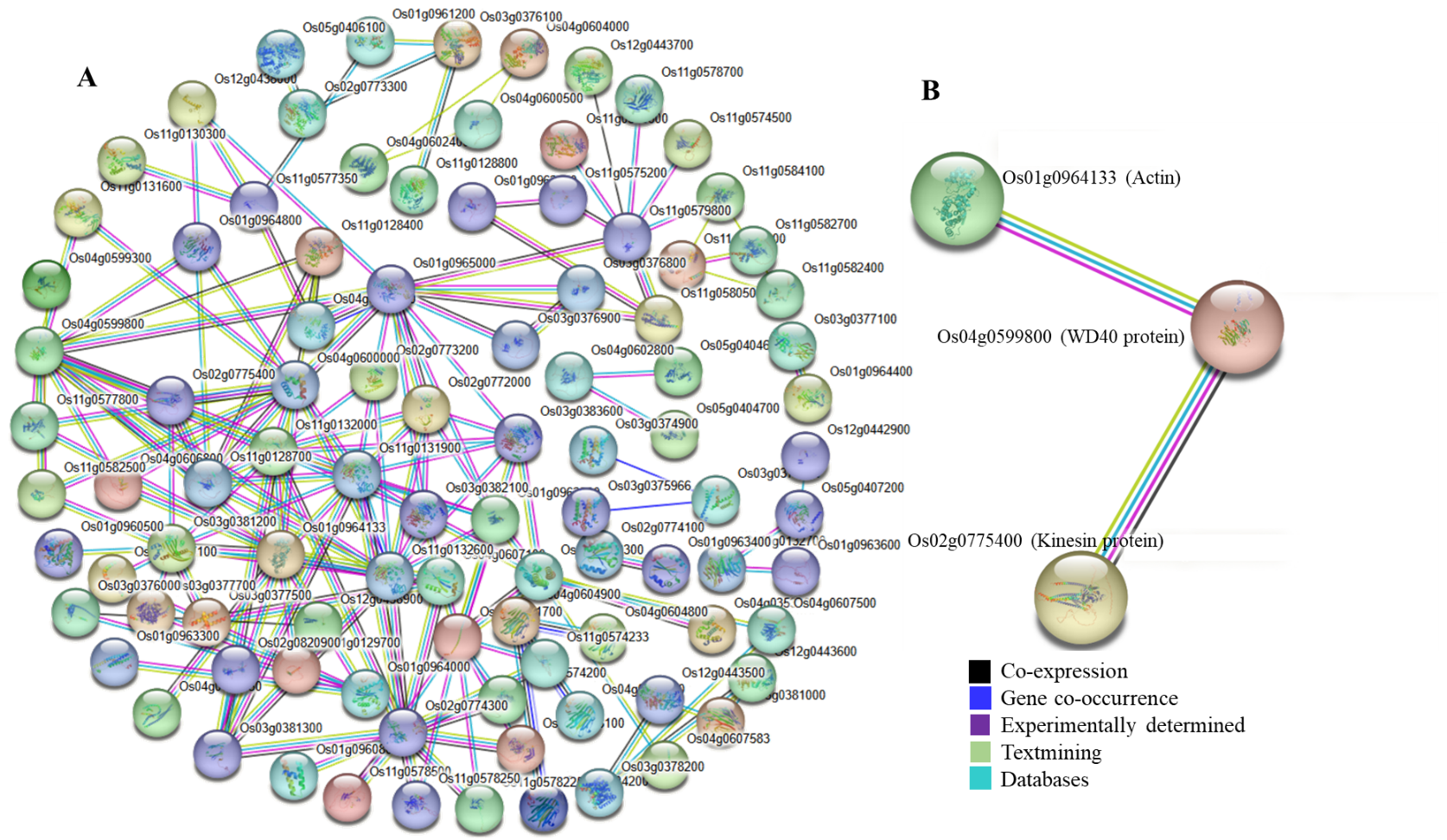


FIGURE S6) PPI network: A) Network among all 230 SNP-associated proteins within the identified QTLs. B) Network among the three candidate genes with the highest number of interactions. Interactions were derived from high-throughput experiments and curated databases with medium confidence (score ≥ 0.40). Nodes represent proteins, edges indicate interactions, line thickness reflects interaction strength, and colored lines denote different interaction types. Gene nodes with ribbon structures indicate the presence of 3D structural information.
