# Supplementary material for: Analysis of Genetic Variation of Rice Straw Characteristics and Its Influence on Biomass
Source: Plant Direct. 2026 Jan 6;10(1):e70134. doi: 10.1002/pld3.70134 (PMC12771682; doi:10.1002/pld3.70134)
Supplement: Supplementary file 3 — Table S1: Information of rice genotypes examined in the study. [file PLD3-10-e70134-s013.pdf]

Table S1. Information of rice genotypes examined in the study.

No.	No. in our pannel	Subpopulation	Location	Longitude	Latitude	Cultivar Name
1	g10	TEJ	United States-CA	-92.318115	34.662732	CS-M3
2	g13	ADMIX	Bangladesh	90.356331	23.684994	HabiganjBoro6
3	g14	IND	HongKong	114.109497	22.396428	HonChim
4	g15	TEJ	Poland	19.145136	51.919438	ItalicaCarolina
5	g16	AUS	India	87.121582	22.903081	Kasalath
6	g17	ADMIX	Indonesia	113.921327	-0.789275	KeritingTingii
7	g28	TRJ	Philippines	121.774017	12.879721	AsseYPung
8	g31	TEJ	India	75.344238	30.472664	Baber
9	g32	TEJ	Afghanistan	67.709953	33.93911	BaghlaniNangarhar
10	g33	TEJ	Spain	-3.74922	40.463667	Bahia
11	g35	ADMIX	Italy	12.56738	41.87194	Baldo
12	g36	AROMATIC	Pakistan	69.345116	30.375321	Basmati
13	g37	TRJ	India	87.121582	22.903081	Basmati217
14	g38	TEJ	France	2.213749	46.227638	Bellardone
15	g39	TEJ	Peru	-75.015152	-9.189967	Benllok
16	g46	ADMIX	Louisiana	-92.1450245	31.2448234	BlueRose
17	g47	TRJ	ElSalvador	-88.89653	13.794185	BoaVista
18	g49	TEJ	Spain	-3.74922	40.463667	Bombon
19	g50	TRJ	Belize	-88.49765	17.189877	BritishHondurasCreole
20	g51	TEJ	South Korea	127.766922	35.907757	BulZo
21	g54	TEJ	Former Soviet Union	37.633333	55.75	Caucasica
22	g55	TRJ	Argentina	-63.616672	-38.416097	Cenit
23	g57	TEJ	Mozambique	35.529562	-18.665695	Chibica
24	g58	IND	Vietnam	108.277199	14.058324	ChiemChanh
25	g70	ADMIX	Thailand	100.992541	15.870032	Dam
26	g71	ADMIX	Nepal	84.124008	28.394857	Darmali
27	g72	IND	Myanmar	95.956223	21.913965	Dawebyan
28	g73	IND	Taiwan	120.960515	23.69781	DeeGeoWooGen
29	g74	TRJ	United States	-92.318115	34.662732	Della
30	g75	TRJ	United States	-92.318115	34.662732	Delrex
31	g76	TEJ	Korea	127.766922	35.907757	Deokjeokjodo
32	g78	AUS	Bangladesh	90.356331	23.684994	DhalaShaitta
33	g79	IND	Guinea	-9.696645	9.945587	Djimoron
34	g80	ADMIX	Uruguay	-55.765835	-32.522779	DobleCarolinaRinaldoBarsani
35	g87	TEJ	Japan	138.252924	36.204824	EdomenScented
36	g88	IND	Myanmar	95.956223	21.913965	EMATAA16-34
37	g90	AROMATIC	Iran	53.688046	32.427908	Firooz
38	g91	TRJ	United States	-92.318115	34.662732	Fortuna
39	g92	TRJ	Burkina Faso	-1.561593	12.238333	FossaAv
40	g93	TEJ	Mali	-3.996166	17.570692	GambiakaSebela
41	g94	AUS	Afghanistan	67.709953	33.93911	GhatiKammaNangarhar
42	g95	AUS	Bangladesh	90.356331	23.684994	Ghorbhai
43	g96	TRJ	Indonesia	113.921327	-0.789275	GogoLempuk
44	g97	AUS	Bangladesh	90.356331	23.684994	Goria
45	g98	ADMIX	Indonesia	113.921327	-0.789275	GotakGatik
46	g99	IND	China	116.872559	27.902527	Guan-Yin-Tsan
47	g100	ADMIX	Guinea	-9.696645	9.945587	Guineandao
48	g108	TRJ	Haiti	-72.285215	18.971187	IguapeCateto
49	g109	IND	Philippines	121.774017	12.879721	IR36
50	g110	IND	Philippines	121.774017	12.879721	IR8
51	g111	IND	Nepal	84.124008	28.394857	IR-44595
52	g112	AUS	Bangladesh	90.356331	23.684994	Jamir
53	g114	IND	India	78.96288	20.593684	JC149
54	g115	AUS	India	87.121582	22.903081	Jhona349
55	g116	TEJ	Japan	138.252924	36.204824	Jouiku393G
56	g117	AUS	Bangladesh	90.356331	23.684994	Kachilon
57	g118	AUS	India	87.121582	22.903081	Kalamkati
58	g119	AUS	SriLanka	80.771797	7.873054	KalubalaVee
59	g120	TEJ	Japan	138.252924	36.204824	Kamenoo
60	g127	AUS	Thailand	100.992541	15.870032	KhaoTotLong227
61	g128	IND	Taiwan	120.960515	23.69781	Kiang-Chou-Chiu
62	g129	TEJ	Tanzania	34.888822	-6.369028	Kihogo
63	g130	TRJ	Philippines	121.774017	12.879721	Kinastano
64	g131	AROMATIC	Madagascar	46.869107	-18.766947	Kitrana508
65	g132	TEJ	Mongolia	103.846656	46.862496	KonSuito
66	g133	TEJ	Japan	138.252924	36.204824	Koshihikari
67	g134	TRJ	United States_CA	-92.318115	34.662732	L-202
68	g135	TRJ	Liberia	-9.429499	6.428055	LAC23
69	g136	TRJ	United States	-92.318115	34.662732	LadyWrightSeln
70	g137	TRJ	United States	-92.318115	34.662732	LaGrue
71	g138	IND	Sri Lanka	80.771797	7.873054	LD24
72	g139	TRJ	United States	-92.318115	34.662732	Lemont
73	g140	TEJ	Thailand	100.992541	15.870032	LeuangHawn
74	g147	ADMIX	United States-CA	N	N	M202
75	g148	TEJ	Japan	138.252924	36.204824	Mansaku
76	g149	TEJ	Italy	12.56738	41.87194	Maratelli
77	g150	TEJ	Tajikistan	71.276093	38.861034	Melanotrix
78	g151	IND	China	116.872559	27.902527	MingHui
79	g152	IND	China	116.872559	27.902527	MUDGO
80	g153	TRJ	Chile	-71.542969	-35.675147	Niquen
81	g154	IND	United States	-92.318115	34.662732	Nira
82	g155	TEJ	Japan	138.252924	36.204824	Norin20
83	g156	ADMIX	United States	-92.318115	34.662732	Nova
84	g166	IND	Taiwan	120.960515	23.69781	Pagaiyahan
85	g169	IND	China	116.872559	27.902527	Pao-Tou-Hung
86	g170	IND	Taiwan	120.960515	23.69781	Pappaku
87	g171	IND	Brazil	-51.92528	-14.235004	ParaibaChinesNova
88	g172	TRJ	Cote D'Ivoire	-5.54708	7.539989	PateBlancMn1
89	g174	ADMIX	Australia	133.775136	-25.274398	PatoDeGallinazo
90	g175	AUS	Myanmar	95.956223	21.913965	PaungMalaung
91	g176	IND	Taiwan	120.960515	23.69781	Peh-Kuh
92	g177	IND	Taiwan	120.960515	23.69781	Peh-Kuh-Tsao-Tu
93	g178	AUS	Bhutan	90.433601	27.514162	Phudugey
94	g185	TEJ	Japan	138.252924	36.204824	RikutoKemochi
95	g187	ADMIX	Italy	12.56738	41.87194	RinaldoBersani
96	g188	ADMIX	Burkina Faso	-1.561593	12.238333	RizLocal
97	g189	ADMIX	Madagascar	46.869107	-18.766947	Rojofotsy738
98	g190	TEJ	Hungary	19.503304	47.162494	Romanica
99	g191	IND	Vietnam	108.277199	14.058324	RTS4
100	g192	TEJ	Egypt	30.802498	26.820553	SabIni
101	g193	IND	Bangladesh	90.356331	23.684994	Sabharaj
102	g194	AROMATIC	Azerbaijan	47.576927	40.143105	SadriBelyi
103	g195	ADMIX	Iran	53.688046	32.427908	SadriTorMisri
104	g196	ADMIX	Mongolia	103.846656	46.862496	Saku
105	g197	ADMIX	Korea	127.766922	35.907757	Sanbyang-Daeme
106	g198	AUS	Pakistan	69.345116	30.375321	SanthiSufaid
107	g200	AUS	Pakistan	69.345116	30.375321	Sathi
108	g208	TEJ	Japan	138.252924	36.204824	Shirogane
109	g209	TEJ	United States	-92.318115	34.662732	Shoemed
110	g210	IND	Thailand	100.992541	15.870032	ShortGrain
111	g211	IND	Indonesia	113.921327	-0.789275	Sigadis
112	g212	TRJ	Philippines	121.774017	12.879721	SinampagaSelection
113	g213	IND	BurkinaFaso	-1.561593	12.238333	SintaneDiofor
114	g214	TEJ	Myanmar	95.956223	21.913965	Sitpwa
115	g215	TEJ	Malaysia	101.975766	4.210484	SriMalaysiaDua
116	g216	AUS	Pakistan	69.345116	30.375321	Sufaid
117	g217	TRJ	Egypt	30.802498	26.820553	Sultani
118	g218	IND	Kazakhstan	66.923684	48.019573	Sundensis
119	g219	TEJ	Korea	127.766922	35.907757	Suweon
120	g220	IND	China	116.872559	27.902527	SzeGuenZim
121	g227	TEJ	Taiwan	120.960515	23.69781	Taipei309
122	g228	IND	Gabon	11.609444	-0.803689	Tchibanga
123	g230	TRJ	Indonesia	113.921327	-0.789275	TiaBura
124	g232	ADMIX	Japan	138.252924	36.204824	TokyoShinoMochi
125	g236	TEJ	Morocco	-7.09262	31.791702	TriompheDuMaroc
126	g237	TEJ	Ecuador	-78.183406	-1.831239	TropicalRice
127	g238	ADMIX	Madagascar	46.869107	-18.766947	Tsipala421
128	g239	TEJ	Uzbekistan	64.585262	41.377491	Uzbekskij2

README

Subpopulation	Description
TEJ	tropical japonica
ADMIX	highly admixed accessions (that are created by mixing within (not between) subspecies)
IND	indica
TEJ	temperate japonica
AUS	aus
AROMATIC	aromatic

129	g240	ADMIX	Madagascar	46.869107	-18.766947	VaryVato462
130	g248	AUS	Thailand	100.992541	15.870032	KhaoPahkMaw
131	g249	TEJ	China	114.23584	32.718285	WC6
132	g250	ADMIX	United States	-92.318115	34.662732	BlueRoseSupreme
133	g251	TRJ	Zaire	25.7752703	-2.425777	Manzano
134	g252	ADMIX	United States	-92.318115	34.662732	Nortai
135	g253	TEJ	Italy	12.56738	41.87194	Romeo
136	g254	TRJ	Philippines	121.774017	12.879721	Sinaguing
137	g255	TEJ	China	114.23584	32.718285	SungLiao2
138	g256	IND	Vietnam	108.277199	14.058324	RTS12
139	g257	IND	Taiwan	120.960515	23.69781	Shuang-Chiang
140	g258	IND	Philippines	121.774017	12.879721	Jasmine85
141	g259	TRJ	Suriname	-56.027783	3.919305	Wanica
142	g267	ADMIX	United States	-92.318115	34.662732	Early
143	g269	TEJ	South Korea	127.766922	35.907757	Heukgyeong
144	g271	TRJ	United States	-92.318115	34.662732	Katy
145	g272	TRJ	United States	-92.318115	34.662732	Kaybonnet
146	g273	AROMATIC	Peru	-75.015152	-9.189967	Lambayeque1
147	g276	TRJ	United States	-92.318115	34.662732	Saber
148	g278	AROMATIC	India	75.344238	30.472664	Pankhari203
149	g279	ADMIX	United States	-92.318115	34.662732	Bengal
