# Supplementary material for: Analysis of Genetic Variation of Rice Straw Characteristics and Its Influence on Biomass
Source: Plant Direct. 2026 Jan 6;10(1):e70134. doi: 10.1002/pld3.70134 (PMC12771682; doi:10.1002/pld3.70134)
Supplement: Supplementary file 4 — Table S2: Estimating the heritability of the traits. [file PLD3-10-e70134-s014.pdf]

Table S2. Estimating the heritability of the traits.

Traits	h2b	h2n	Vg	Va
Node 1 diameter	0.6730567	0.4614785	0.0693723	0.04756482
Node 2 diameter	0.8430357	0.7497722	0.2293303	0.20396
Node 3 diameter	0.8891514	0.8238269	0.4475781	0.4146953
Node 4 diameter	0.881047	0.7889797	0.4608369	0.4126805
Internode 1 diameter	0.8352758	0.7370874	0.1651103	0.1457012
Internode 2 diameter	0.9036462	0.847709	0.3983183	0.3736618
The average diameter of internode 3	0.8389955	0.7421239	0.5200113	0.4599701
The average diameter of internode 4	0.9138349	0.8475904	0.6085106	0.5643993
Longitudinal diameter of internode 3	0.8777114	0.8052769	0.6517679	0.5979798
Transverse diameter of internode 3	0.6053765	0.3345448	0.515504	0.2848792
Longitudinal diameter of internode 4	0.8455894	0.7236374	0.7121835	0.6094715
Transverse diameter of internode 4	0.7848746	0.6095709	0.6361638	0.4940751
Internode 3 thickness	0.9750849	0.9608927	18773.93	18500.68
Cross-section area of internode 3	0.9655109	0.9458313	2.113921	2.070834
Plant height	0.9564383	0.930136	245.7204	238.9631
Stem length	0.9534146	0.9255452	183.1189	177.7662
Panicle length	0.8787163	0.8066706	11.59038	10.64009
Internode 1 length	0.8502392	0.7615157	17.78028	15.92488
Internode 2 length	0.9024365	0.8457725	18.40282	17.24731
Internode 3 length	0.922602	0.877256	20.73807	19.71879
Internode 4 length	0.9401306	0.8945175	9.434727	8.976975
Panicle dry weight	0.8929228	0.8300891	152954.6	142191.4
Shoot dry weight	0.9744407	0.9582914	167310.1	164537.2
Internode 1 dry weight	0.9110041	0.85937	4157.142	3921.522
Internode 2 dry weight	0.9306525	0.8907695	11444.64	10954.18
Internode 3 dry weight	0.9295041	0.8884464	19106.16	18262.2
Internode 4 dry weight	0.9102949	0.8409246	18522.2	17110.69
Node 1 dry weight	0.9019868	0.8442837	2.022762	1.893359
Node 2 dry weight	0.9327958	0.8937817	114.2236	109.4462
Node 3 dry weight	0.9386219	0.9028608	491.1816	472.4678
Total weight of the aerial plant part(Bi	0.936278	0.8951645	373130.2	356745.4

README

Column	Description
Traits	measured parameters(statistics)
h2b	broad-sense heritability
h2n	narrow-sense heritability
Vg	genotypic variance
Va	additive variance
