# Supplementary material for: Analysis of Genetic Variation of Rice Straw Characteristics and Its Influence on Biomass
Source: Plant Direct. 2026 Jan 6;10(1):e70134. doi: 10.1002/pld3.70134 (PMC12771682; doi:10.1002/pld3.70134)
Supplement: Supplementary file 5 — Table S3: Descriptive statistics for 32 morphological traits. [file PLD3-10-e70134-s005.pdf]

Table S3. Descriptive statistics for 32 morphological traits

Statistics	Node 1 diameter	Node 2 dia	Node 3 dia	Node 4 dia	Internode	Internode	The averag
Average	1.6264	2.7617	3.6792	4.4365	2.0913	3.0048	3.27921
Minimum	0.84	1.02	2.13	2.59	1.06	1.2	1.505
Maximum	2.96	4.2	5.76	6.7	3.4	5.08	8.025
Std. Deviation	0.32056	0.52058	0.70806	0.72098	0.44377	0.66257	0.78578
df	148	148	148	148	148	148	148
CV	19.70978849	18.84998	19.24494	16.2511	21.21982	22.05039	23.96248

README

Column	Description
Statistics	measured parameters
Average	Average
Minimum	Minimum
Maximum	Maximum
Std. Deviation	standard deviation
df	degrees of freedom
CV	coefficient of variation

The averag	Longitudin	Transverse	Longitudin	Transverse	Internode :	Cross-secti	Plant heigh	Stem lengtl
3.60185	3.8855	2.6729	4.3264	2.869588	381.6353	3.4252	77.201	58.145
1.825	1.9	1.01	2.2	1.15	140.036	0.94	40.7	25.4
5.93	6.5	12	6.58	5.64	890.531	9.14	123.2	96.7
0.813364	0.86002	0.92153	0.91498	0.897779	138.4517	1.47644	15.9916	13.8277
148	148	148	148	148	148	148	148	148
22.58184	22.13409	34.47679	21.14876	31.28599	36.27854	43.10522	20.71424	23.78141

Panicle len	Number of	Internode 1	Internode 2	Internode 3	Internode 4	Panicle dry	Shoot dry	Internode 1
19.209	3.66	23.138	17.447	12.156	5.773	1803.76	793.217	118.33
11	2	11	4.5	0.7	1	1080	146.4	10
30	5	37	25	22.5	15	3030	2859.9	320
3.6245	0.541	4.5642	4.5066	4.7311	3.1567	413.039	413.4272	67.413
148	148	148	148	148	148	148	148	148
18.86876	14.78142	19.72599	25.83023	38.91987	54.68041	22.89878	52.12032	56.97034

Internode 2	Internode 3	Internode 4	Node 1 dry	Node 2 dry	Node 3 dry	Biomass weight
204.88	239.33	165.94	3.874	26.867	48.877	2600.092
20	10	10	1.4	4.7	12.5	1336.7
590	840	910	9.3	79.3	140.3	5279.9
110.662	143.069	142.16	1.4945	11.0426	22.8271	629.8956
148	148	148	148	148	148	148
54.01308	59.77897	85.66952	38.5777	41.10098	46.70315	24.2259
