# Supplementary material for: Analysis of Genetic Variation of Rice Straw Characteristics and Its Influence on Biomass
Source: Plant Direct. 2026 Jan 6;10(1):e70134. doi: 10.1002/pld3.70134 (PMC12771682; doi:10.1002/pld3.70134)
Supplement: Supplementary file 6 — Table S4: The characteristics of significant SNPs extracted from GWAS. [file PLD3-10-e70134-s001.pdf]

Table S4. The characteristics of significant SNPs extracted from GWAS.

SNP	Chromosome	Position	REF	ALT	Effect	SE	FarmCPU_PVALUE	(-)Log_PVALUE
id10007177	10	22612177	C	G	198.5624942	24.63214549	1.65E-14	13.78296787
id1027609	1	42358018	G	A	120.9078799	19.37150907	1.59E-10	9.799785226
id3005558	3	10628270	A	G	111.8524087	20.43797015	1.34E-09	8.873134495
id4006198	4	20233912	T	A	-56.50941455	10.52447948	6.15E-09	8.211317487
id2009738	2	23775529	G	A	-70.02970546	14.33033017	6.83E-08	7.16558862
id2014525	2	32579679	C	T	-541.0099036	102.6583155	5.36E-07	6.270785741
id2014530	2	32581631	C	A	-541.0099036	102.6583155	5.36E-07	6.270785741
id2014550	2	32604725	G	C	-541.0099036	102.6583155	5.36E-07	6.270785741
id2014606	2	32825028	C	T	-228.7373541	55.09959289	7.02E-07	6.15382139
id9003485	9	12664532	G	A	381.7380477	73.30095369	7.09E-07	6.149543556
id5009418	5	22082907	C	A	-671.0847917	130.3666914	9.27E-07	6.033100018
dd1002393	1	42608865	T	C	82.06542075	18.35905257	1.19E-06	5.923929049
id4011518	4	32902367	A	G	0.450158774	0.089951081	1.68E-06	5.775213919
id1007155	1	9633974	G	T	426.0653254	85.95865701	2.14E-06	5.668739264
id1007156	1	9634128	A	T	426.0653254	85.95865701	2.14E-06	5.668739264
id2016156	2	35258870	T	C	0.541455142	0.10691164	2.35E-06	5.62934955
dd1001700	1	42340167	T	C	375.3927103	76.52046747	2.67E-06	5.573194102
id1000027	1	172923	C	T	5.468448107	1.117434663	2.70E-06	5.568343391
id1002770	1	3397754	T	A	-55.90756547	14.39956815	2.90E-06	5.537407224
id1001247	1	1511711	T	G	-147.9434633	30.32907604	2.91E-06	5.535794771
id11000392	11	1484113	A	G	-1.161388981	0.239291371	3.24E-06	5.489908278
id12006815	12	20913496	T	G	446.6789733	92.41998025	3.65E-06	5.437777209
dd1001737	1	42355255	A	G	348.2542077	72.30716179	3.92E-06	5.406590842
id4004869	4	16929510	G	A	439.6548463	91.34491791	3.97E-06	5.400689315
id11000413	11	1512711	G	A	-1.185725361	0.246911384	4.03E-06	5.394416563
id3007392	3	14788478	G	A	-0.513847201	0.107169365	4.16E-06	5.380471119
id3007405	3	14839134	T	C	-0.513847201	0.107169365	4.16E-06	5.380471119
id1025227	1	39719133	G	A	-253.8139286	61.42446944	4.21E-06	5.376151152
ud7001914	7	26037194	G	A	-0.834218707	0.169623983	4.22E-06	5.374824659
id12005213	12	14487883	C	G	244.9721748	51.23975332	4.56E-06	5.341173176
ud7001914	7	26037194	G	A	-0.803348783	0.164059849	4.59E-06	5.337998337
id9000693	9	2748188	C	T	-43.68639217	11.31147198	4.86E-06	5.313055072
id12005215	12	14488566	T	C	228.8631586	48.17611908	5.18E-06	5.285359666
id3007392	3	14788478	G	A	-0.45743845	0.096862795	5.66E-06	5.247146963
id3007405	3	14839134	T	C	-0.45743845	0.096862795	5.66E-06	5.247146963
id8004716	8	17729217	G	A	-229.8509752	57.39951006	5.74E-06	5.241343503
id7001323	7	7660553	T	C	2.427657627	0.515046895	5.85E-06	5.232985123
id4011523	4	32928401	G	C	0.457714469	0.097177907	5.97E-06	5.224240715
ud4002236	4	32903133	C	T	0.439741179	0.093687361	6.39E-06	5.194258733
wd12002512	12	14659244	G	A	0.38326129	0.081768469	6.57E-06	5.182261752
id5011128	5	24169108	C	A	-2.818090056	0.601668301	6.63E-06	5.178565758
ud7001914	7	26037194	G	A	-0.64249936	0.138050571	7.55E-06	5.121921472
id11002182	11	5357024	G	A	-211.452655	58.94324419	7.56E-06	5.121322582
id6015588	6	27433303	T	C	414.8669521	89.42089024	8.26E-06	5.083044194
id2016156	2	35258870	T	C	0.532007284	0.112278228	8.56E-06	5.067496402
id9007879	9	22755878	T	C	-777.1025507	168.1606686	8.91E-06	5.050014483
id8004106	8	15314965	G	A	0.136316906	0.029551519	8.92E-06	5.049392879
id1001247	1	1511711	T	G	-396.4175667	85.97120699	9.30E-06	5.031743141
id11001392	11	3661173	G	A	436.5249135	94.72597587	9.40E-06	5.026776721
id4011513	4	32897775	A	G	0.423594184	0.092102394	9.50E-06	5.02231632
id12005213	12	14487883	C	G	0.419153306	0.091446455	1.01E-05	4.994214155
id1018646	1	30894634	G	A	0.539512259	0.118125488	1.08E-05	4.964845972
id5004367	5	8532207	T	C	-49.83203625	14.14064288	1.18E-05	4.926493943
ud3000828	3	14790825	G	A	-0.485372851	0.107194169	1.28E-05	4.894369818
id4010231	4	30189848	G	A	0.679811546	0.150607515	1.35E-05	4.869037321
id11000293	11	1307788	G	C	-0.603944273	0.134130797	1.42E-05	4.849169345
wd4002500	4	17002657	A	G	116.4697723	25.8965637	1.45E-05	4.839993843
id4004892	4	17011401	T	C	116.4697723	25.8965637	1.45E-05	4.839993843
id4004901	4	17013663	A	G	116.4697723	25.8965637	1.45E-05	4.839993843
id4004929	4	17047786	T	C	116.4697723	25.8965637	1.45E-05	4.839993843
id2003149	2	6015346	G	A	-1.133962889	0.252239079	1.46E-05	4.83659184
id11000275	11	1275872	A	T	-0.4884093	0.109033853	1.56E-05	4.807869513
id3008667	3	17772284	C	A	-337.6027245	95.04323125	1.64E-05	4.783989694
ud7001914	7	26037194	G	A	-0.68629951	0.153927824	1.69E-05	4.770915492
id11008239	11	21612962	C	T	-0.403752231	0.090645189	1.73E-05	4.76317492
ud3000828	3	14790825	G	A	-0.430757215	0.096905855	1.79E-05	4.747101282
id1000027	1	172923	C	T	0.401079807	0.090399426	1.85E-05	4.732347572
id1000027	1	172923	C	T	10.3018399	2.322250128	1.86E-05	4.731278607
id2016156	2	35258870	T	C	0.547831932	0.120790123	1.87E-05	4.727693985
ud12000862	12	14693039	C	A	0.382814155	0.086366087	1.88E-05	4.724778084
id11008239	11	21612962	C	T	-0.379685139	0.085660503	1.88E-05	4.724746248
wd12000455	12	5552455	G	T	-1.731509415	0.391578104	2.00E-05	4.699963073
id12005213	12	14487883	C	G	0.148163386	0.033584861	2.04E-05	4.690072763
id11000275	11	1275872	A	T	-0.605328433	0.137715391	2.19E-05	4.659812368
id1024348	1	38363629	C	T	0.414579629	0.094496515	2.26E-05	4.645350115
id11008193	11	21542705	T	C	0.150698741	0.03437023	2.28E-05	4.642610371
id4010220	4	30145846	A	G	-0.795553439	0.181526454	2.31E-05	4.637173361
id4010225	4	30181026	C	T	-0.795553439	0.181526454	2.31E-05	4.637173361
id11000275	11	1275872	A	T	-0.457675759	0.104585946	2.36E-05	4.627734104
ud7000591	7	7718166	A	G	2.85869852	0.655398356	2.50E-05	4.602745297
id7001407	7	7841438	G	T	2.85869852	0.655398356	2.50E-05	4.602745297
id4010433	4	30735857	A	G	172.4769962	46.92769015	2.60E-05	4.585010999
wd10002398	10	10659686	T	C	0.555439045	0.127641024	2.61E-05	4.583060196
wd12002512	12	14659244	G	A	0.379071269	0.087453657	2.80E-05	4.553442515
ud4002236	4	32903133	C	T	0.387310372	0.08939645	2.82E-05	4.549907479
id1000027	1	172923	C	T	49.8792554	11.52448326	2.86E-05	4.544146138
id3006551	3	12696199	T	C	40.96553757	11.90458413	3.07E-05	4.512239373
id11000390	11	1465790	C	T	-1.033669932	0.23986723	3.09E-05	4.509772586
id7004429	7	24312823	A	C	3.242384923	0.734904437	3.13E-05	4.504921181
id7004434	7	24316717	C	T	3.242384923	0.734904437	3.13E-05	4.504921181
id7004968	7	26071456	C	T	-0.851609947	0.193572313	3.13E-05	4.504426231
id5008100	5	19715820	C	G	2.244918111	0.521568718	3.14E-05	4.5026451
id5008122	5	19736445	G	A	2.244918111	0.521568718	3.14E-05	4.5026451
id11001839	11	4628799	T	A	0.544861345	0.126798404	3.25E-05	4.488562011
id1013814	1	23790449	A	G	-39.10333085	10.54600147	3.29E-05	4.483335152
ud7001914	7	26037194	G	A	-109.8749212	25.6131855	3.34E-05	4.475986875
id2016104	2	35241812	C	A	0.435378478	0.099363683	3.35E-05	4.475510967
ud12000862	12	14693039	C	A	0.391551736	0.091882148	3.74E-05	4.427254932
id11000275	11	1275872	A	T	-0.340375504	0.079905112	3.75E-05	4.42607809
id2011183	2	25630715	C	A	-259.7821133	75.24735343	3.85E-05	4.415069832
id7001393	7	7837751	T	C	2.906815275	0.683450501	3.85E-05	4.414748477
id12005326	12	14954178	G	A	0.188467906	0.044372084	3.94E-05	4.404966582
id12005213	12	14487883	C	G	0.434158244	0.102371057	4.05E-05	4.392239421
id9002735	9	9640008	G	A	-0.505593283	0.119226557	4.06E-05	4.391528152
id11008437	11	21976195	G	A	-0.418080899	0.098691414	4.13E-05	4.38406121
id5014595	5	28995509	T	C	240.3344196	70.86288559	4.22E-05	4.374436328
id8007520	8	27424246	C	T	-1.057007607	0.244979543	4.29E-05	4.367116742
id2016129	2	35251534	G	C	0.430488277	0.099886181	4.37E-05	4.359242326
wd12002512	12	14659244	G	A	0.387450908	0.091871957	4.45E-05	4.351708461
id8004106	8	15314965	G	A	48.84097125	11.58822596	4.48E-05	4.348995365
id5010361	5	23248349	C	T	3.513110108	0.817563894	4.78E-05	4.320468147
id5010375	5	23252288	G	A	3.513110108	0.817563894	4.78E-05	4.320468147
id4011518	4	32902367	A	G	0.36502932	0.086954748	4.80E-05	4.318744394
id7004968	7	26071456	C	T	-1.166813802	0.278046321	4.83E-05	4.316280712
dd11000522	11	21978827	G	A	-0.378381742	0.090182671	4.84E-05	4.315011631
id11000286	11	1305119	C	T	-1.068385449	0.254933857	4.94E-05	4.306706047
id11008603	11	22106066	G	T	-0.65256141	0.155826988	5.00E-05	4.301440064

README

Column	Description
SNP	uniqe identifier for the SNP
Chromosoı	chromosome number where the SNP is located
Position	genomic position of the SNP
REF	base on the reference genome
Effect	estimated effect size from GWAS analysis
ALT	alternative base considered as SNP
SE	standard error of the effect estimate
FarmCPU	P-value from the FarmCPU GWAS model
PVALUE	statistical significance

id11008620	11	22112337	T	C	0.368477832	0.088013053	5.02E-05	4.299568683
id3007659	3	15214670	C	T	0.761555403	0.182070524	5.09E-05	4.29299434
id2016156	2	35258870	T	C	0.418941762	0.098376947	5.28E-05	4.276991769
id9002643	9	9228486	T	C	0.712379379	0.171049482	5.46E-05	4.262541103
id11000293	11	1307788	G	C	-0.508272757	0.122115686	5.52E-05	4.258261697
id4011523	4	32928401	G	C	0.387791195	0.093206852	5.55E-05	4.255425007
id1020630	1	33172139	C	T	-10.70213678	2.568805004	5.61E-05	4.251126452
id11003684	11	9765954	A	G	-65.53985262	19.23507816	5.68E-05	4.245805547
id12005213	12	14487883	C	G	33.19560851	8.004748717	5.83E-05	4.234160474
wd12002512	12	14659244	G	A	0.289285945	0.069787069	5.87E-05	4.231254087
id5009418	5	22082907	C	A	-139.9072381	33.86959153	6.21E-05	4.206832871
id5008060	5	19664904	T	A	2.133185308	0.517017885	6.33E-05	4.198727368
id4011513	4	32897775	A	G	0.363684406	0.08813367	6.34E-05	4.198109454
id2016152	2	35255967	C	T	-0.611576352	0.145417603	6.42E-05	4.192709081
id12005213	12	14487883	C	G	0.404144026	0.098077895	6.48E-05	4.188263775
id1002058	1	2605844	C	T	1.987071492	0.482666522	6.55E-05	4.183482144
id11000272	11	1273238	C	A	-0.496960703	0.120694891	6.56E-05	4.182984618
id1000027	1	172923	C	T	0.215697294	0.052403116	6.57E-05	4.182230094
id7000727	7	5073227	T	A	-1.330463629	0.316955899	6.61E-05	4.179969932
id8003991	8	14876879	A	G	0.638134226	0.152486905	6.92E-05	4.15970576
id7003855	7	22593381	C	T	-0.433134286	0.105555329	6.93E-05	4.159354413
id11000293	11	1307788	G	C	-0.960669058	0.234155589	6.95E-05	4.158198828
id1000529	1	652340	A	G	2.531381945	0.604122955	7.06E-05	4.151343956
id3007489	3	14930488	G	T	0.441339154	0.10768396	7.06E-05	4.151128859
id3007509	3	14934867	T	A	0.441339154	0.10768396	7.06E-05	4.151128859
ud3000834	3	14962696	G	T	0.441339154	0.10768396	7.06E-05	4.151128859
id1008972	1	13555485	G	A	56.0826897	17.4547432	7.09E-05	4.149284779
id6002230	6	2876987	G	C	-1.95740623	0.477547333	7.13E-05	4.147073353
id1018646	1	30894634	G	A	0.462621379	0.11303259	7.22E-05	4.141685125
id9002735	9	9640008	G	A	-0.885668423	0.216428953	7.23E-05	4.140643244
id4010231	4	30189848	G	A	0.437673282	0.105043296	7.40E-05	4.130842914
id8007520	8	27424246	C	T	-1.130263794	0.272157706	7.77E-05	4.109337914
id4010227	4	30188229	C	T	-0.548655131	0.132166844	7.82E-05	4.106590666
id1014260	1	24242277	A	T	0.332506198	0.081703166	7.89E-05	4.103140202
id9002846	9	10344006	A	T	0.802018944	0.197226589	7.98E-05	4.097817948
id5008175	5	19868090	A	G	-3.29514959	0.793097613	8.02E-05	4.095854631
id1016919	1	28726858	A	G	-0.659170526	0.159336618	8.24E-05	4.084127625
id1021743	1	34744079	G	A	0.626115934	0.15436444	8.31E-05	4.080551547
id9002755	9	9783970	C	A	0.289012351	0.07126766	8.33E-05	4.079256266
id7001476	7	8137803	A	G	2.825574911	0.697665182	8.47E-05	4.071998706
id7001482	7	8165650	T	C	2.825574911	0.697665182	8.47E-05	4.071998706
ud7000659	7	8527552	C	T	2.825574911	0.697665182	8.47E-05	4.071998706
id2011727	2	26574431	T	C	0.468937393	0.115793666	8.51E-05	4.070066252
id10007177	10	22612177	C	G	0.937592988	0.227353983	8.64E-05	4.063639793
ud7001914	7	26037194	G	A	-0.770891568	0.187177437	8.81E-05	4.055118531
id7000727	7	5073227	T	A	-1.186401311	0.288146782	8.84E-05	4.053303979
id4011130	4	32088424	A	G	0.340578302	0.08436117	8.90E-05	4.050622866
id1018710	1	31005664	C	A	0.905207708	0.220036377	8.96E-05	4.04789154
dd11000488	11	21962286	G	A	-0.409367884	0.101462705	9.01E-05	4.045062127
id9007204	9	20941906	G	T	15.63091065	3.879575638	9.21E-05	4.035745425
ud7001914	7	26037194	G	A	-0.598720346	0.148737446	9.34E-05	4.029676409
id12005213	12	14487883	C	G	5.217316738	1.297116213	9.42E-05	4.025986375
id1022375	1	35512611	G	A	1.127301407	0.28038981	9.51E-05	4.021638791
id2016129	2	35251534	G	C	0.464454789	0.113431426	9.60E-05	4.017642591
id1000027	1	172923	C	T	0.291428719	0.072564167	9.67E-05	4.01450429
id2016106	2	35243408	T	A	0.435880789	0.106666928	9.89E-05	4.004802616
