# Supplementary material for: Analysis of Genetic Variation of Rice Straw Characteristics and Its Influence on Biomass
Source: Plant Direct. 2026 Jan 6;10(1):e70134. doi: 10.1002/pld3.70134 (PMC12771682; doi:10.1002/pld3.70134)
Supplement: Supplementary file 7 — Table S5: Examining the significance threshold with the FDR. [file PLD3-10-e70134-s008.pdf]

Table S5. Examining the significance threshold with the FDR.

Traits	SNP	Chromosor	Position	REF	ALT	Effect	SE	FarmCPU	LOP_PVALUE	FDR100%
Internode 4 dry weight	id10007177	10	22612177	C	G	198.5625	24.63215	1.65E-14	1.38E+01	2.92E-05
Internode 4 dry weight	id1027609	1	42358018	G	A	120.9079	19.37151	1.59E-10	9.80E+00	5.84E-05
Internode 4 dry weight	id3005558	3	10628270	A	G	111.8524	20.43797	1.34E-09	8.87E+00	8.76E-05
Internode 4 dry weight	id4006198	4	20233912	T	A	-56.5094	10.52448	6.15E-09	8.21E+00	1.17E-04
Internode 3 thickness	id2009738	2	23775529	G	A	-70.0297	14.33033	6.83E-08	7.17E+00	2.92E-05
Shoot dry weight	id2014525	2	32579679	C	T	-541.01	102.6583	5.36E-07	6.27E+00	2.92E-05
Shoot dry weight	id2014530	2	32581631	C	A	-541.01	102.6583	5.36E-07	6.27E+00	5.84E-05
Shoot dry weight	id2014550	2	32604725	G	C	-541.01	102.6583	5.36E-07	6.27E+00	8.76E-05
Biomass weight	id2014606	2	32825028	C	T	-228.737	55.09959	7.02E-07	6.15E+00	2.92E-05
Shoot dry weight	id9003485	9	12664532	G	A	381.738	73.30095	7.09E-07	6.15E+00	1.17E-04
Shoot dry weight	id5009418	5	22082907	C	A	-671.085	130.3667	9.27E-07	6.03E+00	1.46E-04
Internode 3 thickness	dd1002393	1	42608865	T	C	82.06542	18.35905	1.19E-06	5.92E+00	5.84E-05
Transverse diameter of internode 3	id4011518	4	32902367	A	G	0.450159	0.089951	1.68E-06	5.78E+00	2.92E-05
Shoot dry weight	id1007155	1	9633974	G	T	426.0653	85.95866	2.14E-06	5.67E+00	1.75E-04
Shoot dry weight	id1007156	1	9634128	A	T	426.0653	85.95866	2.14E-06	5.67E+00	2.04E-04
The average diameter of internode 4	id2016156	2	35258870	T	C	0.541455	0.106912	2.35E-06	5.63E+00	2.92E-05
Shoot dry weight	dd1001700	1	42340167	T	C	375.3927	76.52047	2.67E-06	5.57E+00	2.34E-04
Node 2 dry weight	id1000027	1	172923	C	T	5.468448	1.117435	2.70E-06	5.57E+00	2.92E-05
Internode 4 dry weight	id1002770	1	3397754	T	A	-55.9076	14.39957	2.90E-06	5.54E+00	1.46E-04
Internode 3 dry weight	id1001247	1	1511711	T	G	-147.943	30.32908	2.91E-06	5.54E+00	2.92E-05
Cross-section area of internode 3	id11000392	11	1484113	A	G	-1.16139	0.239291	3.24E-06	5.49E+00	2.92E-05
Shoot dry weight	id12006815	12	20913496	T	G	446.679	92.41998	3.65E-06	5.44E+00	2.63E-04
Shoot dry weight	dd1001737	1	42355255	A	G	348.2542	72.30716	3.92E-06	5.41E+00	2.92E-04
Shoot dry weight	id4004869	4	16929510	G	A	439.6548	91.34492	3.97E-06	5.40E+00	3.21E-04
Cross-section area of internode 3	id11000413	11	1512711	G	A	-1.18573	0.246911	4.03E-06	5.39E+00	5.84E-05
Longitudinal diameter of internode 3	id3007392	3	14788478	G	A	-0.51385	0.107169	4.16E-06	5.38E+00	2.92E-05
Longitudinal diameter of internode 3	id3007405	3	14839134	T	C	-0.51385	0.107169	4.16E-06	5.38E+00	5.84E-05
Biomass weight	id1025227	1	39719133	G	A	-253.814	61.42447	4.21E-06	5.38E+00	5.84E-05
Transverse diameter of internode 4	ud7001914	7	26037194	G	A	-0.83422	0.169624	4.22E-06	5.37E+00	2.92E-05
Shoot dry weight	id12005213	12	14487883	C	G	244.9722	51.23975	4.56E-06	5.34E+00	3.51E-04
The average diameter of internode 4	ud7001914	7	26037194	G	A	-0.80335	0.16406	4.59E-06	5.34E+00	5.84E-05
Internode 4 dry weight	id9000693	9	2748188	C	T	-43.6864	11.31147	4.86E-06	5.31E+00	1.75E-04
Shoot dry weight	id12005215	12	14488566	T	C	228.8632	48.17612	5.18E-06	5.29E+00	3.80E-04
The average diameter of internode 3	id3007392	3	14788478	G	A	-0.45744	0.096863	5.66E-06	5.25E+00	2.92E-05
The average diameter of internode 3	id3007405	3	14839134	T	C	-0.45744	0.096863	5.66E-06	5.25E+00	5.84E-05
Biomass weight	id8004716	8	17729217	G	A	-229.851	57.39951	5.74E-06	5.24E+00	8.76E-05
Internode 1 length	id7001323	7	7660553	T	C	2.427658	0.515047	5.85E-06	5.23E+00	2.92E-05
Transverse diameter of internode 3	id4011523	4	32928401	G	C	0.457714	0.097178	5.97E-06	5.22E+00	5.84E-05
Transverse diameter of internode 3	ud4002236	4	32903133	C	T	0.439741	0.093687	6.39E-06	5.19E+00	8.76E-05
The average diameter of internode 3	wd12002512	12	14659244	G	A	0.383261	0.081768	6.57E-06	5.18E+00	8.76E-05
Internode 1 length	id5011128	5	24169108	C	A	-2.81809	0.601668	6.63E-06	5.18E+00	5.84E-05
The average diameter of internode 3	ud7001914	7	26037194	G	A	-0.6425	0.138051	7.55E-06	5.12E+00	1.17E-04
Biomass weight	id11002182	11	5357024	G	A	-211.453	58.94324	7.56E-06	5.12E+00	1.17E-04
Shoot dry weight	id6015588	6	27433303	T	C	414.867	89.42089	8.26E-06	5.08E+00	4.09E-04
Transverse diameter of internode 4	id2016156	2	35258870	T	C	0.532007	0.112278	8.56E-06	5.07E+00	5.84E-05
Shoot dry weight	id9007879	9	22755878	T	C	-777.103	168.1607	8.91E-06	5.05E+00	4.38E-04
Node 1 diameter	id8004106	8	15314965	G	A	0.136317	0.029552	8.92E-06	5.05E+00	2.92E-05
Shoot dry weight	id1001247	1	1511711	T	G	-396.418	85.97121	9.30E-06	5.03E+00	4.67E-04
Shoot dry weight	id11001392	11	3661173	G	A	436.5249	94.72598	9.40E-06	5.03E+00	4.97E-04
Transverse diameter of internode 3	id4011513	4	32897775	A	G	0.423594	0.092102	9.50E-06	5.02E+00	1.17E-04
The average diameter of internode 3	id12005213	12	14487883	C	G	0.419153	0.091446	1.01E-05	4.99E+00	1.46E-04
Transverse diameter of internode 3	id1018646	1	30894634	G	A	0.539512	0.118125	1.08E-05	4.96E+00	1.46E-04
Internode 3 thickness	id5004367	5	8532207	T	C	-49.832	14.14064	1.18E-05	4.93E+00	8.76E-05
Longitudinal diameter of internode 3	ud3000828	3	14790825	G	A	-0.48537	0.107194	1.28E-05	4.89E+00	8.76E-05
Cross-section area of internode 3	id4010231	4	30189848	G	A	0.679812	0.150608	1.35E-05	4.87E+00	8.76E-05
Longitudinal diameter of internode 3	id11000293	11	1307788	G	C	-0.60394	0.134131	1.42E-05	4.85E+00	1.17E-04
Internode 3 dry weight	wd4002500	4	17002657	A	G	116.4698	25.89656	1.45E-05	4.84E+00	5.84E-05
Internode 3 dry weight	id4004892	4	17011401	T	C	116.4698	25.89656	1.45E-05	4.84E+00	8.76E-05
Internode 3 dry weight	id4004901	4	17013663	A	G	116.4698	25.89656	1.45E-05	4.84E+00	1.17E-04
Internode 3 dry weight	id4004929	4	17047786	T	C	116.4698	25.89656	1.45E-05	4.84E+00	1.46E-04
Cross-section area of internode 3	id2003149	2	6015346	G	A	-1.13396	0.252239	1.46E-05	4.84E+00	1.17E-04
Node 3 diameter	id11000275	11	1275872	A	T	-0.48841	0.109034	1.56E-05	4.81E+00	2.92E-05
Biomass weight	id3008667	3	17772284	C	A	-337.603	95.04323	1.64E-05	4.78E+00	1.46E-04
Longitudinal diameter of internode 3	ud7001914	7	26037194	G	A	-0.6863	0.153928	1.69E-05	4.77E+00	1.46E-04
Transverse diameter of internode 3	id11008239	11	21612962	C	T	-0.40375	0.090645	1.73E-05	4.76E+00	1.75E-04
The average diameter of internode 3	ud3000828	3	14790825	G	A	-0.43076	0.096906	1.79E-05	4.75E+00	1.75E-04
Longitudinal diameter of internode 3	id1000027	1	172923	C	T	0.40108	0.090399	1.85E-05	4.73E+00	1.75E-04

README

Column	Description
Traits	measured parameters(statistics)
SNP	uniqe identifier for the SNP
Chromosor	chromosome number where the SNP is located
Position	genomic position of the SNP
REF	base on the reference genome
Effect	estimated effect size from GWAS analysis
ALT	alternative base considered as SNP
SE	standard error of the effect estimate
FarmCPU	P-value from the FarmCPU GWAS model
PVALUE	statistical significance
FDR	false discovery rate in genetic association studies for multiple comparison adjustment

Node 3 dry weight	id1000027	1	172923	C	T	10.30184	2.32225	1.86E-05	4.73E+00	2.92E-05
Longitudinal diameter of internode 4	id2016156	2	35258870	T	C	0.547832	0.12079	1.87E-05	4.73E+00	2.92E-05
The average diameter of internode 3	ud12000862	12	14693039	C	A	0.382814	0.086366	1.88E-05	4.72E+00	2.04E-04
The average diameter of internode 3	id11008239	11	21612962	C	T	-0.37969	0.085661	1.88E-05	4.72E+00	2.34E-04
Panicle length	wd12000455	12	5552455	G	T	-1.73151	0.391578	2.00E-05	4.70E+00	2.92E-05
Node 1 diameter	id12005213	12	14487883	C	G	0.148163	0.033585	2.04E-05	4.69E+00	5.84E-05
Longitudinal diameter of internode 3	id11000275	11	1275872	A	T	-0.60533	0.137715	2.19E-05	4.66E+00	2.04E-04
Longitudinal diameter of internode 3	id1024348	1	38363629	C	T	0.41458	0.094497	2.26E-05	4.65E+00	2.34E-04
Node 1 diameter	id11008193	11	21542705	T	C	0.150699	0.03437	2.28E-05	4.64E+00	8.76E-05
Cross-section area of internode 3	id4010220	4	30145846	A	G	-0.79555	0.181526	2.31E-05	4.64E+00	1.46E-04
Cross-section area of internode 3	id4010225	4	30181026	C	T	-0.79555	0.181526	2.31E-05	4.64E+00	1.75E-04
Internode 2 diameter	id11000275	11	1275872	A	T	-0.45768	0.104586	2.36E-05	4.63E+00	2.92E-05
Internode 1 length	ud7000591	7	7718166	A	G	2.858699	0.655398	2.50E-05	4.60E+00	8.76E-05
Internode 1 length	id7001407	7	7841438	G	T	2.858699	0.655398	2.50E-05	4.60E+00	1.17E-04
Biomass weight	id4010433	4	30735857	A	G	172.477	46.92769	2.60E-05	4.59E+00	1.75E-04
Transverse diameter of internode 3	wd10002398	10	10659686	T	C	0.555439	0.127641	2.61E-05	4.58E+00	2.04E-04
Transverse diameter of internode 3	wd12002512	12	14659244	G	A	0.379071	0.087454	2.80E-05	4.55E+00	2.34E-04
The average diameter of internode 3	ud4002236	4	32903133	C	T	0.38731	0.089396	2.82E-05	4.55E+00	2.63E-04
Internode 2 dry weight	id1000027	1	172923	C	T	49.87926	11.52448	2.86E-05	4.54E+00	2.92E-05
Internode 3 thickness	id3006551	3	12696199	T	C	40.96554	11.90458	3.07E-05	4.51E+00	1.17E-04
Cross-section area of internode 3	id11000390	11	1465790	C	T	-1.03367	0.239867	3.09E-05	4.51E+00	2.04E-04
Internode 4 length	id7004429	7	24312823	A	C	3.242385	0.734904	3.13E-05	4.50E+00	2.92E-05
Internode 4 length	id7004434	7	24316717	C	T	3.242385	0.734904	3.13E-05	4.50E+00	5.84E-05
Transverse diameter of internode 4	id7004968	7	26071456	C	T	-0.85161	0.193572	3.13E-05	4.50E+00	8.76E-05
Internode 2 length	id5008100	5	19715820	C	G	2.244918	0.521569	3.14E-05	4.50E+00	2.92E-05
Internode 2 length	id5008122	5	19736445	G	A	2.244918	0.521569	3.14E-05	4.50E+00	5.84E-05
Transverse diameter of internode 3	id11001839	11	4628799	T	A	0.544861	0.126798	3.25E-05	4.49E+00	2.63E-04
Internode 3 thickness	id1013814	1	23790449	A	G	-39.1033	10.546	3.29E-05	4.48E+00	1.46E-04
Internode 3 dry weight	ud7001914	7	26037194	G	A	-109.875	25.61319	3.34E-05	4.48E+00	1.75E-04
Node 4 diameter	id2016104	2	35241812	C	A	0.435378	0.099364	3.35E-05	4.47E+00	2.92E-05
Transverse diameter of internode 3	ud12000862	12	14693039	C	A	0.391552	0.091882	3.74E-05	4.43E+00	2.92E-04
Node 2 diameter	id11000275	11	1275872	A	T	-0.34038	0.079905	3.75E-05	4.43E+00	2.92E-05
Biomass weight	id2011183	2	25630715	C	A	-259.782	75.24735	3.85E-05	4.42E+00	2.04E-04
Internode 1 length	id7001393	7	7837751	T	C	2.906815	0.683451	3.85E-05	4.41E+00	1.46E-04
Node 1 diameter	id12005326	12	14954178	G	A	0.188468	0.044372	3.94E-05	4.40E+00	1.17E-04
Longitudinal diameter of internode 3	id12005213	12	14487883	C	G	0.434158	0.102371	4.05E-05	4.39E+00	2.63E-04
Transverse diameter of internode 3	id9002735	9	9640008	G	A	-0.50559	0.119227	4.06E-05	4.39E+00	3.21E-04
Transverse diameter of internode 3	id11008437	11	21976195	G	A	-0.41808	0.098691	4.13E-05	4.38E+00	3.51E-04
Biomass weight	id5014595	5	28995509	T	C	240.3344	70.86289	4.22E-05	4.37E+00	2.34E-04
The average diameter of internode 4	id8007520	8	27424246	C	T	-1.05701	0.24498	4.29E-05	4.37E+00	8.76E-05
Node 4 diameter	id2016129	2	35251534	G	C	0.430488	0.099886	4.37E-05	4.36E+00	5.84E-05
Longitudinal diameter of internode 3	wd12002512	12	14659244	G	A	0.387451	0.091872	4.45E-05	4.35E+00	2.92E-04
Internode 2 dry weight	id8004106	8	15314965	G	A	48.84097	11.58823	4.48E-05	4.35E+00	5.84E-05
Internode 4 length	id5010361	5	23248349	C	T	3.51311	0.817564	4.78E-05	4.32E+00	8.76E-05
Internode 4 length	id5010375	5	23252288	G	A	3.51311	0.817564	4.78E-05	4.32E+00	1.17E-04
The average diameter of internode 3	id4011518	4	32902367	A	G	0.365029	0.086955	4.80E-05	4.32E+00	2.92E-04
Cross-section area of internode 3	id7004968	7	26071456	C	T	-1.16681	0.278046	4.83E-05	4.32E+00	2.34E-04
Transverse diameter of internode 3	dd11000522	11	21978827	G	A	-0.37838	0.090183	4.84E-05	4.32E+00	3.80E-04
Cross-section area of internode 3	id11000286	11	1305119	C	T	-1.06839	0.254934	4.94E-05	4.31E+00	2.63E-04
Cross-section area of internode 3	id11008603	11	22106066	G	T	-0.65256	0.155827	5.00E-05	4.30E+00	2.92E-04
Transverse diameter of internode 3	id11008620	11	22112337	T	C	0.368478	0.088013	5.02E-05	4.30E+00	4.09E-04
Transverse diameter of internode 3	id3007659	3	15214670	C	T	0.761555	0.182071	5.09E-05	4.29E+00	4.38E-04
Node 4 diameter	id2016156	2	35258870	T	C	0.418942	0.098377	5.28E-05	4.28E+00	8.76E-05
Cross-section area of internode 3	id9002643	9	9228486	T	C	0.712379	0.171049	5.46E-05	4.26E+00	3.21E-04
The average diameter of internode 3	id11000293	11	1307788	G	C	-0.50827	0.122116	5.52E-05	4.26E+00	3.21E-04
The average diameter of internode 3	id4011523	4	32928401	G	C	0.387791	0.093207	5.55E-05	4.26E+00	3.51E-04
Plant height	id1020630	1	33172139	C	T	-10.7021	2.568805	5.61E-05	4.25E+00	2.92E-05
Internode 3 thickness	id11003684	11	9765954	A	G	-65.5399	19.23508	5.68E-05	4.25E+00	1.75E-04
Internode 1 dry weight	id12005213	12	14487883	C	G	33.19561	8.004749	5.83E-05	4.23E+00	2.92E-05
Internode 2 diameter	wd12002512	12	14659244	G	A	0.289286	0.069787	5.87E-05	4.23E+00	5.84E-05
Internode 2 dry weight	id5009418	5	22082907	C	A	-139.907	33.86959	6.21E-05	4.21E+00	8.76E-05
Internode 2 length	id5008060	5	19664904	T	A	2.133185	0.517018	6.33E-05	4.20E+00	8.76E-05
The average diameter of internode 3	id4011513	4	32897775	A	G	0.363684	0.088134	6.34E-05	4.20E+00	3.80E-04
The average diameter of internode 4	id2016152	2	35255967	C	T	-0.61158	0.145418	6.42E-05	4.19E+00	1.17E-04
Transverse diameter of internode 3	id12005213	12	14487883	C	G	0.404144	0.098078	6.48E-05	4.19E+00	4.67E-04
Internode 1 length	id1002058	1	2605844	C	T	1.987071	0.482667	6.55E-05	4.18E+00	1.75E-04
Node 3 diameter	id11000272	11	1273238	C	A	-0.49696	0.120695	6.56E-05	4.18E+00	5.84E-05
Node 2 diameter	id1000027	1	172923	C	T	0.215697	0.052403	6.57E-05	4.18E+00	5.84E-05
Longitudinal diameter of internode 4	id7000727	7	5073227	T	A	-1.33046	0.316956	6.61E-05	4.18E+00	5.84E-05

Longitudinal diameter of internode 4	id8003991	8	14876879	A	G	0.638134	0.152487	6.92E-05	4.16E+00	8.76E-05
Transverse diameter of internode 3	id7003855	7	22593381	C	T	-0.43313	0.105555	6.93E-05	4.16E+00	4.97E-04
Cross-section area of internode 3	id11000293	11	1307788	G	C	-0.96067	0.234156	6.95E-05	4.16E+00	3.51E-04
Internode 4 length	id1000529	1	652340	A	G	2.531382	0.604123	7.06E-05	4.15E+00	1.46E-04
Longitudinal diameter of internode 3	id3007489	3	14930488	G	T	0.441339	0.107684	7.06E-05	4.15E+00	3.21E-04
Longitudinal diameter of internode 3	id3007509	3	14934867	T	A	0.441339	0.107684	7.06E-05	4.15E+00	3.51E-04
Longitudinal diameter of internode 3	ud3000834	3	14962696	G	T	0.441339	0.107684	7.06E-05	4.15E+00	3.80E-04
Internode 3 thickness	id1008972	1	13555485	G	A	56.08269	17.45474	7.09E-05	4.15E+00	2.04E-04
Panicle length	id6002230	6	2876987	G	C	-1.95741	0.477547	7.13E-05	4.15E+00	5.84E-05
The average diameter of internode 3	id1018646	1	30894634	G	A	0.462621	0.113033	7.22E-05	4.14E+00	4.09E-04
Cross-section area of internode 3	id9002735	9	9640008	G	A	-0.88567	0.216429	7.23E-05	4.14E+00	3.80E-04
The average diameter of internode 4	id4010231	4	30189848	G	A	0.437673	0.105043	7.40E-05	4.13E+00	1.46E-04
Longitudinal diameter of internode 4	id8007520	8	27424246	C	T	-1.13026	0.272158	7.77E-05	4.11E+00	1.17E-04
Longitudinal diameter of internode 4	id4010227	4	30188229	C	T	-0.54866	0.132167	7.82E-05	4.11E+00	1.46E-04
Transverse diameter of internode 3	id1014260	1	24242277	A	T	0.332506	0.081703	7.89E-05	4.10E+00	5.26E-04
Cross-section area of internode 3	id9002846	9	10344006	A	T	0.802019	0.197227	7.98E-05	4.10E+00	4.09E-04
Internode 4 length	id5008175	5	19868090	A	G	-3.29515	0.793098	8.02E-05	4.10E+00	1.75E-04
Node 4 diameter	id1016919	1	28726858	A	G	-0.65917	0.159337	8.24E-05	4.08E+00	1.17E-04
Cross-section area of internode 3	id1021743	1	34744079	G	A	0.626116	0.154364	8.31E-05	4.08E+00	4.38E-04
Transverse diameter of internode 3	id9002755	9	9783970	C	A	0.289012	0.071268	8.33E-05	4.08E+00	5.55E-04
Internode 1 length	id7001476	7	8137803	A	G	2.825575	0.697665	8.47E-05	4.07E+00	2.04E-04
Internode 1 length	id7001482	7	8165650	T	C	2.825575	0.697665	8.47E-05	4.07E+00	2.34E-04
Internode 1 length	ud7000659	7	8527552	C	T	2.825575	0.697665	8.47E-05	4.07E+00	2.63E-04
Longitudinal diameter of internode 3	id2011727	2	26574431	T	C	0.468937	0.115794	8.51E-05	4.07E+00	4.09E-04
Transverse diameter of internode 4	id10007177	10	22612177	C	G	0.937593	0.227354	8.64E-05	4.06E+00	1.17E-04
Longitudinal diameter of internode 4	ud7001914	7	26037194	G	A	-0.77089	0.187177	8.81E-05	4.06E+00	1.75E-04
The average diameter of internode 4	id7000727	7	5073227	T	A	-1.1864	0.288147	8.84E-05	4.05E+00	1.75E-04
Internode 2 diameter	id4011130	4	32088424	A	G	0.340578	0.084361	8.90E-05	4.05E+00	8.76E-05
The average diameter of internode 4	id1018710	1	31005664	C	A	0.905208	0.220036	8.96E-05	4.05E+00	2.04E-04
Transverse diameter of internode 3	dd11000488	11	21962286	G	A	-0.40937	0.101463	9.01E-05	4.05E+00	5.84E-04
Node 3 dry weight	id9007204	9	20941906	G	T	15.63091	3.879576	9.21E-05	4.04E+00	5.84E-05
Transverse diameter of internode 3	ud7001914	7	26037194	G	A	-0.59872	0.148737	9.34E-05	4.03E+00	6.13E-04
Node 2 dry weight	id12005213	12	14487883	C	G	5.217317	1.297116	9.42E-05	4.03E+00	5.84E-05
Cross-section area of internode 3	id1022375	1	35512611	G	A	1.127301	0.28039	9.51E-05	4.02E+00	4.67E-04
The average diameter of internode 4	id2016129	2	35251534	G	C	0.464455	0.113431	9.60E-05	4.02E+00	2.34E-04
Node 3 diameter	id1000027	1	172923	C	T	0.291429	0.072564	9.67E-05	4.01E+00	8.76E-05
Node 4 diameter	id2016106	2	35243408	T	A	0.435881	0.106667	9.89E-05	4.00E+00	1.46E-04
