# Supplementary material for: Analysis of Genetic Variation of Rice Straw Characteristics and Its Influence on Biomass
Source: Plant Direct. 2026 Jan 6;10(1):e70134. doi: 10.1002/pld3.70134 (PMC12771682; doi:10.1002/pld3.70134)
Supplement: Supplementary file 8 — Table S6: Characteristics of the SNPs in the exonic/intronic regions of the identified genes. [file PLD3-10-e70134-s012.pdf]

Table S6. Characteristics of the SNPs in the exonic/intronic regions of the identified genes

Chromosome	start	end	Strand	ID	Position	exon/intron	Discription
1	170798	173144	-	Os01g0103000	172923	intron	ID=Os01g0103000;Name=Os01g0103000;Note=Snf7 family protein.
1	172587	175073	+	Os01g0103050	172923	Exon1	ID=Os01g0103050;Name=Os01g0103050;Note=Non-protein coding transcript.
1	170798	173144	-	Os01g0103000	172923	intron	ID=Os01g0103000;Name=Os01g0103000;Note=Snf7 family protein.
1	172587	175073	+	Os01g0103050	172923	Exon1	ID=Os01g0103050;Name=Os01g0103050;Note=Non-protein coding transcript.
1	170798	173144	-	Os01g0103000	172923	intron	ID=Os01g0103000;Name=Os01g0103000;Note=Snf7 family protein.
1	172587	175073	+	Os01g0103050	172923	Exon1	ID=Os01g0103050;Name=Os01g0103050;Note=Non-protein coding transcript.
1	170798	173144	-	Os01g0103000	172923	intron	ID=Os01g0103000;Name=Os01g0103000;Note=Snf7 family protein.
1	172587	175073	+	Os01g0103050	172923	Exon1	ID=Os01g0103050;Name=Os01g0103050;Note=Non-protein coding transcript.
1	170798	173144	-	Os01g0103000	172923	intron	ID=Os01g0103000;Name=Os01g0103000;Note=Snf7 family protein.
1	172587	175073	+	Os01g0103050	172923	Exon1	ID=Os01g0103050;Name=Os01g0103050;Note=Non-protein coding transcript.
1	170798	173144	-	Os01g0103000	172923	intron	ID=Os01g0103000;Name=Os01g0103000;Note=Snf7 family protein.
1	172587	175073	+	Os01g0103050	172923	Exon1	ID=Os01g0103050;Name=Os01g0103050;Note=Non-protein coding transcript.
1	2605614	2612731	+	Os01g0148050	2605844	Exon1	ID=Os01g0148050;Name=Os01g0148050;Note=Hypothetical conserved gene.
1	3397441	3400487	+	Os01g0166100	3397754	Exon2	ID=Os01g0166100;Name=Os01g0166100;Note=Similar to Ca(2+)-dependent nuclease.
1	9631334	9638881	-	Os01g0275600	9633974	Exon16	ID=Os01g0275600;Name=Os01g0275600;Note=Similar to Argonaute 4 protein.
1	9631334	9638881	-	Os01g0275600	9634128	intron	ID=Os01g0275600;Name=Os01g0275600;Note=Similar to Argonaute 4 protein.
1	13550372	13556984	+	Os01g0343100	13555485	Exon2	ID=Os01g0343100;Name=Os01g0343100;Note=Protein of unknown function DUF594 family protein.
1	24240257	24245230	-	Os01g0611900	24242277	intron	ID=Os01g0611900;Name=Os01g0611900;Note=Pentatricopeptide repeat domain containing protein.
1	28726589	28727227	-	Os01g0694800	28726858	Exon1	ID=Os01g0694800;Name=Os01g0694800;Note=Conserved hypothetical protein.
1	33168429	33172680	-	Os01g0782800	33172139	Exon1	ID=Os01g0782800;Name=Os01g0782800;Note=Similar to Cyclic nucleotide-gated ion channel 4 (AtCNGC4) (Cyclic nucleotide-and calmodulin-regulated ion channel 4) (AtHLM1).
1	34738321	34746452	-	Os01g0816550	34744079	intron	ID=Os01g0816550;Name=Os01g0816550;Note=Non-protein coding transcript.
1	35508869	35513088	+	Os01g0830100	35512611	Exon7	ID=Os01g0830100;Name=Os01g0830100;Note=Pyridine nucleotide-disulphide oxidoreductase NAD-binding region domain containing protein.
1	42336297	42348672	-	Os01g0960400	42340167	Exon23	ID=Os01g0960400;Name=Os01g0960400;Note=Protein kinase core domain containing protein.
1	42350945	42355276	-	Os01g0960500	42355255	Exon1	ID=Os01g0960500;Name=Os01g0960500;Note=Zinc finger RING/FYVE/PHD-type domain containing protein.
2	6014063	6016083	+	Os02g0207300	6015346	intron	ID=Os02g0207300;Name=Os02g0207300;Note=Non-protein coding transcript.
2	23773136	23775628	+	Os02g0606800	23775529	Exon5	ID=Os02g0606800;Name=Os02g0606800;Note=Isochorismatase hydrolase family protein.
2	25626748	25636053	+	Os02g0639000	25630715	intron	ID=Os02g0639000;Name=Os02g0639000;Note=Double-stranded RNA binding domain containing protein.
2	26573548	26576645	-	Os02g0657700	26574431	Exon3	ID=Os02g0657700;Name=Os02g0657700;Note=Uncharacterised conserved protein UCP012943 domain containing protein.
2	32575339	32580717	-	Os02g0771800	32579679	intron	ID=Os02g0771800;Name=Os02g0771800;Note=Similar to predicted protein.
2	32602141	32611044	+	Os02g0772500	32604725	Exon4	ID=Os02g0772500;Name=Os02g0772500;Note=Protein of unknown function DUF1740 domain containing protein.
2	35242169	35243851	+	Os02g0820800	35243408	intron	ID=Os02g0820800;Name=Os02g0820800;Note=Predicted AT-hook DNA-binding domain containing protein.
2	35255363	35256043	+	Os02g0821100	35255967	Exon1	ID=Os02g0821100;Name=Os02g0821100;Note=Non-protein coding transcript.
3	12695305	12706384	+	Os03g0340900	12696199	intron	ID=Os03g0340900;Name=Os03g0340900;Note=Similar to RSH1.
3	14788216	14789968	-	Os03g0375200	14788478	Exon1	ID=Os03g0375200;Name=Os03g0375200;Note=Hypothetical conserved gene.
3	14788216	14789968	-	Os03g0375200	14788478	Exon1	ID=Os03g0375200;Name=Os03g0375200;Note=Hypothetical conserved gene.
3	14790176	14798032	-	Os03g0375300	14790825	Exon6	ID=Os03g0375300;Name=Os03g0375300;Note=Similar to cationic amino acid transporter.
3	14790303	14791119	+	Os03g0375601	14790825	Exon1	ID=Os03g0375601;Name=Os03g0375601;Note=Hypothetical gene.
3	14790176	14798032	-	Os03g0375300	14790825	Exon6	ID=Os03g0375300;Name=Os03g0375300;Note=Similar to cationic amino acid transporter.
3	14790303	14791119	+	Os03g0375601	14790825	Exon1	ID=Os03g0375601;Name=Os03g0375601;Note=Hypothetical gene.
3	15214197	15219070	-	Os03g0383800	15214670	Exon13	ID=Os03g0383800;Name=Os03g0383800;Note=Similar to SAP domain containing protein expressed.
4	16916761	16933860	+	Os04g0354400	16929510	intron	ID=Os04g0354400;Name=Os04g0354400;Note=Similar to OSIGBa0092G14.8 protein.
4	16986167	17002986	+	Os04g0355500	17002657	intron	ID=Os04g0355500;Name=Os04g0355500;Note=Similar to OSIGBa0147J02.3 protein.
4	30145192	30146641	+	Os04g0597400	30145846	Exon2	ID=Os04g0597400;Name=Os04g0597400;Note=Similar to OSJNba0093F12.10 protein.
4	30180330	30181981	+	Os04g0598200	30181026	intron	ID=Os04g0598200;Name=Os04g0598200;Note=Similar to 60S ribosomal protein L12.
4	30734729	30739334	-	Os04g0607600	30735857	intron	ID=Os04g0607600;Name=Os04g0607600;Note=Cation transporter family protein.
5	8526412	8535211	-	Os05g0240200	8532207	Exon2	ID=Os05g0240200;Name=Os05g0240200;Note=Similar to NB-ARC domain containing protein expressed.
5	19718538	19737605	-	Os05g0405000	19736445	intron	ID=Os05g0405000;Name=Os05g0405000;Note=Orthophosphate dikinase precursor (EC 2.7.9.1).
5	22081376	22083466	-	Os05g0449900	22082907	Exon1	ID=Os05g0449900;Name=Os05g0449900;Note=Homeodomain-like containing protein.
5	22081376	22083466	-	Os05g0449900	22082907	Exon1	ID=Os05g0449900;Name=Os05g0449900;Note=Homeodomain-like containing protein.
5	23247706	23250054	+	Os05g0473300	23248349	intron	ID=Os05g0473300;Name=Os05g0473300;Note=Hypothetical conserved gene.
5	23251135	23254403	+	Os05g0473401	23252288	Exon5	ID=Os05g0473401;Name=Os05g0473401;Note=Hypothetical conserved gene.
5	28989788	28995655	+	Os05g0582400	28995509	Exon9	ID=Os05g0582400;Name=Os05g0582400;Note=Similar to Mitogen-activated protein kinase 9.
6	2874384	2877067	+	Os06g0155900	2876987	Exon4	ID=Os06g0155900;Name=Os06g0155900;Note=Conserved hypothetical protein.
7	5071071	5076929	-	Os07g0193600	5073227	intron	ID=Os07g0193600;Name=Os07g0193600;Note=Similar to Aminoacyl-tRNA synthetase.
7	5071071	5076929	-	Os07g0193600	5073227	intron	ID=Os07g0193600;Name=Os07g0193600;Note=Similar to Aminoacyl-tRNA synthetase.
7	7713453	7720559	+	Os07g0238700	7718166	intron	ID=Os07g0238700;Name=Os07g0238700;Note=Similar to Glutamate dehydrogenase (GDH).
7	8136965	8141055	+	Os07g0246200	8137803	intron	ID=Os07g0246200;Name=Os07g0246200;Note=Similar to Calreticulin (Fragment).
7	22590906	22594640	+	Os07g0564000	22593381	intron	ID=Os07g0564000;Name=Os07g0564000;Note=Conserved hypothetical protein.
7	24312094	24315383	-	Os07g0596900	24312823	intron	ID=Os07g0596900;Name=Os07g0596900;Note=Similar to SUSIBA2-like (WRKY transcription factor 80).
7	24315421	24317851	+	Os07g0596950	24316717	Exon1	ID=Os07g0596950;Name=Os07g0596950;Note=Conserved hypothetical protein.
7	26036373	26039287	+	Os07g0628301	26037194	intron	ID=Os07g0628301;Name=Os07g0628301;Note=Hypothetical gene. (Os07t0628301-00);Transcript variants=Os07t0628301-00
7	26036275	26039311	-	Os07g0628400	26037194	intron	ID=Os07g0628400;Name=Os07g0628400;Note=Similar to 40S ribosomal protein S9.
7	26036373	26039287	+	Os07g0628301	26037194	intron	ID=Os07g0628301;Name=Os07g0628301;Note=Hypothetical gene.
7	26036275	26039311	-	Os07g0628400	26037194	intron	ID=Os07g0628400;Name=Os07g0628400;Note=Similar to 40S ribosomal protein S9.
7	26036373	26039287	+	Os07g0628301	26037194	intron	ID=Os07g0628301;Name=Os07g0628301;Note=Hypothetical gene.
7	26036275	26039311	-	Os07g0628400	26037194	intron	ID=Os07g0628400;Name=Os07g0628400;Note=Similar to 40S ribosomal protein S9.
7	26036373	26039287	+	Os07g0628301	26037194	intron	ID=Os07g0628301;Name=Os07g0628301;Note=Hypothetical gene.
7	26036275	26039311	-	Os07g0628400	26037194	intron	ID=Os07g0628400;Name=Os07g0628400;Note=Similar to 40S ribosomal protein S9.
7	26036373	26039287	+	Os07g0628301	26037194	intron	ID=Os07g0628301;Name=Os07g0628301;Note=Hypothetical gene.

README

Column	Description
Chromosoı	chromosome number where the SNP is located
start	5′-end of the DNA strand
end	3' end of the DNA strand
Strand	DNA strand
ID	uniqe identifier for the gene
Position	genomic position of the SNP
exon/intro	the parts that contain protein information/the non-coding regions
Discription	ID& gene name

7	26036275	26039311	-	Os07g0628400	26037194	intron	ID=Os07g0628400;Name=Os07g0628400;Note=Similar to 40S ribosomal protein S9.
7	26036373	26039287	+	Os07g0628301	26037194	intron	ID=Os07g0628301;Name=Os07g0628301;Note=Hypothetical gene.
7	26036275	26039311	-	Os07g0628400	26037194	intron	ID=Os07g0628400;Name=Os07g0628400;Note=Similar to 40S ribosomal protein S9.
7	26036373	26039287	+	Os07g0628301	26037194	intron	ID=Os07g0628301;Name=Os07g0628301;Note=Hypothetical gene
7	26036275	26039311	-	Os07g0628400	26037194	intron	ID=Os07g0628400;Name=Os07g0628400;Note=Similar to 40S ribosomal protein S9.
9	12662824	12667523	-	Os09g0376900	12664532	intron	ID=Os09g0376900;Name=Os09g0376900;Note=Similar to Potassium transporter 13 (AtPOT13) (AtKT5).
9	22755848	22757503	+	Os09g0570100	22755878	intron	ID=Os09g0570100;Name=Os09g0570100;Note=Protein kinase catalytic domain domain containing protein.
11	1274493	1279077	-	Os11g0127700	1275872	intron	ID=Os11g0127700;Name=Os11g0127700;Note=Conserved hypothetical protein.
11	1274493	1279077	-	Os11g0127700	1275872	intron	ID=Os11g0127700;Name=Os11g0127700;Note=Conserved hypothetical protein.
11	1274493	1279077	-	Os11g0127700	1275872	intron	ID=Os11g0127700;Name=Os11g0127700;Note=Conserved hypothetical protein.
11	1274493	1279077	-	Os11g0127700	1275872	intron	ID=Os11g0127700;Name=Os11g0127700;Note=Conserved hypothetical protein.
11	1306316	1308673	+	Os11g0128400	1307788	exon1	ID=Os11g0128400;Name=Os11g0128400;Note=CDC45-like protein family protein.
11	1306316	1308673	+	Os11g0128400	1307788	exon1	ID=Os11g0128400;Name=Os11g0128400;Note=CDC45-like protein family protein.
11	1306316	1308673	+	Os11g0128400	1307788	exon1	ID=Os11g0128400;Name=Os11g0128400;Note=CDC45-like protein family protein.
11	1462162	1467581	+	Os11g0132000	1465790	intron	ID=Os11g0132000;Name=Os11g0132000;Note=Similar to Arabinoxylan arabinofuranohydrolase isoenzyme AXAH-II.
11	1484073	1491751	+	Os11g0132600	1484113	exon1	ID=Os11g0132600;Name=Os11g0132600;Note=Similar to Arabinoxylan arabinofuranohydrolase isoenzyme AXAH-II.
11	3656726	3661466	+	Os11g0173800	3661173	exon2	ID=Os11g0173800;Name=Os11g0173800;Note=Serine/threonine protein kinase-related domain containing protein.
11	4625100	4631163	+	Os11g0191400	4628799	intron	ID=Os11g0191400;Name=Os11g0191400;Note=ATP-NAD kinase%2C PpnK-type%2C all-beta domain containing protein.
11	5356008	5359272	+	Os11g0206150	5357024	intron	ID=Os11g0206150;Name=Os11g0206150;Note=Hypothetical protein.
12	14487420	14491026	-	Os12g0438600	14487883	intron	ID=Os12g0438600;Name=Os12g0438600;Note=Similar to chloride channel protein.
12	14487420	14491026	-	Os12g0438600	14487883	intron	ID=Os12g0438600;Name=Os12g0438600;Note=Similar to chloride channel protein.
12	14487420	14491026	-	Os12g0438600	14487883	intron	ID=Os12g0438600;Name=Os12g0438600;Note=Similar to chloride channel protein.
12	14487420	14491026	-	Os12g0438600	14487883	intron	ID=Os12g0438600;Name=Os12g0438600;Note=Similar to chloride channel protein.
12	14487420	14491026	-	Os12g0438600	14487883	intron	ID=Os12g0438600;Name=Os12g0438600;Note=Similar to chloride channel protein.
12	14487420	14491026	-	Os12g0438600	14487883	intron	ID=Os12g0438600;Name=Os12g0438600;Note=Similar to chloride channel protein.
12	14487420	14491026	-	Os12g0438600	14487883	intron	ID=Os12g0438600;Name=Os12g0438600;Note=Similar to chloride channel protein.
12	14487420	14491026	-	Os12g0438600	14488566	intron	ID=Os12g0438600;Name=Os12g0438600;Note=Similar to chloride channel protein.
