# Supplementary material for: Analysis of Genetic Variation of Rice Straw Characteristics and Its Influence on Biomass
Source: Plant Direct. 2026 Jan 6;10(1):e70134. doi: 10.1002/pld3.70134 (PMC12771682; doi:10.1002/pld3.70134)
Supplement: Supplementary file 9 — Table S7: The presence/absence of cis elements in the gene promoters. [file PLD3-10-e70134-s002.pdf]

Table S7. The presence/absence of *cis* elements in the gene promoters.

Chromosor	pos_snp_prom	start	end	Strand	LOC_number	cis element	Discription
1	1030	651870	653369	-	LOC_Os01g02200	GT1CONSENSUS-GT1GMSCAM4	Similar to Plakoglobin/armadillo/beta-catenin-like protein
1	1248	1510464	1511963	+	LOC_Os01g03660	none	
1	74	1511638	1513137	+	LOC_Os01g03670	none	
1	1248	1510464	1511963	+	LOC_Os01g03660	none	
1	74	1511638	1513137	+	LOC_Os01g03670	none	
1	1139	31004526	31006025	+	LOC_Os01g53920	none	
1	959	42607907	42609406	+	LOC_Os01g73530	none	
2	587	32580718	32582217	-	LOC_Os02g53210	CACTFTPPCA1	Similar to predicted protein
2	222	32823750	32825249	-	LOC_Os02g53660	none	
2	1144	35240669	35242168	+	LOC_Os02g57520	none	
2	1454	35250081	35251580	+	LOC_Os02g57530	1AT-EBOXBNNAPA-MYCCONSENSUSAT -CACTFTP	Similar to Ethylene receptor-like protein 2
2	1454	35250081	35251580	+	LOC_Os02g57530	1AT-EBOXBNNAPA-MYCCONSENSUSAT -CACTFTP	Similar to Ethylene receptor-like protein 2
3	644	14789969	14791468	-	LOC_Os03g25850	SORLIP1AT	Hypothetical conserved gene
3	644	14789969	14791468	-	LOC_Os03g25850	SORLIP1AT	Hypothetical conserved gene
3	492	14838126	14839625	-	LOC_Os03g25920	GCCCORE	Amino acid/polyamine transporter I family protein
3	1489	14839123	14840622	-	LOC_Os03g25920	GCCCORE	Similar to Amino acid permease family protein
3	492	14838126	14839625	-	LOC_Os03g25920	GCCCORE	Amino acid/polyamine transporter I family protein
3	1489	14839123	14840622	-	LOC_Os03g25920	GCCCORE	Similar to Amino acid permease family protein
3	1409	14930397	14931896	-	LOC_Os03g26044	URECOREATSULTR11-CCAATBOX1-CARGNCA	Cellulose synthase-like A5
4	464	17046750	17048249	-	None	CAATBOX1	Hypothetical protein
4	682	30145165	30146664	+	LOC_Os04g50930	none	
5	1106	19866985	19868484	+	LOC_Os05g33730	none	
5	1368	28995377	28996876	-	None	none	
5	1037	28994473	28995972	+	LOC_Os05g50570	GTGANTG10-ARR1AT-INRNTPSADB	Peptidase serine carboxypeptidase domain containing protein
6	1384	27431920	27433419	+	LOC_Os06g45380	none	
7	256	7840194	7841693	-	none	none	
7	816	8164966	8166465	-	LOC_Os07g14310	none	
7	167	24315384	24316883	-	LOC_Os07g40570	ISO1-WRKY71OS -WBOXNTERF3 -EECCRCAH1-CAC	Similar to SUSIBA2-like (WRKY transcription factor 80)
8	1299	14875581	14877080	+	None	none	
8	286	17728932	17730431	+	LOC_Os08g28970	none	
8	90	27422836	27424335	-	LOC_Os08g43390	AGL1ATCONSENSUS-AGL2ATCONSENSUS	Similar to Cytochrome P450 78A1
8	90	27422836	27424335	-	LOC_Os08g43390	AGL1ATCONSENSUS-AGL2ATCONSENSUS	Similar to Cytochrome P450 78A1
9	37	9639972	9641471	+	LOC_Os09g15780	none	
9	37	9639972	9641471	+	LOC_Os09g15780	none	
9	652	10343355	10344854	+	None	OXBNNAPA-MYCCONSENSUSAT-MYB2CONSENSUS	Non-protein coding transcript
9	913	20940994	20942493	+	LOC_Os09g36290	none	
11	304	1304816	1306315	+	LOC_Os11g03430	CACTFTPPCA1	CDC45-like protein family protein
11	870	5356394	5357893	-	None	MYBCORE	Conserved hypothetical protein

README

Column	Description
Chromosor	chromosome number where the SNP is located
pos_snp_p	SNP position in the promoter region
start	5′-end of the DNA strand
end	3' end of the DNA strand
Strand	DNA strand
LOC_numl	uniqe identifier for the gene
cis element	Cis Acting Element: essential elements present in DNA upstream of the transcriptional start site that control gene expression without the need for trans factors
Discription	gene name
