# Supplementary material for: Analysis of Genetic Variation of Rice Straw Characteristics and Its Influence on Biomass
Source: Plant Direct. 2026 Jan 6;10(1):e70134. doi: 10.1002/pld3.70134 (PMC12771682; doi:10.1002/pld3.70134)
Supplement: Supplementary file 10 — Table S8: SNPEff analysis results. [file PLD3-10-e70134-s007.pdf]

| README | |
|---|---|
| Column | Description |
| Chromosoı | chromosome number where the SNP is located |
| Position | genomic position of the SNP |
| ID_Traits | /Measured parameters(statistics) |
| REF | base on the reference genome |
| ALT | alternative base considered as SNP |

**Table S8.** SNPEff analysis results

| Chromosoı | Position | ID_Traits | REF | ALT |
|---|---|---|---|---|
| 1 | 172923 | id1000027_Node 2 diameter | C | T |
| 1 | 172923 | id1000027_Node 3 diameter | C | T |
| 1 | 172923 | id1000027_Longitudinal diameter of internode 3 | C | T |
| 1 | 172923 | id1000027_Internode 2 dry weight | C | T |
| 1 | 172923 | id1000027_Node 2 dry weight | C | T |
| 1 | 172923 | id1000027_Node 3 dry weight | C | T |
| 1 | 652340 | id1000529_Internode 4 length | A | G |
| 1 | 1511711 | id1001247_Shoot dry weight | T | G |
| 1 | 1511711 | id1001247_Internode 3 dry weight | T | G |
| 1 | 2605844 | id1002058_Internode 1 length | C | T |
| 1 | 3397754 | id1002770_Internode 4 dry weight | T | A |
| 1 | 9633974 | id1007155_Shoot dry weight | G | T |
| 1 | 9634128 | id1007156_Shoot dry weight | A | T |
| 1 | 13555485 | id1008972_Internode 3 thickness | G | A |
| 1 | 23790449 | id1013814_Internode 3 thickness | A | G |
| 1 | 24242277 | id1014260_Transverse diameter of internode 3 | A | T |
| 1 | 28726858 | id1016919_Node 4 diameter | A | G |
| 1 | 30894634 | id1018646_The average diameter of internode 3 | G | A |
| 1 | 30894634 | id1018646_Transverse diameter of internode 3 | G | A |
| 1 | 31005664 | id1018710_The average diameter of internode 4 | C | A |
| 1 | 33172139 | id1020630_Plant height | C | T |
| 1 | 34744079 | id1021743_Cross-section area of internode 3 | G | A |
| 1 | 35512611 | id1022375_Cross-section area of internode 3 | G | A |
| 1 | 38363629 | id1024348_Longitudinal diameter of internode 3 | C | T |
| 1 | 39719133 | id1025227_Biomass weight | G | A |
| 1 | 42340167 | dd1001700_Shoot dry weight | T | C |
| 1 | 42355255 | dd1001737_Shoot dry weight | A | G |
| 1 | 42358018 | id1027609_Internode 4 dry weight | G | A |
| 1 | 42608865 | dd1002393_Internode 3 thickness | T | C |
| 2 | 6015346 | id2003149_Cross-section area of internode 3 | G | A |
| 2 | 23775529 | id2009738_Internode 3 thickness | G | A |
| 2 | 25630715 | id2011183_Biomass weight | C | A |
| 2 | 26574431 | id2011727_Longitudinal diameter of internode 3 | T | C |
| 2 | 32579679 | id2014525_Shoot dry weight | C | T |
| 2 | 32581631 | id2014530_Shoot dry weight | C | A |
| 2 | 32604725 | id2014550_Shoot dry weight | G | C |
| 2 | 32825028 | id2014606_Biomass weight | C | T |
| 2 | 35241812 | id2016104_Node 4 diameter | C | A |
| 2 | 35243408 | id2016106_Node 4 diameter | T | A |
| 2 | 35251534 | id2016129_Node 4 diameter | G | C |
| 2 | 35251534 | id2016129_The average diameter of internode 4 | G | C |
| 2 | 35255967 | id2016152_The average diameter of internode 4 | C | T |
| 2 | 35258870 | id2016156_Node 4 diameter | T | C |
| 2 | 35258870 | id2016156_The average diameter of internode 4 | T | C |
| 2 | 35258870 | id2016156_Longitudinal diameter of internode 4 | T | C |
| 2 | 35258870 | id2016156_Transverse diameter of internode 4 | T | C |

| | | | | |
|---|---|---|---|---|
| 3 | 10628270 | id3005558_Internode 4 dry weight | A | G |
| 3 | 12696199 | id3006551_Internode 3 thickness | T | C |
| 3 | 14788478 | id3007392_The average diameter of internode 3 | G | A |
| 3 | 14788478 | id3007392_Longitudinal diameter of internode 3 | G | A |
| 3 | 14790825 | ud3000828_The average diameter of internode 3 | G | A |
| 3 | 14790825 | ud3000828_Longitudinal diameter of internode 3 | G | A |
| 3 | 14839134 | id3007405_The average diameter of internode 3 | T | C |
| 3 | 14839134 | id3007405_Longitudinal diameter of internode 3 | T | C |
| 3 | 14930488 | id3007489_Longitudinal diameter of internode 3 | G | T |
| 3 | 14934867 | id3007509_Longitudinal diameter of internode 3 | T | A |
| 3 | 14962696 | ud3000834_Longitudinal diameter of internode 3 | G | T |
| 3 | 15214670 | id3007659_Transverse diameter of internode 3 | C | T |
| 3 | 17772284 | id3008667_Biomass weight | C | A |
| 4 | 16929510 | id4004869_Shoot dry weight | G | A |
| 4 | 17002657 | wd4002500_Internode 3 dry weight | A | G |
| 4 | 17011401 | id4004892_Internode 3 dry weight | T | C |
| 4 | 17013663 | id4004901_Internode 3 dry weight | A | G |
| 4 | 17047786 | id4004929_Internode 3 dry weight | T | C |
| 4 | 20233912 | id4006198_Internode 4 dry weight | T | A |
| 4 | 30145846 | id4010220_Cross-section area of internode 3 | A | G |
| 4 | 30181026 | id4010225_Cross-section area of internode 3 | C | T |
| 4 | 30188229 | id4010227_Longitudinal diameter of internode 4 | C | T |
| 4 | 30189848 | id4010231_The average diameter of internode 4 | G | A |
| 4 | 30189848 | id4010231_Cross-section area of internode 3 | G | A |
| 4 | 30735857 | id4010433_Biomass weight | A | G |
| 4 | 32088424 | id4011130_Internode 2 diameter | A | G |
| 4 | 32897775 | id4011513_The average diameter of internode 3 | A | G |
| 4 | 32897775 | id4011513_Transverse diameter of internode 3 | A | G |
| 4 | 32902367 | id4011518_The average diameter of internode 3 | A | G |
| 4 | 32902367 | id4011518_Transverse diameter of internode 3 | A | G |
| 4 | 32903133 | ud4002236_The average diameter of internode 3 | C | T |
| 4 | 32903133 | ud4002236_Transverse diameter of internode 3 | C | T |
| 4 | 32928401 | id4011523_The average diameter of internode 3 | G | C |
| 4 | 32928401 | id4011523_Transverse diameter of internode 3 | G | C |
| 5 | 8532207 | id5004367_Internode 3 thickness | T | C |
| 5 | 19664904 | id5008060_Internode 2 length | T | A |
| 5 | 19715820 | id5008100_Internode 2 length | C | G |
| 5 | 19736445 | id5008122_Internode 2 length | G | A |
| 5 | 19868090 | id5008175_Internode 4 length | A | G |
| 5 | 22082907 | id5009418_Shoot dry weight | C | A |
| 5 | 22082907 | id5009418_Internode 2 dry weight | C | A |
| 5 | 23248349 | id5010361_Internode 4 length | C | T |
| 5 | 23252288 | id5010375_Internode 4 length | G | A |
| 5 | 24169108 | id5011128_Internode 1 length | C | A |
| 5 | 28995509 | id5014595_Biomass weight | T | C |
| 6 | 2876987 | id6002230_Panicle length | G | C |
| 6 | 27433303 | id6015588_Shoot dry weight | T | C |
| 7 | 5073227 | id7000727_The average diameter of internode 4 | T | A |
| 7 | 5073227 | id7000727_Longitudinal diameter of internode 4 | T | A |
| 7 | 7660553 | id7001323_Internode 1 length | T | C |
| 7 | 7718166 | ud7000591_Internode 1 length | A | G |
| 7 | 7837751 | id7001393_Internode 1 length | T | C |
| 7 | 7841438 | id7001407_Internode 1 length | G | T |
| 7 | 8137803 | id7001476_Internode 1 length | A | G |
| 7 | 8165650 | id7001482_Internode 1 length | T | C |
| 7 | 8527552 | ud7000659_Internode 1 length | C | T |
| 7 | 22593381 | id7003855_Transverse diameter of internode 3 | C | T |
| 7 | 24312823 | id7004429_Internode 4 length | A | C |

| | | | | |
|---|---|---|---|---|
| 7 | 24316717 | id7004434_Internode 4 length | C | T |
| 7 | 26037194 | ud7001914_The average diameter of internode 3 | G | A |
| 7 | 26037194 | ud7001914_The average diameter of internode 4 | G | A |
| 7 | 26037194 | ud7001914_Longitudinal diameter of internode 3 | G | A |
| 7 | 26037194 | ud7001914_Transverse diameter of internode 3 | G | A |
| 7 | 26037194 | ud7001914_Longitudinal diameter of internode 4 | G | A |
| 7 | 26037194 | ud7001914_Transverse diameter of internode 4 | G | A |
| 7 | 26037194 | ud7001914_Internode 3 dry weight | G | A |
| 7 | 26071456 | id7004968_Transverse diameter of internode 4 | C | T |
| 7 | 26071456 | id7004968_Cross-section area of internode 3 | C | T |
| 8 | 14876879 | id8003991_Longitudinal diameter of internode 4 | A | G |
| 8 | 15314965 | id8004106_Node 1 diameter | G | A |
| 8 | 15314965 | id8004106_Internode 2 dry weight | G | A |
| 8 | 17729217 | id8004716_Biomass weight | G | A |
| 8 | 27424246 | id8007520_The average diameter of internode 4 | C | T |
| 8 | 27424246 | id8007520_Longitudinal diameter of internode 4 | C | T |
| 9 | 2748188 | id9000693_Internode 4 dry weight | C | T |
| 9 | 9228486 | id9002643_Cross-section area of internode 3 | T | C |
| 9 | 9640008 | id9002735_Transverse diameter of internode 3 | G | A |
| 9 | 9640008 | id9002735_Cross-section area of internode 3 | G | A |
| 9 | 9783970 | id9002755_Transverse diameter of internode 3 | C | A |
| 9 | 10344006 | id9002846_Cross-section area of internode 3 | A | T |
| 9 | 12664532 | id9003485_Shoot dry weight | G | A |
| 9 | 20941906 | id9007204_Node 3 dry weight | G | T |
| 9 | 22755878 | id9007879_Shoot dry weight | T | C |
| 10 | 10659686 | wd10002398_Transverse diameter of internode 3 | T | C |
| 10 | 22612177 | id10007177_Transverse diameter of internode 4 | C | G |
| 10 | 22612177 | id10007177_Internode 4 dry weight | C | G |
| 11 | 1273238 | id11000272_Node 3 diameter | C | A |
| 11 | 1275872 | id11000275_Node 2 diameter | A | T |
| 11 | 1275872 | id11000275_Node 3 diameter | A | T |
| 11 | 1275872 | id11000275_Internode 2 diameter | A | T |
| 11 | 1275872 | id11000275_Longitudinal diameter of internode 3 | A | T |
| 11 | 1305119 | id11000286_Cross-section area of internode 3 | C | T |
| 11 | 1307788 | id11000293_The average diameter of internode 3 | G | C |
| 11 | 1307788 | id11000293_Longitudinal diameter of internode 3 | G | C |
| 11 | 1307788 | id11000293_Cross-section area of internode 3 | G | C |
| 11 | 1465790 | id11000390_Cross-section area of internode 3 | C | T |
| 11 | 1484113 | id11000392_Cross-section area of internode 3 | A | G |
| 11 | 1512711 | id11000413_Cross-section area of internode 3 | G | A |
| 11 | 3661173 | id11001392_Shoot dry weight | G | A |
| 11 | 4628799 | id11001839_Transverse diameter of internode 3 | T | A |
| 11 | 5357024 | id11002182_Biomass weight | G | A |
| 11 | 9765954 | id11003684_Internode 3 thickness | A | G |
| 11 | 21542705 | id11008193_Node 1 diameter | T | C |
| 11 | 21612962 | id11008239_The average diameter of internode 3 | C | T |
| 11 | 21612962 | id11008239_Transverse diameter of internode 3 | C | T |
| 11 | 21962286 | dd11000488_Transverse diameter of internode 3 | G | A |
| 11 | 21976195 | id11008437_Transverse diameter of internode 3 | G | A |
| 11 | 21978827 | dd11000522_Transverse diameter of internode 3 | G | A |
| 11 | 22106066 | id11008603_Transverse diameter of internode 3 | G | T |
| 11 | 22112337 | id11008620_Transverse diameter of internode 3 | T | C |
| 12 | 5552455 | wd12000455_Panicle length | G | T |
| 12 | 14487883 | id12005213_Node 1 diameter | C | G |
| 12 | 14487883 | id12005213_The average diameter of internode 3 | C | G |
| 12 | 14487883 | id12005213_Longitudinal diameter of internode 3 | C | G |
| 12 | 14487883 | id12005213_Transverse diameter of internode 3 | C | G |
| 12 | 14487883 | id12005213_Shoot dry weight | C | G |

| | | | | |
|---|---|---|---|---|
| 12 | 14487883 | id12005213_Internode 1 dry weight | C | G |
| 12 | 14487883 | id12005213_Node 2 dry weight | C | G |
| 12 | 14488566 | id12005215_Shoot dry weight | T | C |
| 12 | 14659244 | wd12002512_Internode 2 diameter | G | A |
| 12 | 14659244 | wd12002512_The average diameter of internode 3 | G | A |
| 12 | 14659244 | wd12002512_Longitudinal diameter of internode 3 | G | A |
| 12 | 14659244 | wd12002512_Transverse diameter of internode 3 | G | A |
| 12 | 14693039 | ud12000862_The average diameter of internode 3 | C | A |
| 12 | 14693039 | ud12000862_Transverse diameter of internode 3 | C | A |
| 12 | 14954178 | id12005326_Node 1 diameter | G | A |
| 12 | 20913496 | id12006815_Shoot dry weight | T | G |
