# Supplementary material for: Analysis of Genetic Variation of Rice Straw Characteristics and Its Influence on Biomass
Source: Plant Direct. 2026 Jan 6;10(1):e70134. doi: 10.1002/pld3.70134 (PMC12771682; doi:10.1002/pld3.70134)
Supplement: Supplementary file 11 — Table S9: pld370134‐sup‐0011‐Table_S9.pdf. QTLs. The list of 24 candidate genes. [file PLD3-10-e70134-s004.pdf]

README

Column	Description
SNP	uniqe identifier for the SNP
Chromosome	chromosome number where the SNP is located
Position	genomic position of the SNP
QTL	quantitative trait locus(a region of DNA associated with a specific phenotype or trait that varies within a population)
Traits	measured parameters(statistics)
#SNP	number of SNP in QTL
PVALUE	statistical significance
LOP_PVALUE	negative(-) log PVALUE
Yellow color	significant QTLs in GWAS analysis

Table S9. QTLs. The list of 24 candidate genes.

SNP	Chrom	Position	QTL	Traits	#SNP	LOP_PVALUE
id1000027	1	172923	Q1-1	Internode 4 dry weight,Longitudinal diameter o	1	4.01-5.56
id1000529	1	652340	Q1-2	Internode 4 length	1	4.15
id1001247	1	1511711	Q1-3	Internode 3 dry weight,Shoot dry weight	1	5.03-5.53
id1002058	1	2605844	Q1-4	Internode 1 length	1	4.18
id1002770	1	3397754	Q1-5	Internode 4 dry weight	1	5.53
id1007155,id1007156	1	9633974-963	Q1-6	Shoot dry weight	2	5.66
id1008972	1	13555485	Q1-7	Internode 3 thickness	1	4.14
id1013814	1	23790449	Q1-8	Internode 3 thickness	1	4.48
id1014260	1	24242277	Q1-9	Transverse diameter of internode 3	1	4.1
id1016919	1	28726858	Q1-10	Node 4 diameter	1	4.08
id1018646,id1018710	1	30894634-31	Q1-11	Transverse diameter of internode 3,The average	3	4.04-4.96
id1020630	1	33172139	Q1-12	Plant height	1	4.25
id1021743	1	34744079	Q1-13	Cross-section area of internode 3	1	4.08
id1022375	1	35512611	Q1-14	Cross-section area of internode 3	1	4.02
id1024348	1	38363629	Q1-15	Longitudinal diameter of internode 3	1	4.64
id1025227	1	39719133	Q1-16	Biomass weight	1	5.37
dd1001700, dd1001737,i	1	42340167-42	Q1-17	Shoot dry weight,Internode 4 dry weight,Intern	4	5.04-9.97
id2003149	2	6015346	Q2-1	Cross-section area of internode 3	1	4.83
id2009738	2	23775529	Q2-2	Internode 3 thickness	1	7.16
id2011183	2	25630715	Q2-3	Biomass weight	1	4.41
id2011727	2	26574431	Q2-4	Longitudinal diameter of internode 3	1	4.07
id2014525- id2014606	2	32579679-32	Q2-5	Shoot dry weight,Biomass weight	4	6.15-6.27
id2016104-id2016156	2	35241812-35	Q2-6	Node 4 diameter,The average diameter of intern	5	4.01-5.6
id3005558	3	10628270	Q3-1	Internode 4 dry weight	1	8.87
id3006551	3	12696199	Q3-2	Internode 3 thickness	1	4.51
id3007392-id3007659	3	14788478-15	Q3-3	Longitudinal diameter of internode 3,The avera	7	4.15-5.38
id3008667	3	17772284	Q3-4	Biomass weight	1	4.78
id4004869-id4004929	4	16929510-17	Q4-1	Shoot dry weight,Internode 3 dry weight	5	4.83-5.40
id4006198	4	20233912	Q4-2	Internode 4 dry weight	1	8.21
id4010220-id4010433	4	30145846-30	Q4-3	Cross-section area of internode 3,Longitudinal d	5	4.10-4.63
id4011130-id4011523	4	32088424-32	Q4-4	Internode 2 diameter,The average diameter of ir	5	4019-5.77
id5004367	5	8532207	Q5-1	Internode 3 thickness	1	4.92
id5008060-id5008175	5	19664904-19	Q5-2	Internode 2 length,Internode 4 length	4	4.09-4.50
id5009418	5	22082907	Q5-3	Shoot dry weight,Internode 2 dry weight	2	4.20-6.03
id5010361-id5010375	5	23248349-23	Q5-4	Internode 4 length	2	4.32
id5011128	5	24169108	Q5-5	Internode 1 length	1	5.17
id5014595	5	28995509	Q5-6	Biomass weight	1	4.37
id6002230	6	2876987	Q6-1	Panicle length	1	4.14
id6015588	6	27433303	Q6-2	Shoot dry weight	1	5.08
id7000727	7	5073227	Q7-1	Longitudinal diameter of internode 4,The avera	2	4.05-4.17
id7001323-ud7000659	7	7660553-852	Q7-2	Internode 1 length	7	4.07-5.23
id7003855	7	22593381	Q7-3	Transverse diameter of internode 3	1	4.15
id7004429-id7004434	7	24312823-24	Q7-4	Internode 4 length	2	4.5
ud7001914-id7004968	7	26037194-26	Q7-5	Transverse diameter of internode 4,The average	2	4.02-5.37
id8003991-id8004106	8	14876879-15	Q8-1	Longitudinal diameter of internode 4,Node 1 di	2	4.15-5.04
id8004716	8	17729217	Q8-2	Biomass weight	1	5.24
id8007520	8	27424246	Q8-3	The average diameter of internode 4,Longitudin	1	4.10-4.36
id9000693	9	2748188	Q9-1	Internode 4 dry weight	1	5.31
id9002643-id9002755	9	9228486-978	Q9-2	Cross-section area of internode 3,Transverse dia	3	4.07-4.39
id9002846	9	10344006	Q9-3	Cross-section area of internode 3	1	4.09
id9003485	9	12664532	Q9-4	Shoot dry weight	1	6.14
id9007204	9	20941906	Q9-5	Node 3 dry weight	1	4.03
id9007879	9	22755878	Q9-6	Shoot dry weight	1	5.05
wd10002398	10	10659686	Q10-1	Transverse diameter of internode 3	1	4.58
id10007177	10	22612177	Q10-2	Internode 4 dry weight,Transverse diameter of i	1	4.06-13.78
id11000272-id11000413	11	1273238-151	Q11-1	Node 3 diameter,Longitudinal diameter of intern	7	4.18-5.48
id11001392	11	3661173	Q11-2	Shoot dry weight	1	5.02
id11001839	11	4628799	Q11-3	Transverse diameter of internode 3	1	4.48
id11002182	11	5357024	Q11-4	Biomass weight	1	5.12
id11003684	11	9765954	Q11-5	Internode 3 thickness	1	4.24
id11008193-id11008620	11	21542705-22	Q11-6	Node 1 diameter,Transverse diameter of interno	7	4.04-4.76
wd12000455	12	5552455	Q12-1	Panicle length	1	4.69
id12005213-id12005326	12	14487883-14	Q12-2	Shoot dry weight,Node 1 diameter,The average	5	4.02-5.34
id12006815	12	20913496	Q12-3	Shoot dry weight	1	5.43
