# Supplementary material for: Analysis of Genetic Variation of Rice Straw Characteristics and Its Influence on Biomass
Source: Plant Direct. 2026 Jan 6;10(1):e70134. doi: 10.1002/pld3.70134 (PMC12771682; doi:10.1002/pld3.70134)
Supplement: Supplementary file 12 — Table S10: Identification of QTLs with pleiotropic effects (pQTLs). [file PLD3-10-e70134-s009.pdf]

Table S10. Identification of QTLs with pleiotropic effects (pQTLs).

Traits	QTL	Major category	Category of object character	Character	Chromoso	LOD	Intervals	Explained variance	Additive effect
Shoot dry weight,Biomass weight	Q2-5	Physiological trait	Source activity	seed setting	2	7.1	RM240/RM213	4.02	1.7
Shoot dry weight,Internode 3 dry wei	Q4-1	Morphological trait	Root	Deep root weight	4	3.6	RZ69/RG449	12.3	0.023
		Morphological trait	Root	Deep root weight per tiller	4	6.7	RZ69/RG449	17.6	0.0027
		Morphological trait	Panicle/flower	panicle length (PAL)	4		RG788/RZ69		1.17
The average diameter of internode 4,I	Q4-3	Morphological trait	Root	Maximum new root length Average	4	4.4	RM3288/RM5503	16.1	-0.39
		Morphological trait	Panicle/flower	number of primary branches per pan	4	5.8	RM303/RM348	18	0.95
		Morphological trait	Panicle/flower	Spikelet number per panicle(35 d aft	4	5.05	RM303/RM255	11.6	10.35
Internode 2 length,Internode 4 length	Q5-2	Physiological trait	Source activity	degree of chlorophyll content of the	5	11.62	RM440/RM430	12.7	3.93
		Physiological trait	Source activity	degree of mean chlorophyll content	5	12.14	RM440/RM430	21	4.91
		Morphological trait	Seed	1000-grain weight (TGW)	5		RG13/RG573		0.72
		Morphological trait	Panicle/flower	Spikelet density	5	7.7	RG13/RG346	7.7	
		Morphological trait	Root	maximum root length	5			0.12	-1.9
		Morphological trait	Seed	grain width	5	6.31	RM163/RM31	27.1	-0.03
		Morphological trait	Seed	grain thickness	5	4.36	RM163/RM31	19.6	-0.02
		Physiological trait	Eating quality	white belly	5	4.64	RM163/RM31	20.7	-0.22
		Resistance or Tolerance	Blast resistance	field resistance to neck blast	5	2.42	RZ649/RZ225	11.1	
Node 1 diameter,The average diamete	Q11-6	Resistance or Tolerance	Bacterial blight resistance	bacterial blight disease resistance	11		CDO534/RZ537		
Node 1 diameter,Internode 2 diameter	Q12-2	Resistance or Tolerance	Drought tolerance	Grain yield	12	34	RM28048/RM511	33	172
		Resistance or Tolerance	Drought tolerance	Biomass yield	12	23	RM28048/RM28166	18	634
		Resistance or Tolerance	Drought tolerance	Plant height at maturity	12	9.8	RM28048/RM28166	5	1.4
		Resistance or Tolerance	Drought tolerance	Drought response index	12	39	RM28048/RM511	37	0.89

README

Column	Description
Traits	measured parameters(statistics)
QTL	quantitative trait locus(a region of DNA associated with a specific phenotype or trait that varies within a population)
Major category	major category of quantitative character
Category of object character	category of object character
Character	character
Chromosome	chromosome number where the QTL is located
LOD	LOD score:logarithm of the odds(a statistical estimate of the relative probability that two loci are located near each other on a chromosome and are therefore likely to be inherited together)
Intervals	flanking marker of interval mapping A,B
Explained variance	Explained variance
Additive effect	Additive effect
Reference	Reference

Reference
Xing, Y.Z., Xu, C.G., Hua, J.P., and Tan, Y.F. (2001). Analysis of QTL x environment interaction for rice panicle characteristics. Yi Chuan Xue Bao 28, 439-446.
Courtois, B., Shen, L., Petalcorin, W., Carandang, S., Mauleon, R., and Li, Z. (2003). Locating QTLs controlling constitutive root traits in the rice population IAC 165 ~ Co39. Euphytica V134, 335-345.
Courtois, B., Shen, L., Petalcorin, W., Carandang, S., Mauleon, R., and Li, Z. (2003). Locating QTLs controlling constitutive root traits in the rice population IAC 165 ~ Co39. Euphytica V134, 335-345.
Zhuang, J.Y., Lin, H.X., Lu, J., Qian, H.R., Hittalman, S., Huang, N., and Zheng, K.L. (1996). Analysis of QTL x environment interaction for yield components and plant height in rice. Rice Genetics Newsletter 13, 127-129.
Ikeda, H., Kamoshita, A., and Manabe, T. (2006). Genetic analysis of rooting ability of transplanted rice (Oryza sativa L.) under different water conditions. J. Exp. Bot., erl162.
Yamagishi, J., Miyamoto, N., Hirotsu, S., Laza, R.C., and Nemoto, K. (2004). QTLs for branching, floret formation, and pre-flowering floret abortion of rice panicle in a temperate japonica x tropical japonica cross. Theor Appl Genet 109, 1555-1561.
Takai, T., Fukuta, Y., Shiraiwa, T., and Horie, T. (2005). Time-related mapping of quantitative trait loci controlling grain-filling in rice (Oryza sativa L.). J. Exp. Bot. 56, 2107-2118.
Yoo, S.C., Cho, S.H., Zhang, H., Paik, H.C., Lee, C.H., Li, J., Yoo, J.H., Lee, B.W., Koh, H.J., Seo, H.S., and Paek, N.C. (2007). Quantitative trait loci associated with functional stay-green SNU-SG1 in rice. Mol Cells 24, 83-94.
Yoo, S.C., Cho, S.H., Zhang, H., Paik, H.C., Lee, C.H., Li, J., Yoo, J.H., Lee, B.W., Koh, H.J., Seo, H.S., and Paek, N.C. (2007). Quantitative trait loci associated with functional stay-green SNU-SG1 in rice. Mol Cells 24, 83-94.
Zhuang, J.Y., Lin, H.X., Lu, J., Qian, H.R., Hittalman, S., Huang, N., and Zheng, K.L. (1996). Analysis of QTL x environment interaction for yield components and plant height in rice. Rice Genetics Newsletter 13, 127-129.
Li, Z., Pinson, S.R.M., Stansel, J.W., and Paterson, A.H. (1998). Genetic dissection of the source-sink relationship affecting fecundity and yield in rice (shape Oryza sativa L.) Molecular Breeding 4, 419-426.
Tao, Q.N., Wu, P., and Fang, P. (1999). QTLs for rice root morphological characters. Acta Agronomica Sinica 25.
Yoshida, S., Ikegami, M., Kuze, J., Sawada, K., Hashimoto, Z., Ishii, T., Nakamura, C., and Kamijima, O. (2002). QTL Analysis for Plant and Grain Characters of Sake-brewing Rice Using a Doubled Haploid Population. Breeding Science 52, 309-317.
Yoshida, S., Ikegami, M., Kuze, J., Sawada, K., Hashimoto, Z., Ishii, T., Nakamura, C., and Kamijima, O. (2002). QTL Analysis for Plant and Grain Characters of Sake-brewing Rice Using a Doubled Haploid Population. Breeding Science 52, 309-317.
Yoshida, S., Ikegami, M., Kuze, J., Sawada, K., Hashimoto, Z., Ishii, T., Nakamura, C., and Kamijima, O. (2002). QTL Analysis for Plant and Grain Characters of Sake-brewing Rice Using a Doubled Haploid Population. Breeding Science 52, 309-317.
Bagali, P.G., Hittalmani, S., Srinivasachary, Shadakshari, K., and Shashidhar, H.E. (1998). Genetic markers associated with field resistance to leaf and neck blast across locations in rice (Oryza sativa L.). Rice Genetics Newsletter 15, 128-131.
Ronald, P.C., Albano, B., Tabien, R., Abenes, L., Wu, K.S., McCouch, S., and Tanksley, S.D. (1992). Genetic and physical analysis of the rice bacterial blight disease resistance locus, Xa21. Mol Gen Genet 236, 113-120.
Bernier, J., Kumar, A., Ramaiah, V., Spaner, D., and Atlin, G. (2007). A Large-Effect QTL for Grain Yield under Reproductive-Stage Drought Stress in Upland Rice. Crop Sci. 47, 507-516.
Bernier, J., Kumar, A., Ramaiah, V., Spaner, D., and Atlin, G. (2007). A Large-Effect QTL for Grain Yield under Reproductive-Stage Drought Stress in Upland Rice. Crop Sci. 47, 507-516.
Bernier, J., Kumar, A., Ramaiah, V., Spaner, D., and Atlin, G. (2007). A Large-Effect QTL for Grain Yield under Reproductive-Stage Drought Stress in Upland Rice. Crop Sci. 47, 507-516.
Bernier, J., Kumar, A., Ramaiah, V., Spaner, D., and Atlin, G. (2007). A Large-Effect QTL for Grain Yield under Reproductive-Stage Drought Stress in Upland Rice. Crop Sci. 47, 507-516.
