# Supplementary material for: Analysis of Genetic Variation of Rice Straw Characteristics and Its Influence on Biomass
Source: Plant Direct. 2026 Jan 6;10(1):e70134. doi: 10.1002/pld3.70134 (PMC12771682; doi:10.1002/pld3.70134)
Supplement: Supplementary file 13 — Table S11: Path coefficient analysis showing direct (colorful) and indirect effects of various traits in biomass. [file PLD3-10-e70134-s003.pdf]

Table S11. Path coefficient analysis showing direct (collorfull) and indirect effects of various traits in biomass.

README: The numbers on the main diameter indicate direct effects

	Coeff.Nod	Coeff.Nod	Coeff.Nod	Coeff.Nod	Coeff.Inter	Coeff.Inter	Coeff.Lon	Coeff.Tran	Coeff.Lon	Coeff.Tran	Coeff.Inter	Coeff.Cros	Coeff.Stem	Coeff.Pani	Coeff.Num	Coeff.Inter
Node 1 dia	-0.0544	0.046623	0.075634	0.0192	-0.03393	-0.04106	0.173064	-0.02039	-0.09163	-0.17632	0.016913	-0.02954	-0.14161	0.008562	0.013106	0.088227
Node 2 dia	-0.0359	0.070641	0.091677	0.027601	-0.03713	-0.04529	0.180376	-0.02663	-0.11059	-0.23287	0.020816	-0.03256	-0.15448	0.008173	-0.01966	0.105873
Node 3 dia	-0.0359	0.056513	0.114597	0.032801	-0.03649	-0.04347	0.185251	-0.02247	-0.12007	-0.22289	0.022117	-0.03437	-0.13517	0.009146	-0.10485	0.100831
Node 4 dia	-0.02611	0.048742	0.093969	0.040001	-0.02624	-0.03442	0.158438	-0.02289	-0.11375	-0.20293	0.020816	-0.02593	-0.11586	0.005643	-0.00655	0.068061
Internode	-0.02883	0.040972	0.06532	0.0164	-0.06401	-0.03985	0.148688	-0.01914	-0.07741	-0.15303	0.021467	-0.02532	-0.01931	0.00467	-0.10485	0.042853
Internode	-0.03699	0.052981	0.08251	0.0228	-0.04225	-0.06038	0.204751	-0.02913	-0.10585	-0.21957	0.027322	-0.03618	-0.10943	0.008562	-0.04587	0.090748
Longitudin	-0.03862	0.052274	0.087094	0.026001	-0.03905	-0.05072	0.243752	-0.02955	-0.12639	-0.21957	0.024069	-0.03919	-0.12874	0.008951	-0.05242	0.088227
Transverse	-0.02666	0.04521	0.061882	0.022	-0.02944	-0.04227	0.173064	-0.04161	-0.10269	-0.24285	0.02472	-0.03738	-0.07724	0.005254	0.032764	0.083186
Longitudin	-0.03155	0.049449	0.087094	0.028801	-0.03137	-0.04046	0.195001	-0.02705	-0.15799	-0.23953	0.022117	-0.03135	-0.16736	0.008757	0.104845	0.09831
Transverse	-0.02883	0.049449	0.07678	0.024401	-0.02944	-0.03985	0.160876	-0.03038	-0.11375	-0.33268	0.032526	-0.03557	-0.11586	0.008367	0.098292	0.113435
Internode	-0.01414	0.022605	0.038963	0.0128	-0.02112	-0.02536	0.090188	-0.01581	-0.05372	-0.16634	0.065051	-0.02532	0.038621	0.002724	-0.13106	0.060499
Cross-secti	-0.02666	0.038146	0.06532	0.0172	-0.02688	-0.03623	0.158438	-0.0258	-0.08215	-0.19628	0.027322	-0.06029	-0.10943	0.006421	-0.03932	0.09579
Stem lengt	-0.01197	0.016954	0.024065	0.0072	-0.00192	-0.01026	0.04875	-0.00499	-0.04108	-0.05988	-0.0039	-0.01025	-0.64369	0.010702	0.471804	0.168892
Panicle len	-0.02394	0.029669	0.053861	0.0116	-0.01536	-0.02657	0.112126	-0.01124	-0.07109	-0.14305	0.009107	-0.0199	-0.35403	0.019459	0.124504	0.16133
Number of	-0.00109	-0.00212	-0.01834	-0.0004	0.010242	0.004227	-0.0195	-0.00208	-0.02528	-0.0499	-0.01301	0.003618	-0.46345	0.003697	0.655283	0.063019
Internode	-0.01904	0.029669	0.045839	0.0108	-0.01088	-0.02174	0.085313	-0.01373	-0.06162	-0.1497	0.015612	-0.02291	-0.43127	0.012454	0.163821	0.252078
Internode	-0.01142	0.018367	0.035525	0.002	-0.00384	-0.01208	0.060938	-0.00125	-0.03002	-0.00998	-0.00651	-0.00965	-0.53426	0.010313	0.268666	0.146205
Internode	-0.00598	0.005651	0.004584	0.004	0.00256	-0.00242	0.021938	0.000416	-0.01738	0.036594	-0.00585	-0.00181	-0.54713	0.007005	0.465251	0.088227
Internode	0.004352	-0.00636	-0.00344	-0.0012	0.004481	0.004227	-0.02194	0.002913	0.009479	0.039921	-0.00911	0.004823	-0.32828	0.000389	0.281772	0.002521
Panicle dry	-0.01088	0.016247	0.020627	0.0036	-0.01216	-0.01751	0.0585	-0.00707	-0.0395	-0.06654	0.008457	-0.00181	-0.10299	0.005643	0	0.042853
Internode	-0.02611	0.034614	0.056152	0.0148	-0.02112	-0.02717	0.109688	-0.01789	-0.08057	-0.20293	0.021467	-0.02593	-0.22529	0.011092	0.032764	0.153767
Internode	-0.03046	0.04521	0.073342	0.0188	-0.02688	-0.03925	0.153563	-0.02414	-0.09163	-0.20626	0.02472	-0.03316	-0.25747	0.010508	0.04587	0.146205
Internode	-0.02502	0.040265	0.058444	0.022	-0.02176	-0.03442	0.146251	-0.02414	-0.10901	-0.22622	0.017564	-0.03075	-0.35403	0.010119	0.209691	0.136122
Internode	-0.01686	0.026844	0.052715	0.0156	-0.01344	-0.02717	0.114563	-0.01789	-0.07583	-0.14638	0.014962	-0.01628	-0.27035	0.005838	0.183479	0.090748
Node 1 dry	-0.03427	0.046623	0.072196	0.0224	-0.03969	-0.03925	0.158438	-0.02206	-0.10111	-0.1863	0.019515	-0.03195	-0.19954	0.010508	0.013106	0.09831
Node 2 dry	-0.03699	0.051568	0.083656	0.024801	-0.03649	-0.04287	0.170626	-0.0258	-0.11059	-0.2362	0.020816	-0.03195	-0.16736	0.009924	-0.00655	0.108393
Node 3 dry	-0.02992	0.046623	0.081364	0.026401	-0.03585	-0.03502	0.151126	-0.02164	-0.09795	-0.21957	0.020816	-0.02954	-0.08368	0.008367	-0.09829	0.083186

and the remaining numbers indicate indirect effects.Yellow shows direct effects,(-):indicates a negative effect, red:the most direct negative impact, green:the most direct positive impact

Coeff.Inter	Coeff.Inter	Coeff.Inter	Coeff.Pani	Coeff.Inter	Coeff.Inter	Coeff.Inter	Coeff.Inter	Coeff.Nod	Coeff.Nod	Coeff.Node 3 dry weight
0.028752	-0.02438	-0.01763	0.129254	0.083389	0.037099	0.262512	-0.00977	0.011783	0.029646	0.056896
0.035598	-0.01773	-0.01984	0.148642	0.085126	0.042399	0.325287	-0.01197	0.012344	0.031826	0.068275
0.042443	-0.00887	-0.00661	0.116328	0.085126	0.042399	0.291046	-0.01449	0.011783	0.031826	0.073447
0.006846	-0.02216	-0.00661	0.058164	0.064279	0.031136	0.313873	-0.01229	0.010473	0.027031	0.068275
0.008215	0.008866	-0.01543	0.122791	0.05733	0.027824	0.194031	-0.00662	0.011596	0.024851	0.05793
0.027383	-0.00887	-0.01543	0.187418	0.078177	0.043061	0.325287	-0.01418	0.012157	0.030954	0.059999
0.034228	-0.01995	-0.01984	0.155105	0.078177	0.041736	0.342407	-0.01481	0.012157	0.030518	0.064137
0.004107	0.002216	-0.01543	0.109866	0.074703	0.038424	0.330993	-0.01355	0.009912	0.027031	0.053792
0.026014	-0.02438	-0.01322	0.161567	0.088601	0.038424	0.393768	-0.01512	0.01197	0.030518	0.064137
0.004107	0.024381	-0.02645	0.129254	0.105974	0.041074	0.388061	-0.01386	0.010473	0.030954	0.068275
-0.01369	0.019948	-0.03086	0.084015	0.05733	0.025174	0.154083	-0.00725	0.005611	0.013951	0.033103
0.021906	-0.00665	-0.01763	0.019388	0.074703	0.036436	0.291046	-0.00851	0.009912	0.023107	0.050689
0.113638	-0.1884	0.112404	0.103403	0.060805	0.026499	0.313873	-0.01323	0.005798	0.011335	0.013448
0.072564	-0.07979	0.004408	0.187418	0.099025	0.035774	0.296753	-0.00945	0.010099	0.022235	0.044482
0.056135	-0.15737	0.094772	0	0.008686	0.004637	0.182617	-0.00882	0.000374	-0.00044	-0.01552
0.07941	-0.07757	0.002204	0.109866	0.105974	0.038424	0.308166	-0.01134	0.007294	0.018747	0.034137
0.136914	-0.13963	0.017632	0.103403	0.055593	0.029812	0.239685	-0.00252	0.004676	0.011771	0.019655
0.086256	-0.22164	0.090364	0.07109	0.024322	0.011262	0.251098	-0.00536	0.002431	0.003488	0.001034
0.010953	-0.09087	0.2204	0.077552	0.003475	0.005962	0.062775	-0.01701	0.001683	0.000872	-0.01034
0.021906	-0.02438	0.026448	0.646269	0.050381	0.01325	0.102722	-0.00599	0.004489	0.009156	0.008276
0.043812	-0.03103	0.004408	0.187418	0.173727	0.043061	0.313873	-0.01292	0.010099	0.027467	0.052758
0.061611	-0.03768	0.019836	0.129254	0.112923	0.066248	0.405182	-0.0189	0.011596	0.032698	0.068275
0.057504	-0.09752	0.024244	0.116328	0.09555	0.047036	0.570678	-0.02111	0.009725	0.026595	0.055861
0.010953	-0.03768	0.119016	0.122791	0.071228	0.039749	0.382354	-0.0315	0.008229	0.023979	0.040344
0.034228	-0.02881	0.019836	0.155105	0.093813	0.041074	0.296753	-0.01386	0.018703	0.033134	0.063103
0.036967	-0.01773	0.004408	0.135716	0.109448	0.049686	0.348114	-0.01733	0.014214	0.043598	0.08793
0.026014	-0.00222	-0.02204	0.051702	0.088601	0.043724	0.308166	-0.01229	0.011409	0.037058	0.103447
