# Supplementary material for: Analysis of Genetic Variation of Rice Straw Characteristics and Its Influence on Biomass
Source: Plant Direct. 2026 Jan 6;10(1):e70134. doi: 10.1002/pld3.70134 (PMC12771682; doi:10.1002/pld3.70134)
Supplement: Supplementary file 14 — Table S12: Correlation coefficient analysis between the traits. [file PLD3-10-e70134-s006.pdf]

Table S12. Correlation coefficient analysis between the traits.

Traits	Node 1 dia	Node 2 dia	Node 3 dia	Node 4 dia	Internode	Internode	The averag	The averag	Longitudin	Transverse	Longitudin	Transverse	Internode	Cross-secti	Plant heigh
Node 1 diameter	1	0.66	0.66	0.48	0.53	0.68	0.65	0.59	0.71	0.49	0.58	0.53	0.26	0.49	0.29
Node 2 diameter		1	0.8	0.69	0.58	0.75	0.75	0.75	0.74	0.64	0.7	0.7	0.32	0.54	0.28
Node 3 diameter			1	0.82	0.57	0.72	0.71	0.78	0.76	0.54	0.76	0.67	0.34	0.57	0.28
Node 4 diameter				1	0.41	0.57	0.65	0.73	0.65	0.55	0.72	0.61	0.32	0.43	0.22
Internode 1 diameter					1	0.66	0.59	0.52	0.61	0.46	0.49	0.46	0.33	0.42	0.07
Internode 2 diameter						1	0.84	0.72	0.84	0.7	0.67	0.66	0.42	0.6	0.23
The average diameter of internode 3							1	0.83	0.93	0.92	0.78	0.76	0.41	0.69	0.23
The average diameter of internode 4								1	0.79	0.74	0.92	0.93	0.46	0.59	0.33
Longitudinal diameter of internode 3									1	0.71	0.8	0.66	0.37	0.65	0.28
Transverse diameter of internode 3										1	0.65	0.73	0.38	0.62	0.15
Longitudinal diameter of internode 4											1	0.72	0.34	0.52	0.34
Transverse diameter of internode 4												1	0.5	0.59	0.26
Internode 3 thickness													1	0.42	-0.03
Cross-section area of internode 3														1	0.21
Plant height															1
Stem length															
Panicle length															
Number of nodes															
Internode 1 length															
Internode 2 length															
Internode 3 length															
Internode 4 length															
Panicle dry weight															
Shoot dry weight															
Internode 1 dry weight															
Internode 2 dry weight															
Internode 3 dry weight															
Internode 4 dry weight															
Node 1 dry weight															
Node 2 dry weight															
Node 3 dry weight															
Biomass weight															

Stem lengt	Panicle len	Number of	Internode 1	Internode 2	Internode 3	Internode 4	Panicle dry	Shoot dry	Internode 1	Internode 2	Internode 3	Internode 4	Node 1 dry	Node 2 dry	Node 3 dry	Biomass weight
0.22	0.44	0.02	0.35	0.21	0.11	-0.08	0.2	0.49	0.48	0.56	0.46	0.31	0.63	0.68	0.55	0.44
0.24	0.42	-0.03	0.42	0.26	0.08	-0.09	0.23	0.56	0.49	0.64	0.57	0.38	0.66	0.73	0.66	0.51
0.21	0.47	-0.16	0.4	0.31	0.04	-0.03	0.18	0.51	0.49	0.64	0.51	0.46	0.63	0.73	0.71	0.43
0.18	0.29	-0.01	0.27	0.05	0.1	-0.03	0.09	0.54	0.37	0.47	0.55	0.39	0.56	0.62	0.66	0.4
0.03	0.24	-0.16	0.17	0.06	-0.04	-0.07	0.19	0.31	0.33	0.42	0.34	0.21	0.62	0.57	0.56	0.3
0.17	0.44	-0.07	0.36	0.2	0.04	-0.07	0.29	0.55	0.45	0.65	0.57	0.45	0.65	0.71	0.58	0.53
0.18	0.39	-0.02	0.37	0.15	0.04	-0.09	0.22	0.61	0.48	0.66	0.64	0.49	0.64	0.72	0.62	0.53
0.24	0.46	0.16	0.44	0.11	-0.01	-0.09	0.25	0.74	0.59	0.64	0.74	0.49	0.65	0.76	0.69	0.66
0.2	0.46	-0.08	0.35	0.25	0.09	-0.09	0.24	0.56	0.45	0.63	0.6	0.47	0.65	0.7	0.62	0.51
0.12	0.27	0.05	0.33	0.03	-0.01	-0.07	0.17	0.57	0.43	0.58	0.58	0.43	0.53	0.62	0.52	0.47
0.26	0.45	0.16	0.39	0.19	0.11	-0.06	0.25	0.68	0.51	0.58	0.69	0.48	0.64	0.7	0.62	0.63
0.18	0.43	0.15	0.45	0.03	-0.11	-0.12	0.2	0.7	0.61	0.62	0.68	0.44	0.56	0.71	0.66	0.6
-0.06	0.14	-0.2	0.24	-0.1	-0.09	-0.14	0.13	0.24	0.33	0.38	0.27	0.23	0.3	0.32	0.32	0.22
0.17	0.33	-0.06	0.38	0.16	0.03	-0.08	0.03	0.45	0.43	0.55	0.51	0.27	0.53	0.53	0.49	0.3
0.98	0.7	0.66	0.71	0.82	0.81	0.42	0.18	0.64	0.4	0.45	0.59	0.42	0.37	0.32	0.2	0.55
1	0.55	0.72	0.67	0.83	0.85	0.51	0.16	0.62	0.35	0.4	0.55	0.42	0.31	0.26	0.13	0.52
	1	0.19	0.64	0.53	0.36	0.02	0.29	0.54	0.57	0.54	0.52	0.3	0.54	0.51	0.43	0.54
		1	0.25	0.41	0.71	0.43	0	0.45	0.05	0.07	0.32	0.28	0.02	-0.01	-0.15	0.31
			1	0.58	0.35	0.01	0.17	0.6	0.61	0.58	0.54	0.36	0.39	0.43	0.33	0.5
				1	0.63	0.08	0.16	0.44	0.32	0.45	0.42	0.08	0.25	0.27	0.19	0.4
					1	0.41	0.11	0.43	0.14	0.17	0.44	0.17	0.13	0.08	0.01	0.37
						1	0.12	0.25	0.02	0.09	0.11	0.54	0.09	0.02	-0.1	0.25
							1	0.2	0.29	0.2	0.18	0.19	0.24	0.21	0.08	0.75
								1	0.66	0.81	0.9	0.83	0.57	0.69	0.55	0.79
									1	0.65	0.55	0.41	0.54	0.63	0.51	0.62
										1	0.71	0.6	0.62	0.75	0.66	0.66
											1	0.67	0.52	0.61	0.54	0.7
												1	0.44	0.55	0.39	0.67
													1	0.76	0.61	0.5
														1	0.85	0.57
															1	0.4
																1
