# Supplementary material for: Analysis of Genetic Variation of Rice Straw Characteristics and Its Influence on Biomass
Source: Plant Direct. 2026 Jan 6;10(1):e70134. doi: 10.1002/pld3.70134 (PMC12771682; doi:10.1002/pld3.70134)
Supplement: Supplementary file 1 — Data S1: Peer Review. [file PLD3-10-e70134-s010.docx]

Analysis of genetic variation of rice straw characteristics and its influence on biomass

Pär K Ingvarsson, Mahta Mohamadiaza , Naser Farrokhi Asadollah Ahmadikhah , Mehdi Jahanfar.

Decision Letter Round 1:

May 4, 2025

Pär K Ingvarsson

Sveriges lantbruksuniversitet

Plant biology

Box 7080

Uppsala, N/A 71507

Sweden

RE: Analysis of genetic variation of rice straw characteristics and its influence on biomass and stem lodging

Dear Dr. Ingvarsson:

Thank you for submitting to Plant Direct. All required reviews have been returned and we have now finished our evaluation of your manuscript. In light of the reviewers' and editor's comments, further revisions are needed before the paper can be accepted for publication in Plant Direct.

Please view the editors' and reviewers' comments below and use their suggestions as a guide while you work on your revision.

When uploading the revised version of this article, please be sure to include the following:

-A word document that contains your response to the reviewers. You should respond to each reviewer comment and note the changes made to the manuscript. If you do not agree with a reviewer's comment and choose not to make a suggested revision, please explain why. Please try to provide as complete an answer as possible to each reviewer's criticisms in the "response to reviewers" section.

-A tracked changes document

- A clean version of the latest version of the manuscript

Comments from Academic Editor:

After careful review, we feel that the submitted manuscript does not meet Plant Direct's publication criteria. We invite you to submit a revised manuscript that carefully addresses each of the points outlined by Reviewer 1 and the academic editor. A carefully revised and reorganized version may be considered for publication. Key themes include (but are not limited) to:

1. Abbreviations Table: The use of trait abbreviations/codes throughout the text, figures and tables makes it very difficult to follow the biological context of the results. Please replace the abbreviations with trait names throughout.

2. Lodging is not measured in this study: Both reviews note that lodging is not measured in this study. Instead, that may be related to lodging are assessed. Therefore, title and text should be revised to not make conclusions about lodging. A specific section could be added to the Discussion that relates the current study with what is known about lodging resistance.

3. Discussion: The discussion is long and not well organized. Please consider Reviewer 1's suggestions on how to reframe the discussion.

4. Figures: Please carefully reevaluate figures for key components that should be in the main vs. supplementary figures. Please resize all fonts in figures so that they can be read. Currently, many of the results presented in figures could not be evaluated due to size.

5. Readability and clarity: Please carefully edit the manuscript for word use, grammar and clarity.

Detailed review by academic editor:

This manuscript describes genome wide association study on rice traits that are related to biomass and perhaps lodging. A such, the manuscript provides useful knowledge to our understanding of the genetic architecture of important rice traits. However, the study does not include measurements of lodging itself, and therefore the title and text throughout that directly links this study to lodging resistance extends beyond what the data support. While a relationship with lodging could be discussed in a paragraph of the discussion, without addition of an actual lodging trait, lodging should be removed from the title and elsewhere. The manuscript would greatly benefit from careful editing for grammar, clarity and organization. It was often difficult to interpret the intended meaning of the text. Key areas to address:

1. Abstract: Abstract is difficult to read. It should be reorganized such that traits that were measured and used for GWAS are reported, then GWAS results are reported. Text needs to be edited for clarity, flow and grammar.

2. Abbreviations Table: The trait abbreviations make it very difficult to follow the biological results in the manuscript. These abbreviations should be replaced with actual trait names to that results presented in the text and figures and tables are directly linked to the trait names and readers do not have to refer to an abbreviations table. This change will make it much easier to follow the paper and understand the impact of the results.

3. Lodging: The study does not specifically study lodging (i.e. lodging itself is not measured in the population). All direct connections that are made between the results presented in this paper and lodging should be removed. As mentioned above, a possible relationship with lodging could be briefly mentioned in the discussion. For example, candidate genes in this study could be compared to those identified in studies that directly examined lodging, as the authors have cited

(Yadav et al., 2017; Sowadan et al., 2018; Chigira et al., 2020; Guo

et al., 2021; Meng et al., 2021; Nomura et al., 2021; Rashid et al., 2022; Badri et al., 2024). It is unclear whether in the discussion of haplotypes, the authors can identify the allele combinations at specific candidate genes and if that would provide additional insights.

4. The title of the manuscript should be edited to not include lodging.

5. Integration of analyses: The authors present different characterizations of candidate genes, including haplotype analysis (which is particularly difficult to follow), expression patterns from public datasets, epistatic interactions, promoter analysis, etc. However, it is hard to understand how these different analyses come together to give a more coherent story about the candidate genes. The authors might consider modifying one of the tables to include key results from these analyses for the 24 candidate genes so it is clearer how these candidates may function in biomass accumulation and stem traits and how they might interact.

6. Discussion: The discussion is quite long and could be trimmed and reorganized for clarity. A comparison of the results in this study with similar ones in rice would be helpful, particularly when it comes to studies directly involving lodging.

7. Figures and Tables: Many panels in the main figures could be removed and included as supplemental if the authors wish. Currently many elements of the figures are too small to read and therefore the figures are hard to interpret (please see comments on specific figures and tables below). For this reason, some of the results could not be evaluated by the reviewers. Many of the supplemental tables are informal and not enough information is provided to understand what is being presented. Consider instead submitting them in multi-tabbed excel files that will allow for clearer formatting and ReadMe tabs.

Specific comments, by line:

Lines 19 and 20: The phrasing "biodiversity of the internodes of rice straw

20 was put onto prospect for association mapping" is unclear and should be restated.

Lines 26-27: "DUF (1740, 3464, 27 248, 3475, 309, 3444, 295) gene families." This is unclear. Does this mean that some number of candidate genes harbor each of these domains of unknown function and they are being labeled as "gene families"? This needs to be clarified.

Lines 29-30: The text states that candidate genes "were grouped into five general categories". These were grouped relative to GO terms?

Lines 33-34: Haplotype analysis showed that seven haplotypes have significant haplogroups. This sentence is very hard to interpret, particularly "seven haplotypes have significant haplogroups".

Line 67: "result of splitting and repeating long events", is very unclear. Please use standard language in developmental biology.

Lines 70-71: "The internode area at the end of the stem is more mature, while the cells inside the internode at the top of that area reach maturity just before the formation of the next node" This sentence is very hard to interpret. What end of the stem?

Line 77: "Involved in mediating", word seems to be missing. involved in mediating what?

Line 84: It would be helpful to state why/how these 149 were selected out of the set of 282 genotypes.

Line 89: Define the ADMIX subpopulation.

Line 91: "five piles of plants" is unclear. This is unclear. What is a pile? How many plants are in a pile? Perhaps a different word was intended here.

Line 92: Does this mean that from these plants the tallest tiller and the main stem were collected? Please clarify and use a different word than "pile".

Line 107: Define major and minor diameters.

Line 108: Define "the middle point". The middle point of what?

Line 159: Transposable elements are mentioned. If this means that transposable elements were also discarded, rephrase so this is clear.

Line 169: Remove the Error message and replace with correct reference.

Line 183: This link doesn't work.

Line 186: Is "characteristics" meant instead of "characters"?

Line 190: Lodging wasn't measured in this study.

Line 206: Figure 3B does not show a statistically significant SNP on Chromosome 10 for W6. The numbers reported in this paragraph should be carefully cross-referenced with the results in Figure 3 and corrected as needed. Instead of saying "W6 trait", state the actual trait. Also, it doesn't seem to be stated that it is specifically W6 in Figure 3B, however perhaps the font size is a factor.

Line 218: The text in this paragraph is hard to cross-reference with Figure 3C because the QTL designations are not used in 3C. Please add the QTL designations (e.g. Q5-2) to Fig. 3C.

Lines 242-243: Perhaps explicitly state why the H003 haplotype would be preferred in breeding programs (i.e. why is internode 3 thickness important).

Line 244-247: The results presented in this sentence are hard to follow, in part because it is difficult to interpret the information presented in the cited figure. Also, it is not clear what is meant by H001/H003 and H002/H003. Perhaps this means a specific comparison - comparing H001 and H003, or H002 and H003. If that is the case, please rephrase for clarity.

Lines 290-299: IN section 3.4.2., it is unclear whether gene expression data was collected for all 230 genes. It seems unexpected that only 30 of the 230 genes would have available expression data. Figure A, B, and Da have very small fonts and so it is difficult to evaluate the results of these analyses. Also, the text in this section does not summarize the results. It is unclear why the authors would expect that these genes would have stem-specific expression. This section could be expanded for clarity.

Lines 306-307: It is not clear what is meant by "SNPs which have greater bandwidth". Also, should "SNPs that interact more several other SNPs," say "SNPs that interact with several other SNPs"? That is, replace "more" with "with".

Section 3.4.4. It is unclear whether this promoter analysis was performed on the 230 genes within the 64 QTL, for the 24 genes the authors decided to focus on or whether it was a global analysis of all maize genes. "SNPs (repetitive)", though defined earlier, and "12 CREs (repetitive" are difficult to interpret.

Section 3.4.5. This is a very brief section that may not be necessary or should be expanded. Is this section stating that three of the 24 candidate genes that were pursued are TFs?

Section 3.4.1. It would be helpful to quickly define a path analysis and what data it produces. In the first line of this section, should be "where". Also, the numbering of sections seems incorrect. Section 3.4.1 followed section 3.4.5.

Figure 1: The description of internode length in the legend does not match what is shown in the figure. In the figure internode 1 is the longest and internode 3 or 4 looks to be the shortest. The drawing and text should match.

Figure 2: This figure is not needed. The analysis type and method are adequately described in the Methods section.

Figure 3. The legend states, "SNP density

per chromosome along is presented from the center outwards." A word may be missing or "along" could be deleted. For panel C, is each red dot a statistically significant SNP. If so, specifically state this in the legend.

Figure 4: This figure has many elements and many of the individual elements are hard to see at that size and as such, I couldn't review. Authors could consider reorganizing to move some content to supplemental figures and enlarge/focus on the remaining pieces for the main figure. In the legend, the text for (c) is hard to interpret. The haplotype figures are very difficult to interpret. Numbers within each piece of the pie would be helpful and should be clearly defined in the legend. It is odd for the size to vary according to accession number, unless this means that size varies according to the number of accessions in that haplotype. If this is the case, please rephrase for clarity. The symbols on the lines between haplotypes are also hard to interpret. Consider adding the number of variants instead of symbols.

Figure 5. Font sizes are too small in panels A, B and D and so cannot evaluate. The color coding in the PPI plot in Panel D (a) could be shown in a key in the panel. It is not clear what data was used to generate this PPI network. For this figure, please reorganize, enlarge and consider whether some panels could be moved to supplemental figures.

Table 1: In addition to the gene name column, a gene annotation or function column would provide helpful information, particularly for those entries that say only "Similar to.... [protein ID]". A footnote should be added to clarify what is meant the column title, "number of interactions". Presumably, this refers to epistatic interactions identified in section 3.4.3. Note that Table 1 is cited after Table 2 in the text.

Table 2: Title of this table is hard to understand. Also, Table 2 is cited in the text before Table 1. These should be reorganized then.

Figure S1. Please list the name of each trait in each plot instead of the code. The x-axis of each plots needs a label.

Figure S2. Please list what data were used for PCA and population structure analysis.

Figure S4. The trait codes are difficult because they obscure the traits themselves. Please use trait names as labels of these plots.

Figure S5. It is difficult to distinguish between circle sizes and sometimes the numbers within the circles overlap. Also, I think that "accession numbers" should instead be "number of accessions" at each location.

Table S2. The table should have a footnote for each heading that is defined below. "h2b" and "h2n" need to be specifically defined.

Table S4. It seems that some of the definitions have been cut off from the margins. Also, rmCPU, and Pva need to be defined.

Table S8 is very hard to interpret and seems very informal. The title states it is a list of 24 candidate genes, however there are no ZmIDs, or gene names/annotations included in the table. It is also not stated what the yellow highlighting denotes (this comment applies to other supplemental tables that include yellow highlighting). Consider presenting the supplemental tables as tabs in Excel files, with a ReadMe tab for each to that provides definitions and other required information.

Table S11. This table should include the candidate SNP, where it resides relative to the candidate TF, TF gene name, what traits are associated with this SNP (and putatively, TF), etc. Also, the information in the "Function" column is unclear. I'm not sure what "developmental stage", for example, refers to as it relates to the TF. This table needs footnotes to define each column.

Table S12. It is unclear how to interpret this table. For example, it is not clear how these numbers demonstrate direct effects (and can these be positive or negative), or positive or negative indirect effects.

To upload your revision, please click the link below.

https://plantdirect.msubmit.net/cgi-bin/main.plex?el=A5Lr4mu1A4pIk1I4A9ftdAXs56Iu1mCq8V108l67WHQZ

In order to provide as timely a service as possible, we ask that your revision is resubmitted within three months after receipt of this request. If an extension is needed, please send a request, along with a brief explanation, to the editorial office at plantdirect@wiley.com .

Thank you very much for giving us an opportunity to review your work. I look forward to receiving the next version.

Sincerely,

Marna Yandeau-Nelson

---------------------------------------------------------------------------- Editor comments:

After careful review, we feel that the submitted manuscript does not meet Plant Direct's publication criteria. We invite you to submit a revised manuscript that carefully addresses each of the points outlined by Reviewer 1 and the academic editor. A carefully revised and reorganized version may be considered for publication. Key themes include (but are not limited) to:

1. Abbreviations Table: The use of trait abbreviations/codes throughout the text, figures and tables makes it very difficult to follow the biological context of the results. Please replace the abbreviations with trait names throughout.

2. Lodging is not measured in this study: Both reviews note that lodging is not measured in this study. Instead, that may be related to lodging are assessed. Therefore, title and text should be revised to not make conclusions about lodging. A specific section could be added to the Discussion that relates the current study with what is known about lodging resistance.

3. Discussion: The discussion is long and not well organized. Please consider Reviewer 1's suggestions on how to reframe the discussion.

4. Figures: Please carefully reevaluate figures for key components that should be in the main vs. supplementary figures. Please resize all fonts in figures so that they can be read. Currently, many of the results presented in figures could not be evaluated due to size.

5. Readability and clarity: Please carefully edit the manuscript for word use, grammar and clarity.

Detailed review by academic editor:

This manuscript describes genome wide association study on rice traits that are related to biomass and perhaps lodging. A such, the manuscript provides useful knowledge to our understanding of the genetic architecture of important rice traits. However, the study does not include measurements of lodging itself, and therefore the title and text throughout that directly links this study to lodging resistance extends beyond what the data support. While a relationship with lodging could be discussed in a paragraph of the discussion, without addition of an actual lodging trait, lodging should be removed from the title and elsewhere. The manuscript would greatly benefit from careful editing for grammar, clarity and organization. It was often difficult to interpret the intended meaning of the text. Key areas to address:

1. Abstract: Abstract is difficult to read. It should be reorganized such that traits that were measured and used for GWAS are reported, then GWAS results are reported. Text needs to be edited for clarity, flow and grammar.

2. Abbreviations Table: The trait abbreviations make it very difficult to follow the biological results in the manuscript. These abbreviations should be replaced with actual trait names to that results presented in the text and figures and tables are directly linked to the trait names and readers do not have to refer to an abbreviations table. This change will make it much easier to follow the paper and understand the impact of the results.

3. Lodging: The study does not specifically study lodging (i.e. lodging itself is not measured in the population). All direct connections that are made between the results presented in this paper and lodging should be removed. As mentioned above, a possible relationship with lodging could be briefly mentioned in the discussion. For example, candidate genes in this study could be compared to those identified in studies that directly examined lodging, as the authors have cited

(Yadav et al., 2017; Sowadan et al., 2018; Chigira et al., 2020; Guo

et al., 2021; Meng et al., 2021; Nomura et al., 2021; Rashid et al., 2022; Badri et al., 2024). It is unclear whether in the discussion of haplotypes, the authors can identify the allele combinations at specific candidate genes and if that would provide additional insights.

4. The title of the manuscript should be edited to not include lodging.

5. Integration of analyses: The authors present different characterizations of candidate genes, including haplotype analysis (which is particularly difficult to follow), expression patterns from public datasets, epistatic interactions, promoter analysis, etc. However, it is hard to understand how these different analyses come together to give a more coherent story about the candidate genes. The authors might consider modifying one of the tables to include key results from these analyses for the 24 candidate genes so it is clearer how these candidates may function in biomass accumulation and stem traits and how they might interact.

6. Discussion: The discussion is quite long and could be trimmed and reorganized for clarity. A comparison of the results in this study with similar ones in rice would be helpful, particularly when it comes to studies directly involving lodging.

7. Figures and Tables: Many panels in the main figures could be removed and included as supplemental if the authors wish. Currently many elements of the figures are too small to read and therefore the figures are hard to interpret (please see comments on specific figures and tables below). For this reason, some of the results could not be evaluated by the reviewers. Many of the supplemental tables are informal and not enough information is provided to understand what is being presented. Consider instead submitting them in multi-tabbed excel files that will allow for clearer formatting and ReadMe tabs.

Specific comments, by line:

Lines 19 and 20: The phrasing "biodiversity of the internodes of rice straw

20 was put onto prospect for association mapping" is unclear and should be restated.

Lines 26-27: "DUF (1740, 3464, 27 248, 3475, 309, 3444, 295) gene families." This is unclear. Does this mean that some number of candidate genes harbor each of these domains of unknown function and they are being labeled as "gene families"? This needs to be clarified.

Lines 29-30: The text states that candidate genes "were grouped into five general categories". These were grouped relative to GO terms?

Lines 33-34: Haplotype analysis showed that seven haplotypes have significant haplogroups. This sentence is very hard to interpret, particularly "seven haplotypes have significant haplogroups".

Line 67: "result of splitting and repeating long events", is very unclear. Please use standard language in developmental biology.

Lines 70-71: "The internode area at the end of the stem is more mature, while the cells inside the internode at the top of that area reach maturity just before the formation of the next node" This sentence is very hard to interpret. What end of the stem?

Line 77: "Involved in mediating", word seems to be missing. involved in mediating what?

Line 84: It would be helpful to state why/how these 149 were selected out of the set of 282 genotypes.

Line 89: Define the ADMIX subpopulation.

Line 91: "five piles of plants" is unclear. This is unclear. What is a pile? How many plants are in a pile? Perhaps a different word was intended here.

Line 92: Does this mean that from these plants the tallest tiller and the main stem were collected? Please clarify and use a different word than "pile".

Line 107: Define major and minor diameters.

Line 108: Define "the middle point". The middle point of what?

Line 159: Transposable elements are mentioned. If this means that transposable elements were also discarded, rephrase so this is clear.

Line 169: Remove the Error message and replace with correct reference.

Line 183: This link doesn't work.

Line 186: Is "characteristics" meant instead of "characters"?

Line 190: Lodging wasn't measured in this study.

Line 206: Figure 3B does not show a statistically significant SNP on Chromosome 10 for W6. The numbers reported in this paragraph should be carefully cross-referenced with the results in Figure 3 and corrected as needed. Instead of saying "W6 trait", state the actual trait. Also, it doesn't seem to be stated that it is specifically W6 in Figure 3B, however perhaps the font size is a factor.

Line 218: The text in this paragraph is hard to cross-reference with Figure 3C because the QTL designations are not used in 3C. Please add the QTL designations (e.g. Q5-2) to Fig. 3C.

Lines 242-243: Perhaps explicitly state why the H003 haplotype would be preferred in breeding programs (i.e. why is internode 3 thickness important).

Line 244-247: The results presented in this sentence are hard to follow, in part because it is difficult to interpret the information presented in the cited figure. Also, it is not clear what is meant by H001/H003 and H002/H003. Perhaps this means a specific comparison - comparing H001 and H003, or H002 and H003. If that is the case, please rephrase for clarity.

Lines 290-299: IN section 3.4.2., it is unclear whether gene expression data was collected for all 230 genes. It seems unexpected that only 30 of the 230 genes would have available expression data. Figure A, B, and Da have very small fonts and so it is difficult to evaluate the results of these analyses. Also, the text in this section does not summarize the results. It is unclear why the authors would expect that these genes would have stem-specific expression. This section could be expanded for clarity.

Lines 306-307: It is not clear what is meant by "SNPs which have greater bandwidth". Also, should "SNPs that interact more several other SNPs," say "SNPs that interact with several other SNPs"? That is, replace "more" with "with".

Section 3.4.4. It is unclear whether this promoter analysis was performed on the 230 genes within the 64 QTL, for the 24 genes the authors decided to focus on or whether it was a global analysis of all maize genes. "SNPs (repetitive)", though defined earlier, and "12 CREs (repetitive" are difficult to interpret.

Section 3.4.5. This is a very brief section that may not be necessary or should be expanded. Is this section stating that three of the 24 candidate genes that were pursued are TFs?

Section 3.4.1. It would be helpful to quickly define a path analysis and what data it produces. In the first line of this section, should be "where". Also, the numbering of sections seems incorrect. Section 3.4.1 followed section 3.4.5.

Figure 1: The description of internode length in the legend does not match what is shown in the figure. In the figure internode 1 is the longest and internode 3 or 4 looks to be the shortest. The drawing and text should match.

Figure 2: This figure is not needed. The analysis type and method are adequately described in the Methods section.

Figure 3. The legend states, "SNP density

per chromosome along is presented from the center outwards." A word may be missing or "along" could be deleted. For panel C, is each red dot a statistically significant SNP. If so, specifically state this in the legend.

Figure 4: This figure has many elements and many of the individual elements are hard to see at that size and as such, I couldn't review. Authors could consider reorganizing to move some content to supplemental figures and enlarge/focus on the remaining pieces for the main figure. In the legend, the text for (c) is hard to interpret. The haplotype figures are very difficult to interpret. Numbers within each piece of the pie would be helpful and should be clearly defined in the legend. It is odd for the size to vary according to accession number, unless this means that size varies according to the number of accessions in that haplotype. If this is the case, please rephrase for clarity. The symbols on the lines between haplotypes are also hard to interpret. Consider adding the number of variants instead of symbols.

Figure 5. Font sizes are too small in panels A, B and D and so cannot evaluate. The color coding in the PPI plot in Panel D (a) could be shown in a key in the panel. It is not clear what data was used to generate this PPI network. For this figure, please reorganize, enlarge and consider whether some panels could be moved to supplemental figures.

Table 1: In addition to the gene name column, a gene annotation or function column would provide helpful information, particularly for those entries that say only "Similar to.... [protein ID]". A footnote should be added to clarify what is meant the column title, "number of interactions". Presumably, this refers to epistatic interactions identified in section 3.4.3. Note that Table 1 is cited after Table 2 in the text.

Table 2: Title of this table is hard to understand. Also, Table 2 is cited in the text before Table 1. These should be reorganized then.

Figure S1. Please list the name of each trait in each plot instead of the code. The x-axis of each plots needs a label.

Figure S2. Please list what data were used for PCA and population structure analysis.

Figure S4. The trait codes are difficult because they obscure the traits themselves. Please use trait names as labels of these plots.

Figure S5. It is difficult to distinguish between circle sizes and sometimes the numbers within the circles overlap. Also, I think that "accession numbers" should instead be "number of accessions" at each location.

Table S2. The table should have a footnote for each heading that is defined below. "h2b" and "h2n" need to be specifically defined.

Table S4. It seems that some of the definitions have been cut off from the margins. Also, rmCPU, and Pva need to be defined.

Table S8 is very hard to interpret and seems very informal. The title states it is a list of 24 candidate genes, however there are no ZmIDs, or gene names/annotations included in the table. It is also not stated what the yellow highlighting denotes (this comment applies to other supplemental tables that include yellow highlighting). Consider presenting the supplemental tables as tabs in Excel files, with a ReadMe tab for each to that provides definitions and other required information.

Table S11. This table should include the candidate SNP, where it resides relative to the candidate TF, TF gene name, what traits are associated with this SNP (and putatively, TF), etc. Also, the information in the "Function" column is unclear. I'm not sure what "developmental stage", for example, refers to as it relates to the TF. This table needs footnotes to define each column.

Table S12. It is unclear how to interpret this table. For example, it is not clear how these numbers demonstrate direct effects (and can these be positive or negative), or positive or negative indirect effects.

---------------------------------------------------------------------------- Reviewer comments:

Reviewer #1:

I have attached my comments as a separate word file.

Reviewer #2:

The authors conducted a genome-wide association study on 149 rice accessions using obtained morphological traits with genotype data of SNP array published over ten years ago. They performed several post-GWAS analyses, including haplotype variations and expression analyses. However, the presented data are preliminary and not well organized, failing to meet acceptable technical criteria. In addition to poor description, several results were derived from the previously published or online data, and did not yield insightful conclusions. The results should be reorganized effectively and legibly using appropriate figures, tables and dataset for conclusiveness and clarity.

Decision Letter Round 2:

July 17, 2025

Pär K Ingvarsson

Sveriges lantbruksuniversitet

Plant biology

Box 7080

Uppsala, N/A 71507

Sweden

RE: Analysis of genetic variation of rice straw characteristics and its influence on biomass

Dear Dr. Ingvarsson:

Thank you for submitting to Plant Direct. All required reviews have been returned and we have now finished our evaluation of your manuscript. In light of the reviewers' and editor's comments, further revisions are needed before the paper can be accepted for publication in Plant Direct.

Please view the editors' and reviewers' comments below and use their suggestions as a guide while you work on your revision.

When uploading the revised version of this article, please be sure to include the following:

-A word document that contains your response to the reviewers. You should respond to each reviewer comment and note the changes made to the manuscript. If you do not agree with a reviewer's comment and choose not to make a suggested revision, please explain why. Please try to provide as complete an answer as possible to each reviewer's criticisms in the "response to reviewers" section.

-A tracked changes document

- A clean version of the latest version of the manuscript

Comments from Academic Editor:

The revised version addresses several of the concerns raised by the reviewers and academic editor. However, after careful review, we feel that the revised manuscript does not meet Plant Direct's publication criteria. We invite you to submit a revised manuscript that carefully addresses each of the points outlined by Reviewer 3. In addition, it seems that the specific comments provided for the original submission by Reviewer 1 were not addressed. These have been copied below for your consideration. A carefully revised version may be considered for publication.

Key themes to address:

1. Methods: Key details should be added to the methods for experimental design and timing of sampling traits, conducting GWAS, calculating heritability (formula used), assessing population structure and kinship, model type (fixed vs random effects), and other details as listed by Reviewer 3 and Reviewer 1.

2. Pleiotropy: Consider Reviewer 3's comments regarding alternate methods for testing for pleiotropy, including multivariate multiple-testing adjustment, or a multi-trait GWAS method.

3. Candidate genes: Consider the cases being made to name a gene as a candidate underlying biomass traits. Many of these candidates seem minimally supported, and SNP effects or gene-level allele effects are not reported. Reviewer 3 provides many comments regarding genes listed as candidates.

4. Organization of Results and Discussion: Please consider reordering topics in the Results and Discussion for better flow and clarity (see comments by Reviewer 3).

5. Figure legends for the main figures do not seem to be included in the revised manuscript.

Comments provided by Reviewer 1 (original submission, comments provided as an attachment perhaps not seen by authors):

In the manuscript by Mohamadiaza et al., entitled "Analysis of genetic variation of rice straw characteristics and its influence on biomass and stem lodging," authors used a GWA approach to identify candidate genes for biomass and stalk lodging resistance. Overall, the methods are sound, and the manuscript is well written. Below I outline minor comments that would improve the manuscript.

1. The abbreviation table and use of abbreviations throughout the manuscript is distracting and confusing. These abbreviations are not always intuitive and make the interpretability of results difficult. While abbreviations are OK for figures and tables (with the abbreviation spelled out in the legend), I suggest writing out the traits in the manuscript for ease of interpretability.

2. The manuscript wants to link biomass and lodging, however, lodging data was not collected. I think this is a reach and can be discussed in the discussion, however, as the manuscript stands now the authors refer to lodging when they lack data to support this.

-There are many examples throughout, however one includes on line 189-190 and a second example on lines 287-288.

3. Line 70: "area at the end of the stem" this is confusing. I suggest being more specific and possibly referencing Figure 1. The area of the stem closest to the ground perhaps?

4. Experimental Design/Section 2.1:

-Line 86: How many plants were in a plot?

-Line 90: Were the three biological replicates = to three total plants? Were these three plants from a single plot? Was this one plant from each plot and three plots total?

- Line 91: What does "five piles of plants from each accession" mean? Five plants per plot?

- How long after harvest were these traits measured? I would expect node and internode diameters to change (not uniformly) due to drying/storage at 25 degrees C.

5. Lines 141-149:

-Were all of these candidate genes associated with a significant SNP with a-log10(p) > 4? Or was this just true for #3 in the list?

-Was a single gene for each SNP chosen? Or was it possible that more than one candidate gene was chosen if it fit the criteria listed in this section?

6. Line 168/169: Error with this citation generation.

7. Line 170 (and elsewhere): What was included in your model as fixed and random effects? For example, how did you calculate heritability?

8. Line 174: Why was 300 kb chosen as a window size? Is this a standard size for rice?

9. Line 206: The W6 trait has the strongest association. However, W6 also has a lot of genetic variation (Line 191 and 192), while also having multiple SNPs across multiple chromosomes having an effect (Figure 3B). I think this is worth elaborating on in the discussion.

10. Line 215 and Table S9: Where did these 11 highlighted SNPs come from? How were these chosen. It is confusing how above (Line 207) 173 significant SNPs were discussed and then you end up discussing 11 a few lines later. At this point it becomes confusing the criteria used to select these. You discuss on lines 141-149 that multiple criteria were considered from selecting candidate genes. It might make more sense to break these results into sections to clearly explain how different results were determined.

11. Discussion (overall) reads like a laundry list and is not very organized. I suggest reframing this to highlight the goals of this project and how your results (collectively) fill these gaps with support from additional studies. Many traits were tested in this experiment (and many of which are correlated), so going through each class of genes and significant associations (as it is now) makes it difficult to collectively synthesize the results and findings in a greater context.

12. Figure 1: The legend and the figure do not match. Specifically, the legend illustrates the internode length getting larger from bottom (internode 4) to top (internode 1). Yet the legend does not describe these size comparisons this way.

13. Figure 2: This does not need to be a main body figure, in my opinion. This adds no context to the manuscript/methods that can not be described in the text.

14. The overall quality of Figures is poor but maybe this is a result of PDF generation during submission?

15. Figure 3: How does the information in panel A and panel B differ? What is found in one that is not found in the other? I don't think these are both necessary for a main body figure.

16. Figure 4: The quality of this figure/text size made it impossible to evaluate. However, I suspect that this is not necessary for a main body figure. What is the take home here? What is needed for the main body and what can be moved to supplement?

17. Figure 5: I was also unable to evaluate this due to text size and quality. Same comment as above.

18. Figure S1: What do the colors mean?

To upload your revision, please click the link below.

https://plantdirect.msubmit.net/cgi-bin/main.plex?el=A6Lr7mu2B4pIk1I5A9ftdAXs56Iu1mCq8V108l67WHQZ

In order to provide as timely a service as possible, we ask that your revision is resubmitted within three months after receipt of this request. If an extension is needed, please send a request, along with a brief explanation, to the editorial office at plantdirect@wiley.com .

Thank you very much for giving us an opportunity to review your work. I look forward to receiving the next version.

Sincerely,

Marna Yandeau-Nelson

---------------------------------------------------------------------------- Editor comments:

The revised version addresses several of the concerns raised by the reviewers and academic editor. However, after careful review, we feel that the revised manuscript does not meet Plant Direct's publication criteria. We invite you to submit a revised manuscript that carefully addresses each of the points outlined by Reviewer 3. In addition, it seems that the specific comments provided for the original submission by Reviewer 1 were not addressed. These have been copied below for your consideration. A carefully revised version may be considered for publication.

Key themes to address:

1. Methods: Key details should be added to the methods for experimental design and timing of sampling traits, conducting GWAS, calculating heritability (formula used), assessing population structure and kinship, model type (fixed vs random effects), and other details as listed by Reviewer 3 and Reviewer 1.

2. Pleiotropy: Consider Reviewer 3's comments regarding alternate methods for testing for pleiotropy, including multivariate multiple-testing adjustment, or a multi-trait GWAS method.

3. Candidate genes: Consider the cases being made to name a gene as a candidate underlying biomass traits. Many of these candidates seem minimally supported, and SNP effects or gene-level allele effects are not reported. Reviewer 3 provides many comments regarding genes listed as candidates.

4. Organization of Results and Discussion: Please consider reordering topics in the Results and Discussion for better flow and clarity (see comments by Reviewer 3).

5. Figure legends for the main figures do not seem to be included in the revised manuscript.

Comments provided by Reviewer 1 (original submission):

In the manuscript by Mohamadiaza et al., entitled "Analysis of genetic variation of rice straw characteristics and its influence on biomass and stem lodging," authors used a GWA approach to identify candidate genes for biomass and stalk lodging resistance. Overall, the methods are sound, and the manuscript is well written. Below I outline minor comments that would improve the manuscript.

1. The abbreviation table and use of abbreviations throughout the manuscript is distracting and confusing. These abbreviations are not always intuitive and make the interpretability of results difficult. While abbreviations are OK for figures and tables (with the abbreviation spelled out in the legend), I suggest writing out the traits in the manuscript for ease of interpretability.

2. The manuscript wants to link biomass and lodging, however, lodging data was not collected. I think this is a reach and can be discussed in the discussion, however, as the manuscript stands now the authors refer to lodging when they lack data to support this.

-There are many examples throughout, however one includes on line 189-190 and a second example on lines 287-288.

3. Line 70: "area at the end of the stem" this is confusing. I suggest being more specific and possibly referencing Figure 1. The area of the stem closest to the ground perhaps?

4. Experimental Design/Section 2.1:

-Line 86: How many plants were in a plot?

-Line 90: Were the three biological replicates = to three total plants? Were these three plants from a single plot? Was this one plant from each plot and three plots total?

- Line 91: What does "five piles of plants from each accession" mean? Five plants per plot?

- How long after harvest were these traits measured? I would expect node and internode diameters to change (not uniformly) due to drying/storage at 25 degrees C.

5. Lines 141-149:

-Were all of these candidate genes associated with a significant SNP with a-log10(p) > 4? Or was this just true for #3 in the list?

-Was a single gene for each SNP chosen? Or was it possible that more than one candidate gene was chosen if it fit the criteria listed in this section?

6. Line 168/169: Error with this citation generation.

7. Line 170 (and elsewhere): What was included in your model as fixed and random effects? For example, how did you calculate heritability?

8. Line 174: Why was 300 kb chosen as a window size? Is this a standard size for rice?

9. Line 206: The W6 trait has the strongest association. However, W6 also has a lot of genetic variation (Line 191 and 192), while also having multiple SNPs across multiple chromosomes having an effect (Figure 3B). I think this is worth elaborating on in the discussion.

10. Line 215 and Table S9: Where did these 11 highlighted SNPs come from? How were these chosen. It is confusing how above (Line 207) 173 significant SNPs were discussed and then you end up discussing 11 a few lines later. At this point it becomes confusing the criteria used to select these. You discuss on lines 141-149 that multiple criteria were considered from selecting candidate genes. It might make more sense to break these results into sections to clearly explain how different results were determined.

11. Discussion (overall) reads like a laundry list and is not very organized. I suggest reframing this to highlight the goals of this project and how your results (collectively) fill these gaps with support from additional studies. Many traits were tested in this experiment (and many of which are correlated), so going through each class of genes and significant associations (as it is now) makes it difficult to collectively synthesize the results and findings in a greater context.

12. Figure 1: The legend and the figure do not match. Specifically, the legend illustrates the internode length getting larger from bottom (internode 4) to top (internode 1). Yet the legend does not describe these size comparisons this way.

13. Figure 2: This does not need to be a main body figure, in my opinion. This adds no context to the manuscript/methods that can not be described in the text.

14. The overall quality of Figures is poor but maybe this is a result of PDF generation during submission?

15. Figure 3: How does the information in panel A and panel B differ? What is found in one that is not found in the other? I don't think these are both necessary for a main body figure.

16. Figure 4: The quality of this figure/text size made it impossible to evaluate. However, I suspect that this is not necessary for a main body figure. What is the take home here? What is needed for the main body and what can be moved to supplement?

17. Figure 5: I was also unable to evaluate this due to text size and quality. Same comment as above.

18. Figure S1: What do the colors mean?

---------------------------------------------------------------------------- Reviewer comments:

Reviewer #3:

(Starting at Page 83):

- Overall:

- Introduction:

- Consider collating all uses of RS into a single section. As it stands, there are multiple disparate areas where the use of RS is discussed

- Methods:

- Descriptions of methods are very brief, and as such, are both difficult to follow and non-informative. Key details are often missing, and there is no clear logical progression of steps in the process of analysis.

- Results:

- Results and introductions of analyses are often missing biological or rational justification, or such justification is very limited

- Claims are often missing proper citation: e.g. Page 99 "CREs generally play a role in plant growth, development, and stress response."

- Analysis of GWAS results identifies candidate genes by large haplotype groups, but does not indicate whether there is evidence to implicate individual genes in stalk biomass (e.g. SNP effect, gene-level allele effects, etc.)

- The ordering of results makes things difficult to follow. For example, path analysis concerning the correlation of multiple traits is nested between a section on candidate transcription factors and a PPI Network. Trait correlations could be considered prior to GWAS, and reporting on candidate genes should come after all analyses.

- Discussion:

- Many candidate genes are cited as having functions in "plant grown and development." This is not sufficient evidence to support their identification as purportedly regulating stalk biomass

- Compounding with the issues listed for "Results," "Discussion" is difficult to follow, and does not follow a clear logical flow.

- Results are often presented in "Discussion," but their relevance to the traits of interest is unclear.

- Line-by-line (Starting on Page 83 to see previous edits)

- 92-94: It is unclear whether 6 T/ha is the waste generated per year, and what "370-520 million tons" refers to, whether that be waste or grain yield

- 94: What environmental issues may this refer to?

- 99-100: From this single sentence, it is unclear why lodging is discussed amid a section on RS waste

- 109: Cellular elongation does not produce new cells

- 109-111: From this single sentence, it is not clear how stem architecture is arranged

- 118: "GWAS study" is redundant, as in 'genome-wide association study study'

- 118-122: Are the goals of this study to identify candidates for biomass production, beneficial RS composition, or limit lodging? What are the "node and internode traits" quantified? How many rice accessions are studied? What is the total SNP count or density?

- 125: How was this subset of rice lines selected?

- 126: How did these rice lines adapt?

- 131-134: It is unclear how samples were collected and quantified. What does "five hills" refer to?

- 134: What does "were selected" refer to in this context? Were the 3 tallest plants (based on tiller height) selected as the three biological replicates (as referenced earlier in the text)? Is the "main stem" of only one plant collected?

- 150-154: An equation is provided for the diameter of an ellipse, but is is unclear how the thickness of the third internode was quantified. Was the wall-thickness quantified at a single point, or multiple points? Was there variation in the thickness of different axes? If not, was thickness quantified across the transverse or longitudinal axis?

- 156-160: Which genome or SNP assembly was used, specifically?

- 163-168: Plotting in and of itself does not characterize population structure. Was each PC calculated using genome-wide SNP genotypes, or were PCs calculated using some other measure. If so, which panel of SNPs was used for PCA? 44.1K SNPs or 34.2K SNPs? This needs to be elaborated. Based on Figure S1, there is clear separation of accessions into one of three groups. Do these groupings correspond to heterologous group or ancestry? How was clustering determined? What parameters were used for the clustering algorithm employed? It is unclear whether these principle components, nor how many, were included in the GWAS models. See further comments of Figure S1 below.

- 168-171: Methods must include how kinship was determined and what this kinship measures. Was kinship based on genome-wide SNP identity, representing the additive genetic similarity? Was kinship based on pedigree? etc. Which algorithm specifically was used to estimate this kinship matrix?

- 173-176 It is unclear why (or if) the authors elected to use two different GWAS algorithms. Line 176 indicates that only FarmCPU was used, but MLM is mentioned on Line 173. If FarmCPU was the only algorithm used, then estimating kinship for the panel of rice accessions is meaningless.

- 173-174: It is unclear whether trait errors are normally distributed. If errors are skewed (as with the left/right skewed trait distributions), employ either a Box-Cox or logarithmic transformation.

- 177: It is not mentioned how this p-value threshold is set.

- 181-184: This is not an adequate test for pleiotropy, owing to multiple testing and correlated traits. Consider using a multivariate multiple-testing adjustment, such as ASH or MASH, or a multi-trait GWAS algorithm. It is unclear how QT information from Q-TARO was utilized in this study.

- 184-186: What results were visualized?

- 210-247: Multiple times within this section it is mentioned that analysis was performed (using some package or database), but fail to mention what each analysis actually entailed.

- 273: It is unclear why unadjusted p-value thresholds (-log10 > 4) are used when multiple testing (FDR < 0.05) is a superior measure of significance for GWAS

- 282: It is not stated how 11 QTL regions are identified from the intial set of 173 significant SNPs

- 288: It is not stated what other functions were identified in this set of 230 genes, aside from cell wall metabolism or biomass production.

- 297-299: It is unclear to me whether haplotypes and haplogroups are estimated using significant SNPs or using all available gene-wide SNPs, as this section mentions both "all SNPs" and SNPs "that appeared in more than one trait." If

- 305: What portion of SNPs were classified as pleiotropic?

- 313-318: This section is confusing. Does "QTL" refer to H001-H003? What LD threshold defines a block? Does "allelic variation" refer to haplotype variants or to the two SNP markers?

- 327-372: See comments on sections covering Chromosomes 1 and 2

- 377: Missing an overview of these 30 genes. What are their functions?

- 380-383: It is unclear what this is an example of, or what this is meant to convey. If results were not stem-specific, what was the ratio of stem expression to other tissues? What about different stages of development? Are these genes likely to be involved in stem biomass (unclear, as the section only refers to these as genes with significant SNPs)?

- 388: What portion of genes are involved in epistatic interactions? Are the a small number of genes that explain many of the 110 interactions? Or are the 110 interactions mainly unique gene-gene pairwise interactions?

- 389: Does this p-value account for multiple pairwise testing?

- (Pages 99-101 are missing line numbers)

- 3.4.4:

- What is the promoter analysis? Why was it performed?

- It is unclear where the "64 QTLs" come from, given that only 11 QTLs were highlighted previously.

- What are the "reported functions" used to identify 24 candidate genes?

- "38 SNPs due to some being reported for different traits." I do not understand the meaning of this sentence.

- It is unclear what "promoter region of different genes" refers to.

- 3.4.5:

- It is unclear what "the results" is referring to.

- It is unclear whether "GWAS signals" refers to SNPs, haplotypes, or QTL regions

- Many claims on transcription factor function are missing appropriate citation

- 3.4.6:

- It is unclear whether any of these associations are significant or not

- Given the high degree of correlation between traits, it is necessary to account for trait correlations during post-GWAS or GWAS analyses (e.g. multi-trait GWAS, multi-trait multiple testing adjustment, or multi-trait screening such as ASSET in R)

- (Page 106 resets line numbering to "1")

- 3.4.7 Line 4: Is "candidate genes" referring to the 230 candidate genes? If so, "candidate genes" cannot be used to identify 230 candidate genes -- the logic is circular.

- 7: It is unclear why the interaction between these three genes is noteworthy, nor what "other candidate proteins" refer to.

- 2-15: The section on the PPI Network does not convey any compelling results as currently written.

- 54: "RS" has not been defined within the discussion

- 57-66: None of these claims are properly cited

- 78-79: Missing citation, or list of constituent traits

- 79-84: The importance of multi-trait GWAS is stressed, but analysis performed within this paper does not seem to include proper multi-trait GWAS considerations

- 86: It is unclear where "6 QTLs" comes from, as the previous number of QTLs from results were 11 and 64

- 98: Missing citation to previous research

- 104: What number have not been reported?

- 125-131: This is not adequate evidence to connect stalk biomass and actin

- 141: Mutants in what organism?

- 155: Pleiotropic effects on what traits?

- 159: What number of target traits? Were these multiple target traits highly correlated?

- 170: See comments on "159"

- 171-375: See comments on 125-170.

- 386-390: The third and fourth internode only represent a portion of all traits analyzed. As GWAS results do not directly reference the number of GWAS candidate associated with the third and fourth internode, this claim is overreaching.

- As I continue reading, I see that presentation of these results comes AFTER this section, in Haplotype analysis.

- 396-399: It is recommended to condense this section on haplotype analysis into the initial overview as included in Results and Methods

- 447: It is unclear how "important interaction" is being quantified.

- Figures and Tables:

- Table 1: The layout of this table is unintuitive, given that there is no association between traits belonging to the same row. Column titles may be more clear if they referred to general trait classes (e.g. 'Diameter Traits'). Units (mm, mg, cm) do not seem necessary if the goal of this table is to summarize which traits were collected throughout the study.

- Figure S1: A biplot in and of itself does not characterize population structure or stratification. Rather, this is a method of visualization. Methods must include section on clustering or the generation of population principle components. See further comments on lines 163-168.

Decision Letter Round 3:

September 19, 2025

Pär K Ingvarsson

Sveriges lantbruksuniversitet

Plant biology

Box 7080

Uppsala, N/A 71507

Sweden

RE: Analysis of genetic variation of rice straw characteristics and its influence on biomass

Dear Dr. Ingvarsson:

Thank you for submitting to Plant Direct. All required reviews have been returned and we have now finished our evaluation of your manuscript. In light of the reviewers' and editor's comments, further revisions are needed before the paper can be accepted for publication in Plant Direct.

Please view the editors' and reviewers' comments below and use their suggestions as a guide while you work on your revision.

When uploading the revised version of this article, please be sure to include the following:

-A word document that contains your response to the reviewers. You should respond to each reviewer comment and note the changes made to the manuscript. If you do not agree with a reviewer's comment and choose not to make a suggested revision, please explain why. Please try to provide as complete an answer as possible to each reviewer's criticisms in the "response to reviewers" section.

-A tracked changes document

- A clean version of the latest version of the manuscript

Comments from Academic Editor:

While this revised manuscript addresses some of the comments made by the reviewer, many significant issues remain. After careful review by the academic editor, we feel that the revised manuscript does not meet Plant Direct's publication criteria. If the authors do not substantially revise the manuscript for clarity, organization and experimental rigor, the paper will be rejected. The authors are encouraged to consider a professional editing service or other assistance to improve writing and organization of the manuscript.

The following issues must be resolved:

1. Methods: In addition to stating the package used for each analysis, the datasets and the parameters that were used should be included.

2. Kinship: I appreciate that the authors have named the VanRaden method. Please include a citation. Please also note in the text that the VanRaden method utilizes genetic markers (as stated in the Response to Reviewers but not added in the manuscript text), and importantly, the source of the genetic markers that were used (with citation).

3. QTL assignment: The objective criteria and method for identifying QTL from the statistically significant SNPs is not provided, nor is it clear why a threshold of 4 SNPs was used. As it reads, it seems that the grouping of SNPs into QTLs was arbitrary and not based on an objective method. Please either remove this QTL aspect from the manuscript or very clearly state and show results for the objective method that was used for defining QTL regions, and their statistical support. In line 188, "(6) genes identified through significant QTLs" is listed as a criterion for candidate gene selection. Does this mean QTL regions from other published studies or the QTL that were identified in this manuscript? No statistical method was stated for QTL identification, and so it is not clear what is meant by "significant QTLs". The terminology should be changed.

4. Haplotype Analyses: Please clarify the results in this section. For example, it is hard to interpret lines 317-318 that state "in which repeated SNPs (SNPs with the same LD that appeared in more than one trait) were ignored (merged in one haplotype)". In addition, it is not clear how these haplotype groups have been defined. In Figure 3, it isn't clear what the numbers in parentheses after each haplotype name represent.

5. Path analysis: It is not clear what data were used to perform this analysis or the type of output, and whether SNP data were used. Though the text in lines 290-292 suggest positive and negative effects in a range of 0-1, such effects are not presented in Table S9, which is cited. Only p-values are presented in this table, presumably from this path analysis, though the title of the table does not reference this analysis.

6. Pleiotropy and Q-TARO: Per the methods, pleiotropy was tested by using the Q-TARO database that reports rice QTLs from research publications. Per their website, it was last updated in 2008. While data from Q-TARO can be included in the paper, it needs to be clearly stated what Q-TARO provides. It seems that pleiotropy was not specifically tested in the data presented by the authors. If this paper is going to present evidence of pleiotropy, a formal analysis of their data needs to be conducted and reported.

7. Figure quality: Figure quality is poor and often text is too small to read and so data cannot be properly analyzed by the reader. Figure 3 is particularly difficult to interpret. The figure is currently titled, "Post-GWAS Results". Please rename with a descriptive title of the actual results that are being presented. Figure 3 should be improved for better visualization of the data, perhaps by focusing on only 1 chromosome and putting the remainder in supplemental figures. For the LD block figures in the middle panel of parts D and E, the titles of the panels are hard to interpret and should likely instead provide that information in the legend (and without underscores between words). It is also not clear what the numbers specify under the gene model.

8. Reorganization of the manuscript: In its current format, there doesn't seem to be a logical flow in the Results and it is difficult to follow. The section on transcription factors in the results reads like a discussion in many parts. The discussion should be trimmed and focused on a discussion of the trait data and candidate genes. The candidate gene section reads as a list of each gene and its function or potential function, instead of a cohesive discussion of the different types of candidate genes and how they are or might be associated with the biomass and stem traits.

Some other issues:

Abstract: The first half of the abstract is difficult to interpret. The first sentence states that rice is a good model for studying grass cell walls. However, the manuscript does not focus on cell wall traits. The abstract does not establish the focus of the study, which is GWAS of biomass production, and stem node and internode traits. Further, in "149 rice accessions grown out of 282 genotypes" it seems that 149 rice accessions were evaluated. This may have been from a larger panel of 282 accessions. However, since this study focuses only on 149, simply state that 149 rice accessions were evaluated (this can also be clarified in the methods). It is also hard to unpack the sentence, "GWAS identified significant SNPs (173) at 64 QTLs associated with 230 genes for 26 (of the 32 measured traits) significant traits, located in genes with a putative function in biomass production." I think this is saying that 173 statistically significant SNPs within 230 genes mapped to 64 QTLs that were associated with 26 measured traits. This sentence should be split into more than 1 sentence to clearly state the results. Finally, the last sentence of the abstract states that this is the first report of GWAS on stems of rice. The authors should consider these publications:

https://onlinelibrary.wiley.com/doi/full/10.1111/tpj.16872

https://www.pnas.org/doi/full/10.1073/pnas.1904964116#sec-3

To upload your revision, please click the link below.

https://plantdirect.msubmit.net/cgi-bin/main.plex?el=A7Lr1mu6C7pIk1I3A9ftdAXs56Iu1mCq8V108l67WHQZ

In order to provide as timely a service as possible, we ask that your revision is resubmitted within three months after receipt of this request. If an extension is needed, please send a request, along with a brief explanation, to the editorial office at plantdirect@wiley.com .

Thank you very much for giving us an opportunity to review your work. I look forward to receiving the next version.

Sincerely,

Marna Yandeau-Nelson

---------------------------------------------------------------------------- Editor comments:

While this revised manuscript addresses some of the comments made by the reviewer, many significant issues remain. After careful review by the academic editor, we feel that the revised manuscript does not meet Plant Direct's publication criteria. If the authors do not substantially revise the manuscript for clarity, organization and experimental rigor, the paper will be rejected. The authors are encouraged to consider a professional editing service or other assistance to improve writing and organization of the manuscript.

The following issues must be resolved:

1. Methods: In addition to stating the package used for each analysis, the datasets and the parameters that were used should be included.

2. Kinship: I appreciate that the authors have named the VanRaden method. Please include a citation. Please also note in the text that the VanRaden method utilizes genetic markers (as stated in the Response to Reviewers but not added in the manuscript text), and importantly, the source of the genetic markers that were used (with citation).

3. QTL assignment: The objective criteria and method for identifying QTL from the statistically significant SNPs is not provided, nor is it clear why a threshold of 4 SNPs was used. As it reads, it seems that the grouping of SNPs into QTLs was arbitrary and not based on an objective method. Please either remove this QTL aspect from the manuscript or very clearly state and show results for the objective method that was used for defining QTL regions, and their statistical support. In line 188, "(6) genes identified through significant QTLs" is listed as a criterion for candidate gene selection. Does this mean QTL regions from other published studies or the QTL that were identified in this manuscript? No statistical method was stated for QTL identification, and so it is not clear what is meant by "significant QTLs". The terminology should be changed.

4. Haplotype Analyses: Please clarify the results in this section. For example, it is hard to interpret lines 317-318 that state "in which repeated SNPs (SNPs with the same LD that appeared in more than one trait) were ignored (merged in one haplotype)". In addition, it is not clear how these haplotype groups have been defined. In Figure 3, it isn't clear what the numbers in parentheses after each haplotype name represent.

5. Path analysis: It is not clear what data were used to perform this analysis or the type of output, and whether SNP data were used. Though the text in lines 290-292 suggest positive and negative effects in a range of 0-1, such effects are not presented in Table S9, which is cited. Only p-values are presented in this table, presumably from this path analysis, though the title of the table does not reference this analysis.

6. Pleiotropy and Q-TARO: Per the methods, pleiotropy was tested by using the Q-TARO database that reports rice QTLs from research publications. Per their website, it was last updated in 2008. While data from Q-TARO can be included in the paper, it needs to be clearly stated what Q-TARO provides. It seems that pleiotropy was not specifically tested in the data presented by the authors. If this paper is going to present evidence of pleiotropy, a formal analysis of their data needs to be conducted and reported.

7. Figure quality: Figure quality is poor and often text is too small to read and so data cannot be properly analyzed by the reader. Figure 3 is particularly difficult to interpret. The figure is currently titled, "Post-GWAS Results". Please rename with a descriptive title of the actual results that are being presented. Figure 3 should be improved for better visualization of the data, perhaps by focusing on only 1 chromosome and putting the remainder in supplemental figures. For the LD block figures in the middle panel of parts D and E, the titles of the panels are hard to interpret and should likely instead provide that information in the legend (and without underscores between words). It is also not clear what the numbers specify under the gene model.

8. Reorganization of the manuscript: In its current format, there doesn't seem to be a logical flow in the Results and it is difficult to follow. The section on transcription factors in the results reads like a discussion in many parts. The discussion should be trimmed and focused on a discussion of the trait data and candidate genes. The candidate gene section reads as a list of each gene and its function or potential function, instead of a cohesive discussion of the different types of candidate genes and how they are or might be associated with the biomass and stem traits.

Some other issues:

Abstract: The first half of the abstract is difficult to interpret. The first sentence states that rice is a good model for studying grass cell walls. However, the manuscript does not focus on cell wall traits. The abstract does not establish the focus of the study, which is GWAS of biomass production, and stem node and internode traits. Further, in "149 rice accessions grown out of 282 genotypes" it seems that 149 rice accessions were evaluated. This may have been from a larger panel of 282 accessions. However, since this study focuses only on 149, simply state that 149 rice accessions were evaluated (this can also be clarified in the methods). It is also hard to unpack the sentence, "GWAS identified significant SNPs (173) at 64 QTLs associated with 230 genes for 26 (of the 32 measured traits) significant traits, located in genes with a putative function in biomass production." I think this is saying that 173 statistically significant SNPs within 230 genes mapped to 64 QTLs that were associated with 26 measured traits. This sentence should be split into more than 1 sentence to clearly state the results. Finally, the last sentence of the abstract states that this is the first report of GWAS on stems of rice. The authors should consider these publications:

https://onlinelibrary.wiley.com/doi/full/10.1111/tpj.16872

https://www.pnas.org/doi/full/10.1073/pnas.1904964116#sec-3

---------------------------------------------------------------------------- Reviewer comments:

Reviewer #3:

\# Overall

- Many claims are missing citations.

- Text often contains typos, incomplete sentences, run-on sentences, or improper formatting (e.g. missing bolding, misplacement of parentheses).

- Methods are missing adequate details to replicate experiment.

- Methods are missing proper justification or rationale.

- Results/Discussion of genetic analyses are often lacking or missing adequate justification, rationale, or interpretation.

- Methods/Results/Discussion are difficult to follow.

- Results are not ordered in a way such that the conveyance of information follows a logical flow, causing confusion and leaving the reader unsure of why or what analysis was actually performed. For example, authors identify candidate genes by proximity or inclusion of a significant SNP, conduct expression assays of candidate genes (associated with SNPs), then significant SNPs are used to construct QTL regions (regions with a significant SNPs from at least 4 traits), and then candidate genes are again identified between the first and last SNP of each QTL. As another example, Methods outlines criteria for identifying candidate genes, but identification of candidate genes within Results do no address the criteria outlined in Methods, and add new criteria such as indications of pleiotropy and protein-protein interactions. In Discussion, trait variability and heritability are both mentioned after GWAS and the number of QTLs identified in this study, before returning to the number of candidate genes.

\# Line-by-Line

- 44: missing citation for a specific FAO study or source

- 45-49: it is unclear why two metrics of rice production are listed (760 million, and 370 to 520 million tons).

- 47: missing citations for "~6 T/ha per year" and "370 to 520 tons"

- 58-59: this sentence lists the function of rice straw, and should thus be presented earlier in the paragraph.

- 60-63: this conclusion needs more justification. How do different ratios of secondary cell wall metabolites impact lodging? Has this been empirically tested before? What are "important characteristics?" Consider adding more details to logically connect lodging, rice straw, and the secondary cell wall.

- 65: Cellular elongation does not produce new cells

- Unaddressed from previous review

- 67-68: leaves are not mentioned before or after this section. Either remove mention of leaves or add details to justify their inclusion in this section

- 64-74: consider combining this paragraph with the previous paragraph. As mentioned in

- 75: What are the "node and internode traits RS" quantified?

- Unaddressed from previous review

- 76: Are the goals of this study to identify candidates for biomass production, beneficial RS composition, or limit lodging? This paragraph needs a stronger conclusion to define the goals of the study.

- Unaddressed from previous review

- 83: The way in which plants "were able to adapt and grow" is not qualified. Consider lightening this phrasing to "were grown" or something similar.

- Unaddressed from previous review

- 90: What was collected from the "the three plants with the tallest tillers?" From the way this sentence is written, I assume the main stem from was collected from all plants, and something else was collected from these three plants. Were three plants collected per subpopulation? or per genotype? What does "were selected" refer to in this context?

- Unaddressed from previous review

- 91: An overview of the traits measured in this study should be included in the introduction as well as the methods. Introduce traits immediately before "Phenotypic variation in the grown rice genotypes was estimated by measuring relevant traits using three biological replicates", prior to descriptions of harvest conditions

- 113: What is the reason for measuring internode thickness at seven different points?

- 116: Which genome or SNP assembly was used, specifically?

- Unaddressed from previous review

- 121-124: Plotting in and of itself does not characterize population structure. Was each PC calculated using genome-wide SNP genotypes, or were PCs calculated using some other measure. If so, which panel of SNPs was used for PCA? 44.1K SNPs or 34.2K SNPs? This needs to be elaborated. Based on Figure S1, there is clear separation of accessions into one of three groups. Do these groupings correspond to heterologous group or ancestry? How was clustering determined? What parameters were used for the clustering algorithm employed? It is unclear whether these principle components, nor how many, were included in the GWAS models. See further comments of Figure S1 below.

- Unaddressed from previous review

- 124: Methods must include how kinship was determined and what this kinship measures. Was kinship based on genome-wide SNP identity, or pedigree?

- Unaddressed from previous review

- 127-133: It is unclear why the authors initially elected to use two different GWAS. FarmCPU. It is unclear how many population structure covariates (PCs) were included in the MLM.

- 131-132: "due to LD between SNPs, the multi-locus model in FarmCPU." It is recommended to assess LD between SNPs prior to selecting a GWAS model. If LD between SNPs is high, then it is unclear why a single-marker model such as MLM was initially tested. If this sentence is meant to justify the use of FarmCPU over MLM following the researchers analysis of GWAS results, then this amount of detail is insufficient

- Partially addressed from previous review

- 133-135: Q-Q plots are unrelated to setting genome-wide significance thresholds, and are rather a qualitative measure of overconfidence across marker p-values, or inflation of marker significance / prevalence of false positives. What method was used to set p-value threshold? Bonferroni? What method was used to set the FDR threshold? Benjamini-Hochberg?

- Unaddressed from previous review

- 147-166: Multiple times within this section it is mentioned that analysis was performed (using some package or database), but fail to mention what each analysis actually entailed.

- Unaddressed from previous review

- 147-149: "Promoter analysis" is vague. What specific analysis was performed? Why was this analysis performed? If this analysis was performed in order to determine whether SNPs were located within promoter regions, then it is not clear from the phrase "determine the location of the significant SNPs on the identified genes associated with them."

- 159: is additive genetic variance calculated from single-marker alleles or from the entire SNP array?

- 159: It is unclear what "haplotype analysis" refers to or entails.

- 164: It is unclear what "path analysis" refers to or entails.

- 165: it is unclear what "analysis of epistasis" refers to or entails.

- 173: What is the threshold for grouping SNPs? LD? Physical distance?

- 173-175: It is not clear how QTL (or hotspot) regions were constructed. A hotspot containing SNPs associated with multiple traits is an indication of pleiotropy (not strength of association) and it is inappropriate to use the word "significant" when no formal statistical test is performed. Why was a threshold of 4 traits for a hotspot chosen? Since the threshold for "QTL significance" is arbitrarily set by the researchers, this needs to be justified.

- 175-179: It is not clear why the authors refer to both candidate genes (identified by proximity to or inclusion of SNPs) and significant SNPs are differentiated, e.g. "SNPs that overlap with genomic regions (i.e., their associated SNPs)." The phrase "were annotated" could refer to either function annotation or gene name annotation.

- 179-180: What is the justification for discarding these sequences? Is there evidence that these regions are not transcribed?

- 180: It is unclear what a information the researchers attempted to collect following their literature search, be it gene function, transcription level, translation evidence, homology, etc.

- 188: It is unclear how "genes identified through significant QTLs" is different from genes identified through significant SNPs. If this is referring to significant "QTLs" (hotspots SNPs from different traits), it is unclear how large these regions may be, and thus whether the genes contained within these regions are worth investigating

- 182-190: There are multiple criteria listed for SNP/gene prioritization due to potential function in cell wall development or biomass production, including 1) "genes with significant SNPs and functional annotation related to biomass or cell wall biology", 2) "genes whose function was previously identified in rice", 3) "similar genes in other species having a known role in biomass production or cell wall biology", 4) "genes obtained via annotations of significant SNPs conducted using SNPEff based on the relevance of their biological function to biomass production and cell wall biology"

- Consider condensing and simplifying these criteria into a single phrase

- 182-190: In is unclear whether these criteria are ordered by importance, or if certain criteria are more indicative of potential biological significance

- 196-199: This is not an adequate test for pleiotropy, owing to multiple testing and correlated traits. Consider using a multivariate multiple-testing adjustment, such as ASH or MASH, or a multi-trait GWAS algorithm. It is unclear how QT information from Q-TARO was utilized in this study

- Unaddressed from previous review

- 211: Do these groupings correspond to heterologous group or ancestry?

- 217-227: Why is -log10(P) > 4 used here instead of FDR < 0.05, as mentioned earlier in methods?

- Unaddressed from previous reviews

- 226-227: "Promoter analysis of putative candidate genes." This sentence is incomplete

- 235: "Due to some were. reported for different traits, they were located in the promoter region of different genes ..." I do not understand this sentence

- 242-243: Missing citation

- 243-246: Missing citation

- 248-251: Missing citation

- 254-256: Missing citation

- 267-268: Missing citation

- 268-270: Missing citation

- 273-274: Missing citation

- 280-281: Missing citation

- 281: TFs do not have phenotypes; rather, overexpression lines of these TFs do.

- 287-297: It is not clear why path analysis (a phenotypic assay) is performed post-GWAS, as this section does not include how the results of path analysis relate to "genetic attributes." Consider moving this section to the start of results, so as to convey to readers the importance of these traits on biomass prior to discussion on candidate genes

- 304: "SNPs that have greater thickness" should refer to the lines in the figure that connect or join SNPs around the circular plot, not the SNPs themselves.

- 305-308: It is unclear whether epistasis was conducted for ALL SNPs within some window including non-significant SNPs (e.g. 1 kb, here referred to as a QTL) or only significant SNPs within that region. If ALL SNPs were included in this analysis, provide justification for excluding them from further investigation when the -log10(p) < 4, as epistasis analysis may identify important genetic elements which were not identified by GWAS.

- 309-311: Are the "strongest epistatic interactions" determined by significance, effect size, or number of SNP-SNP interactions (as stated at the end)?

- 317-318: I assume "SNPs with the same LD that appeared in more than one trait" is referring to SNPs in high LD with a candidate SNP (on a per trait basis). As written, it is unclear how this analysis was actually performed. If SNPs were merged into one haplotype, then it is inappropriate to say they were ignored.

- 315-323: Why is haplotype analysis performed post-GWAS? The most common use of haplotype analysis for quantitative genetic studies is to identify regions of the genome that are associated with a trait of interest which would otherwise be insignificantly or weakly associated in a single-SNP analysis. If this analysis is performed for another reason, it is not clear.

- 321: What are the "three main haplotypes"?

- 323: Incorrect parenthesis placement

- 332: Missing LD threshold for experiment

- 389-391: "genes where we had identified significant SNPs" and "located in either gene or promoter regions" are redundant

- 392-395: The sentence including an example of high stem expression and the following sentence contradict

- 398: It is inappropriate to use the word "significant" when no formal statistical test is performed.

- 399: This is not an adequate test for pleiotropy.

- 404-406: It is not clear which candidate genes were associated with individual makers or included within a hotspot.

- 434-435: Missing citation

- 441-444: Missing citation

- 458-462: Importance of GWAS should be discussed before the importance of Multi-trait GWAS and meta-analysis

- 471-480: Trait variability and heritability should be mentioned prior to GWAS and the number of QTLs identified in this study.

- 491: How is "most important interactions" quantified?

- Discussion: Consider grouping discussion by gene "classes" (common function), and condense 4.1 and 4.2 (which cover the importance of methods) into Methods/Results. Additional information regarding candidate genes in 4.1 and 4.2 should be discussed in with subsections of each candidate genes -- such that information pertaining to a single candidate gene is not spread throughout different sections / subsections of the Discussion.

Decision Letter Round 4:

December 7, 2025

Pär K Ingvarsson

Sveriges lantbruksuniversitet

Plant biology

Box 7080

Uppsala, N/A 71507

Sweden

RE: Analysis of genetic variation of rice straw characteristics and its influence on biomass

Dear Dr. Ingvarsson:

Thank you for submitting to Plant Direct. All required reviews have been returned and we have now finished our evaluation of your manuscript. In light of the reviewers' and editor's comments, further revisions are needed before the paper can be accepted for publication in Plant Direct.

Please view the editors' and reviewers' comments below and use their suggestions as a guide while you work on your revision.

When uploading the revised version of this article, please be sure to include the following:

-A word document that contains your response to the reviewers. You should respond to each reviewer comment and note the changes made to the manuscript. If you do not agree with a reviewer's comment and choose not to make a suggested revision, please explain why. Please try to provide as complete an answer as possible to each reviewer's criticisms.

-A tracked changes document with each change highlighted

- A clean version of the latest version of the manuscript

Comments from Academic Editor:

The revised submission is greatly improved. After careful review by the academic editor and consideration of the reviewer's comments, we feel that the revised manuscript requires minor revisions. A revised manuscript should address the following:

1. Grammar: Please read carefully for minor grammar issues and missing words. For example, the title of Section 3.3.1 reads "Analysis of Promoter Regions Putative Candidate Genes". The word "of" is missing.

2. Table 3: Some rows in the table have more than one gene listed in the Candidate Gene ID Column, and only one name in the Candidate Gene Name column. It is unclear whether the genes are orthologs and hence have only one gene name or if only one gene is listed inadvertently. Please clarify in the table. Also, LOC_Os11g36560 is listed twice.

3. References to Multi-trait GWAS: Although the text states that multi-trait GWAS was performed, the methods do not state a multi-trait GWAS method. Multi-trait GWAS uses a single model to look at multiple traits simultaneously. If that was performed, clearly state these methods.

4. Discussion on lodging: Please adjust the title of Discussion section 4.2, as this study did not directly test lodging resistance, nor were candidate genes specifically associated with lodging. If there indeed are candidate genes previously associated with lodging in other studies, this section of the discussion could be modified to specifically note these genes and their published associations with lodging resistance. Also, the sentence (and those like it) that state "Taken together, our findings provide a comprehensive genetic framework underlying lignocellulosic biomass production and its relationship with lodging resistance in rice." should be softened as this study does not provide a proven genetic framework for lodging resistance.

To upload your revision, please click the link below.

https://plantdirect.msubmit.net/cgi-bin/main.plex?el=A3Lr5mu4D4pIk3I4A9ftdAXs56Iu1mCq8V108l67WHQZ

In order to provide as timely a service as possible, we ask that your revision is resubmitted within three months after receipt of this request. If an extension is needed, please send a request, along with a brief explanation, to the editorial office at plantdirect@wiley.com .

Please note that, in addition to publishing reviewer comments, the author's responses to review comments will also be published alongside the final version of the paper. If you would not like the author's responses to be published, please contact the editorial office at plantdirect@wiley.com .

Thank you very much for giving us an opportunity to review your work. I look forward to receiving the next version.

Sincerely,

Marna Yandeau-Nelson

Gustavo MacIntosh

Editor, Plant Direct

---------------------------------------------------------------------------- Editor comments:

The revised submission is greatly improved. After careful review by the academic editor and consideration of the reviewer's comments, we feel that the revised manuscript requires minor revisions. A revised manuscript should address the following:

1. Grammar: Please read carefully for minor grammar issues and missing words. For example, the title of Section 3.3.1 reads "Analysis of Promoter Regions Putative Candidate Genes". The word "of" is missing.

2. Table 3: Some rows in the table have more than one gene listed in the Candidate Gene ID Column, and only one name in the Candidate Gene Name column. It is unclear whether the genes are orthologs and hence have only one gene name or if only one gene is listed inadvertently. Please clarify in the table. Also, LOC_Os11g36560 is listed twice.

3. References to Multi-trait GWAS: Although the text states that multi-trait GWAS was performed, the methods do not state a multi-trait GWAS method. Multi-trait GWAS uses a single model to look at multiple traits simultaneously. If that was performed, clearly state these methods.

4. Discussion on lodging: Please adjust the title of Discussion section 4.2, as this study did not directly test lodging resistance, nor were candidate genes specifically associated with lodging. If there indeed are candidate genes previously associated with lodging in other studies, this section of the discussion could be modified to specifically note these genes and their published associations with lodging resistance. Also, the sentence (and those like it) that state "Taken together, our findings provide a comprehensive genetic framework underlying lignocellulosic biomass production and its relationship with lodging resistance in rice." should be softened as this study does not provide a proven genetic framework for lodging resistance.

---------------------------------------------------------------------------- Reviewer comments:

Reviewer #3:

SUMMARY

Genetic diversity of rice straw characteristics is understudied, particularly for biomass. In total 26 of 32 total traits contained significant SNPs, associated with genes purportedly regulating biomass: including 1) cytoskeletal and cell wall components, 2) growth and development, 3) cell wall biosynthesis, 4) cell wall regulation, and 4) cell wall modification. In addition, genes could be associated with 5 significant haplogroups. Intertrait correlation analysis revealed high positive associations between panicle dry weight, internode dry weight, and biomass. Overall, the authors hope these findings contribute to increased biomass production in rice.

COMMENTS

Overall:

- Several sections are missing clear or obvious citations, generally pertaining purported candidate gene function.

- Discussion sections often repeat the importance and validity of GWAS as a general research approach where it is otherwise unnecessary (as GWAS is both widely used and well accepted).

- Lodging resistance is only referenced in the Introduction and Discussion sections, whereas biomass (the trait of study) is referenced and supported throughout with many citations. Despite this, lodging resistance plays a major role in the Conclusions section. If the authors intend to connect lodging resistance to their traits of interest, consider discussing how individual candidate genes have been associated or could be directly associated with lodging.

o Lines mentioning lodging: 67, 69-76, 453 (single citation), 619-643.

Results:

- 219: Broad-sense heritability does not seem to be reported in Table S3

- 221: These are traits which contribute to biomass, as this sentence does not report the heritability of biomass as its own trait.

- 223: It is unclear why 26 of 32 traits were analyzed from this paragraph alone

- 229 & Figure S1: PCA itself does not define clusters or subpopulations. Figure legend should indicate how clusters were defined, or what these heterologous subpopulations are defined as in rice.

- 255: Please indicate which traits these TFs were associated with.

- 257-261: Not cited.

- 272-274: Are these findings from Wei et al 2021? If so, include additional citation for these two statements.

- 3.4.1 Path Analysis: Results of path analysis, and correlation of traits should be discussed before genetic analyses, as highly correlated traits and significantly impact strength of findings. As written, it does not appear that path analysis integrates or accounts for any genetic component.

- Figure S3: Distributions of phenotypes should be reported and discussed prior to candidate gene identification, as the shape of these distributions should be considered prior to GWAS or statistical analysis.

- 3.5.1 PPI Network: Creation and analysis of the PPI network were not discussed in Methods

Discussion:

- 462-468: This paper does not analyze pleiotropy, so it is confusing why this section on the benefits of multi-trait GWAS has been included.

Decision Letter Round 5:

December 17, 2025

Pär K Ingvarsson

Sveriges lantbruksuniversitet

Plant biology

Box 7080

Uppsala, N/A 71507

Sweden

MSID: 2025-01721R4

MS TITLE: Analysis of genetic variation of rice straw characteristics and its influence on biomass

Dear Dr. Pär Ingvarsson:

I am pleased to inform you that your manuscript "Analysis of genetic variation of rice straw characteristics and its influence on biomass" has been accepted for publication in Plant Direct.

Your article will appear online in the next available issue of Plant Direct. To ensure your article gets published as quickly as possible, please pay attention to the steps detailed below. We have found that most of the delays happen at this stage, especially at the payment stage, so please respond as quickly as possible when prompted.

License Agreement: Once your article has been accepted it will move to Production and undergo admin and file checking - you may receive an email with any queries we have at this stage. When all required items are received by the publisher and queries resolved, the corresponding author will receive an email from Wiley's Author Services system which will ask them to log on at https://authorservices.wiley.com/bauthor and will present them with the appropriate license for completion. Your article cannot be published until both the signed license agreement and payment of the article fee have been received.

Payment of the Open Access Article Publication Fee: All articles published in Plant Direct are fully open access: immediately and freely available to read, download and share. Plant Direct charges a publication fee to cover the publication costs. The corresponding author for this manuscript should have already received a quote from the payments team (cs-openaccess@wiley.com) with the estimated article publication fee; please email cs-openaccess@wiley.com if this has not been received. The corresponding author should log on to the Wiley Author Services site, where the publication fee can be paid by credit card or an invoice. Pro Forma can also be requested. Payment of the publication charge must be received before the article will be published online.

Proofs: You will have the opportunity to look over your paper once more when you receive the author proofs for your article. The proofs will be with you in approximately two weeks. Please note that, in addition to publishing reviewer comments, the author's responses to review comments will also be published alongside the final version of the paper. If you would not like the author's responses to be published, please contact the editorial office at plantdirect@wiley.com .

Promotion of your article: You can help your research get the attention it deserves! Check out Wiley's free Promotion Guide for best-practice recommendations for promoting your work at www.wileyauthors.com/eeo/guide. And learn more about Wiley Editing Services which offers professional video, design, and writing services to create shareable video abstracts, infographics, conference posters, lay summaries, and research news stories for your research at www.wileyauthors.com/eeo/promotion.

Thank you again for your contribution to Plant Direct. If you have any questions, feel free to contact the editorial office at plantdirect@wiley.com .

Sincerely,

Marna Yandeau-Nelson

Gustavo MacIntosh

Editor, Plant Direct

---------------------------------------------------------------------------- Editor comments

We thank the authors for submitting a revised manuscript that addressed the reviewer and academic editor's comments and concerns. This manuscript meets Plant Direct's publication criteria, and we are pleased to publish this work.

---------------------------------------------------------------------------- Reviewer comments:

Response letter Round 1:

In the manuscript by Mohamadiaza et al., entitled “Analysis of genetic variation of rice

straw charachteristics and its influence on biomass and stem lodging,” authors used a

GWA approach to identify candidate genes for biomass and stalk lodging resistance.

Overall, the methods are sound, and the manuscript is well written. Below I outline

minor comments that would improve the manuscript.

 The abbreviation table and use of abbreviations throughout the manuscript is

distracting and confusing. These abbreviations are not always intuitive and make

the interpretability of results difficult. While abbreviations are OK for figures and

tables (with the abbreviation spelled out in the legend), I suggest writing out the

traits in the manuscript for ease of interpretability.

 The manuscript wants to link biomass and lodging, however, lodging data was

not collected. I think this is a reach and can be discussed in the discussion,

however, as the manuscript stands now the authors refer to lodging when they

lack data to support this.

o There are many examples throughout, however one includes on line 189-

190 and a second example on lines 287-288.

 Line 70: "area at the end of the stem” this is confusing. I suggest being more

specific and possibly referencing Figure 1. The area of the stem closest to the

ground perhaps?

 Experimental Design/Section 2.1:

o Line 86: How many plants were in a plot?

o Line 90: Were the three biological replicates = to three total plants? Were

these three plants from a single plot? Was this one plant from each plot

and three plots total?

o Line 91: What does “five piles of plants from each accession” mean? Five

plants per plot?

o How long after harvest were these traits measured? I would expect node

and internode diameters to change (not uniformly) due to drying/storage at

25 degrees C.

 Lines 141-149:

o Were all of these candidate genes associated with a significant SNP with

a-log10(p) > 4? Or was this just true for #3 in the list?

o Was a single gene for each SNP chosen? Or was it possible that more

than one candidate gene was chosen if it fit the criteria listed in this

section?

 Line 168/169: Error with this citation generation.

 Line 170 (and elsewhere): What was included in your model as fixed and random

effects? For example, how did you calculate heritability?

 Line 174: Why was 300 kb chosen as a window size? Is this a standard size for

rice?

 Line 206: The W6 trait has the strongest association. However, W6 also has a lot

of genetic variation (Line 191 and 192), while also having multiple SNPs across

multiple chromosomes having an effect (Figure 3B). I think this is worth

elaborating on in the discussion.

 Line 215 and Table S9: Where did these 11 highlighted SNPs come from? How

were these chosen. It is confusing how above (Line 207) 173 significant SNPs

were discussed and then you end up discussing 11 a few lines later. At this point

it becomes confusing the criteria used to select these. You discuss on lines 141-

149 that multiple criteria were considered from selecting candidate genes. It

might make more sense to break these results into sections to clearly explain

how different results were determined.

 Discussion (overall) reads like a laundry list and is not very organized. I suggest

reframing this to highlight the goals of this project and how your results

(collectively) fill these gaps with support from additional studies. Many traits were

tested in this experiment (and many of which are correlated), so going through

each class of genes and significant associations (as it is now) makes it difficult to

collectively synthesize the results and findings in a greater context.

 Figure 1: The legend and the figure do not match. Specifically, the legend

illustrates the internode length getting larger from bottom (internode 4) to top

(internode 1). Yet the legend does not describe these size comparisons this way.

 Figure 2: This does not need to be a main body figure, in my opinion. This adds

no context to the manuscript/methods that can not be described in the text.

 The overall quality of Figures is poor but maybe this is a result of PDF generation

during submission?

 Figure 3: How does the information in panel A and panel B differ? What is found

in one that is not found in the other? I don’t think these are both necessary for a

main body figure.

 Figure 4: The quality of this figure/text size made it impossible to evaluate.

However, I suspect that this is not necessary for a main body figure. What is the

take home here? What is needed for the main body and what can be moved to

supplement?

 Figure 5: I was also unable to evaluate this due to text size and quality. Same

comment as above.

 Figure S1: What do the colors mean?

Response Letter Round 2:

In the manuscript by Mohamadiaza et al., entitled “Analysis of genetic variation of rice

straw charachteristics and its influence on biomass and stem lodging,” authors used a

GWA approach to identify candidate genes for biomass and stalk lodging resistance.

Overall, the methods are sound, and the manuscript is well written. Below I outline

minor comments that would improve the manuscript.

 The abbreviation table and use of abbreviations throughout the manuscript is

distracting and confusing. These abbreviations are not always intuitive and make

the interpretability of results difficult. While abbreviations are OK for figures and

tables (with the abbreviation spelled out in the legend), I suggest writing out the

traits in the manuscript for ease of interpretability.

 The manuscript wants to link biomass and lodging, however, lodging data was

not collected. I think this is a reach and can be discussed in the discussion,

however, as the manuscript stands now the authors refer to lodging when they

lack data to support this.

o There are many examples throughout, however one includes on line 189-

190 and a second example on lines 287-288.

 Line 70: "area at the end of the stem” this is confusing. I suggest being more

specific and possibly referencing Figure 1. The area of the stem closest to the

ground perhaps?

 Experimental Design/Section 2.1:

o Line 86: How many plants were in a plot?

o Line 90: Were the three biological replicates = to three total plants? Were

these three plants from a single plot? Was this one plant from each plot

and three plots total?

o Line 91: What does “five piles of plants from each accession” mean? Five

plants per plot?

o How long after harvest were these traits measured? I would expect node

and internode diameters to change (not uniformly) due to drying/storage at

25 degrees C.

 Lines 141-149:

o Were all of these candidate genes associated with a significant SNP with

a-log10(p) > 4? Or was this just true for #3 in the list?

o Was a single gene for each SNP chosen? Or was it possible that more

than one candidate gene was chosen if it fit the criteria listed in this

section?

 Line 168/169: Error with this citation generation.

 Line 170 (and elsewhere): What was included in your model as fixed and random

effects? For example, how did you calculate heritability?

 Line 174: Why was 300 kb chosen as a window size? Is this a standard size for

rice?

 Line 206: The W6 trait has the strongest association. However, W6 also has a lot

of genetic variation (Line 191 and 192), while also having multiple SNPs across

multiple chromosomes having an effect (Figure 3B). I think this is worth

elaborating on in the discussion.

 Line 215 and Table S9: Where did these 11 highlighted SNPs come from? How

were these chosen. It is confusing how above (Line 207) 173 significant SNPs

were discussed and then you end up discussing 11 a few lines later. At this point

it becomes confusing the criteria used to select these. You discuss on lines 141-

149 that multiple criteria were considered from selecting candidate genes. It

might make more sense to break these results into sections to clearly explain

how different results were determined.

 Discussion (overall) reads like a laundry list and is not very organized. I suggest

reframing this to highlight the goals of this project and how your results

(collectively) fill these gaps with support from additional studies. Many traits were

tested in this experiment (and many of which are correlated), so going through

each class of genes and significant associations (as it is now) makes it difficult to

collectively synthesize the results and findings in a greater context.

 Figure 1: The legend and the figure do not match. Specifically, the legend

illustrates the internode length getting larger from bottom (internode 4) to top

(internode 1). Yet the legend does not describe these size comparisons this way.

 Figure 2: This does not need to be a main body figure, in my opinion. This adds

no context to the manuscript/methods that can not be described in the text.

 The overall quality of Figures is poor but maybe this is a result of PDF generation

during submission?

 Figure 3: How does the information in panel A and panel B differ? What is found

in one that is not found in the other? I don’t think these are both necessary for a

main body figure.

 Figure 4: The quality of this figure/text size made it impossible to evaluate.

However, I suspect that this is not necessary for a main body figure. What is the

take home here? What is needed for the main body and what can be moved to

supplement?

 Figure 5: I was also unable to evaluate this due to text size and quality. Same

comment as above.

 Figure S1: What do the colors mean?

Response Letter Round 3:

In the manuscript by Mohamadiaza et al., entitled “Analysis of genetic variation of rice

straw charachteristics and its influence on biomass and stem lodging,” authors used a

GWA approach to identify candidate genes for biomass and stalk lodging resistance.

Overall, the methods are sound, and the manuscript is well written. Below I outline

minor comments that would improve the manuscript.

 The abbreviation table and use of abbreviations throughout the manuscript is

distracting and confusing. These abbreviations are not always intuitive and make

the interpretability of results difficult. While abbreviations are OK for figures and

tables (with the abbreviation spelled out in the legend), I suggest writing out the

traits in the manuscript for ease of interpretability.

 The manuscript wants to link biomass and lodging, however, lodging data was

not collected. I think this is a reach and can be discussed in the discussion,

however, as the manuscript stands now the authors refer to lodging when they

lack data to support this.

o There are many examples throughout, however one includes on line 189-

190 and a second example on lines 287-288.

 Line 70: "area at the end of the stem” this is confusing. I suggest being more

specific and possibly referencing Figure 1. The area of the stem closest to the

ground perhaps?

 Experimental Design/Section 2.1:

o Line 86: How many plants were in a plot?

o Line 90: Were the three biological replicates = to three total plants? Were

these three plants from a single plot? Was this one plant from each plot

and three plots total?

o Line 91: What does “five piles of plants from each accession” mean? Five

plants per plot?

o How long after harvest were these traits measured? I would expect node

and internode diameters to change (not uniformly) due to drying/storage at

25 degrees C.

 Lines 141-149:

o Were all of these candidate genes associated with a significant SNP with

a-log10(p) > 4? Or was this just true for #3 in the list?

o Was a single gene for each SNP chosen? Or was it possible that more

than one candidate gene was chosen if it fit the criteria listed in this

section?

 Line 168/169: Error with this citation generation.

 Line 170 (and elsewhere): What was included in your model as fixed and random

effects? For example, how did you calculate heritability?

 Line 174: Why was 300 kb chosen as a window size? Is this a standard size for

rice?

 Line 206: The W6 trait has the strongest association. However, W6 also has a lot

of genetic variation (Line 191 and 192), while also having multiple SNPs across

multiple chromosomes having an effect (Figure 3B). I think this is worth

elaborating on in the discussion.

 Line 215 and Table S9: Where did these 11 highlighted SNPs come from? How

were these chosen. It is confusing how above (Line 207) 173 significant SNPs

were discussed and then you end up discussing 11 a few lines later. At this point

it becomes confusing the criteria used to select these. You discuss on lines 141-

149 that multiple criteria were considered from selecting candidate genes. It

might make more sense to break these results into sections to clearly explain

how different results were determined.

 Discussion (overall) reads like a laundry list and is not very organized. I suggest

reframing this to highlight the goals of this project and how your results

(collectively) fill these gaps with support from additional studies. Many traits were

tested in this experiment (and many of which are correlated), so going through

each class of genes and significant associations (as it is now) makes it difficult to

collectively synthesize the results and findings in a greater context.

 Figure 1: The legend and the figure do not match. Specifically, the legend

illustrates the internode length getting larger from bottom (internode 4) to top

(internode 1). Yet the legend does not describe these size comparisons this way.

 Figure 2: This does not need to be a main body figure, in my opinion. This adds

no context to the manuscript/methods that can not be described in the text.

 The overall quality of Figures is poor but maybe this is a result of PDF generation

during submission?

 Figure 3: How does the information in panel A and panel B differ? What is found

in one that is not found in the other? I don’t think these are both necessary for a

main body figure.

 Figure 4: The quality of this figure/text size made it impossible to evaluate.

However, I suspect that this is not necessary for a main body figure. What is the

take home here? What is needed for the main body and what can be moved to

supplement?

 Figure 5: I was also unable to evaluate this due to text size and quality. Same

comment as above.

 Figure S1: What do the colors mean?

Response Letter Round 4:

In the manuscript by Mohamadiaza et al., entitled “Analysis of genetic variation of rice

straw charachteristics and its influence on biomass and stem lodging,” authors used a

GWA approach to identify candidate genes for biomass and stalk lodging resistance.

Overall, the methods are sound, and the manuscript is well written. Below I outline

minor comments that would improve the manuscript.

 The abbreviation table and use of abbreviations throughout the manuscript is

distracting and confusing. These abbreviations are not always intuitive and make

the interpretability of results difficult. While abbreviations are OK for figures and

tables (with the abbreviation spelled out in the legend), I suggest writing out the

traits in the manuscript for ease of interpretability.

 The manuscript wants to link biomass and lodging, however, lodging data was

not collected. I think this is a reach and can be discussed in the discussion,

however, as the manuscript stands now the authors refer to lodging when they

lack data to support this.

o There are many examples throughout, however one includes on line 189-

190 and a second example on lines 287-288.

 Line 70: "area at the end of the stem” this is confusing. I suggest being more

specific and possibly referencing Figure 1. The area of the stem closest to the

ground perhaps?

 Experimental Design/Section 2.1:

o Line 86: How many plants were in a plot?

o Line 90: Were the three biological replicates = to three total plants? Were

these three plants from a single plot? Was this one plant from each plot

and three plots total?

o Line 91: What does “five piles of plants from each accession” mean? Five

plants per plot?

o How long after harvest were these traits measured? I would expect node

and internode diameters to change (not uniformly) due to drying/storage at

25 degrees C.

 Lines 141-149:

o Were all of these candidate genes associated with a significant SNP with

a-log10(p) > 4? Or was this just true for #3 in the list?

o Was a single gene for each SNP chosen? Or was it possible that more

than one candidate gene was chosen if it fit the criteria listed in this

section?

 Line 168/169: Error with this citation generation.

 Line 170 (and elsewhere): What was included in your model as fixed and random

effects? For example, how did you calculate heritability?

 Line 174: Why was 300 kb chosen as a window size? Is this a standard size for

rice?

 Line 206: The W6 trait has the strongest association. However, W6 also has a lot

of genetic variation (Line 191 and 192), while also having multiple SNPs across

multiple chromosomes having an effect (Figure 3B). I think this is worth

elaborating on in the discussion.

 Line 215 and Table S9: Where did these 11 highlighted SNPs come from? How

were these chosen. It is confusing how above (Line 207) 173 significant SNPs

were discussed and then you end up discussing 11 a few lines later. At this point

it becomes confusing the criteria used to select these. You discuss on lines 141-

149 that multiple criteria were considered from selecting candidate genes. It

might make more sense to break these results into sections to clearly explain

how different results were determined.

 Discussion (overall) reads like a laundry list and is not very organized. I suggest

reframing this to highlight the goals of this project and how your results

(collectively) fill these gaps with support from additional studies. Many traits were

tested in this experiment (and many of which are correlated), so going through

each class of genes and significant associations (as it is now) makes it difficult to

collectively synthesize the results and findings in a greater context.

 Figure 1: The legend and the figure do not match. Specifically, the legend

illustrates the internode length getting larger from bottom (internode 4) to top

(internode 1). Yet the legend does not describe these size comparisons this way.

 Figure 2: This does not need to be a main body figure, in my opinion. This adds

no context to the manuscript/methods that can not be described in the text.

 The overall quality of Figures is poor but maybe this is a result of PDF generation

during submission?

 Figure 3: How does the information in panel A and panel B differ? What is found

in one that is not found in the other? I don’t think these are both necessary for a

main body figure.

 Figure 4: The quality of this figure/text size made it impossible to evaluate.

However, I suspect that this is not necessary for a main body figure. What is the

take home here? What is needed for the main body and what can be moved to

supplement?

 Figure 5: I was also unable to evaluate this due to text size and quality. Same

comment as above.

 Figure S1: What do the colors mean?
